# O-GlcNAcylation and stablization of SIRT7 promote pancreatic cancer progression by blocking the SIRT7-REGγ interaction

**DOI:** 10.1038/s41418-022-00984-3

**Published:** 2022-04-14

**Authors:** Xiaoman He, Yongzhou Li, Qing Chen, Lei Zheng, Jianyao Lou, Chuanshuai Lin, Jiali Gong, Yi Zhu, Yulian Wu

**Affiliations:** 1 Department of Hepato-Pancreato-Biliary Surgery, The Second Affiliated Hospital, Zhejiang University School of Medicine, Hangzhou, People’s Republic of China; 2 Key Laboratory of Cancer Prevention and Intervention, China National Ministry of Education, Cancer Institute, The Second Affiliated Hospital, Zhejiang University School of Medicine, Hangzhou, Zhejiang People’s Republic of China; 3 The Affiliated Hospital of Qingdao University, Qingdao, People’s Republic of China

**Keywords:** Oncogenes, Glycobiology

## Abstract

Pancreatic ductal adenocarcinoma (PDAC) is one of the most lethal cancers and its dismal prognosis indicates the urgent need to elucidate the potential oncogenic mechanisms. SIRT7 is a classic NAD^+^-dependent deacetylase that stabilizes the transformed state of cancer cells. However, its functional roles in PDAC are still unclear. Here, we found that SIRT7 expression is upregulated and predicts poor prognosis in PDAC. Then we screened the new interacting proteins of SIRT7 by mass spectrometry and the results showed that SIRT7 can interact with O-GlcNAc transferase (OGT). O-GlcNAcylation stabilizes the SIRT7 protein by inhibiting its interaction with REGγ to prevent degradation, and hyper-O-GlcNAcylation in pancreatic cancer cells leads to hypoacetylation of H3K18 via SIRT7, which promotes transcriptional repression of several tumour suppressor genes. In addition, SIRT7 O-GlcNAcylation at the serine 136 residue (S136) is required to maintain its protein stability and deacetylation ability. In vivo and in vitro experiments showed that blocking SIRT7 O-GlcNAcylation at S136 attenuates tumour progression. Collectively, we demonstrate that O-GlcNAcylation is an important post-translational modification of SIRT7 in pancreatic cancer cells, and elucidating this mechanism of SIRT7 is expected to pave the way for the development of novel therapeutic methods in the future.

## Introduction

Pancreatic ductal adenocarcinoma (PDAC) is the seventh leading cause of cancer-related death [1]. It is an aggressive disease with a poor clinical prognosis. Remarkable progress has been made in fundamental research over the past century, including gene mutation, tumour-stromal crosstalk and post-transcriptional modification. Despite these advances, PDAC remains a deadly disease [2, 3]. Hence, we need to further explore more biological mechanisms that contribute to the development and progression of pancreatic tumours.

Sirtuins, are a highly conserved family of NAD^+^-dependent deacetylases that target both histone and nonhistone proteins [4]. Mammalian sirtuins have been linked to a wide range of biological processes, such as cellular stress resistance, tumorigenesis and energy metabolism [5]. The importance of sirtuins is demonstrated by their role in various age-related diseases, including cancer, diabetes, and neurodegenerative disease [6]. To date, the sirtuin family consists of seven members (SIRT1–SIRT7) in mammals, which have different functions and cellular localizations [7].

SIRT7, which is primarily located in the nucleolus, binds to the ribosomal RNA (rRNA) gene and participates to the process of rDNA transcription during mitosis [8]. Recently, increasing evidence has demonstrated that SIRT7 expression is altered in many human cancers, which suggests its important functions in various cellular events with a potential impact on oncogenic transformation and tumour biology [9, 10]. An important cellular function of SIRT7 is regulation of the chromatin remodelling: it catalyses the selective deacetylation of lysine 18 on histone H3 (H3K18), an emerging histone biomarker of aggressive tumours and poor clinical outcome in patients with cancer. As a result, H3K18 hypoacetylation at the promoter loci of several tumour-suppressive genes can stabilize the transformed state of cancer cells [11, 12]. In addition, SIRT7 regulates rRNA and tRNA synthesis, which ultimately leads to an enhancement of ribosome biogenesis necessary for tumour cell growth and proliferation [13]. However, abolishment of SIRT7 function can reverse the transformed phenotype of cancer cells and reduce their metastasis in vivo [14]. These findings suggest that SIRT7 is a potential therapeutic target for epigenetic cancer therapy.

O-GlcNAcylation is an important modification for regulating numerous nuclear and cytoplasmic proteins. The O-GlcNAc groups are attached to serine or threonine hydroxyl moieties of the proteins under the catalysis of O-GlcNAc transferase (OGT) [15]. O-GlcNAc signalling is a considerable crosslink between epigenetics and cellular metabolism [16]. Emerging studies indicate that the levels of total O-GlcNAcylation are altered in cancer cells and that aberrant protein O-GlcNAcylation can control key signalling and metabolic pathways by modulating protein stability, polymerization or interaction [17–19]. In particular, OGT can interact with SIRT1 and catalyse the O-GlcNAcylation modification at Serine 549 under stress, which directly increases its deacetylase activity [20]. However, the relationship between O-GlcNAcylation and SIRT7 in cancer cells is poorly studied.

In this study, we demonstrate a connection between SIRT7 and OGT, providing an unexpected link between nutrient sensor O-GlcNAcylation and H3K18 acetylation in pancreatic cancer cells.

## Materials and methods

### Immunohistochemistry staining and scoring standard

We detected the expression of SIRT7 in pancreatic cancer tissue microarrays which contained pancreatic cancer and adjacent tissues by immunohistochemistry (IHC). The pancreatic cancer tissue microarrays were purchased from Shanghai Outdo Biotech Co., Ltd. (CAT: HPan-Ade180Sur-02). Informed consent was obtained from all subjects. Tissue samples on the microarrays from 56 patients (unqualified cases were eliminated) with pancreatic cancer were examined. The tissues were dewaxed and subjected to peroxidase blocking. Then, the tissues were subjected to antigen retrieval, primary antibody incubation (anti-SIRT7, 1:100, ab259968, Abcam), secondary antibody incubation, DAB (diaminobenzidine) staining, counterstaining of cell nuclei and dehydration. The immunohistochemical staining of SIRT7 was evaluated by two experienced pathologists in a double-blinded manner. The percentage of stained area was divided into four grades: 0–25%, 26–50%, 51–75% and 76–100%, which were assigned as 1 point, 2 points, 3 points, and 4 points, respectively. The staining intensity was also divided into four parts: negative (score 0), weak (score 1), moderate (score 2) and strong (score 3). Subsequently, SIRT7 expression was calculated as the value of percentage of stained area score x staining intensity score, which ranged from 0 to 12. The final expression level of SIRT7 was defined as ‘low’ (0–6) and ‘high’ (7–12). The study was approved by the Second Affiliated Hospital of the School of Medicine of Zhejiang University Review Board and the ethics committees of Zhejiang University.

### Proximity ligation assay

Tissues on slides were fixed, permeabilized, deparaffinized and antigen retrieved. After incubation with anti-SIRT7 (1:100, MA5-31904, Invitrogen) and anti-OGT (1:50, 24083, CST) overnight at 4 °C, probes were added to the slides according to the manufacturer’s protocol (Supplementary Table 7). Then, probe ligation, amplification, bright field detection, substrate development, nuclear staining and imaging were performed.

### Cell culture

Human PANC-1, MiaPaCa-2, HEK293T, AsPC-1, BxPC-3, hTERT-HPNE, CFPAC-1 cells were purchased from the Cell Bank of the Chinese Academy of Science. Cells were cultured in regular 1640 or DMEM medium plus 10% FBS and 1% penicillin/streptomycin. All cells were grown at 37 °C in the presence of 5% CO2. Testing for mycoplasma contamination is carried out every six months and STR profiles of the above cells are tested.

### Reagents

PugNAc, a potent O-GlcNAcase and hexosaminidase A and B inhibitor (ab144670) was purchased from Abcam. Thiamet-G (TMG) is an inhibitor of O-GlcNAcylase (S7213), which potently enhances protein O-GlcNAcylation and was purchased from Selleck. Cycloheximide, a eukaryotic protein synthesis inhibitor (S7418), was purchased from Selleck.

### Cycloheximide (CHX) assay for protein stability

For the CHX assay, 5 × 10^5^ pancreatic cancer cells were seeded in a 6 cm dish for 24 h. To measure the protein stability of SIRT7, 300 µg/ml CHX was added to block protein synthesis. Cells were collected at the indicated time points and then were processed to perform western blots.

### Recombinant plasmid experiments

SIRT7 was cloned into pcDNA 3.1(+) plasmid (Addgene). The primers were designed with Primer Premier 5.0 (see in Supplementary Table 3) and cDNA was synthesized according to the manufacture of PrimeScript RT Reagent Kit (TaKaRa). PCR products were separated by electrophoresis in 1.5% agarose gel and purified using the SanPrep Column DNA Gel Extraction Kit (Sangon Biotech). Then restriction endonuclease were added into the plasmid and the purified products. Then the recombinant plasmid was constructed using the Hieff CloneTM Plus One Step Cloning Kit (Yeasen). SIRT7 mutants (S134A, S136A, S377A) were prepared using a site-directed mutagenesis protocol (Hieff Mut™ Site-Directed Mutagenesis Kit, Yeasen). All the DNA sequences of the SIRT7 constructs were assessed by a sequencing service. The construction of the HA-OGT plasmid was similar to that of SIRT7 (primers are listed in supplementary Table 3). HA-Ub, HA-Ub K63R (mutation of lysine at 63 residue to arginine), HA-Ub K48R (mutation of lysine at 48 residue to arginine), HA-Ub K63 (mutation of lysine at 6, 11, 27, 29, 33, 48 residues to arginine) and HA-Ub K48 (mutation of lysine at 6, 11, 27, 29, 33, 63 residues to arginine) were synthesized by Mailgene Biosciences Co., Ltd.

### siRNA experiments

5 × 10^5^ pancreatic cancer cells were seeded into 6-well plates. After 24 h, the cells were treated with OGT siRNA/REGγ siRNA (GenePharma) or NC (negative control) using Lipofectamine 3000 reagent (Invitrogen) for 48 h. The siRNA sequences for OGT/REGγ are listed in Supplementary Table 4.

### Stable cell lines construction

We constructed short hairpin RNA (shRNA) expression vectors to knock down SIRT7 expression. The shRNA-targeted sequences for SIRT7 are listed in Supplementary Table 5. Cells that stably expressed the constructs were selected with puromycin (2 mg/ml) for one week. Then we transduced the treated cells with lentiviral vectors carrying the SIRT7-wildtype (WT)/S136A/S134A/S377A plasmid for overexpression. To avoid knockdown by shRNA, we made a synonymous mutation. The primers are listed in the Supplementary Table 6. Then the cells were selected with blasticidin S (10 µg/ml) for approximately one week. ShOGT was similar to shSIRT7. The targets and primers of shOGT are listed in Supplementary Table 5.

### Western blot analysis

Cells were lysed in RIPA lysis buffer (Sigma) containing phosphorylase and protease inhibitors (Sigma) and the proteins were separated through electrophoresis in 8–15% SDS-PAGE gels. Then the proteins were transferred from the gels to PVDF (polyvinylidenedifluoride) membranes (Bio-Rad) and incubated with the indicated antibodies (listed in Supplementary Table 7, used according to the product instructions). After incubation with a secondary antibody, the proteins were visualized by chemiluminescence. All original western blots are shown in Supplementary original data.

### Immunoprecipitation (IP) and Co-immunoprecipitation (co-IP)

Cells were lysed in Pierce IP buffer (Thermo Fisher) containing protease and phosphatase inhibitors (Sigma). After incubation with indicated antibodies, the lysates were mixed with protein A/G agarose (Thermo Fisher). For proteins with a tag, we used immunomagnetic beads coupled with anti-Flag/anti-HA antibody. After IP, protein A/G agarose or magnetic beads were washed three times with TBST (0.1% Tween-20, 150 mM NaCl, 10 mM Tris-HCl pH7.5) and eluted in SDS lysis buffer (100 mM NaCl, 1%SDS, 50 mM Tris-HCl pH 7.5) for western blots.

### Ubiquitination assay

Pancreatic cancer cells were transfected with HA-Ub WT/K48/K48R/K63/K63R and MYC-OGT plasmids for 48 h. Then the cells were performed co-IP experiments.

### Quantitative real-time PCR (qPCR)

Total RNA in cells was extracted using TRIzol RNA isolation system (Invitrogen) and converted to cDNA using PrimeScript RT Reagent Kit (TaKaRa). Then q-PCR was performed with a 7500 Fast™ System (Applied Biosystems) using the Sensi Mix SYBR Kit (Bio-Rad). The mRNA level was calculated using the 2-ΔΔCt method and normalized to *GAPDH*. The sequences of primers were designed by Primer 5 software (see Supplementary 1 Table 8).

### Chromatin immunoprecipitation (ChIP)

Cells were treated with plasmids for 48 h, then CHIP was performed using Pierce Agarose ChIP Kit (No.26156, Thermo Fisher Scientific). DNA template enrichment was analysed by qPCR using primers specific for each target genes promoter. All of the sequences of the primers are listed in Supplementary Table 9 [11].

### Mass spectrometry analysis of strip-complex and SIRT7 O-GlcNAcylation sites

The cut strips (the complex pulled down by anti-Flag immunomagnetic beads) containing recombinant O-GlcNAcylated SIRT7 proteins or SIRT7-interacting proteins were sent for LC-MS/MS (liquid chromatograph-mass spectroscopy/ mass spectroscopy) at Shanghai Applied Protein Technology [18].

### Cell proliferation assay

Cell proliferation assay was quantified every 24 h by using the MTT assay. Cells were plated at 1 × 10^3^ per well and incubated for the indicated hours. First, 10 µl MTT reagent was added. The mixture was incubated for 4 h until purple precipitate was visible. Then, 100 µl detergent reagent was added. The plate was shaken for 10 min and the absorbance was recorded at 562 nm. All experiments were repeated three times to determine the means and SDs.

### Colony formation assay

A total of 5 × 10^3^ stable cancer cells were transplanted into 6-well plates. Every 3–4 days the medium was replaced with fresh medium. On the 14th day, the colonies were clearly visible to the naked eye. The colonies were stained with crystal violet for 5 min.

### Tumour growth experiments in vivo

All animal experiments were approved by the ethics committee of Zhejiang University. For the xenograft assay, male athymic nude mice (6-8 weeks), obtained from the SLAC (Shanghai SLAC Laboratory Animal Co., Ltd.), were randomly divided into 3 or 4 groups. A total of 1 × 10^6^ cells in 100 µL PBS were injected into the right flanks of nude mice. The growth of tumours were observed every 3 days. At 4 weeks after the injection, all mice were sacrificed, and the tumours were harvested to detect tumour volume (V_tumour_ = 0.5 × L × W^2;^ L = length, W = width). Then the tumours were paraffin-embedded and sectioned for HE (hematoxylin-eosin) and Ki67 staining. The investigators were blinded to the group allocation during the experiment and when assessing the outcome.

### Statistical analysis

All experiments were performed in triplicates. All data are shown as the mean ± SD. Data were analysed using two-tailed *t*-tests, Chi-square (x2) tests or one-way analysis of variance (ANOVA). Kaplan–Meier survival analyses were used to compare survival times among PDAC patients based on SIRT7 expression; the log-rank test was used to generate P values. Cox proportional hazards regression analyses were used to assess the effect of clinical variables on patient survival. Univariate and multivariate analyses were used to assess the influence of clinical variables on survival. The p values and hazard ratios are indicated. *P* values < 0.05 were considered statistically significant. (*P < 0.05; **P < 0.01; ***P < 0.001; ****P < 0.0001). All histograms and curves were constructed with GraphPad Prism 8.0.2 software (GraphPad Software, La Jolla, CA, USA) and SPSS V.21.0 software (SPSS, Inc., Chicago, IL, USA).

## Results

### SIRT7 overexpression is associated with poor prognosis in pancreatic cancer and enhances pancreatic cancer cell growth in vitro and in vivo

**Fig. 1:**
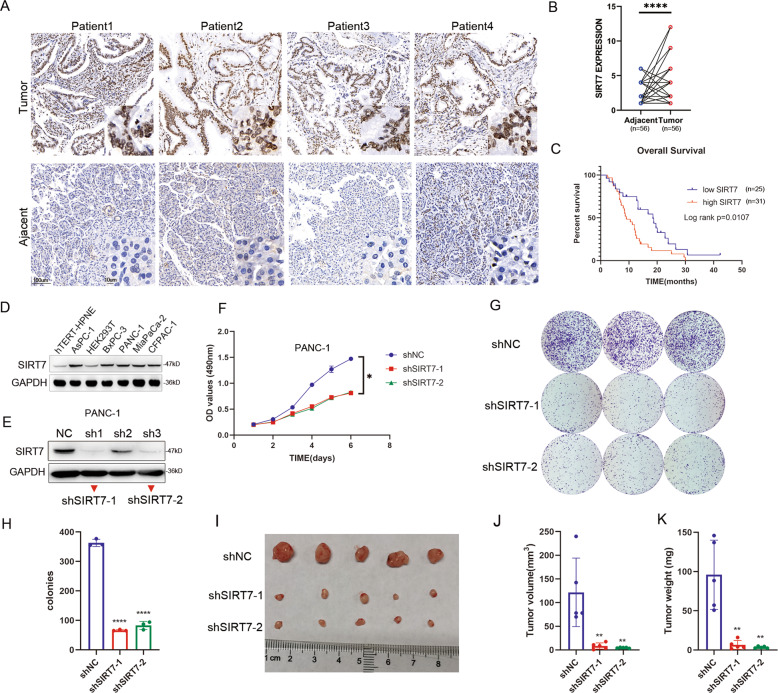
SIRT7 overexpression predicts a poor prognosis in pancreatic cancer and is crucial for pancreatic cancer cell proliferation and progression in vitro and in vivo. **A,** **B** Representative IHC staining and statistical analysis of SIRT7 from 56 pairs of pancreatic tumour and normal adjacent tissues. Scale bar as shown. **C** Kaplan–Meier overall survival curves for all 56 patients with pancreatic cancer stratified by high and low SIRT7 expression. **D** Western blotting of SIRT7 expression in HEK293T cells, normal pancreatic ductal epithelial cells (hTERT-HPNE) and pancreatic cancer cell lines. One representative experiment of *n* = 3 independent experiments is shown. **E** Western blotting of SIRT7 expression in PANC-1 cells transfected with shRNAs. One representative experiment of *n* = 3 independent experiments is shown. **F** MTT assays of PANC-1 cells transfected with shNC, shSIRT7-1 or shSIRT7-2 plasmids. One representative experiment of *n* = 3 independent experiments is shown. **G,** **H** Colony formation assays and statistical analysis of the above groups of PANC-1 cells. One representative experiment of *n* = 3 independent experiments is shown. **I**–**K** The Effects of SIRT7 on tumour xenografts in nude mice. PANC-1 cells with stable SIRT7 silencing by shRNA (shNC, shSIRT7-1 or shSIRT7-2) were injected subcutaneously into the axillae of nude mice (*n* = 5 for each group). Mice were sacrificed after 4 weeks, and their tumour masses were excised and weighed. V_tumour_ = 0.5 × L × W^2^. One representative experiment of *n* = 3 independent experiments is shown. (The data are shown as the means ± SD. Statistical significance was determined by two-tailed *t*-tests (*P < 0.05; **P < 0.01; ***P < 0.001; ****P < 0.0001; NS, no significance)).

To achieve a comprehensive understanding of SIRT7 expression, we evaluated the SIRT7 expression levels in a high-density tissue microarray by IHC. Representative images indicated that expression of SIRT7 in tumour tissues was higher than that in matched peritumour tissues (Fig. 1A and Supplementary Table 1). The expression levels of SIRT7 were relatively higher in the PDAC tissues than in the adjacent normal pancreatic tissues (*P* = 0.001). There was no significant difference in SIRT7 expression between different groups stratified by age (*P* = 0.611), sex (*P* = 0.884) or TNM stage (*P* = 0.555). However, a higher expression level of SIRT7 was associated with a worse differentiation (*P* = 0.015) and a higher lymph node metastasis (*P* = 0.005) (Fig. 1B and Table 1). The Kaplan–Meier survival curves showed that overall survival (OS) in the patients with higher SIRT7 expression levels was significantly worse than in those with lower SIRT7 expression (*P* = 0.0107) (Fig. 1C). In addition, multivariate analysis indicated that classification of TNM stage (*P* = 0.007, HR = 0.257(0.095–0.692)) and SIRT7 expression (*P* = 0.032, HR = 0.507(0.272–0.944)) were independent predictors of prognosis (Supplementary Table 2). In summary, SIRT7 was generally upregulated in PDAC tissues and was associated with a poor OS.

**Table 1:** Correlation analysis of the clinicopathological parameters with the level of SIRT7 protein expression in 56 patients with PDAC.

Variables	*N*	SIRT7		
		High	Low	P
Gender				0.884
Male	33	18	15	
Female	23	13	10	
Age				0.611
<60	27	14	13	
>=60	29	17	12	
Differentiation				**0.015**
Well	43	20	23	
Poorly	13	11	2	
Lymph node metastasis				**0.005**
Yes	25	19	6	
No	31	12	19	
Classification of TNM				0.555
I–II	50	27	23	
III–IV	6	4	2	

(*P* value: Chi-squared test; *P* values < 0.05 were considered statistically significant (shown in bold)).

Next, we detected the expression levels of SIRT7 in pancreatic cell lines and found that the expression of SIRT7 was higher in cancer cells (AsPC-1, BxPC-3, PANC-1, MiaPaCa-2, CFPAC-1) than in normal ductal epithelial cell (hTERT-HPNE) (Fig. 1D). To determine the internal connection between SIRT7 and pancreatic cancer progression, we generated endogenous SIRT7 knockdown pancreatic cancer: PANC-1 and MiaPaCa-2 cells by shRNAs. The sequences of sh1 and sh3 for SIRT7 knockdown were effective and were named shSIRT7-1 and shSIRT7-2, respectively (Fig. 1E and Supplementary Fig. S1A). MTT assays revealed that SIRT7 knockdown substantially inhibited cell proliferation in PANC-1 and MiaPaCa-2 cell lines (Fig. 1F and Supplementary Fig. S1B). In the colony formation assay, the mean colony number was also significantly decreased in these two cells with SIRT7 depletion compared to that of the control group (Figs. 1G, 1H and Supplementary Fig. S1C, S1D). To validate the effect of SIRT7 on the growth of pancreatic cancer cells in vivo, negative control (NC) and SIRT7-depleted PANC-1 and MiaPaCa-2 cells were injected into nude mice, and the tumour volume and weight were measured after 4 weeks. Tumour weight and volume in the animals injected with NC cells increased significantly compared with those in the mice administered SIRT7-depleted cells (Fig. 1I–K and Supplementary Fig. S1E–G). SIRT7 inactivation in pancreatic cancer cells impaired their progression in vitro and in vivo.

### OGT binds to SIRT7 and promotes SIRT7 O-GlcNAcylation

**Fig. 2:**
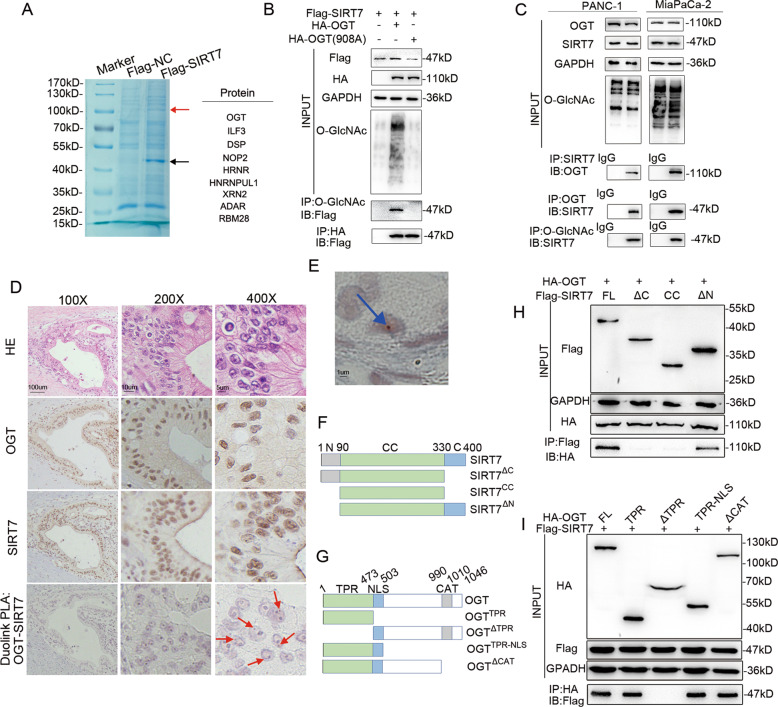
SIRT7 can be O-GlcNAcylated by OGT. **A** Affinity purification of SIRT7 interacting proteins from HEK293T cell stably expressing Flag-SIRT7. SDS-PAGE and Coomassie Brilliant Blue staining shows proteins co-eluted with SIRT7. The black arrow points to SIRT7, and the red arrow points to the differential band of interest. The differential band was cut for analysis by LC-MS/MS, and a series of peptides that contained OGT were detected at 110kD. **B** Co-IP assays were performed in HEK293T cells transfected with the HA-OGT, HA-OGT-908A or Flag-SIRT7 plasmids. One representative experiment of *n* = 3 independent experiments is shown. **C** Co-IP assays of OGT and SIRT7 were examined in PANC-1 and MiaPaCa-2 cells. One representative experiment of *n* = 3 independent experiments is shown. **D** Representative IHC staining of OGT, SIRT7 and Duolink® PLA brightfield signals (red arrows) in one PDAC patient (consecutive sections). Scale bar as shown. One representative experiment of *n* = 3 independent experiments is shown. **E** Schematic drawing of full-length SIRT7 and truncations. **F** Schematic drawing of full-length OGT and truncations. **G** Co-IP assays were performed in HEK293T cells transfected with the HA-OGT and Flag-SIRT7 truncations. One representative experiment of *n* = 3 independent experiments is shown. **H**, **I** Co-IP assays were performed in HEK293T cells transfected with the Flag-SIRT7 and HA-OGT truncations. One representative experiment of *n* = 3 independent experiments is shown.

We previously showed that higher expression of SIRT7 correlated with a worse prognosis of pancreatic cancer and promoted the progression of pancreatic cancer cells. To identify novel proteins that may regulate SIRT7 functions, we transfected HEK293T cells with the Flag-SIRT7 plasmid and then purified the SIRT7-interacting proteins for LC-MS/MS analysis. A series of peptides were detected, and one of the most abundant bands matched the peptide of OGT (Fig. 2A). Exogenous co-IP assays validated that SIRT7 could interact with OGT and then be O-GlcNAcylated in HEK293T cells. We sought to determine whether the interaction between OGT and SIRT7 depended on its enzymatic activity by generating a mutation with lysine 908 replaced by alanine (K908A) in human OGT [21]. As previously described, the K908A mutation abolished the enzymatic function of OGT without damaging its protein abundance. In our work, we found that the OGT K908A mutant could still interact with SIRT7. However, this mutant failed to induce SIRT7 O-GlcNAcylation, indicating that O-GlcNAcylated SIRT7 is dependent on the enzymatic activity of OGT (Fig. 2B). We next investigated the endogenous protein interaction by further exploring endogenous SIRT7 and OGT in pancreatic cancer cell lines. Consistently, the results indicated that endogenous SIRT7 pulled down by the SIRT7 antibody could be recognized by O-GlcNAc antibody when SIRT7 and OGT were expressed at endogenous levels and SIRT7 and OGT interacted mutually in PANC-1 and MiaPaCa-2 cells (Fig. 2C). We performed IHC staining of SIRT7 and OGT on PDAC patient specimens, and examined the interaction of SIRT7 and OGT in specimens with Duolink PLA analysis. The results indicated that OGT and SIRT7 interacted in the nucleus in pancreatic tumours (Figs. 2D, E).

To determine the interacting domain between SIRT7 and OGT, we made 3 truncations of the SIRT7 protein: the catalytic core region (CC: 91-330), the N-terminus with CC region (ΔC: 1-330) and the C-terminus with CC region (ΔN: 91-400) (Fig. 2F). We observed that the full length (FL) protein and C-terminus with CC region (ΔN) could bind to OGT. However, the catalytic core region (CC: 91-330) and N-terminus with CC region (ΔC: 1-330) did not bind with OGT (Fig. 2H). These results suggested that residues 331-400 of SIRT7 might be necessary for its interaction with OGT. OGT is composed of several tetratricopeptide repeats (TPR, 1-473), a signal for nuclear localization (NLS, 487-503) and a catalytic region (CAT, 991-1010). Four OGT truncations (OGT^TPR^, OGT^ΔTPR^, OGT^TPR-NLS^ and OGT^ΔCAT^) were constructed (Fig. 2G). In co-IP assays, only OGT^ΔTPR^ showed no Flag-SIRT7 precipitation, indicating that deleting the tetratricopeptide repeats severely impaired the OGT-SIRT7 interaction (Fig. 2I). Taken together, the results suggested that the C-terminal domain (331-400) of SIRT7 and the TPR domain of OGT might be potential interaction domains. Overall, OGT is an interacting protein of SIRT7 and can induce the O-GlcNAcylation of SIRT7.

### O-GlcNAcylation stabilizes SIRT7 protein

**Fig. 3:**
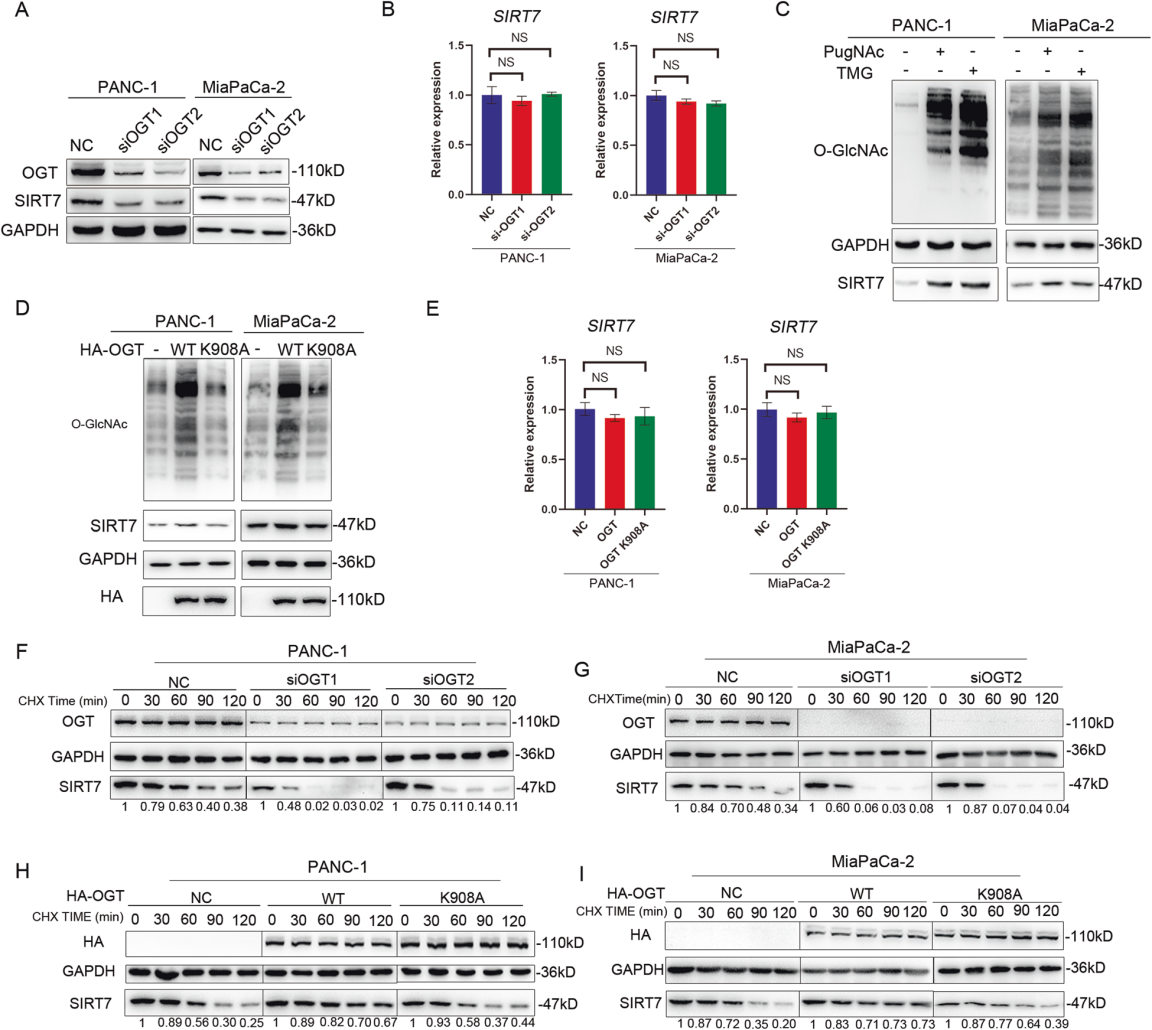
O-GlcNAcylation stabilizes SIRT7 protein. **A** Knockdown of OGT regulated the protein level of SIRT7 in PANC-1 and MiaPaCa-2 cells. One representative experiment of *n* = 3 independent experiments is shown. **B** Knockdown of OGT regulated the mRNA level of SIRT7 in PANC-1 and MiaPaCa-2 cells. One representative experiment of *n* = 3 independent experiments is shown. **C** OGA inhibitors PugNAc and thiamet-G (TMG) treatment regulated the protein level of SIRT7. PANC-1 and MiaPaCa-2 cells were treated with PugNAc (1 µM, 4 h) and thiamet-G (TMG) (10 µM, 4 h), and cell lysates were immunoblotted with indicated antibodies. One representative experiment of *n* = 3 independent experiments is shown. **D** Empty vector or expression vector for OGT WT or K908A mutant was transfected to PANC-1 and MiaPaCa-2 cells, and cell lysates were immunoblotted with indicated antibodies. One representative experiment of *n* = 3 independent experiments is shown. **E** Empty vector or expression vector for OGT WT or K908A mutant was transfected to PANC-1 and MiaPaCa-2 cells, and the mRNA level of SIRT7 was detected. One representative experiment of *n* = 3 independent experiments is shown. **F**, **G** The NC group and siOGT groups of PANC-1 and MiaPaCa-2 cells were treated with CHX (300 µg/ml), and the protein level of SIRT7 was recorded at 0, 30, 60, 90 and 120 min. Relative SIRT7 band intensities were quantified through densitometry and are presented. One representative experiment of *n* = 3 independent experiments is shown. **H**, **I** PANC-1 and MiaPaCa-2 cells transfected with NC, HA-OGT or HA-OGT-908A were treated with CHX (300 µg/ml), and the protein level of SIRT7 was recorded at 0, 30, 60, 90 and 120 min. Relative SIRT7 band intensities were quantified through densitometry and are presented. One representative experiment of *n* = 3 independent experiments is shown. The data are shown as the means ± SD. *P* values were calculated by two-tailed *t*-tests (*P < 0.05; **P < 0.01; ***P < 0.001; ****P < 0.0001; NS, no significance).

As O-GlcNAcylation usually affects the protein stability, we repressed OGT expression in PANC-1 and MiaPaCa-2 cells and tested the protein extracts by western blotting to determine the expression level of SIRT7. OGT deprivation by siRNA reduced the expression of SIRT7 at the post-translational level (Fig. 3A) but not at the post-transcriptional level (Fig. 3B), which suggested that OGT might regulate SIRT7 expression at the post-translational level. The protein level of SIRT7 was also elevated by PugNAc or TMG treatment in HEK293T (Supplementary Fig. S2A) or PANC-1 or MiaPaCa-2 cells (Fig. 3C). Consistent with the above results, overexpression of OGT, but not the K908A mutant, markedly promoted SIRT7 expression (Fig. 3D). Additionally, overexpression of OGT did not alter SIRT7 expression at the transcript level (Fig. 3E). Moreover, to test whether the change in O-GlcNAc could alter the stability of SIRT7, we treated PANC-1 NC and siOGT groups with CHX to inhibit protein synthesis. As shown in Fig. 3F, 60 min after CHX treatment, the protein level of SIRT7 dropped to only approximately 63% in the NC group, whereas the protein level of SIRT7 dropped to nearly 0% in the siOGT1 group and 11% in the siOGT2 group. MiaPaCa-2 showed similar results (Fig. 3G). We also overexpressed OGT in PANC-1 and MiaPaCa-2 cells to explore endogenous SIRT7 degradation. In PANC-1 NC and K908A groups, SIRT7 dropped to 30% and 37% 90 min after treatment with CHX, while it was 70% in the WT group (Fig. 3H). MiaPaCa-2 showed similar results (Fig. 3I).

### OGT promotes the stability of SIRT7 via inhibiting the interaction between SIRT7 and REGγ

**Fig. 4:**
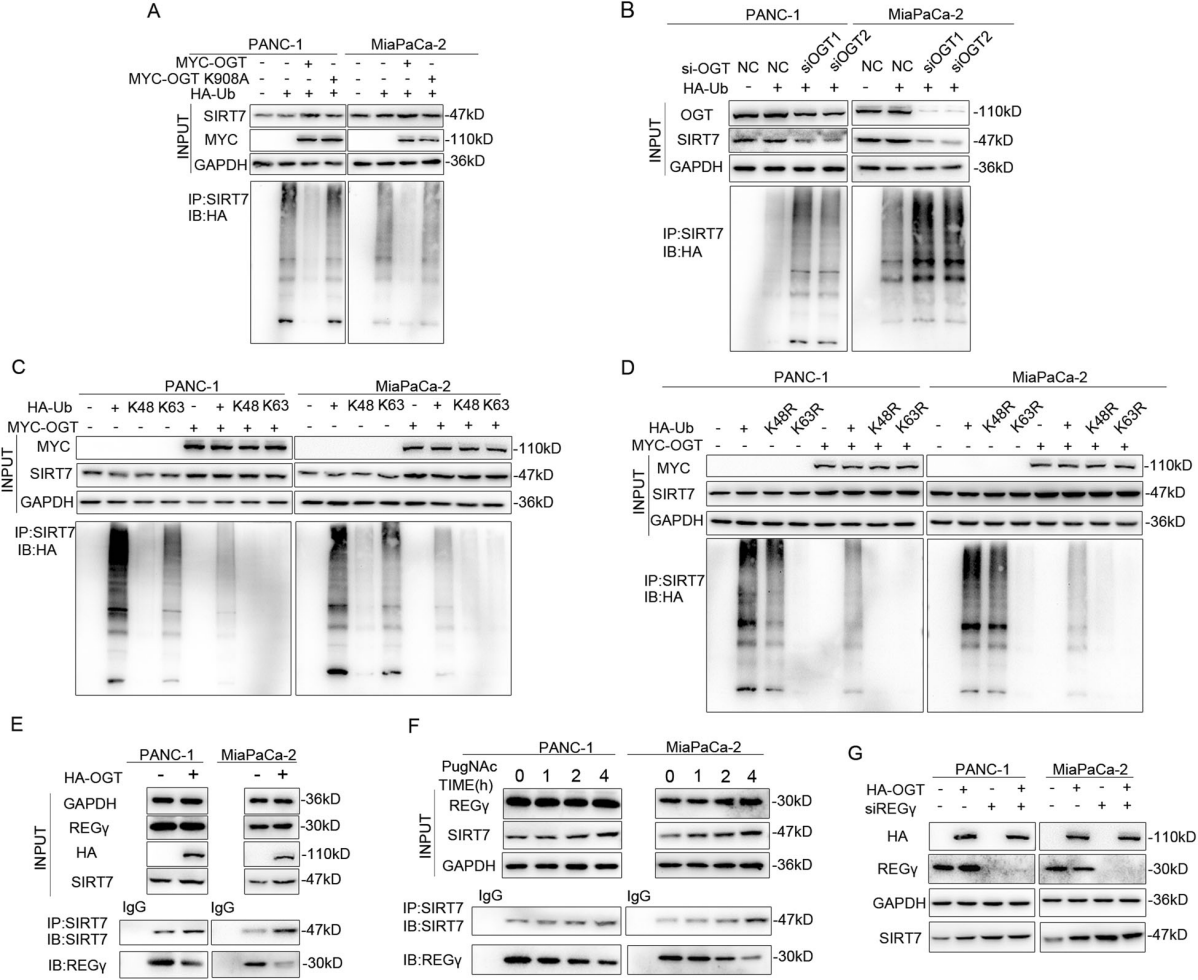
OGT promotes the stability of SIRT7 via preventing its interaction with REGγ. **A** PANC-1 and MiaPaCa-2 cells transfected with MYC-OGT, MYC-OGT-K908A and HA-Ub were used for immunoprecipitation with Protein A/G agarose incubated with anti-SIRT7 antibody, followed by HA western blotting. One representative experiment of *n* = 3 independent experiments is shown. **B** PANC-1 and MiaPaCa-2 cells treated with NC/siOGT and HA-Ub were immunoprecipitated with Protein A/G agarose incubated with anti-SIRT7 antibody, followed by HA western blotting. One representative experiment of *n* = 3 independent experiments is shown. **C** PANC-1 and MiaPaCa-2c ells transfected with MYC-OGT, HA-Ub, HA-Ub K48, and HA-Ub K63 were immunoprecipitated with Protein A/G agarose incubated with anti-SIRT7 antibody, followed by HA western blotting. One representative experiment of *n* = 3 independent experiments is shown. **D** PANC-1 and MiaPaCa-2 cells transfected with MYC-OGT, HA-Ub, HA-Ub K48R, and HA-Ub K63R were immunoprecipitated with immunoprecipitated with Protein A/G agarose incubated with anti-SIRT7 antibody, followed by HA western blotting. One representative experiment of *n* = 3 independent experiments is shown. **E** The relationship between SIRT7/REGγ interaction and OGT was detected. PANC-1 and MiaPaCa-2 cells transfected with HA-OGT were immunoprecipitated with Protein A/G agarose incubated with anti-SIRT7 antibody, followed by western blotting for SIRT7 and REGγ. One representative experiment of *n* = 3 independent experiments is shown. **F** The relationship between SIRT7/REGγ interaction and O-GlcNAcylation was detected. PANC-1 and MiaPaCa-2 cells treated with PugNAc (1 µM) were immunoprecipitated with Protein A/G agarose and incubated with anti-SIRT7 antibody, followed by western blotting for SIRT7 and REGγ. One representative experiment of *n* = 3 independent experiments is shown. **G** The relationship between REGγ and OGT in SIRT7 expression was detected. PANC-1 and MiaPaCa-2 cells were transfected with OGT and siREGγ as indicated. One representative experiment of *n* = 3 independent experiments is shown.

From the results above, we found that inhibition of OGT accelerated SIRT7 degradation. Protein degradation mediated by the ubiquitin proteasome pathway is a common mechanism by which cells regulate the level and function of intracellular proteins. Therefore, we tested the association between SIRT7 O-GlcNAcylation and ubiquitination. We found that SIRT7 was ubiquitinated and that ubiquitination was decreased by overexpression of OGT but not OGT-K908A in PANC-1 and MiaPaCa-2 cells, indicating that the inhibition was dependent on enzymatic activity (Fig. 4A). The ubiquitination of SIRT7 was enhanced after treatment with siOGT (Fig. 4B). Using mutant ubiquitin constructs, we showed that SIRT7 was polyubiquitinated in a K63-dependent manner (Fig. 4C). The ubiquitination mode of SIRT7 was further validated by using Ub-K63R and Ub-K48R plasmids. Similarly, our results showed that SIRT7 was polyubiquitinated by Ub-K48R but not Ub-K63R and that OGT dramatically decreased K48R ubiquitination (Fig. 4D). The most common consequence of K48-linked polyubiquitination is proteasome-mediated degradation, while K63-linked polyubiquitination is related to other cellular processes. Taken together, our data suggested that the stability of SIRT7 might not be affected by ubiquitination. Recently, REGγ proteasome, as a core molecule of a new ubiquitin-independent pathway, was shown to mediate SIRT7 degradation by binding to it. Then, we explored whether OGT promoted SIRT7 stability by regulating REGγ proteasome pathway. We overexpressed OGT in PANC-1 and MiaPaCa-2 cells and found that SIRT7 pulled down less REGγ (Fig. 4E). We also treated PANC-1 cells with PugNAc and found that more O-GlcNAcylation of SIRT7 resulted in less REGγ interaction (Fig. 4F). Overexpression of OGT increased SIRT7 expression in PANC-1 and MiaPaCa-2 cells. However, knockdown of REGγ abolished this effect, which indicated that OGT could increase the stability of SIRT7 in a REGγ-dependent manner (Fig. 4G). Collectively, O-GlcNAcylated SIRT7 might increase the stability of SIRT7 by disrupting the interaction between SIRT7 and REGγ.

### SIRT7 O-GlcNAcylation represses target genes via hypoacetylation of H3K18

**Fig. 5:**
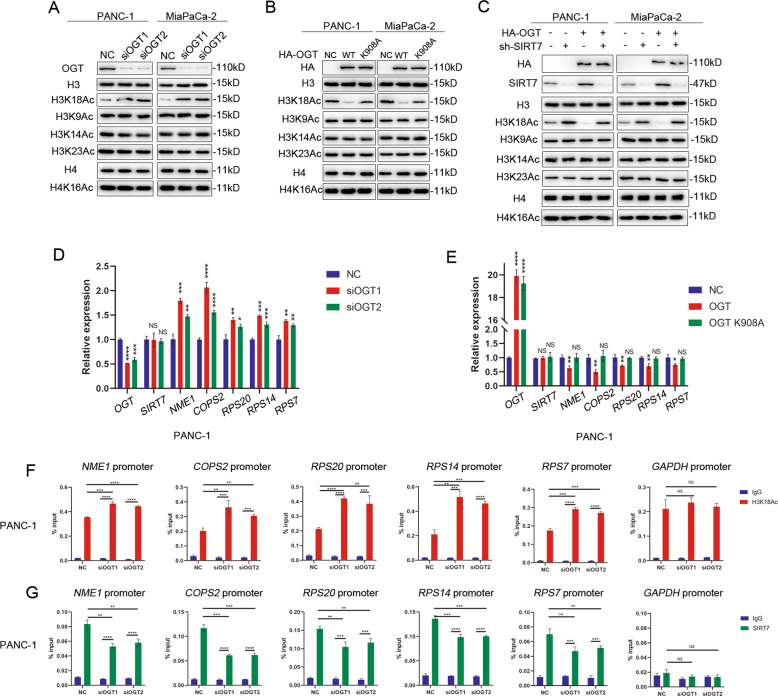
Loss of SIRT7 O-GlcNAcylation inhibits expression of target genes via hyperacetylation of H3K18. **A** Western analysis of multiple histone acetylation sites of H3, H4 in PANC-1 and MiaPaCa-2 cells treated with SIRT7 knockdown. One representative experiment of *n* = 3 independent experiments is shown. **B** Western analysis of multiple histone acetylation sites of H3, H4 in PANC-1 and MiaPaCa-2cells transfected with NC, HA-OGT or HA-OGT-908A. One representative experiment of *n* = 3 independent experiments is shown. **C** Western analysis of multiple histone acetylation sites of H3, H4 in PANC-1 and MiaPaCa-2cells transfected with HA-OGT or/and siSIRT7. One representative experiment of *n* = 3 independent experiments is shown. **D** The expression of SIRT7 target genes in the PANC-1 cells treated with SIRT7 knockdown, as determined by qPCR. One representative experiment of *n* = 3 independent experiments is shown. **E** The expression of SIRT7 target genes in the PANC-1 cells transfected with NC, HA-OGT or HA-OGT-908A, as determined by qPCR. One representative experiment of three independent experiments is shown. **F** ChIP-qPCR results showing H3K18Ac occupancy at the promoters of SIRT7 target genes in control or OGT knockdown PANC-1 cells. One representative experiment of *n* = 3 independent experiments is shown. **G** ChIP-qPCR results showing SIRT7 occupancy at the promoters of SIRT7 target genes in control or OGT knockdown PANC-1 cells. One representative experiment of *n* = 3 independent experiments is shown. The data are shown as the means ± SD. *P* values were calculated by two-tailed *t* tests (*P < 0.05; **P < 0.01; ***P < 0.001; ****P < 0.0001; NS, no significance).

Next, we sought to determine whether SIRT7 O-GlcNAcylation could affect its deacetylation on H3K18. We used siRNA to knock down OGT expression in PANC-1and MiaPaCa-2 cells and the expression levels of H3K18Ac were increased, while the H3, H4 and other acetylation sites were unchanged (Fig. 5A). In addition, hyper-O-GlcNAcylation by OGT overexpression but not K908A decreased the acetylation level of H3K18 (Fig. 5B), while other acetylation sites did not change (Fig. 5B). In PANC-1 cells, H3K18Ac was reduced by overexpression of OGT (Fig. 5C), and this reduction could be partially rescued by SIRT7 knockdown (Fig. 5C), which indicated that OGT promoted the deacetylation of H3K18 via SIRT7. Additionally, the silencing of OGT led to the upregulation of the expression of six tumour suppressor genes (*NME1, RPS20, RPS7, RPS14* and *COPS2*), which were directly targeted by SIRT7 (Fig. 5D) [11], and overexpression of OGT led to a reduction of these suppressor genes at the transcriptional level dependent on its catalytic activity in PANC-1 and MiaPaCa-2 cells (Fig. 5E and Supplementary Fig. S3C, S3D). Furthermore, deprivation of SIRT7 O-GlcNAcylation led to hyperacetylation of H3K18 and lower recruitment of SIRT7 at the promoters of the *NME1, RPS20, RPS7, RPS14* and *COPS2* genes but not the NC promoter (*GAPDH*) according to the ChIP assay (Fig. 5F, G and Supplementary Fig. S3E, S3F). Accordingly, enhanced SIRT7 O-GlcNAcylation caused hypoacetylation of H3K18 and increased recruitment of SIRT7 to the promoters of the genes (Supplementary Fig. S3A, S3B, S3G, S3H).

### SIRT7 is O-GlcNAcylated at Serine 136

**Fig. 6:**
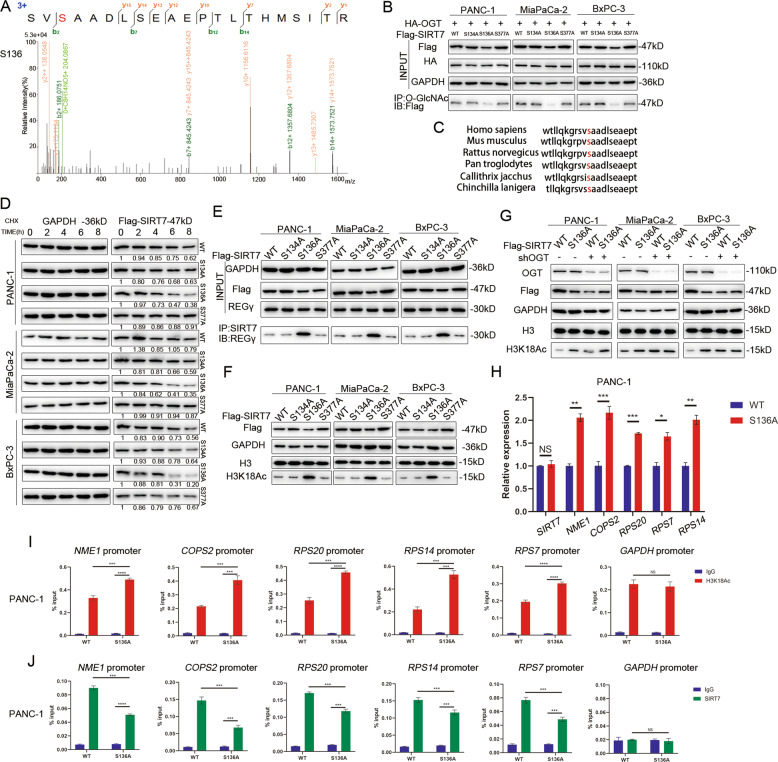
SIRT7 is O-GlcNAcylated at Ser136. **A** Detection of the O-GlcNAcylation site(s) on SIRT7. SIRT7 was purified from HEK293T cells and analysed by LC-MS/MS analysis to identify the potential O-GlcNAcylation sites. The serine 136 O-GlcNAcylation site of SIRT7 was shown. **B** The O-GlcNAcylation levels of SIRT7 in PANC-1, MiaPaCa-2 and Bxpc-3 cells were detected. Endogenous SIRT7 was knocked down and then rescued by Flag-tagged SIRT7 WT, S134A, S136A, and S377A plasmids in these three cell lines. One representative experiment of *n* = 3 independent experiments is shown. **C** Serine 136 residue of SIRT7 is conserved in multiple species. **D** Western analysis of indicated cells treated with CHX (300 µg/ml) for 0, 1, 2, 4, 6, or 8 h. One representative experiment of *n* = 3 independent experiments is shown. **E** Co-IP assays of SIRT7 and REGγ were examined in indicated cells. One representative experiment of *n* = 3 independent experiments is shown. **F** The expression level of H3K18Ac in the indicated cells. One representative experiment of *n* = 3 independent experiments is shown. **G** The expression level of H3K18Ac in the indicated cells with or without OGT knockdown. One representative experiment of *n* = 3 independent experiments is shown. **H** Expression of SIRT7 target genes in SIRT7 WT and SIRT7 S136A PANC-1 cells, as determined by qPCR. One representative experiment of *n* = 3 independent experiments is shown. **I** ChIP-qPCR results showing H3K18Ac occupancy at the promoters of SIRT7 target genes in SIRT7 WT and SIRT7 S136A PANC-1 cells. One representative experiment of *n* = 3 independent experiments is shown. **J** ChIP-qPCR results showing SIRT7 occupancy at the promoters of SIRT7 target genes in SIRT7 WT and SIRT7 S136A PANC-1 cells. One representative experiment of *n* = 3 independent experiments is shown. The data are shown as the means ± SD. *P* values were calculated by two-tailed *t*-tests (*P < 0.05; **P < 0.01; ***P < 0.001; ****P < 0.0001; NS, no significance).

To identify the O-GlcNAcylation site(s) on SIRT7, we performed protein purification and mass spectrometry analysis, and three potential O-GlcNAcylation sites (serines 134 (S134), 136 (S136) and 377 (S377)) of SIRT7 were revealed by LC-MS/MS (Fig. 6A and Supplementary Fig. S4A, S4B). However, the functional roles of these sites have not been determined. We treated PANC-1, MiaPaCa-2 and BxPC-3 cells with shSIRT7 (Fig. 1E, Supplementary Fig. S1A, S1H) and rescued them with a series of mutant constructs of SIRT7-mutating potential O-GlcNAcylation sites to alanine (S134A, S136A, S377A). The co-IP results in the above cells revealed that the mutation at S136, not S134 or S377, caused the main loss of the O-GlcNAc signal, indicating that SIRT7 was mainly O-GlcNAcylated on S136 (Fig. 6B). We also observed that S136 of SIRT7 was conserved in multiple species (Fig. 6C). We then explored whether the loss of SIRT7 O-GlcNAcylation at S136 affected protein stability. We found that SIRT7-S136A degraded fastest after CHX treatment. In contrast, SIRT7 WT and the other two mutants decreased slightly, which suggested that SIRT7 O-GlcNAcylation at S136 might promote the stability of SIRT7 (Fig. 6D). Then we found that S136A strongly interacted with REGγ while less REGγ was detected in the other three groups, indicating that loss of SIRT7 O-GlcNAcylation at S136 could increase the interaction between REGγ and SIRT7, therefore leading to the degradation of SIRT7 (Fig. 6E). Furthermore, deprivation of O-GlcNAcylation at S136 also increased the acetylation of H3K18 in the indicated cells (Fig. 6F), and knockdown of OGT abrogated the deacetylation ability of Flag-SIRT7-WT (Fig. 6G). As a result, in the S136A group, the direct target genes of SIRT7, including *NME1*, *RPS20*, *RPS7*, *RPS14*, and *COPS2* were upregulated at the mRNA level, as shown by qPCR (Fig. 6H and Supplementary Fig. S4C, S4F). S136A contributed to hyperacetylation of H3K18 and lower recruitment of SIRT7 at the promoters of the *NME1, RPS20, RPS7, RPS14* and *COPS2* genes compared to the WT but not the NC promoter (*GAPDH*), as shown by ChIP assays (Fig. 6I, J and Supplementary Fig. S4D, S4E, S4G, S4H). Thus, SIRT7 O-GlcNAcylation at S136 increases the SIRT7 recruitment at the promoters of tumour suppressor genes to inhibit their expression, leading to tumour progression.

### SIRT7 O-GlcNAcylation on S136 promotes tumour progression in pancreatic cancer cells

**Fig. 7:**
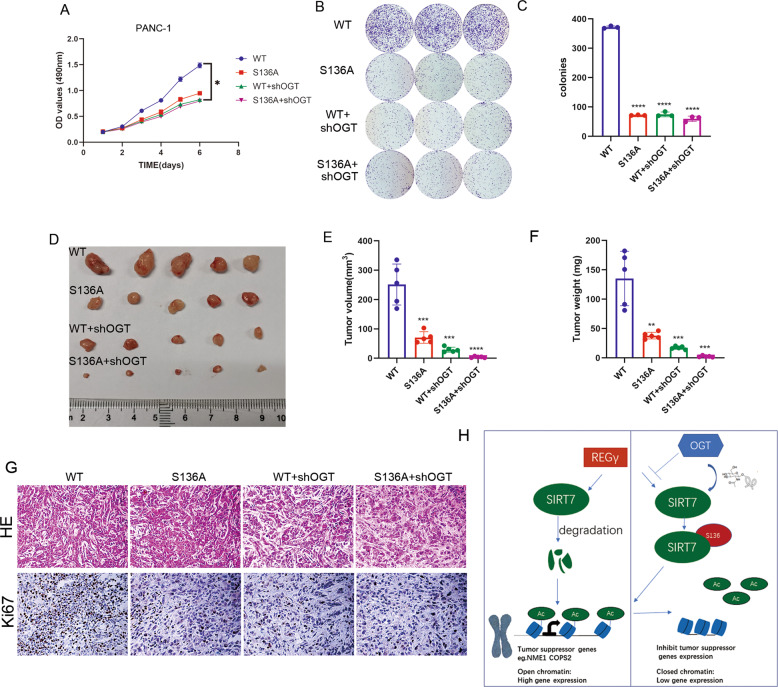
SIRT7 O-GlcNAcylation on S136 promotes tumour progression in pancreatic cancer cells. **A** PANC-1 cells were transfected with shSIRT7 and rescued by SIRT7-WT and S136A. Then, the cells were treated with or without shOGT (WT, S136A, WT+shOGT, S136A+shOGT). MTT assays were performed in the indicated cells. One representative experiment of *n* = 3 independent experiments is shown. **B**, **C** Colony formation assays and statistical analysis of the indicated cells. One representative experiment of *n* = 3 independent experiments is shown. **D**–**F** The effects of SIRT7 S136A mutation on tumour xenografts in nude mice. Four groups of PANC-1 cells were injected subcutaneously into the axillae of nude mice (*n* = 5 for each group). Mice were sacrificed after 4 weeks, and their tumour masses were excised and weighed. V_tumour_ = 0.5 × L × W^2^. One representative experiment of *n* = 3 independent experiments is shown. **G** Xenograft samples were subjected to Ki67 and HE staining. One representative experiment of *n* = 3 independent experiments is shown. **H** A schematic model of how SIRT7 O-GlcNAcylation exerts its function in pancreatic cancer cells. The data were shown as the means ± SD. *P* values were calculated by two-tailed *t*-tests (*P < 0.05; **P < 0.01; ***P < 0.001; ****P < 0.0001; NS, no significance).

To substantiate the physiological function of SIRT7 O-GlcNAcylation at S136, we next examined the role of OGT-dependent SIRT7 activation in oncogenic transformation. Compared with the group expressing SIRT7 WT, the other three groups (S136A, WT+shOGT, and S136A+shOGT) that lost SIRT7 or SIRT7 O-GlcNAcylation displayed a lower growth rate in PANC-1 (Fig. 7A) and MiaPaCa-2 cells (Supplementary Fig. S5A). In the colony formation assay, there were much more colonies in the SIRT7 WT group than in the other three groups (Fig. 7B, C and Supplementary Fig. S5B,S5C). We also injected these cell lines into nude mice to observe subcutaneous tumour formation. Compared with the mice injected with SIRT7 WT, the mice injected with S136A, WT+shOGT, or S136A+shOGT exhibited decreased tumour volume and mass (Fig. 7D–F and Supplementary Fig. S5D–F). In line with the impaired tumour growth, Ki-67, the classic marker of cell proliferation, was significantly decreased in those tumour tissues (Fig. 7G). These data indicated that O-GlcNAcylation of SIRT7 at S136 showed a growth advantage for tumour cells in vitro and in vivo.

## Discussion

In this study, we first provide evidence that the expression of SIRT7 is upregulated in pancreatic cancer tissues and associated with a poor prognosis. Next, coimmunoprecipitation combined with subsequent MS was performed to identify O-GlcNAcylation as a potential novel PTM of SIRT7 by interaction with OGT in pancreatic cancer cell lines. Further results demonstrate that the conserved role of O-GlcNAcylation can maintain SIRT7 protein stability by suppressing the interaction between SIRT7 and REGγ and enhancing the deacetylation of histones. In addition, OGT can modify SIRT7 with O-GlcNAcylation at several residues, and O-GlcNAcylation at the serine 136 residue plays a key role (Fig. 7H).

In recent years, the relationship between sirtuin family members and pancreatic cancer has been widely studied due to their controversial functions in cancers. For example, SIRT1 can promote the pancreatic cancer progression by regulating cell proliferation, chemosensitivity, and acinar-to-ductal metaplasia [22–24]. SIRT6 enhances cytokine production and migration in pancreatic cancer cells by regulating Ca^2+^ responses [25]. A previous study identifies that low levels of nuclear SIRT7 expression are associated with an aggressive tumour phenotype and poorer outcome [26]. In contrast, our findings confirm that high levels of nuclear SIRT7 expression predict a poor prognosis, and this contradictory result may be caused by racial or pathological differences.

To date, the protein function of mature SIRT7 can be affected by multiple post-translational modifications. AMPK-directed SIRT7 phosphorylation at T153 is induced under energy starvation. Subsequently, SIRT7 is subcellular redistributed and degraded in a REGγ-dependent manner, thereby further reducing rDNA transcription to save energy to overcome cell death [27]. Moreover, protein arginine methyltransferase 6 (PRMT6) can directly interact with and then methylate SIRT7 at R388 both in vitro and in vivo. R388 methylation of SIRT7 triggers suppression of H3K18 deacetylase activity without modulating its subcellular localization [28]. Despite the expanding data on its post-translational modifications, to our knowledge, SIRT7 has never been linked to O-GlcNAc modification.

The effect of post-translational modification of intracellular proteins by O-GlcNAc and the interplay between O-GlcNAcylation and ubiquitination is still controversial. O-GlcNAcylation can inhibit protein ubiquitination by competing with phosphorylation and other unknown mechanisms, which stabilize numerous nonhistone proteins to maintain their functions [29, 30]. O-GlcNAc is also linked with the histone code and it is important to identify the interactions between O-GlcNAcylation and histone markers. O-GlcNAcylation of H2B at S112, which is sensitive to glucose stimulation, facilitates monoubiquitination at K120 residue by recruiting the BRE1A/1B complex [31]. Moreover, O-GlcNAcylation of the signalling adaptor MAVS on S366 residue is required for K63-linked ubiquitination of MAVS and subsequent downstream signalling activation [21]. Therefore, O-GlcNAcylation can also induce ubiquitination in mammalian cells. However, in this study, we observed that SIRT7 ubiquitination can be directly repressed in the presence of hyper-O-GlcNAcylation. Furthermore, K63-linked ubiquitination, not K48-linked ubiquitination, is repressed by hyper-O-GlcNAcylation. This finding is consistent with a previous study [32]. In line with the above conclusion, our results show that SIRT7 could be associated with K-63-branched but not K-48-branched, which suggests that the OGT promotion of SIRT7 protein stability might not be mediated by ubiquitination. Recently, some proteasomal substrates have been identified to be degraded by Ub-independent proteasome pathway (UIPP). A previous study reported that energy starvation can increase the interaction between REGγ and SIRT7 and induce the degradation of SIRT7 in a ubiquitin-independent manner and that SIRT7 T153 phosphorylation is important for this pathway [27]. In this study, we show that overexpression of OGT or treatment with PugNAc can disrupt the interaction between SIRT7 and REGγ, thus promoting SIRT7 stability. However, the association between the T153 and S136 residues was not investigated in this study.

Additionally, more experiments should be designed to determine the potential functions of other O-GlcNAcylation sites both in vitro and in vivo. Moreover, the associations between O-GlcNAcylation of SIRT7 and other main functions of SIRT7, including ribosome biogenesis, rDNA transcription and stress regulation, should be investigated in the future.

## Conclusion

SIRT7 can be O-GlcNAcylated and promotes pancreatic cancer progression by regulating its stability and deacetylation ability.

## Supplementary information

Supplementary Figures

Supplementary Tables

checklist

contribution form

Supplementary original data

## Data Availability

All data generated or analyzed during this study are included in this published article and its supplementary information files.
