# Supplementary material for: O-GlcNAcylation and stablization of SIRT7 promote pancreatic cancer progression by blocking the SIRT7-REGγ interaction
Source: Cell Death Differ. 2022 Apr 14;29(10):1970–81. doi: 10.1038/s41418-022-00984-3 (PMC9525610; doi:10.1038/s41418-022-00984-3)
Supplement: Supplementary file 1 — Supplementary Figures [file 41418_2022_984_MOESM1_ESM.docx]

**
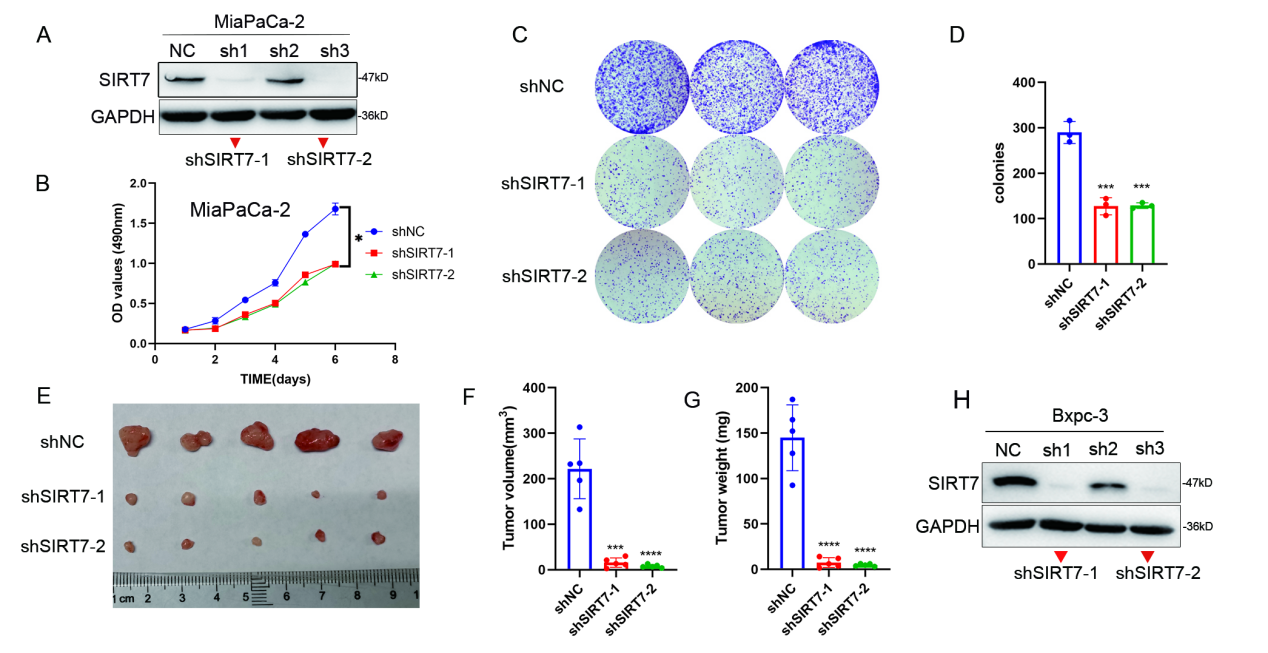
**

**Supplementary Fig.S1 SIRT7 expression affects tumour progression in MiaPaCa-2 cells.**

(A) Western blotting of SIRT7 expression in MiaPaCa-2 cells transfected with shRNAs. One representative experiment of n=3 independent experiments is shown.

(B) MTT assays of MiaPaCa-2 cells transfected with shNC, shSIRT7-1 or shSIRT7-2 plasmids. One representative experiment of n=3 independent experiments is shown.

(C and D) Colony formation assays and statistical analysis of the above groups of MiaPaCa-2 cells. One representative experiment of n=3 independent experiments is shown.

(E-G) The Effects of SIRT7 on tumour xenografts in nude mice. MiaPaCa-2 cells with stable SIRT7 silencing by shRNA (shNC, shSIRT7-1 or shSIRT7-2) were injected subcutaneously into the axillae of nude mice (n= 5 for each group). Mice were sacrificed after 4 weeks, and their tumour masses were excised and weighed. V_tumor_ = 0.5 × L × W^2^. One representative experiment of n=3 independent experiments is shown.

(H) SIRT7 was knocked down by shRNA or shNC in BxPC-3 cells. One representative experiment of n=3 independent experiments is shown.

The data are shown as the means ± SD. Statistical significance was determined by two-tailed t tests (*P < 0.05; **P < 0.01; ***P < 0.001; ****P < 0.0001; NS, no significance)


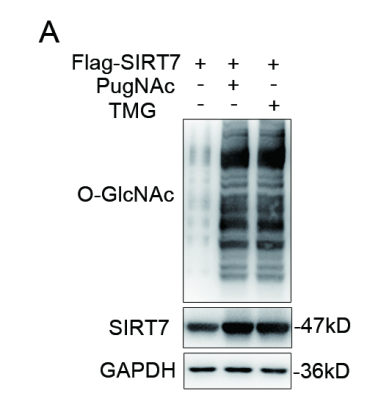


**Supplementary Fig.S2**

(A) The expression levels of SIRT7 and O-GlcNAc were elevated by PugNAc (1 µM, 4 h) and thiamet-G (TMG) (10 µM, 4 h) treatment in 293T cells at the protein level. One representative experiment of n=3 independent experiments is shown.


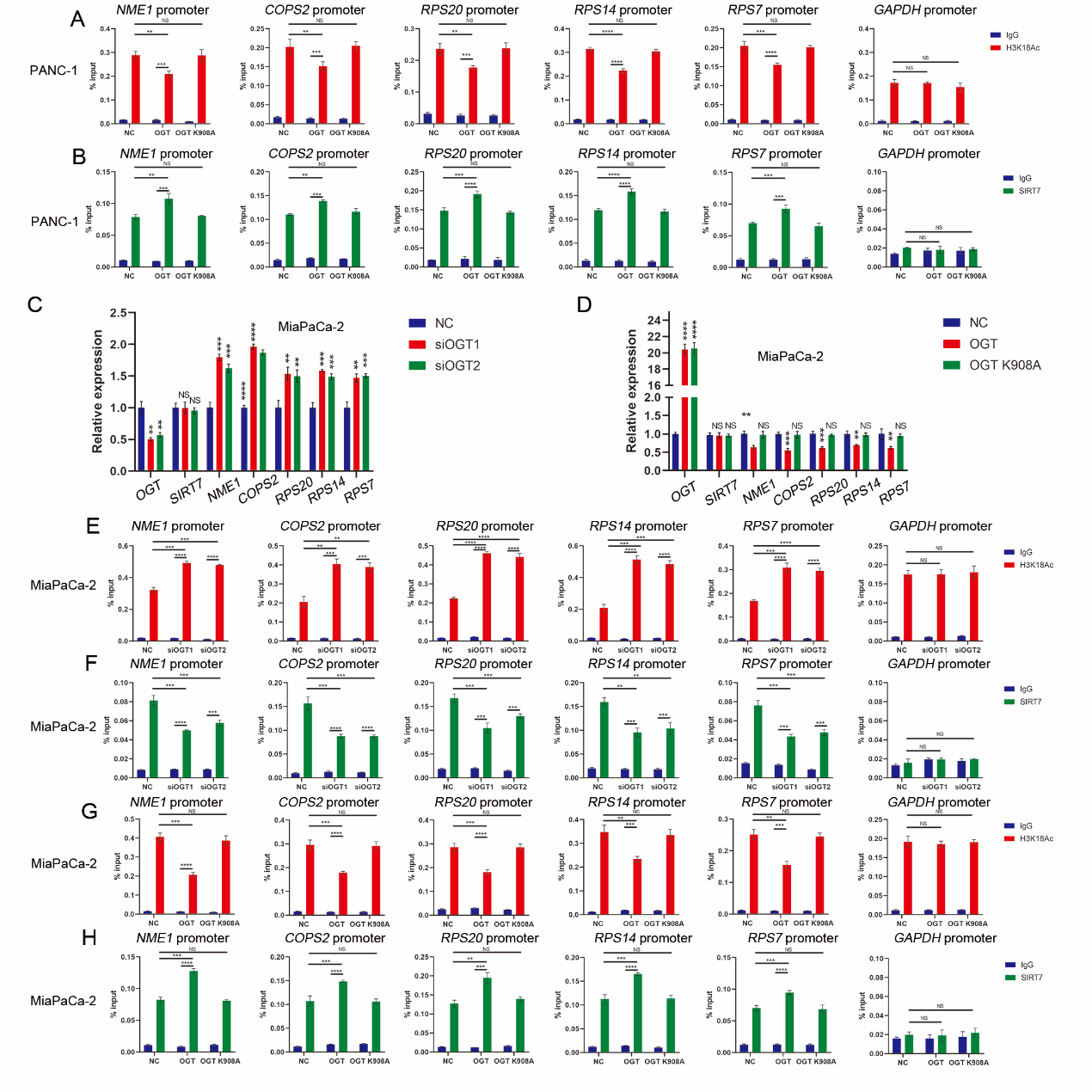


**Supplementary Fig.S3 SIRT7 O-GlcNAcylation represses target genes via hypoacetylation of H3K18.**

1. ChIP-qPCR results showing the occupancy of H3K18Ac at the promoters of SIRT7 target genes in PANC-1 cells transfected with OGT WT or OGT K908A mutant plasmid. One representative experiment of n=3 independent experiments is shown.
2. ChIP-qPCR results showing the occupancy of SIRT7 at the promoters of SIRT7 target genes in PANC-1 cells transfected with OGT WT or OGT K908A mutant plasmid. One representative experiment of n=3 independent experiments is shown.
3. The expression of SIRT7 target genes in the siOGT MiaPaCa-2 cells, as determined by qPCR. One representative experiment of n=3 independent experiments is shown.
4. The expression of SIRT7 target genes in MiaPaCa-2 cells transfected with OGT WT or OGT K908A mutant plasmid, as determined by qPCR. One representative experiment of n=3 independent experiments is shown.
5. ChIP-qPCR results showing the occupancy of H3K18Ac at the promoters of SIRT7 target genes in the siOGT MiaPaCa-2 cells. One representative experiment of n=3 independent experiments is shown.
6. ChIP-qPCR results showing the occupancy of SIRT7 at the promoters of SIRT7 target genes in the siOGT MiaPaCa-2 cells. One representative experiment of n=3 independent experiments is shown.
7. ChIP-qPCR results showing the occupancy of H3K18Ac at the promoters of SIRT7 target genes in MiaPaCa-2 cells transfected with OGT WT or OGT K908A mutant plasmid. One representative experiment of n=3 independent experiments is shown.
8. ChIP-qPCR results showing the occupancy of SIRT7 at the promoters of SIRT7 target genes in MiaPaCa-2 cells transfected with OGT WT or OGT K908A mutant plasmid. One representative experiment of n=3 independent experiments is shown.

The data are shown as the mean ± SD. P values were calculated by two- tailed t tests (*P < 0.05; **P < 0.01; ***P < 0.001; ****P<0.0001; NS, no significance).


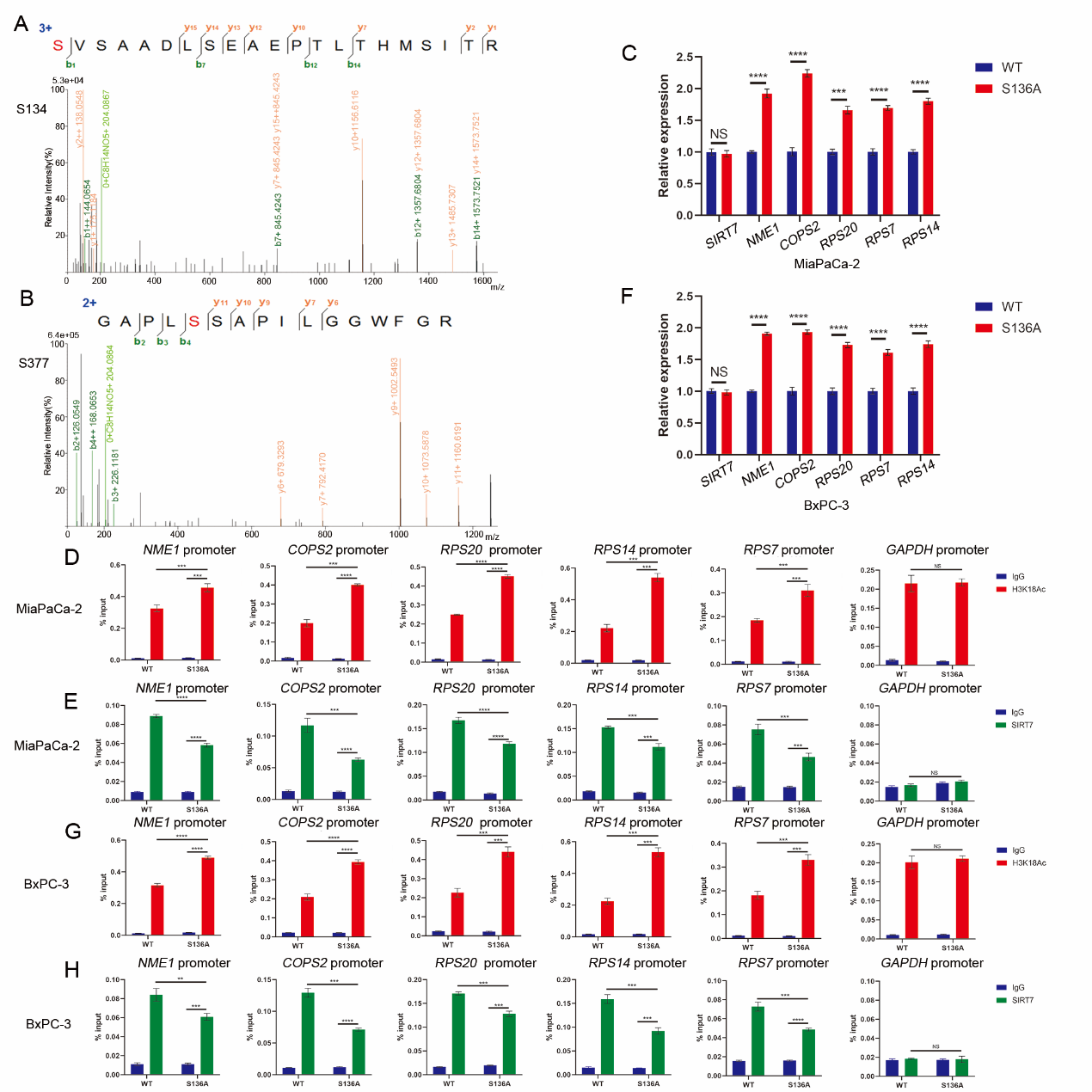


**Supplementary Fig.S4 SIRT7 is O-GlcNAcylated at Ser136.**

(A and B) Detection of the O-GlcNAcylation site(s) on SIRT7. SIRT7 was purified from HEK293T cells and analysed by LC-MS/MS analysis to identify the potential O-GlcNAcylation sites. The serine 134, 377 O-GlcNAcylation sites of SIRT7 were shown.

(C) Expression of SIRT7 target genes in SIRT7 WT and SIRT7 S136A MiaPaCa-2 cells, as determined by qPCR. One representative experiment of n=3 independent experiments is shown.

(D) ChIP-qPCR results showing H3K18Ac occupancy at the promoters of SIRT7 target genes in SIRT7 WT and SIRT7 S136A MiaPaCa-2 cells. One representative experiment of n=3 independent experiments is shown.

(E) ChIP-qPCR results showing SIRT7 occupancy at the promoters of SIRT7 target genes in SIRT7 WT and SIRT7 S136A MiaPaCa-2 cells. One representative experiment of n=3 independent experiments is shown.

(F) Expression of SIRT7 target genes in SIRT7 WT and SIRT7 S136A BxPC-3 cells, as determined by qPCR. One representative experiment of n=3 independent experiments is shown.

(G) ChIP-qPCR results showing H3K18Ac occupancy at the promoters of SIRT7 target genes in SIRT7 WT and SIRT7 S136A BxPC-3 cells. One representative experiment of n=3 independent experiments is shown.

(H) ChIP-qPCR results showing SIRT7 occupancy at the promoters of SIRT7 target genes in SIRT7 WT and SIRT7 S136A BxPC-3 cells. One representative experiment of n=3 independent experiments is shown.

And the data are shown as the mean ± SD. P values were calculated by two-tailed t tests (*P < 0.05; **P < 0.01; ***P < 0.001; ****P<0.0001; NS, no significance)**.**


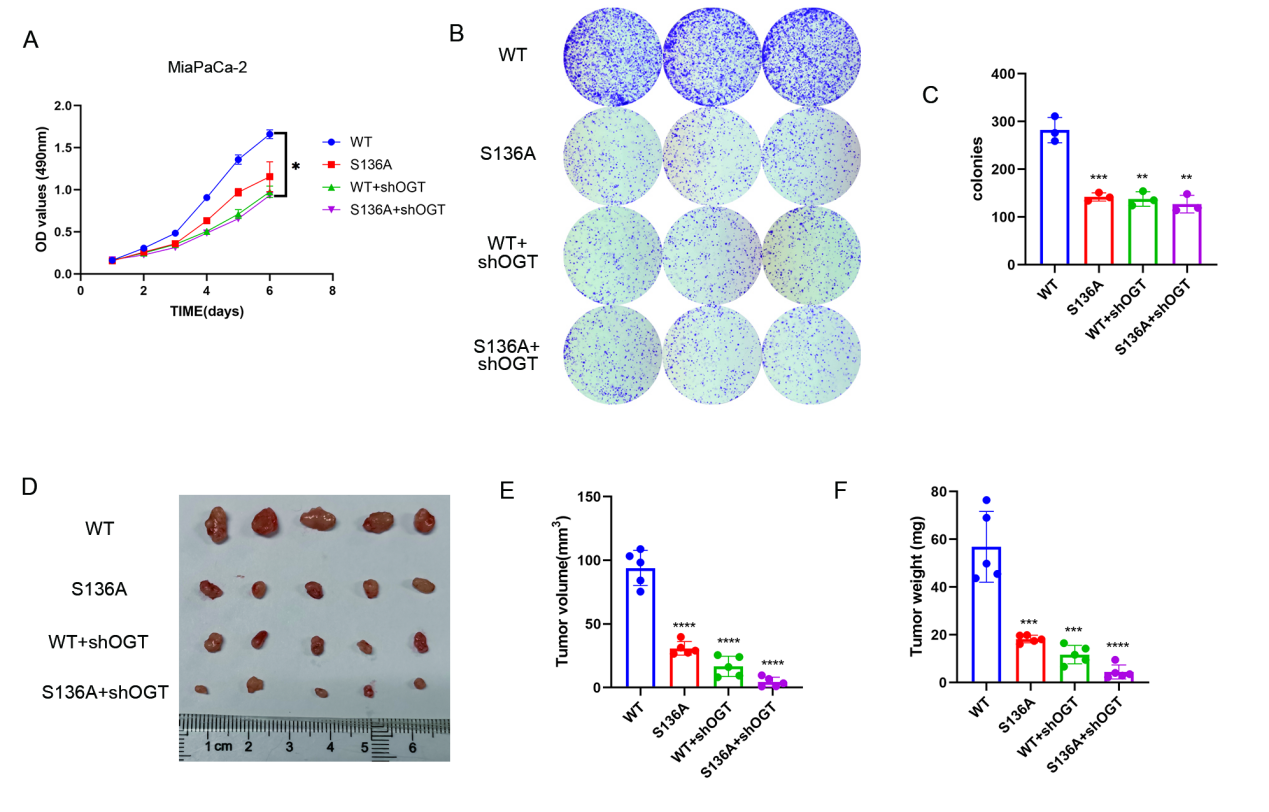


**Supplementary Fig.S5 SIRT7 O-GlcNAcylation on S136 promotes tumour progression in MiaPaCa-2 cells.**

(A) MiaPaCa-2 cells were transfected with shSIRT7 and rescued by SIRT7-WT and S136A. Then, the cells were treated with or without shOGT (WT, S136A, WT+shOGT, S136A+shOGT). MTT assays were performed in the indicated cells. One representative experiment of n=3 independent experiments is shown.

(B and C) Colony formation assays and statistical analysis of the indicated cells. One representative experiment of n=3 independent experiments is shown.

(D-F) The effects of SIRT7 S136A mutation on tumour xenografts in nude mice. Four groups of PANC-1 cells were injected subcutaneously into the axillae of nude mice (n= 5 for each group). Mice were sacrificed after 4 weeks, and their tumour masses were excised and weighed. V_tumor_ = 0.5 × L × W^2^. One representative experiment of n=3 independent experiments is shown.

The data are shown as the mean ± SD. P values were calculated by two-tailed t tests (*P < 0.05; **P < 0.01; ***P < 0.001; ****P<0.0001; NS, no significance).
