# Supplementary material for: O-GlcNAcylation and stablization of SIRT7 promote pancreatic cancer progression by blocking the SIRT7-REGγ interaction
Source: Cell Death Differ. 2022 Apr 14;29(10):1970–81. doi: 10.1038/s41418-022-00984-3 (PMC9525610; doi:10.1038/s41418-022-00984-3)
Supplement: Supplementary file 2 — Supplementary Tables [file 41418_2022_984_MOESM2_ESM.docx]

**Table 1**

The expression of SIRT7 in peri-tumor and PDAC tissues. (P: Chi-square test; P values < 0.05 were considered statistically significant.)

| Variables | SIRT7 expression | | |
| --- | --- | --- | --- |
|  | Low | High | P=0.001 |
| peri-tumor tissues | 42 | 14 |  |
| PDAC tissues | 25 | 31 |  |

**Table 2**

Univariate Kaplan–Meier survival analysis and Multivariate Cox regression model of OS in 56 patients with PDAC. (P1 value: log-rank test, P2: Cox regression test. HR Hazard Ratio, CI confidence interval; P values < 0.05 were considered statistically significant.)

| Variables | N | Univariate analysis of OS(Months) | | | Multivariate Cox analysis of OS(Months) | |  |
| --- | --- | --- | --- | --- | --- | --- | --- |
|  |  | Mean ± SE | 95 % CI | P^1^ | HR (95 % CI) | P^2^ |  |
| Gender |  |  |  | 0.051 |  |  |  |
| Male | 33 | 12.324±1.250 | 9.874-14.773 |  |  |  |  |
| Female | 23 | 17.202±2.611 | 12.084-22.319 |  |  |  |  |
| Age |  |  |  | 0.145 |  |  |  |
| <60 | 27 | 15.815±2.186 | 11.530-20.100 |  |  |  |  |
| >=60 | 29 | 12.551±1.336 | 9.933-15.169 |  |  |  |  |
| Differentiation | |  |  | 0.842 |  |  |  |
| Well | 43 | 14.160±1.558 | 11.106-17.214 |  |  |  |  |
| poorly | 13 | 13.979±2.053 | 9.956-18.003 |  |  |  |  |
|  |  |  |  |  |  |  |  |
| SIRT7 expression |  |  |  | **0.011** |  | **0.032** |  |
| High | 31 | 11.532±1.321 | 8.943-14.120 |  | 1 |  |  |
| Low | 25 | 17.864±2.267 | 13.421-22.306 |  | 0.507（0.272-0.944） |  |  |
| Lymph node metastasis | |  |  | **0.027** |  | 0.068 |  |
| Yes | 25 | 11.406±1.339 | 8.781-14.031 |  | 1 |  |  |
| No | 31 | 16.575±2.083 | 12.579-20.570 |  | 0.565（0.307-1.043） |  |  |
| Classification of TNM | |  |  | **0.005** |  | **0.007** |  |
| I-II | 50 | 14.832±1.343 | 12.200-17.463 |  | 0.257（0.095-0.692） |  |  |
| III-IV | 6 | 6.933±1.719 | 3.563-10.303 |  | 1 |  |  |

**Table 3**

Primers of plasmids.

| Gene | Primer | Sequence 5’→3’ |
| --- | --- | --- |
| SIRT7 | Forward | ATGGCAGCCGGGGGTCTG |
|  | Reverse | TTACGTCACTTTCTTCCTTTTTGTGC |
| SIRT7-S134A | Forward | AAAGGGAGAGCCGTTAGTGCTGCCGACCTG AGC |
|  | Reverse | ACTAACGGCTCTCCCTTTCTGAAGCAGTGTCCA |
| SIRT7-S136A | Forward | AGAAGCGTTGCTGCTGCCGACCTG AGCGAGG |
|  | Reverse | CAGCAGCAACGCTTCTCCCTTTCTGAAGCAG |
| SIRT7-S377A | Forward | ACCGCTTGCCTCGGCCCCCATCCTAGGGGGC |
|  | Reverse | GGCCGAGGCAAGCGGTGCACCCCGGTCCCCA |
| SIRT7-ΔN | Forward | ACGACGATGACAAAGAATTCCGGGAGCTGGCCAGCGCCGTC |
|  | Reverse | GCCCTCTAGACTCGAGCG |
| SIRT7-CC | Forward | ACGACGATGACAAAGAATTCCGGGAGCTGGCCAGCGCCGTC |
|  | Reverse | GCCCTCTAGACTCGAGCGGCCGCTTAGATCTCCAAGCCCAGCTCG |
| SIRT7-ΔC | Forward | GGACGACGATGACAAAGAATTC |
|  | Reverse | GCCCTCTAGACTCGAGCGGCCGCTTAGATCTCCAAGCCCAGCTCG |
| OGT | Forward | ATGGCGTCTTCCGTGGGC |
|  | Reverse | TTATGCTGACTCAGTGACTTCAACA |
| OGT-TPR | Forward | AGGAGATCTGCCGCCGCGATCG |
|  | Reverse | TCGAGCGGCCGCGTACGCGTCAGGCAATGAGCCAAGTTAC |
| OGT-ΔTPR | Forward | AGGAGATCTGCCGCCGCGATCGCCATGCAGATTGTCTGTGATTGGACAG |
|  | Reverse | TCGAGCGGCCGCGTACGCGTTTTGGTGTTGAACAGAGGGCTAG |
| OGT-TPR-NLS | Forward | AGGAGATCTGCCGCCGCGATCG |
|  | Reverse | TCGAGCGGCCGCGTACGCGTAGGCAACCTATTCTTCTCTAACTG |
| OGT-ΔCAT | Forward | AGGAGATCTGCCGCCGCGATCG |
|  | Reverse | TCGAGCGGCCGCGTACGCGTCAGGTATTCTAGATCAGTTCCCAGCTTC |
| HA-OGT 908A | Forward | TGCTCCTGCAGAGGAACACG |
|  | Reverse | GTTCCTCTGCAGGAGCAACAG |

**Table 4**

Primers of siRNAs.

| siRNA | Primer | Sequence 5’→3’ |
| --- | --- | --- |
| Negative control | Forward | UUCUCCGAACGUGUCACGUTT |
|  | Reverse | ACGUGACACGUUCGGAGAATT |
| SiOGT-1 | Forward | GCCUGAUAGAUCUGGCAAUTT |
|  | Reverse | AUUGCCAGAUCUAUCAGGCTT |
| SiOGT-2 | Forward | CGCGUGCCAUCCAAAUUAATT |
|  | Reverse | UUAAUUUGGAUGGCACGCGTT |
| SiREGγ | Forward | CGAAGGUUGGAUGAGUGUGAA |
|  | Reverse | UUCACACUCAUCCAACCUUCG |

**Table 5**

Targets and primers of shRNAs.

SIRT7 shRNA targets

| NO. | Target Sequences |
| --- | --- |
| NC | TTCTCCGAACGTGTCACGT |
| 1 | GCCTGAAGGTTCTAAAGAAGT |
| 2 | GGAAGTGTGATGACGTCATGC |
| 3 | GGGACACCATTGTGCACTTTG |

SIRT7 shRNA primers

| oligo | DNA sequence 5’to 3’ |
| --- | --- |
| Primer-NC-T | gatctGTTCTCCGAACGTGTCACGTTTCAAGAGAACGTGACACGTTCGGAGAATTTTTTc |
| Primer-NC-B | aattgAAAAAATTCTCCGAACGTGTCACGTTCTCTTGAAACGTGACACGTTCGGAGAACa |
| Primer-T1 | GATCCGCCTGAAGGTTCTAAAGAAGTCTCGAGACTTCTTTAGAACCTTCAGGCTTTTTT |
| Primer-B1 | AATTAAAAAAGCCTGAAGGTTCTAAAGAAGTCTCGAGACTTCTTTAGAACCTTCAGGCG |
| Primer-T2 | GATCCGGAAGTGTGATGACGTCATGCCTCGAGGCATGACGTCATCACACTTCCTTTTTT |
| Primer-B2 | AATTAAAAAAGGAAGTGTGATGACGTCATGCCTCGAGGCATGACGTCATCACACTTCCG |
| Primer-T3 | GATCCGGGACACCATTGTGCACTTTGCTCGAGCAAAGTGCACAATGGTGTCCCTTTTTT |
| Primer-B3 | AATTAAAAAAGGGACACCATTGTGCACTTTGCTCGAGCAAAGTGCACAATGGTGTCCCG |

OGT shRNA targets

| NO. | Target Sequences |
| --- | --- |
| NC | TTCTCCGAACGTGTCACGT |
| shOGT | GCCCTAAGTTTGAGTCCAAAT |

OGT shRNA primers

| oligo | DNA sequence 5’to 3’ |
| --- | --- |
| Primer-NC-T | GATCTGTTCTCCGAACGTGTCACGTTTCAAGAGAACGTGACACGTTCGGAGAATTTTTTC |
| Primer-NC-B | AATTGAAAAAATTCTCCGAACGTGTCACGTTCTCTTGAAACGTGACACGTTCGGAGAACA |
| Primer-T | GATCCGCCCTAAGTTTGAGTCCAAATCTCGAGATTTGGACTCAAACTTAGGGCTTTTTT |
| Primer-B | AATTAAAAAAGCCCTAAGTTTGAGTCCAAATCTCGAGATTTGGACTCAAACTTAGGGCG |

**Table 6**

Primers of SIRT7 WT/S134A/S136A/S377A [synonymous mutation](https://www.baidu.com/link?url=6soYzKTEjTciMdCxU0u3L7S967y3lA80cCJpJgMrvbAUpRNjOePX7sgjjRwcA-NN5ZfhHDlW6zKspaaNGrgNE_&wd=&eqid=9dc8fbac0024aba200000004609b3cf8) plasmids

| Primer | Sequence 5’to 3’ |
| --- | --- |
| Primer-F1 | CGCGAATTCGAAGTATACCTCGAGGCCACCATGGCAGCCGGGGGTCTGAGCC |
| Primer-R1 | GAAATGGACGATTGTATCTCGCAGCTGGGTCCCACACTTG |
| Primer-F2 | GATACAATCGTCCATTTCGGGGAGAGGGGGACGTTGG |
| Primer-R2 | GCGATCGCAGATCCTTGGATCCTTACGTCACTTTCTTCCTTTTTGTGCGTTTTG |

**Table 7**

Antibodies/Duolink PLA products used in this study.

| Antibodies | Source | Catalog # |
| --- | --- | --- |
| GAPDH | CST | 5174 |
| O-GlcNAc | CST | 9875 |
| FLAG | HUABIO | M1403-2 |
| HA | HUABIO | 0906-1 |
| MYC | HUABIO | EM31105 |
| Rabbit-IgG | HUABIO | HA1002 |
| Mouse-IgG | HUABIO | HA-1027 |
| SIRT7 | Abcam | ab259968 |
| OGT | CST | 24083 |
| H3 | HUABIO | EM30605 |
| H3K18Ac | CST | 13998 |
| REGγ | proteintech | 14907-1-AP |
| SIRT7 | Invitrogen | MA5-31904 |
| Duolink in Situ PLA Probe Anti-Mouse MINUS | Sigma-Aldrich | [DUO92004](https://www.sigmaaldrich.cn/CN/zh/product/sigma/duo92004) |
| Duolink in Situ PLA Probe Anti-Rabbit PLUS | Sigma-Aldrich | DUO92002 |
| Duolink In Situ Detection Reagents Brightfield | Sigma-Aldrich | DUO92012 |

**Table 8**

Q-PCR primers

| Gene | Primer | Sequence |
| --- | --- | --- |
| *COPS2* | Forward | GTGCGATGATGAGGAGGACT |
|  | Reverse | CACATTTGGCTCGGAGTTACTA |
| *GAPDH* | Forward | AGCCACATCGCTCAGACAC |
|  | Reverse | GCCCAATACGACCAAATCC |
| *NME1* | Forward | CAGCCGGAGTTCAAACCTAA |
|  | Reverse | GCAATGAAGGTACGCTCACA |
| *RPS7* | Forward | TCGCGAGATTTGGGTCTCT |
|  | Reverse | GGCGCTCGAACTGAACAT |
| *RPS14* | Forward | TCGTTCCTGGTCTCAGAAGG |
|  | Reverse | CCTTTCGAGGTGCCATTTT |
| *RPS20* | Forward | AGGGCTGAGGATTTTTGGTC |
|  | Reverse | GGGTGTTTTTCCGGTATCCT |
| *SIRT7* | Forward | TGCCCTCCACAGACACCAGAC |
|  | Reverse | CGCCGCTTCCCAGTTCAAAGG |
| *OGT* | Forward | ATAATACAGCTCTCCGTCTGTG |
|  | Reverse | ATGCTTTACGATACAAGCGAAC |

**Table 9**

CHIP primers

| Target | Primer | Sequence |
| --- | --- | --- |
| *COPS2* promoter | Forward | CGAATCAGCTACAAGTCGAGATAA |
|  | Reverse | CGCCTTCCAGTCCCTCTT |
| *GAPDH*  promoter | Forward | GACCTTCCGTGCAGAAACC |
|  | Reverse | CTGGCTCCTGGCATCTCT |
| *NME1* promoter | Forward | CCGTAATACTTGGCTCTCGAA |
|  | Reverse | GAATAGACCTGCATGAAGTGAGG |
| *RPS7* promoter | Forward | CGGCTGAAAGTAACTCTTGCAT |
|  | Reverse | GCTGTGGACAGGGAATTTAATC |
| *RPS14* promoter | Forward | ACAGGAGGACGGATTGAGC |
|  | Reverse | ACGGGGTCTCCCTGTGTT |
| *RPS20* promoter | Forward | AAGTTCTTTCTTTTTGAGGAAGACG |
|  | Reverse | GAACAGCGGTGAGTCAGGA |
