# Supplementary material for: O-GlcNAcylation and stablization of SIRT7 promote pancreatic cancer progression by blocking the SIRT7-REGγ interaction
Source: Cell Death Differ. 2022 Apr 14;29(10):1970–81. doi: 10.1038/s41418-022-00984-3 (PMC9525610; doi:10.1038/s41418-022-00984-3)
Supplement: Supplementary file 4 — contribution form [file 41418_2022_984_MOESM4_ESM.pdf]

DECLARATION OF CONTRIBUTIONS TO ARTICLE

ADMC

Manuscript Number: CDD-21-2249

Journal Name: *Cell Death & Differentiation* (the 'Journal')

Proposed Title of the Contribution:
O-GlcNAcylation and Stablization of SIRT7 Promote Pancreatic Cancer Progression by Blocking SIRT7-REGâ interaction (the 'Contribution')

Author(s):
Xiaoman He, Yongzhou Li, Qing Chen, Lei Zheng, Jianyao Lou, Chuanshuai Lin, Jiali Gong, Yi Zhu, Yulian Wu (the 'Authors')

For all *CDD* articles, each person named as an author in the published version must be able to show he or she has contributed substantially to the article.

Authorship credit should be based on 1) substantial contributions to conception and design, acquisition of data, or analysis and interpretation of data; 2) drafting the article or revising it critically for important intellectual content; and 3) final approval of the version to be published. Authors should meet conditions 1, 2 and 3.

Any person who cannot be shown to have made a substantial contribution to the article cannot be listed as an author in the final version. The name of any person who is deemed to have made a minor contribution can, however, appear in the Acknowledgments section of the article.

Please complete the table below to indicate the contributions of all named authors to the manuscript.

Author Full Name:	Specification of Contribution to the Manuscript:
Xiaoman He	Conceptualization;Investigation;Methodology;Resources;Formal analysis;Writing - Original Draft;Writing - Review & Editing;Final approval of the version to be published.
Yongzhou Li	Visualization;Writing - Review & Editing;Final approval of the version to be published.
Qing Chen	Software;Writing - Review & Editing;Final approval of the version to be published.
Lei Zheng	Formal analysis;Writing - Review & Editing;Final approval of the version to be published.
Jianyao Lou	Resources;Writing - Review & Editing;Final approval of the version to be published.
Chuanshuai Lin	Data Curation;Writing - Review & Editing;Final approval of the version to be published.
Jiali Gong	Data Curation;Writing - Review & Editing;Final approval of the version to be published.
Yi Zhu	Conceptualization;Investigation;Writing - Original Draft;Writing - Review & Editing; Funding acquisition;Final approval of the version to be published.
Yulian Wu	Data Interpretation;Supervision;Writing - Review & Editing;Funding acquisition;Final approval of the version to be published.

扫描全能王 创建

Please complete the table below to indicate the contributions of all named authors to the figures.

Figure 1:

Xiaoman He generated the data(A-K). Yongzhou Li and Qing Chen assembled the figure(F,H,J,K). Jianyao Lou collected patient information(Figure A-C). Yi Zhu and Yulian Wu helped with the methodology(A,I).

Figure 2:

Xiaoman He generated the data(A-J). Lei Zheng labelled the image and assembled the figure (B,C,I,J). Yi Zhu and Yulian Wu helped provide the experimental platform(E-F).

Figure 3:

Xiaoman He generated the data(A-I). Chuanshuai Lin and Jiali Gong anzlyzed the data(B,E).

Figure 4:

Xiaoman He generated the data(A-G).Yongzhou Li and Qing Chen assembled the figure(A-G).

Figure 5:

Xiaoman He generated the data(A-G).Chuanshuai Lin and Jiali Gong anzlyzed the data(D,G).Yongzhou Li and Qing Chen assembled the figure(A-G).

Figure 6:

Xiaoman He generated the data(A-J).Chuanshuai Lin and Jiali Gong anzlyzed the data(H-J).

Signed for and on behalf of the Author(s):	Print Name:	Date:
Yulian Wu	Yulian Wu	2022-02-15

扫描全能王 创建
