# Supplementary material for: O-GlcNAcylation and stablization of SIRT7 promote pancreatic cancer progression by blocking the SIRT7-REGγ interaction
Source: Cell Death Differ. 2022 Apr 14;29(10):1970–81. doi: 10.1038/s41418-022-00984-3 (PMC9525610; doi:10.1038/s41418-022-00984-3)

Original western blots

CDD-21-2249

Figure 1D GAPDH

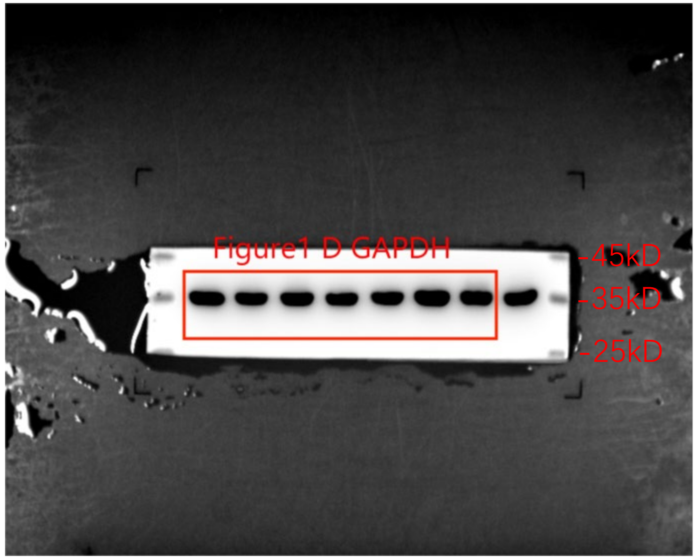

Figure 1D SIRT7

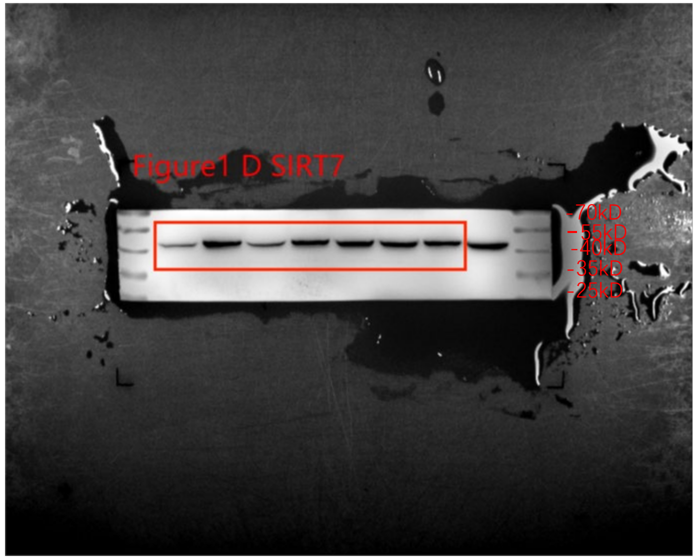

Figure 1E PANC-1 GAPDH

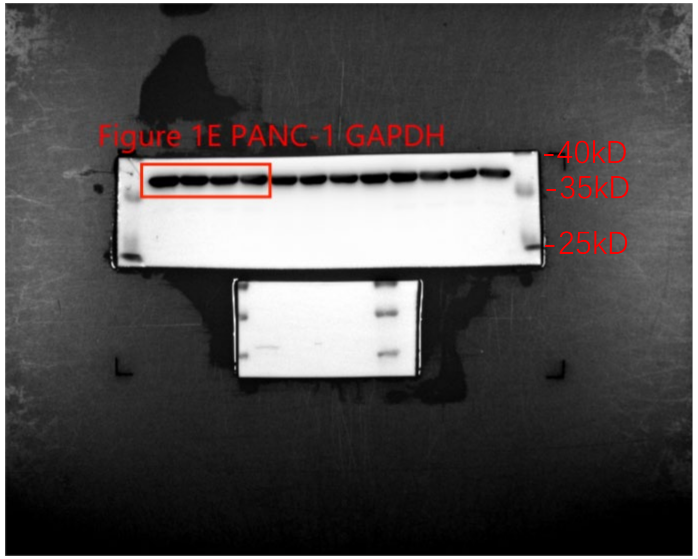

Figure 1E PANC-1 SIRT7

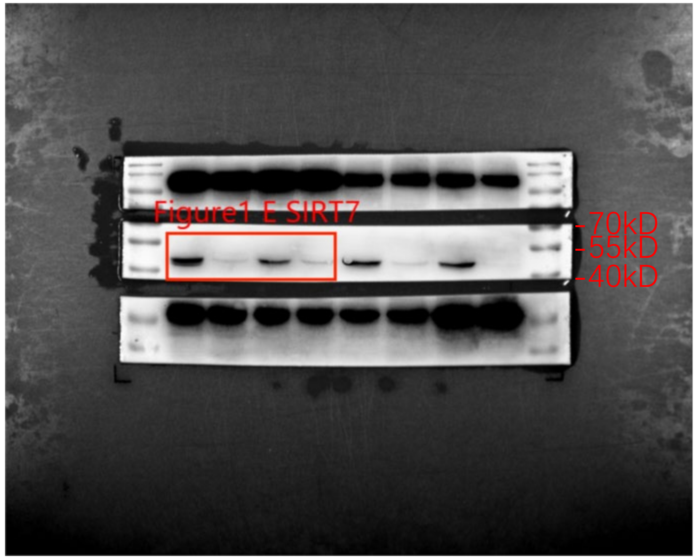

Figure 2B Flag

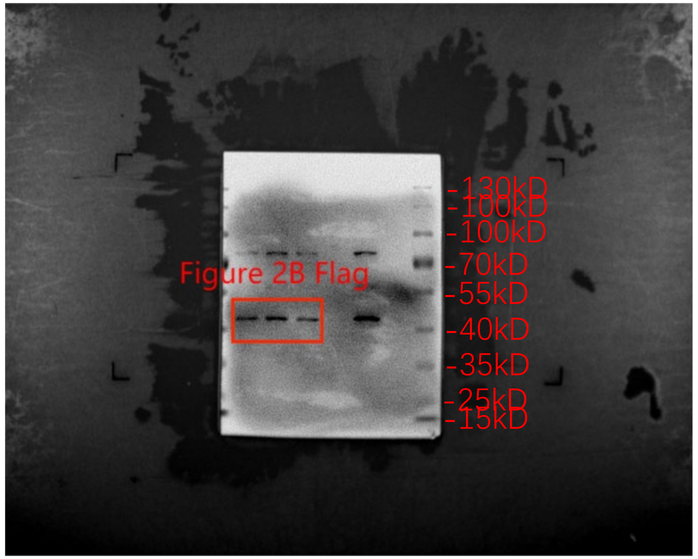

Figure 2B GAPDH

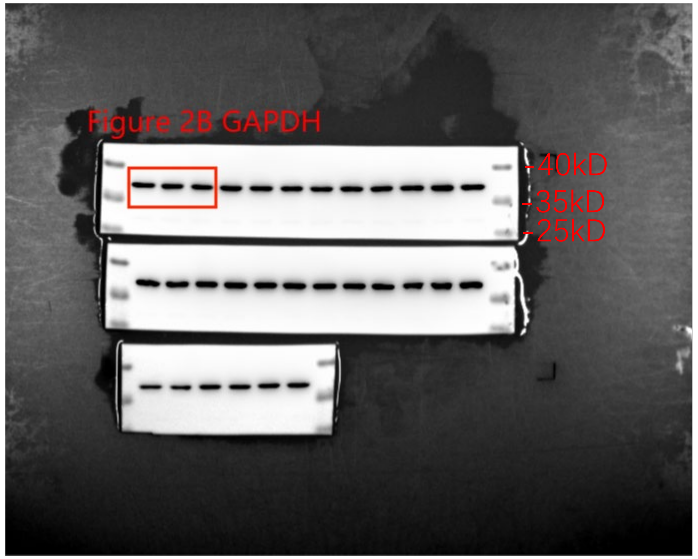

Figure 2B HA

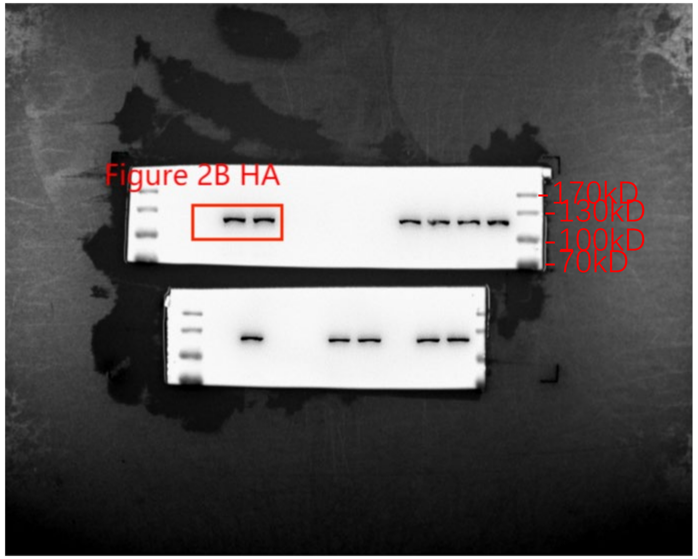

Figure 2B IP HA IB Flag

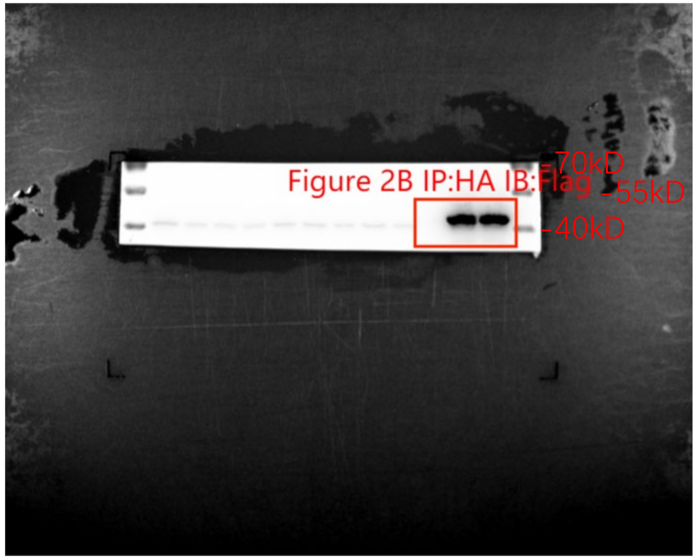

Figure 2B IP O-GlcNAc IB Flag

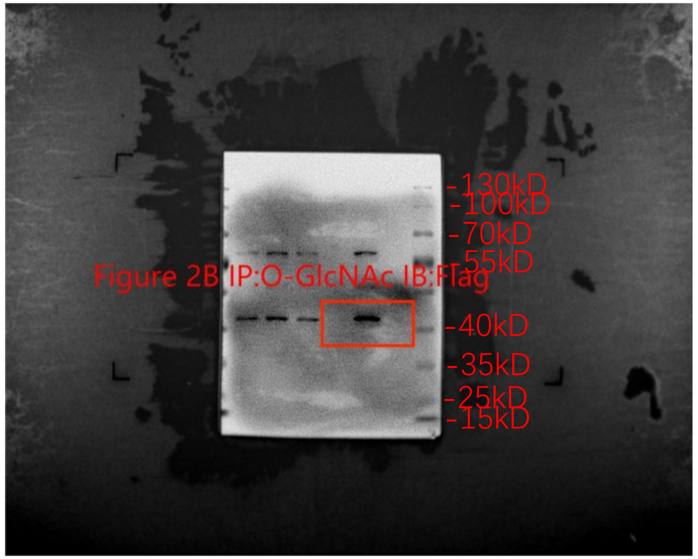

Figure 2B O-GlcNAc

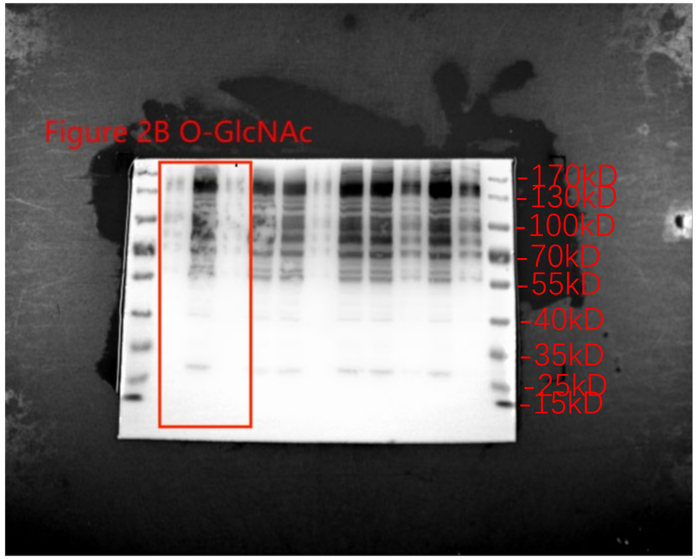

Figure 2C PANC-1 GAPDH

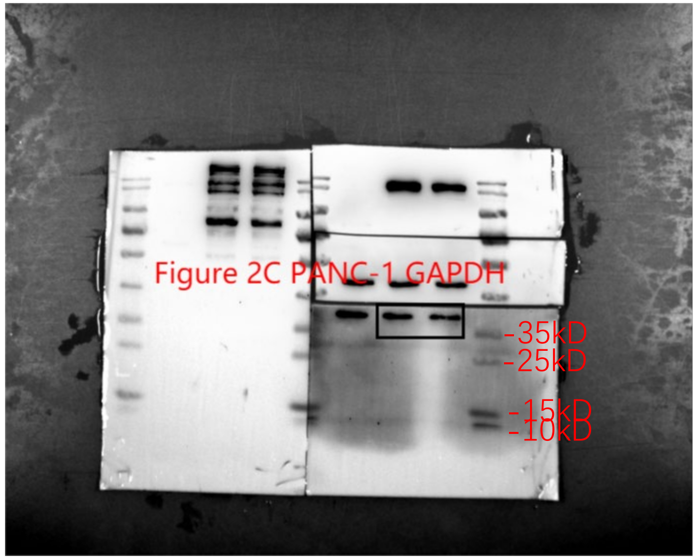

Figure 2C PANC-1 IP O-GlcNAc IB SIRT7

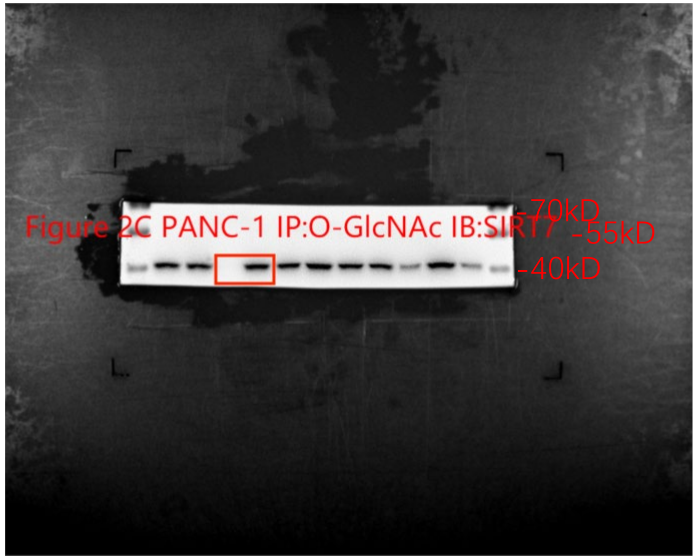

Figure 2C PANC-1 IP OGT IB SIRT7

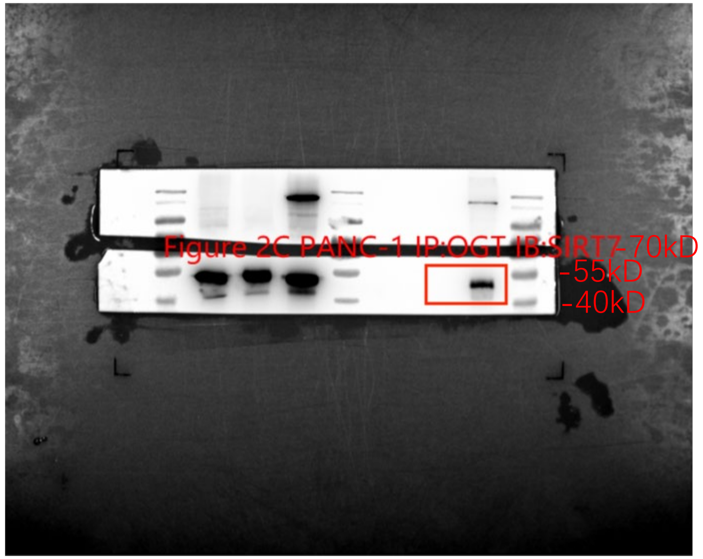

Figure 2C PANC-1 IP SIRT7 IB OGT

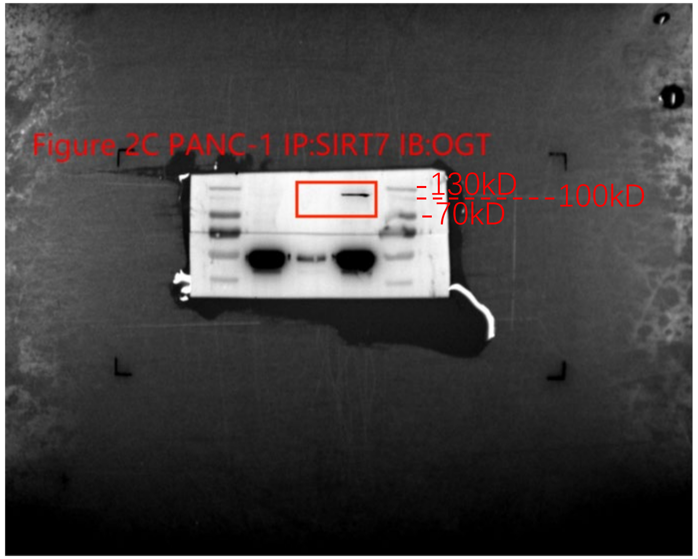

Figure 2C PANC-1 O-GlcNAc

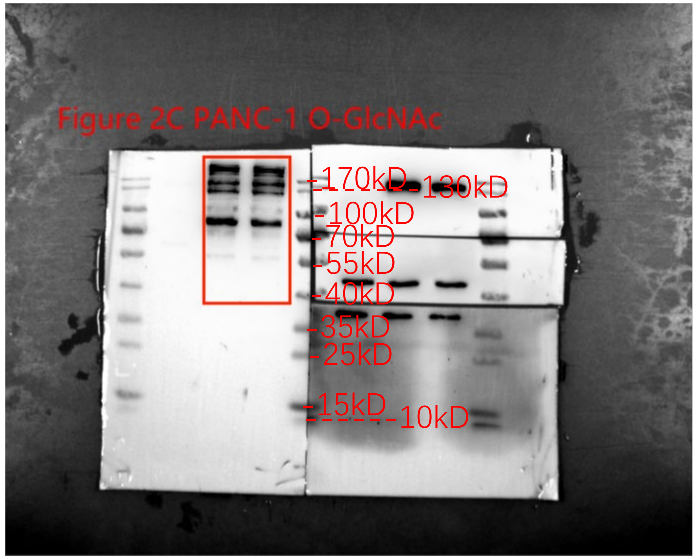

Figure 2C PANC-1 OGT

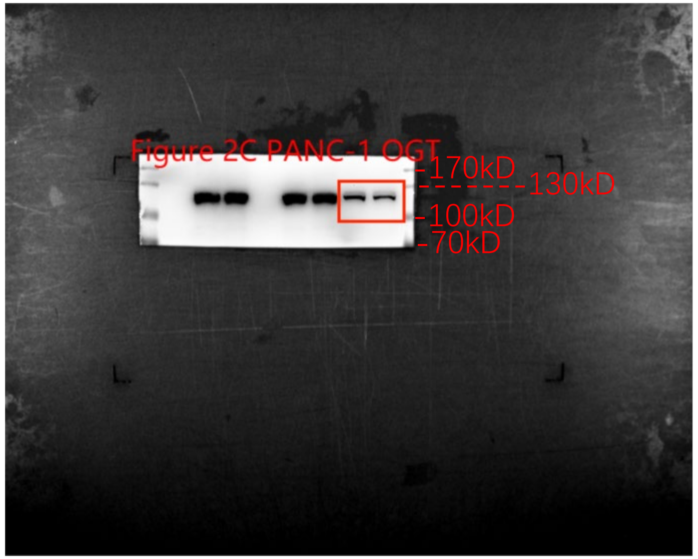

Figure 2C PANC-1 SIRT7

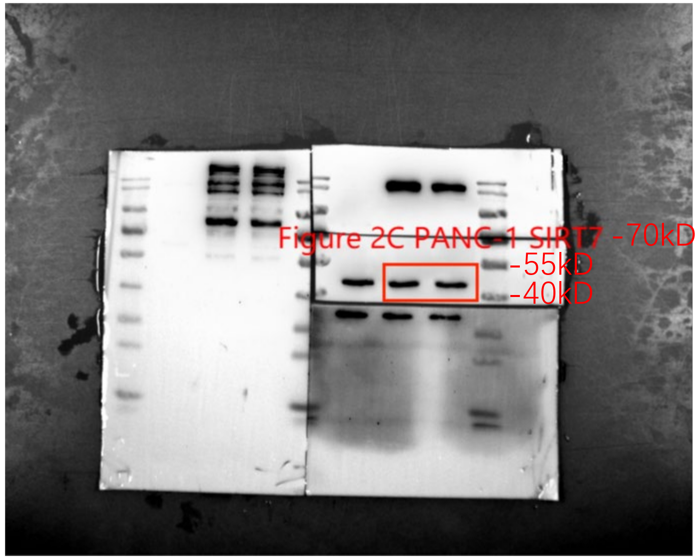

Figure 2C MiaPaCa-2 GAPDH

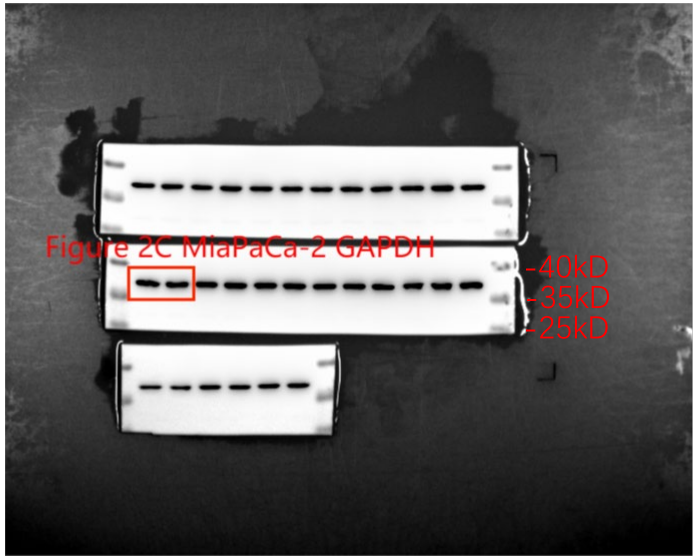

Figure 2C MiaPaCa-2 IP O-GlcNAc IB SIRT7

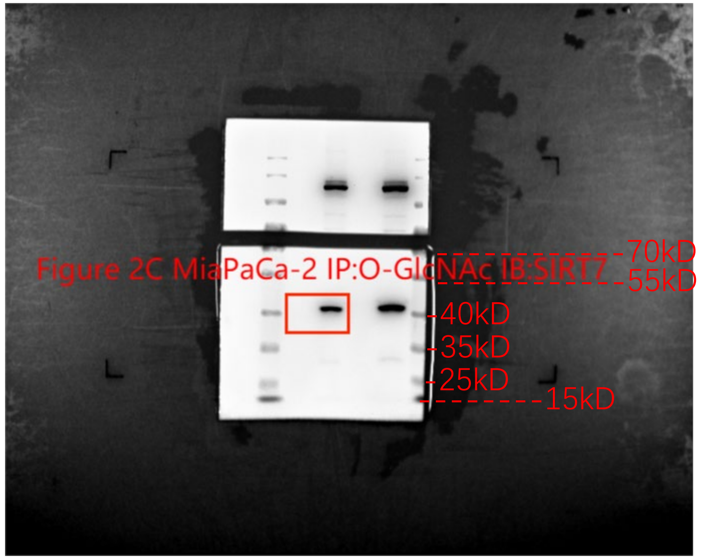

Figure 2C MiaPaCa-2 IP OGT IB SIRT7

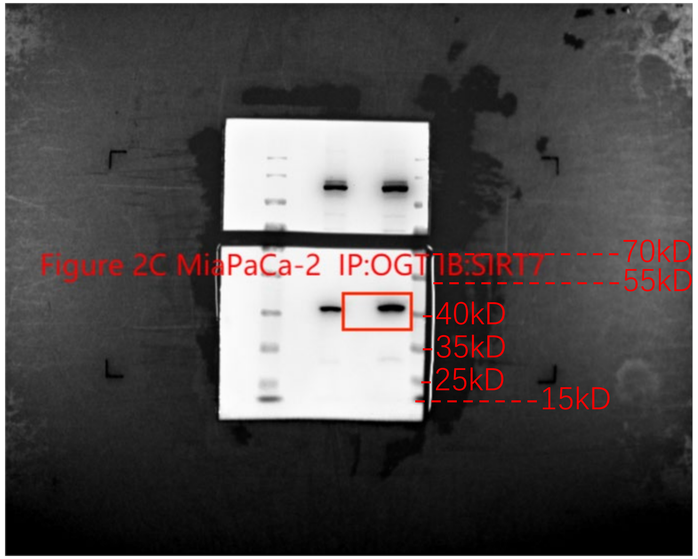

Figure 2C MiaPaCa-2 IP SIRT7 IB OGT

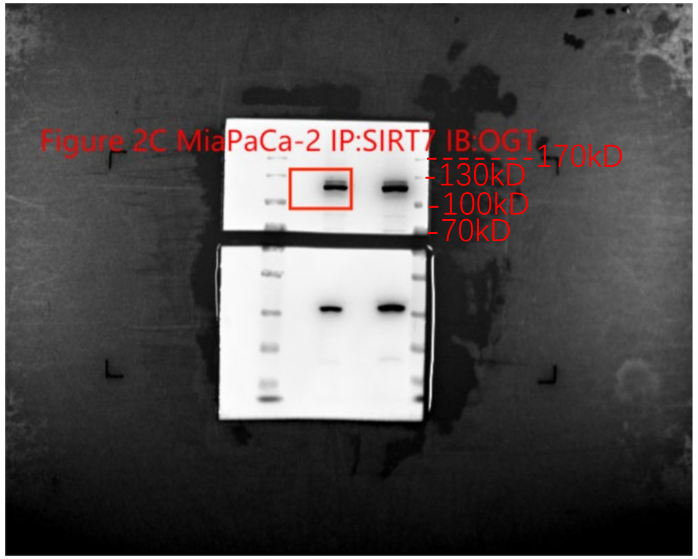

Figure 2C MiaPaCa-2 O-GlcNAc

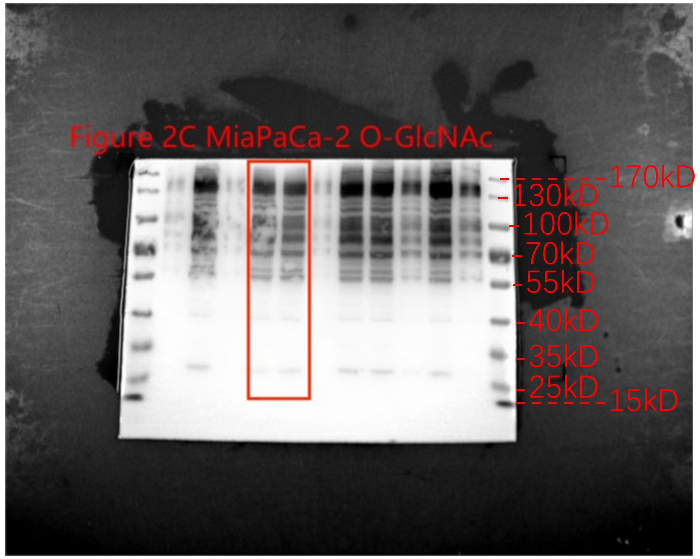

Figure 2C OGT

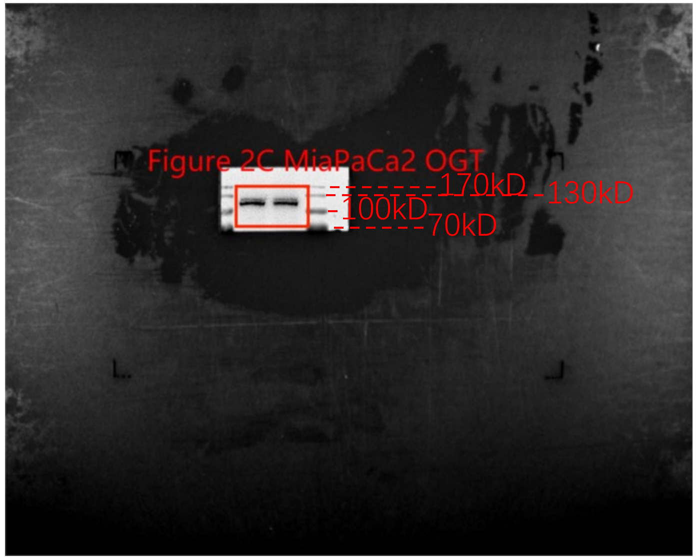

Figure 2C MiaPaCa-2 SIRT7

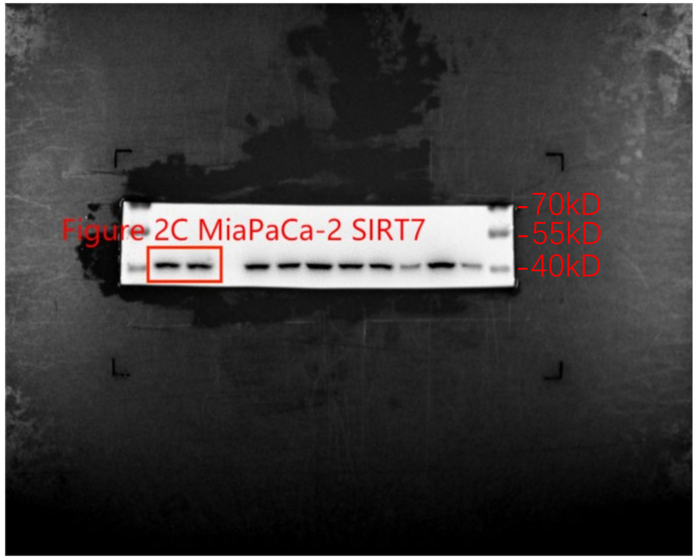

Figure 2H Flag

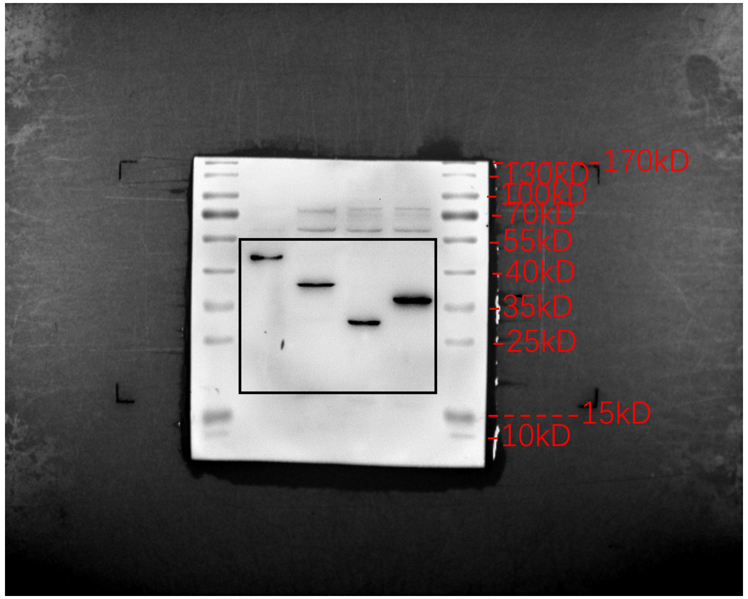

Figure 2H GAPDH

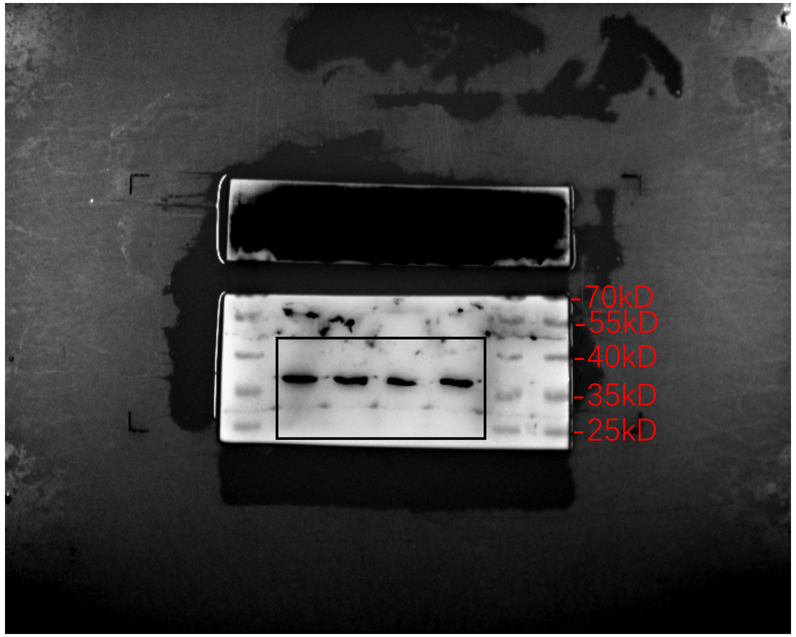

Figure 2H HA

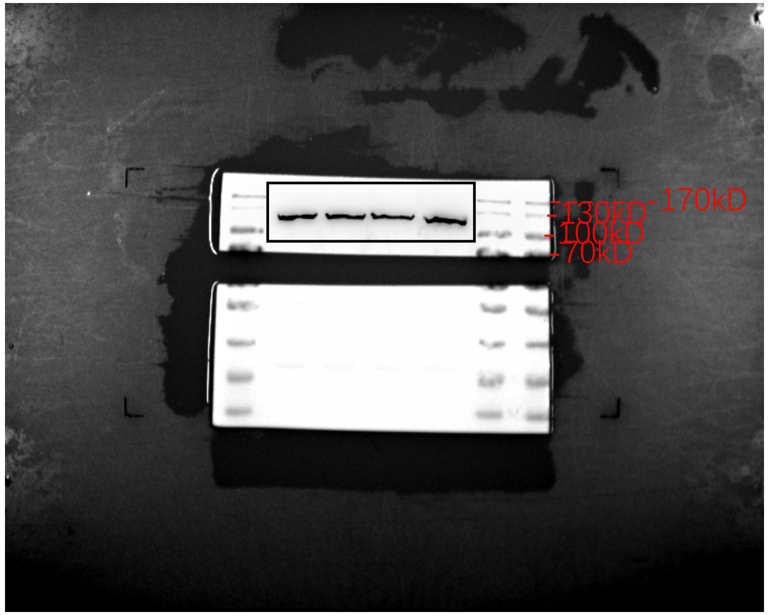

Figure 2H IP Flag IB HA

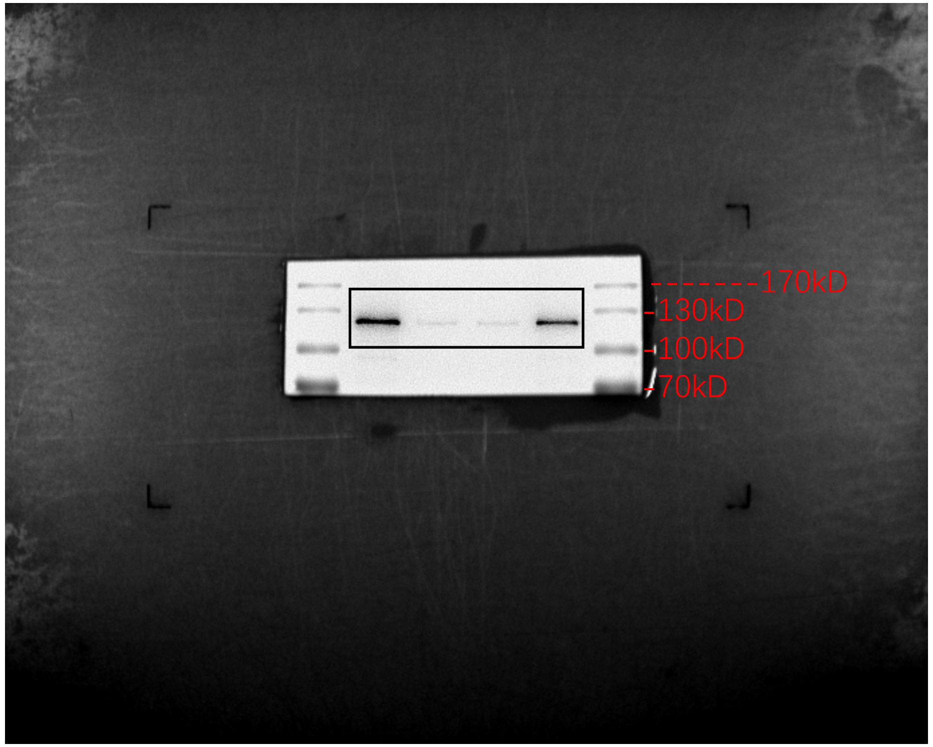

Figure 2I Flag

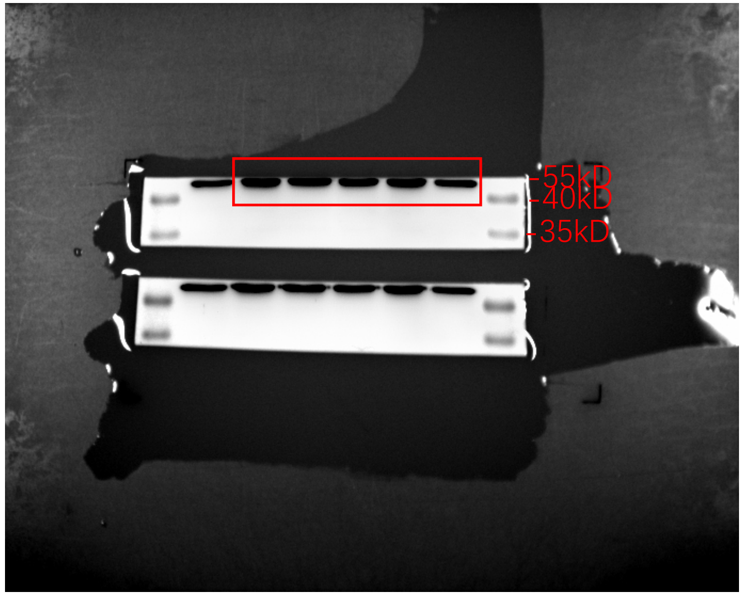

Figure 2I GAPDH

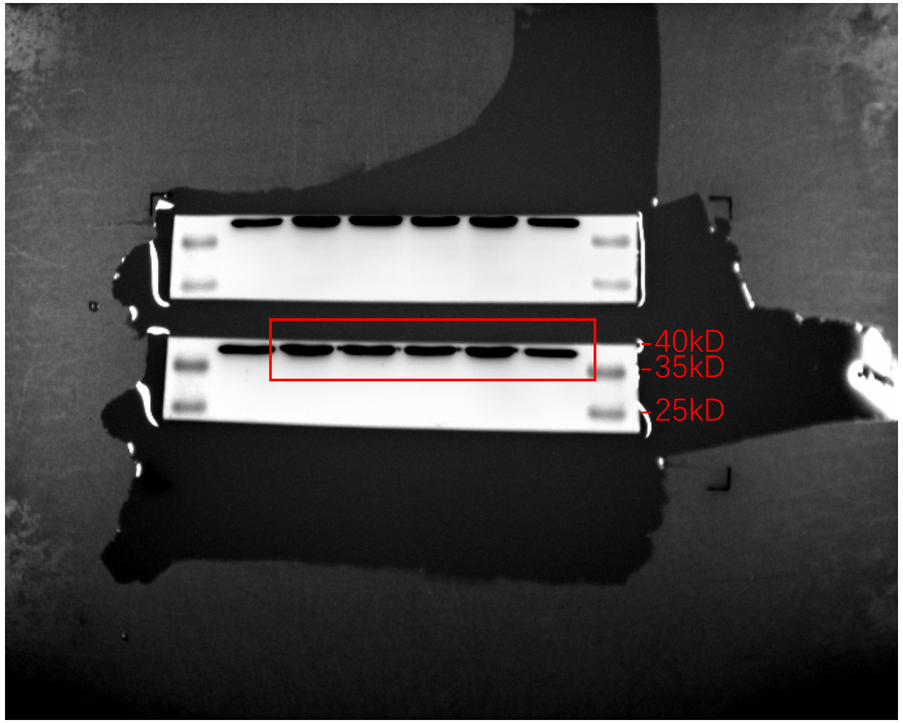

Figure 2I HA

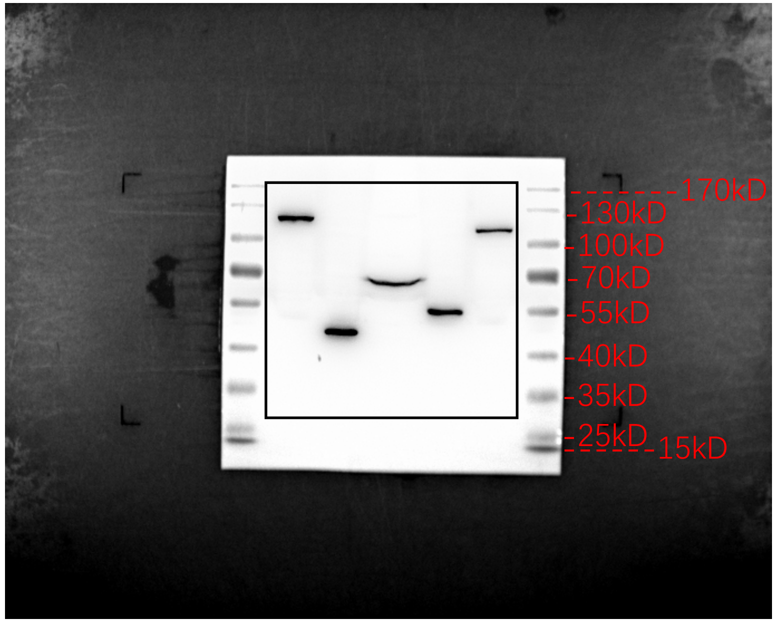

Figure 2I IP HA IB Flag

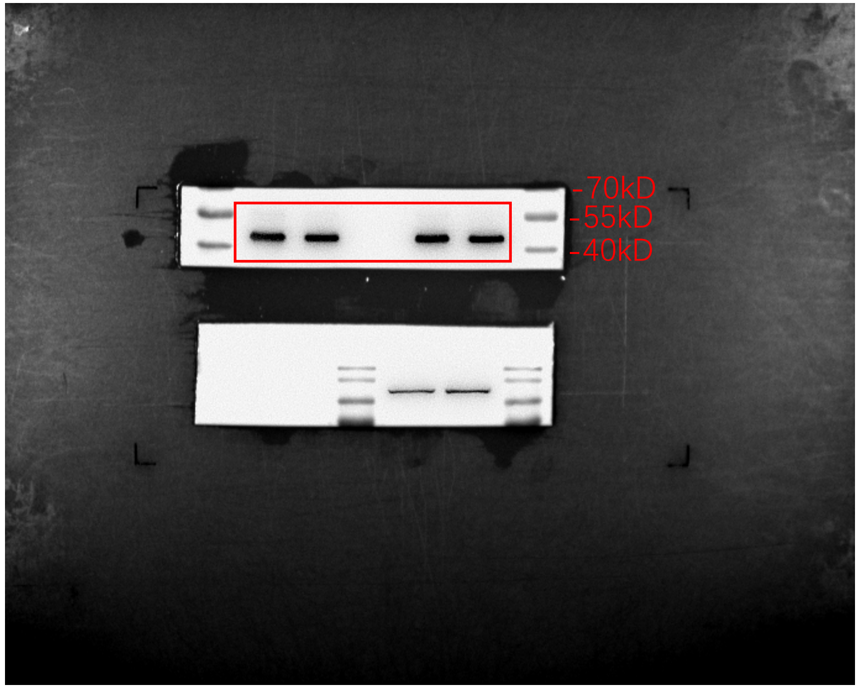

Figure 3A PANC-1 GAPDH

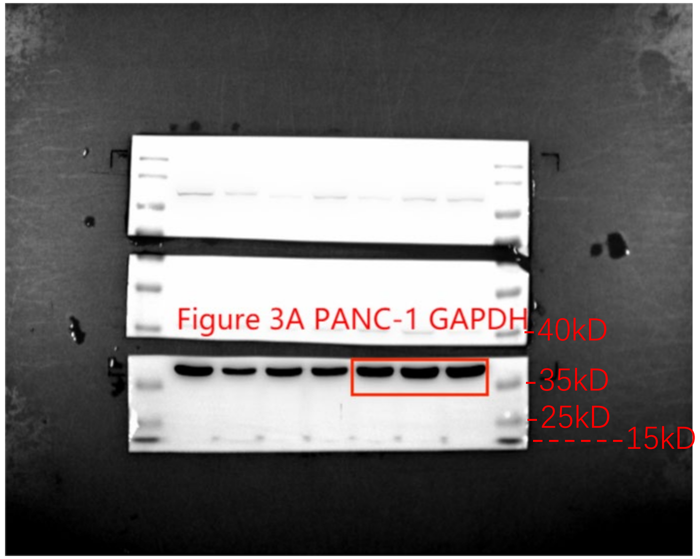

Figure 3A PANC-1 SIRT7

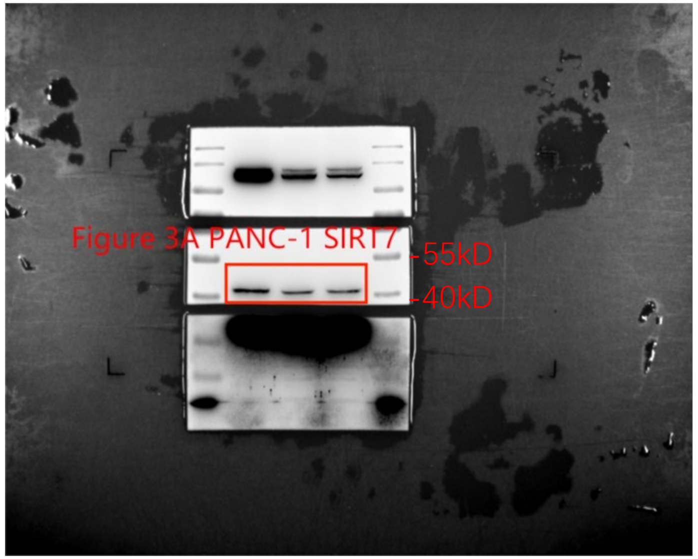

Figure 3A PANC-1 OGT

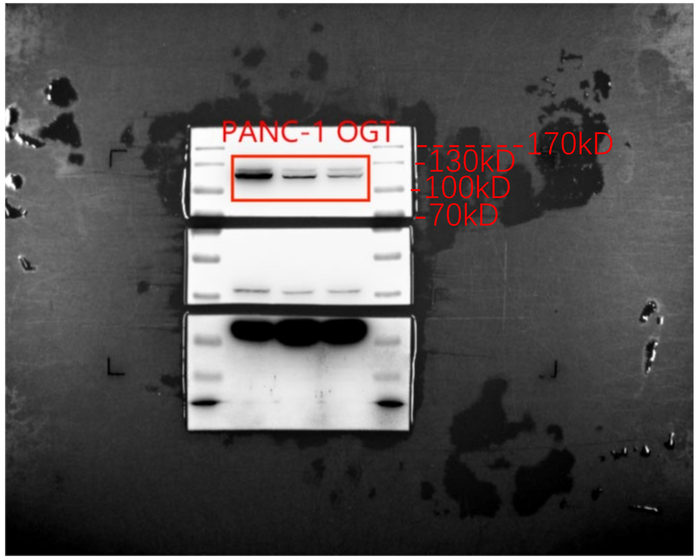

Figure 3A MiaPaCa-2 GAPDH

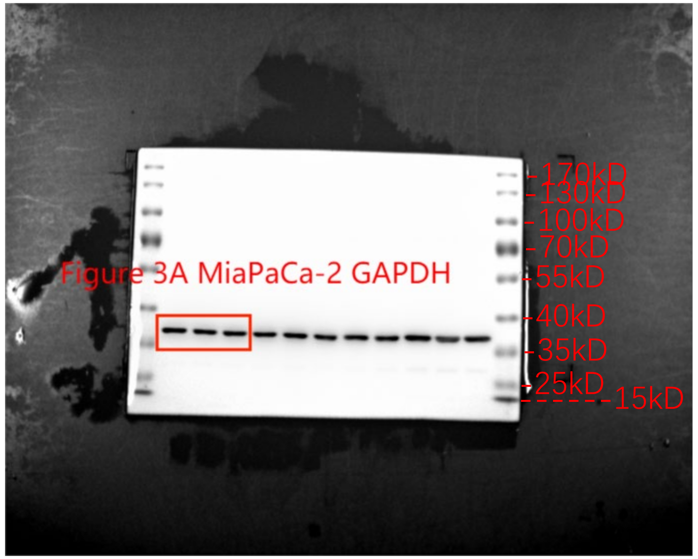

Figure 3A MiaPaCa-2 SIRT7

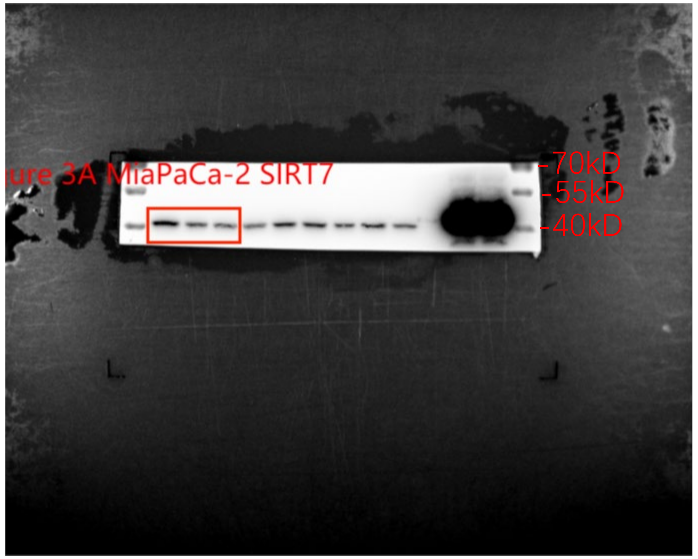

Figure 3A MiaPaCa-2 OGT

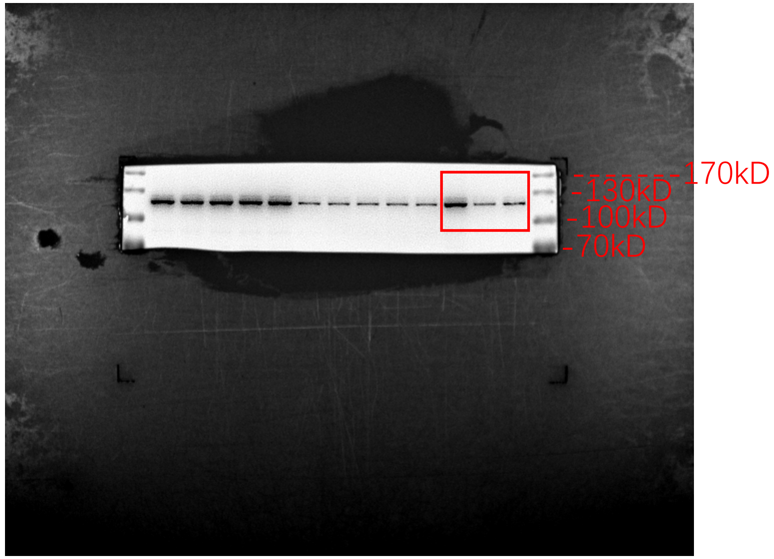

Figure 3C PANC-1 GAPDH

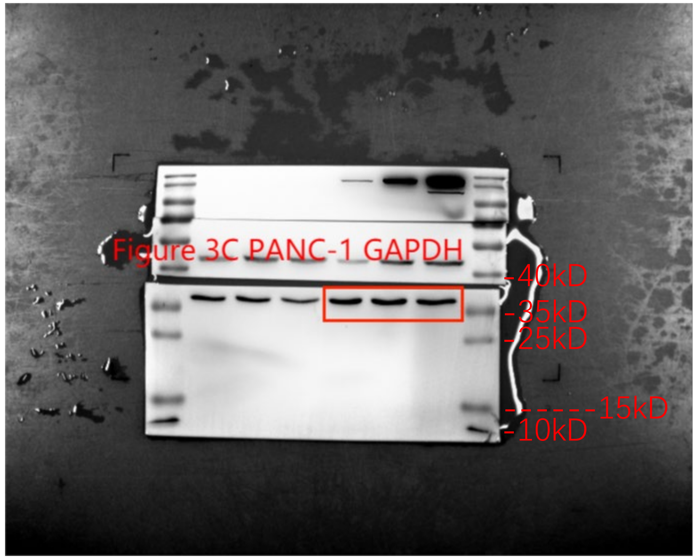

Figure 3C PANC-1 O-GlcNAc

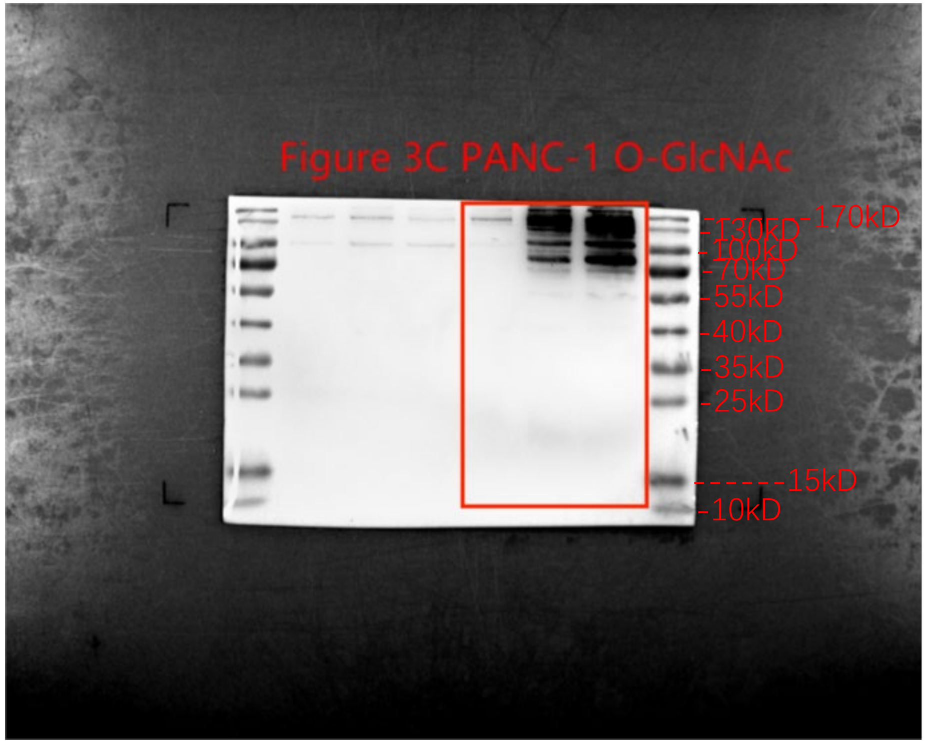

Figure 3C PANC-1 SIRT7

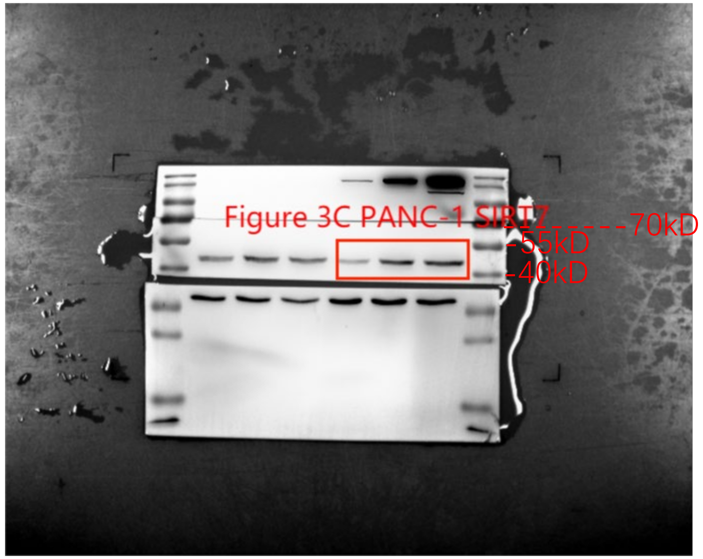

Figure 3C MiaPaCa-2 GAPDH

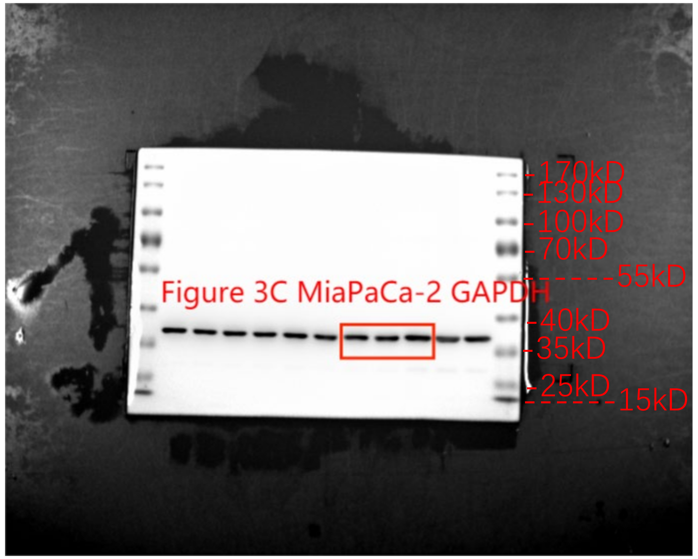

Figure 3C MiaPaCa-2 O-GlcNAc

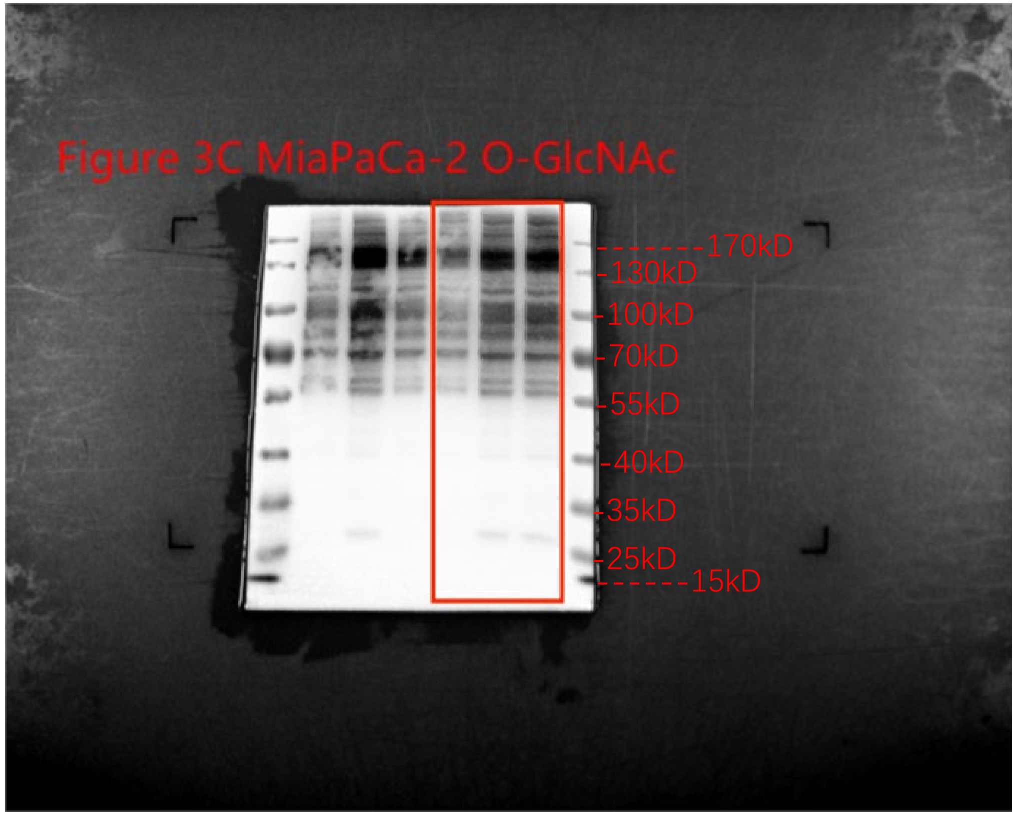

Figure 3C MiaPaCa-2 SIRT7

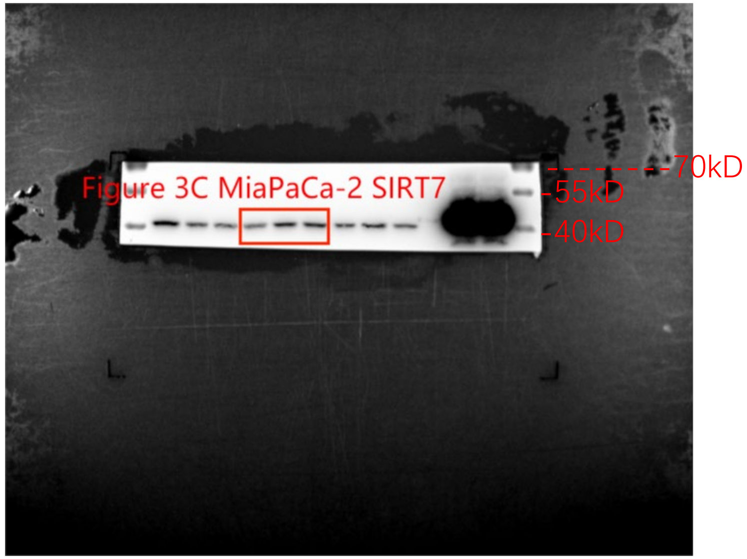

Figure 3D PANC-1 O-GlcNAc

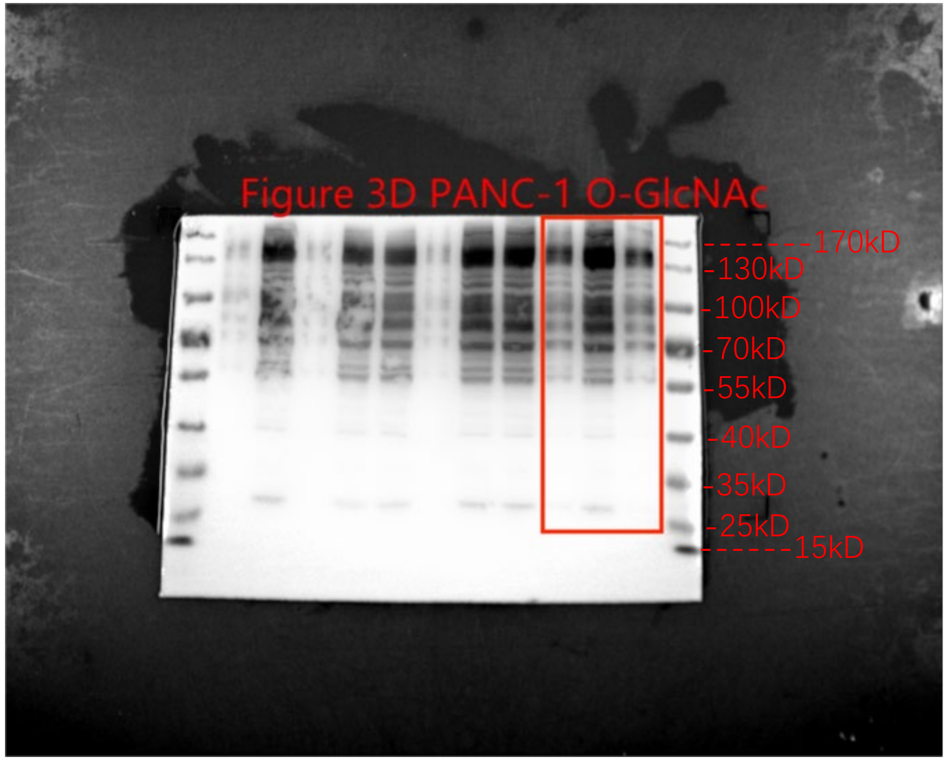

Figure 3D PANC-1 SIRT7

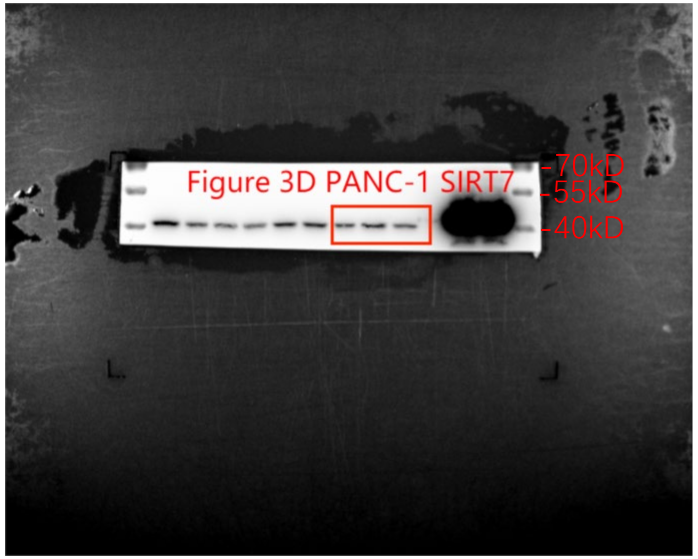

Figure 3D PANC-1 GAPDH

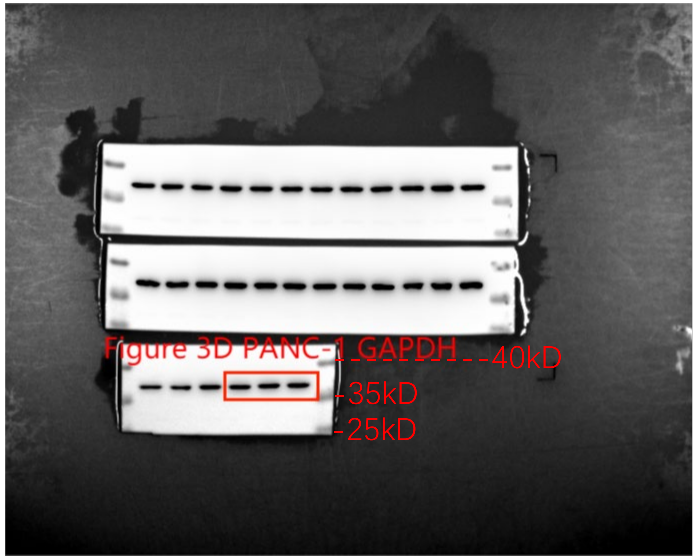

Figure 3D PANC-1 HA

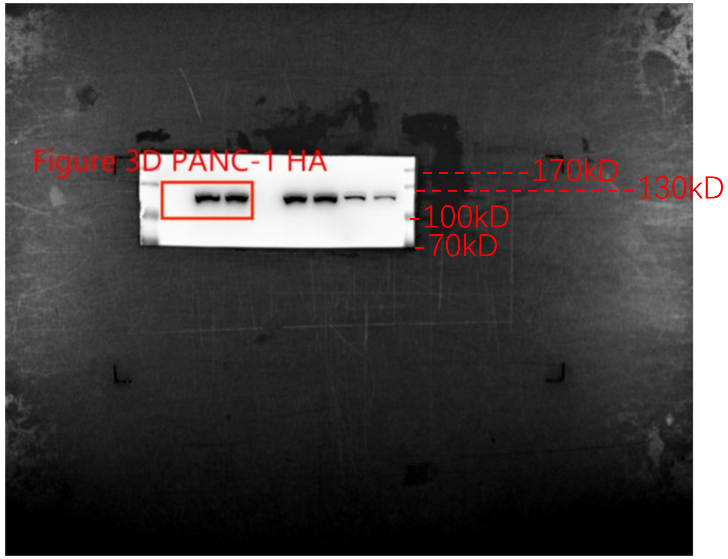

Figure 3D MiaPaCa-2 O-GlcNAc

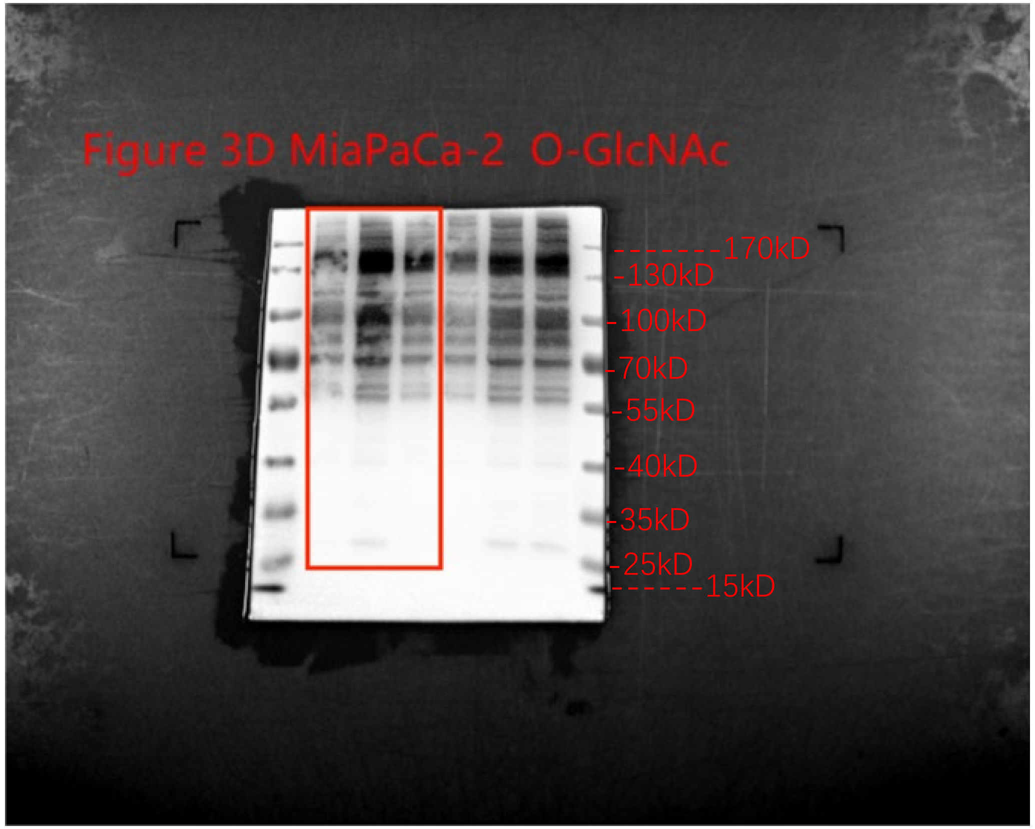

Figure 3D MiaPaCa-2 SIRT7

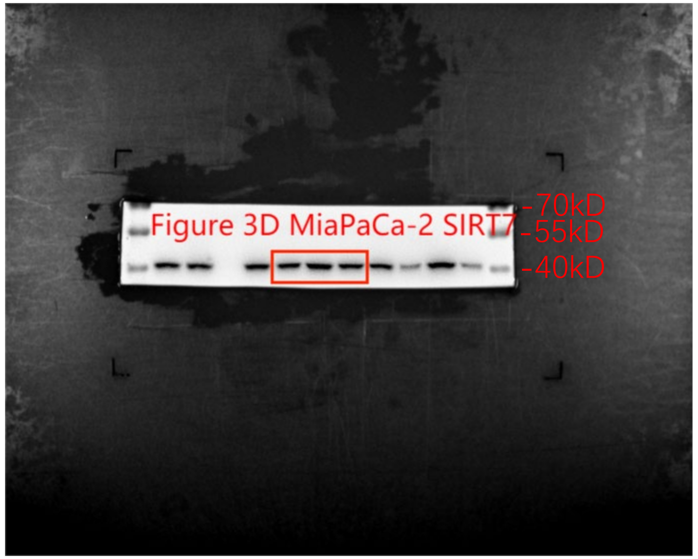

Figure 3D MiaPaCa-2 GAPDH

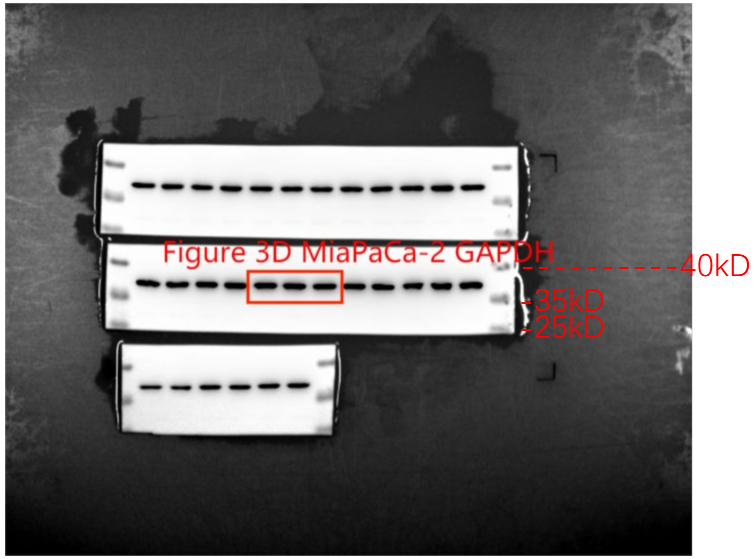

Figure 3D MiaPaCa-2 HA

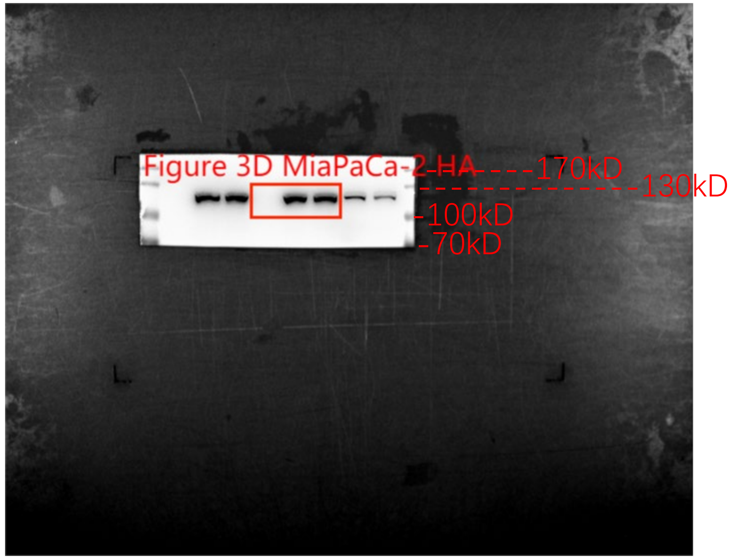

Figure 3F NC OGT

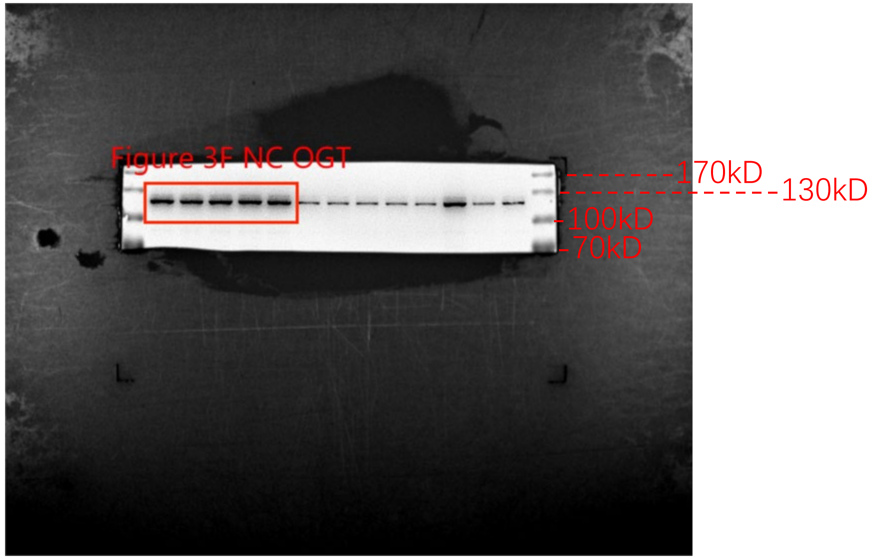

Figure 3F siOGT1 OGT

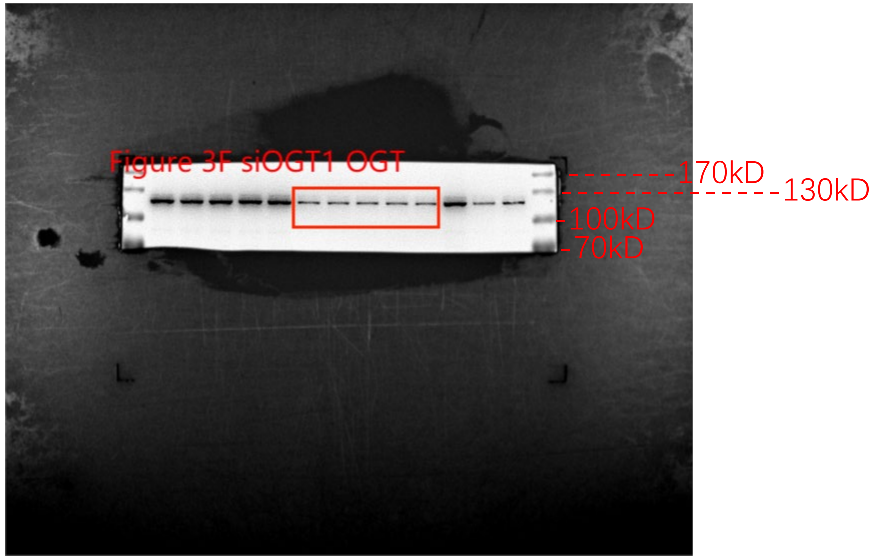

Figure 3F siOGT2 OGT

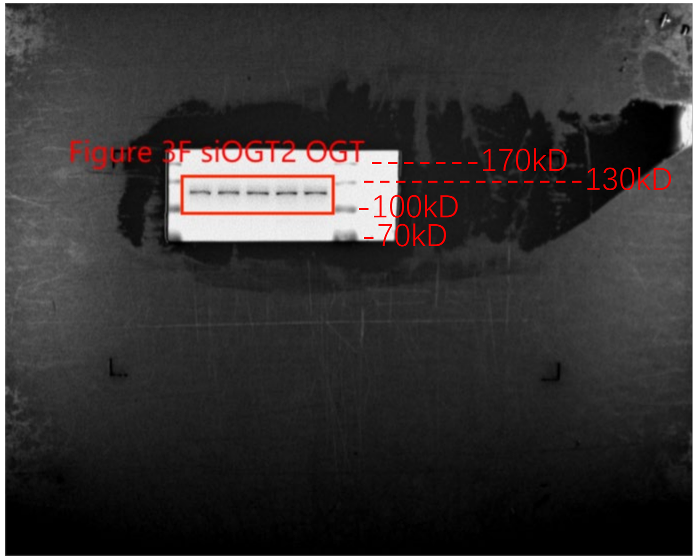

Figure 3F NC GAPDH

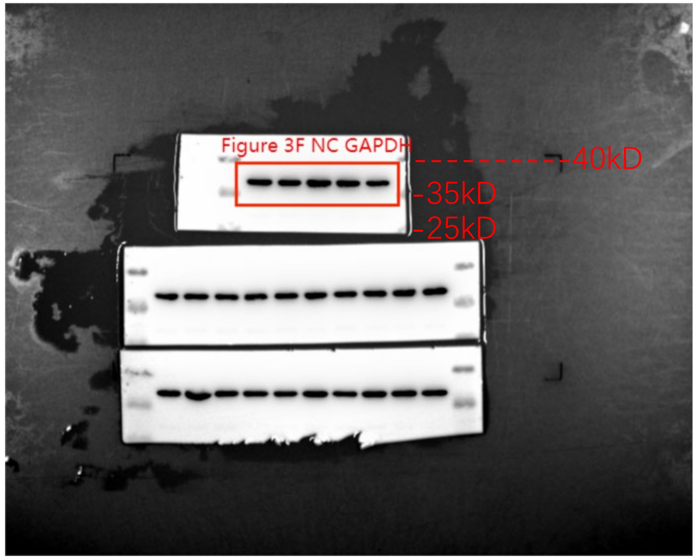

Figure 3F siOGT1 GAPDH

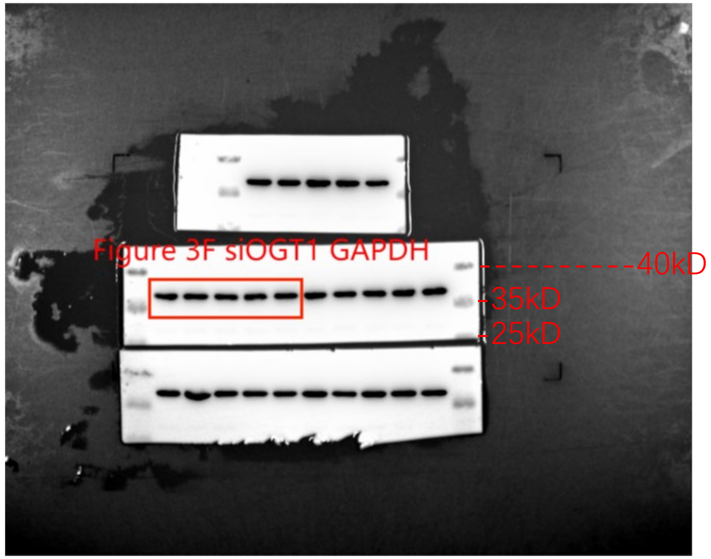

Figure 3F siOGT2 GAPDH

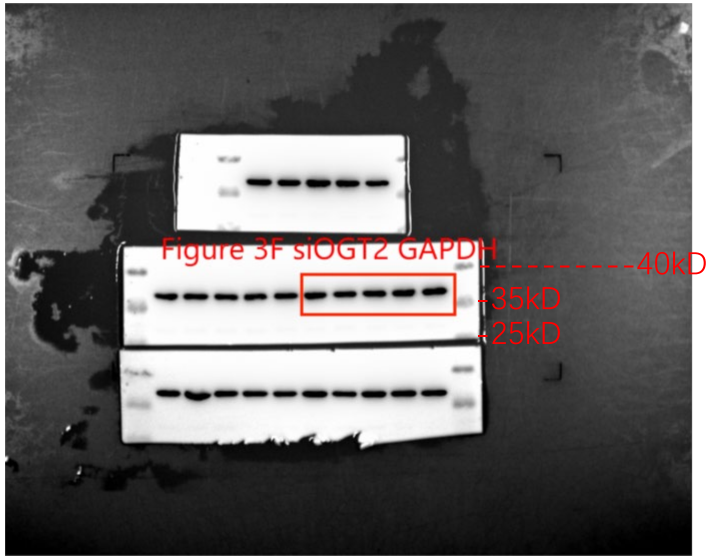

Figure 3F NC SIRT7

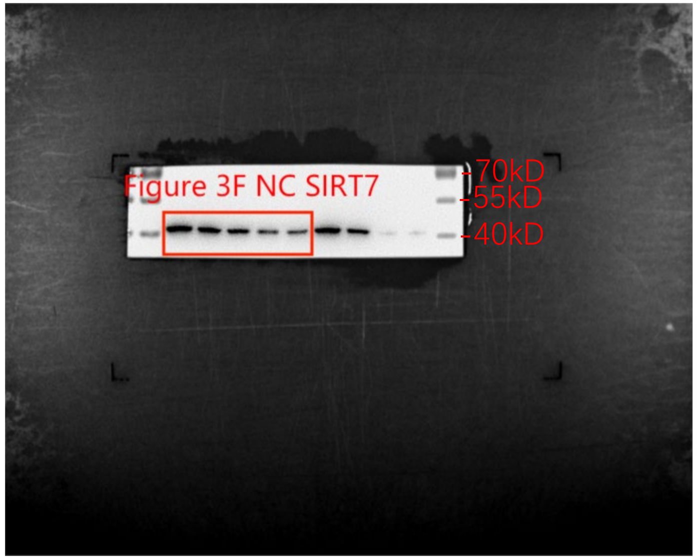

Figure 3F siOGT1 SIRT7

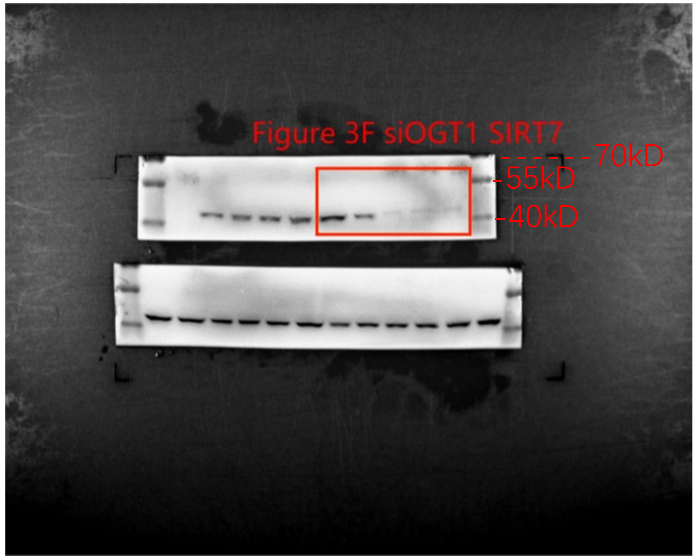

Figure 3F siOGT2 SIRT7

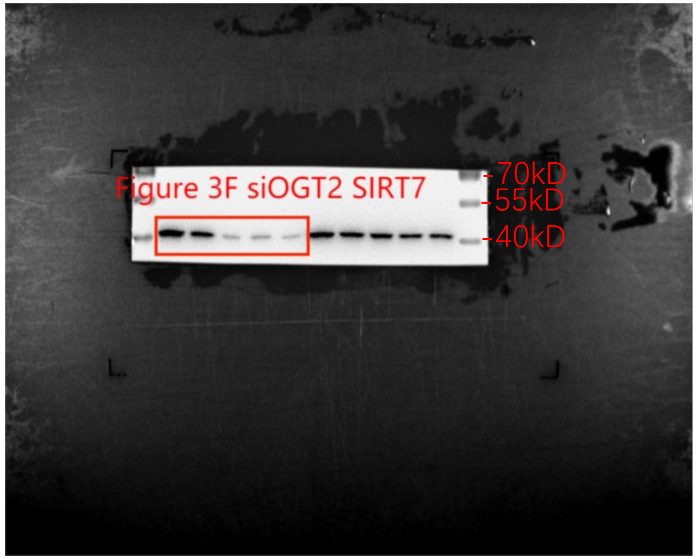

Figure 3G NC OGT

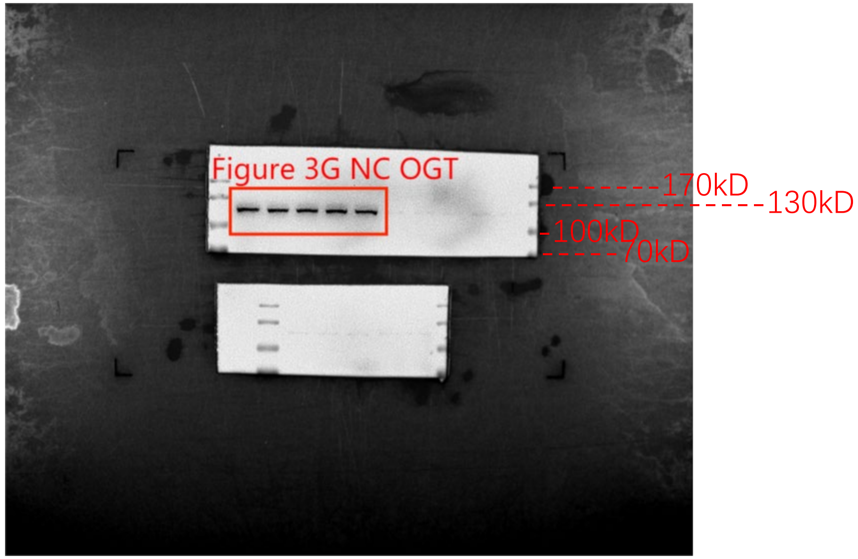

Figure 3G siOGT1 OGT

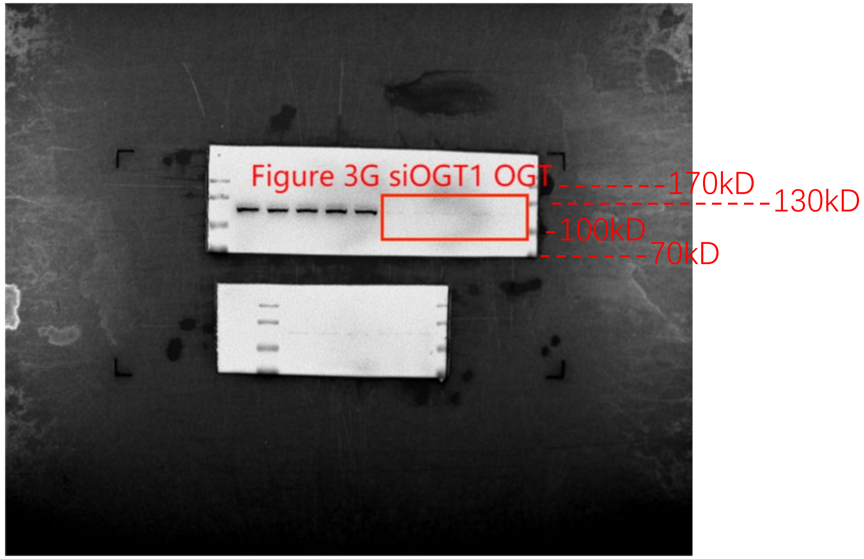

Figure 3G siOGT2 OGT

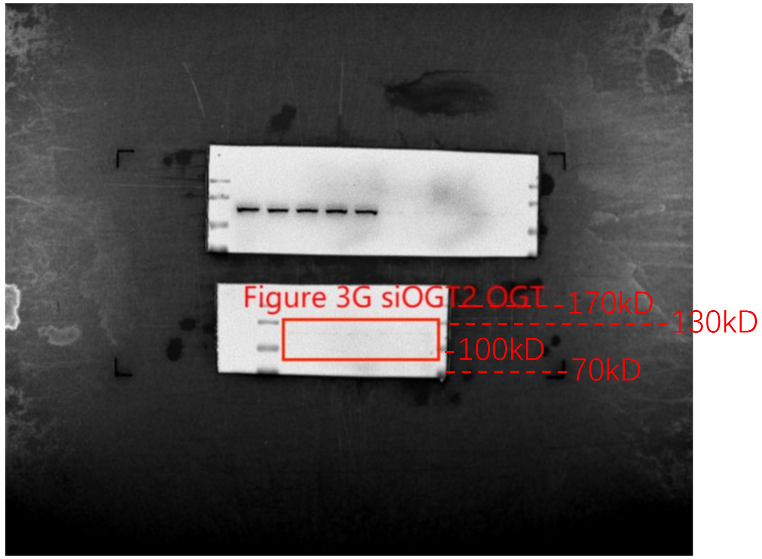

Figure 3G NC GAPDH

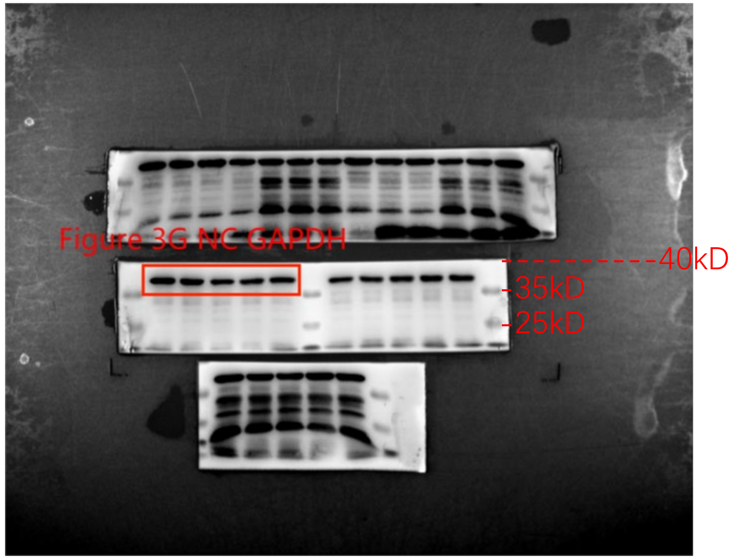

Figure 3G siOGT1 GAPDH

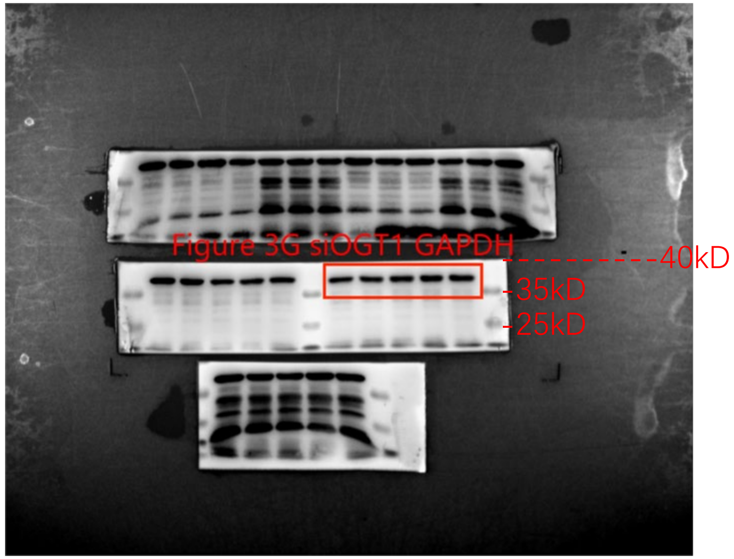

Figure 3G siOGT2 GAPDH

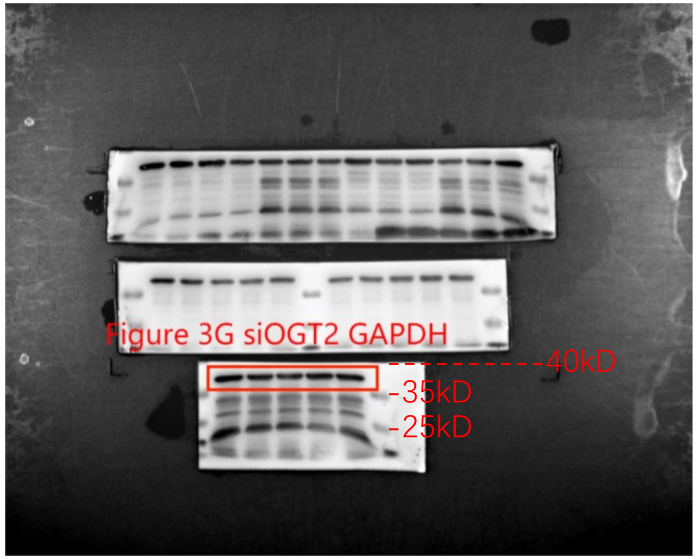

Figure 3G NC SIRT7

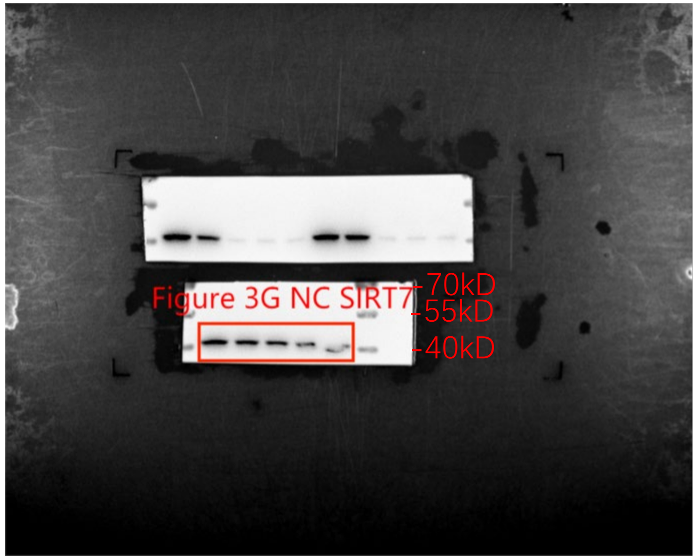

Figure 3G siOGT1 SIRT7

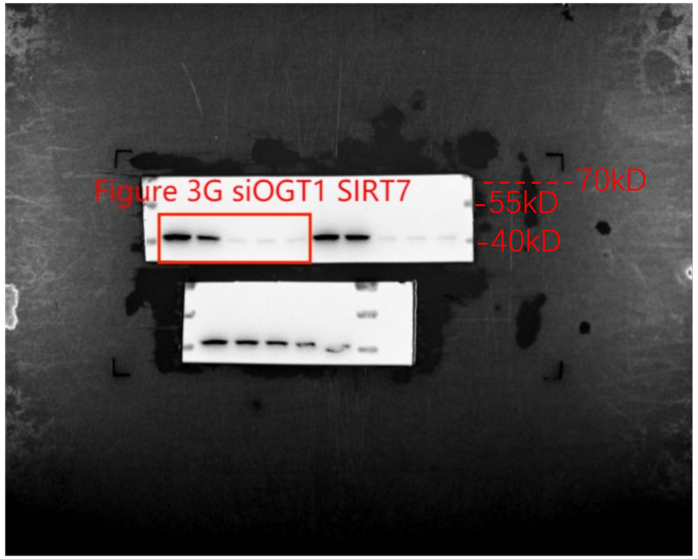

Figure 3G siOGT2 SIRT7

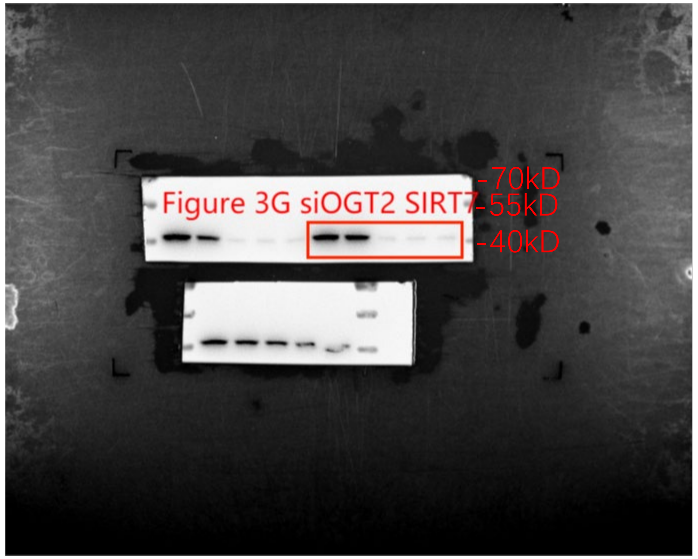

Figure 3H NC HA

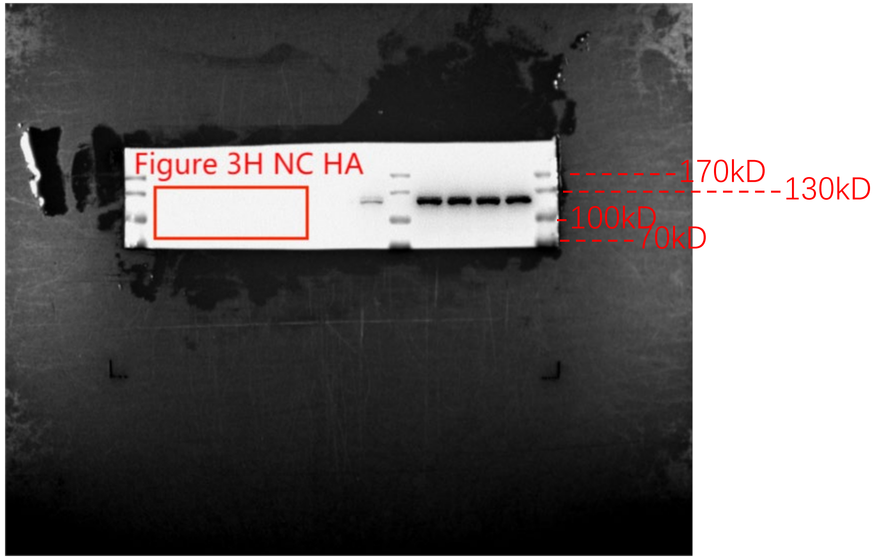

Figure 3H WT HA

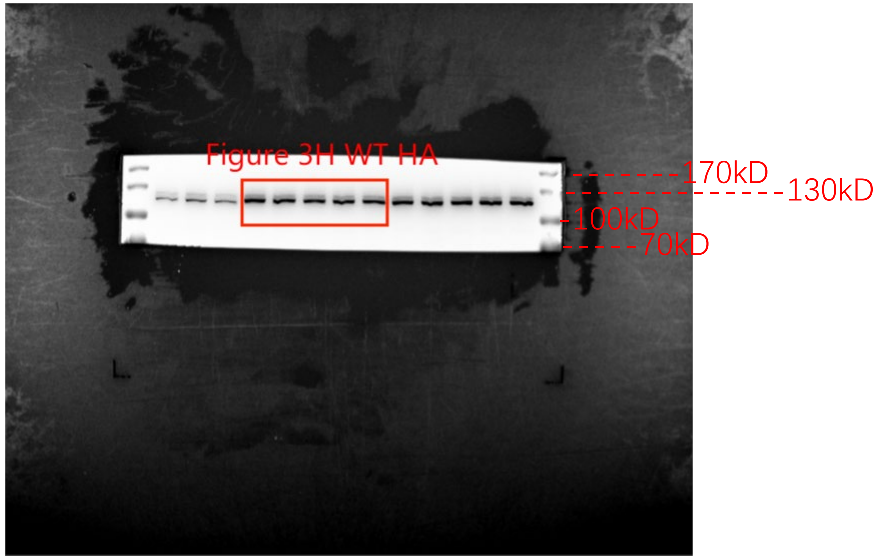

Figure 3H K908A HA

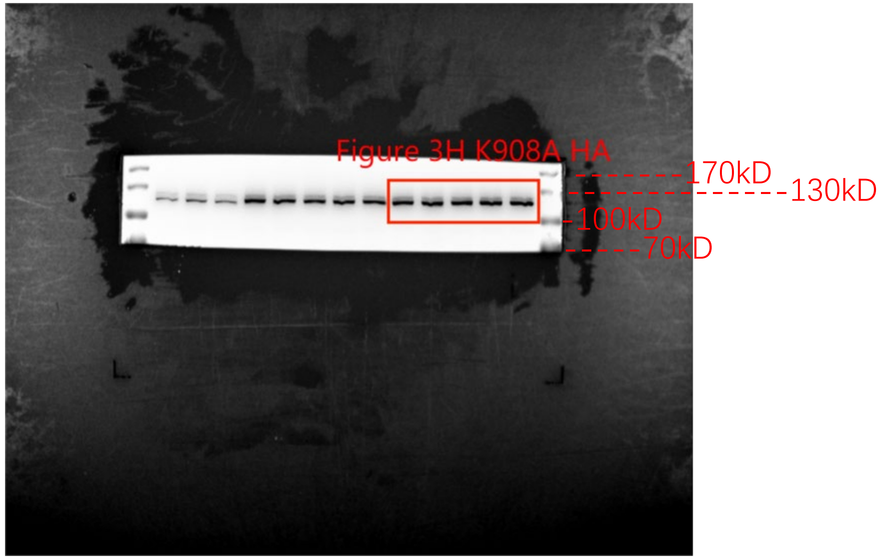

Figure 3H NC GAPDH

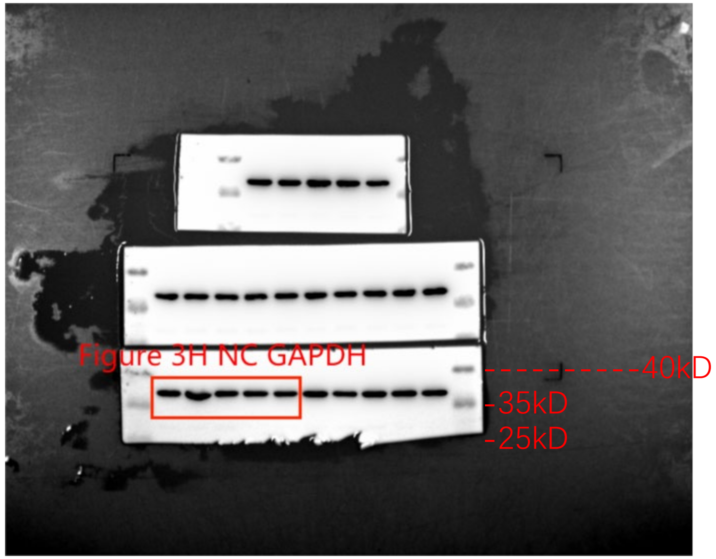

Figure 3H WT GAPDH

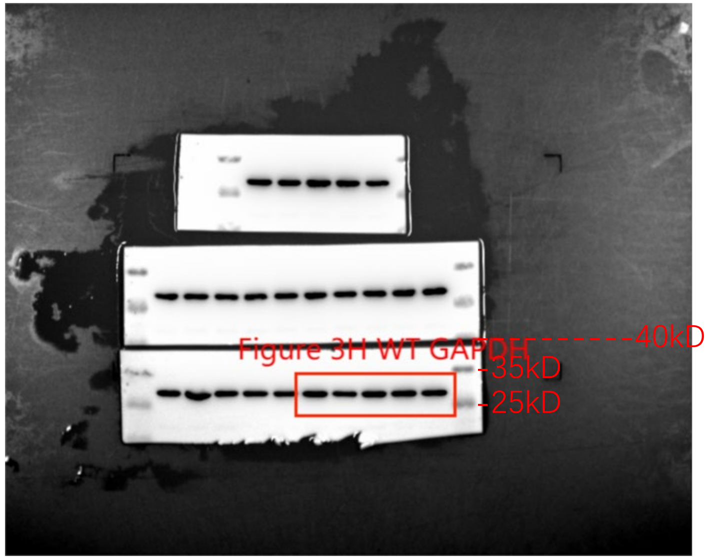

Figure 3H K908A GAPDH

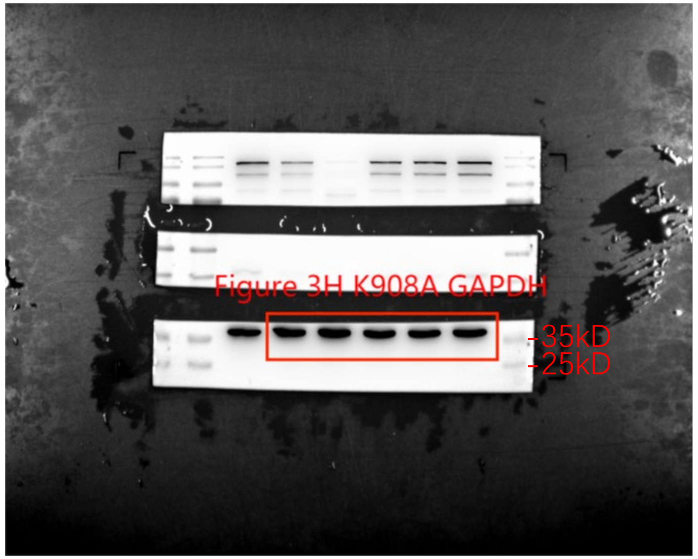

Figure 3H NC SIRT7

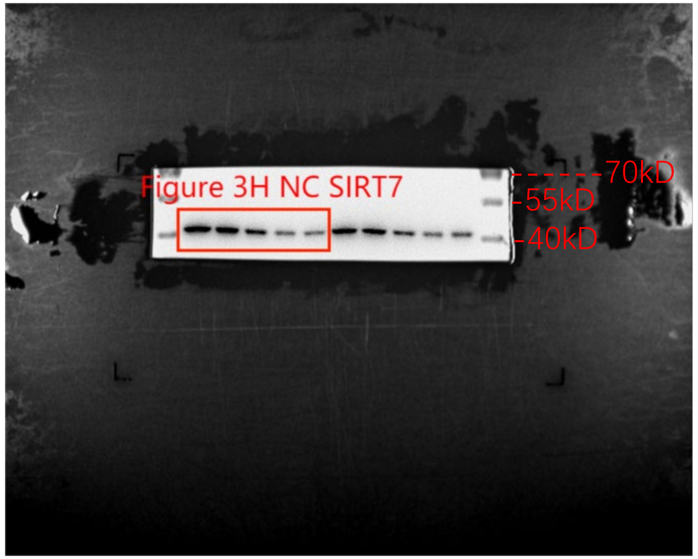

Figure 3H WT SIRT7

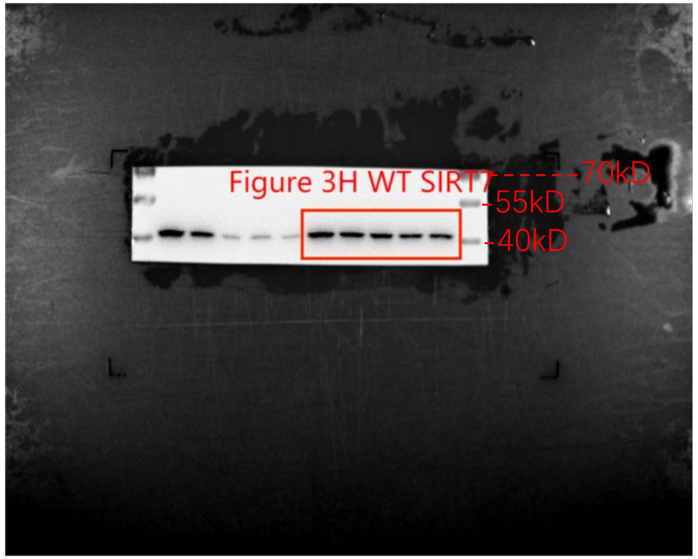

Figure 3H K908A SIRT7

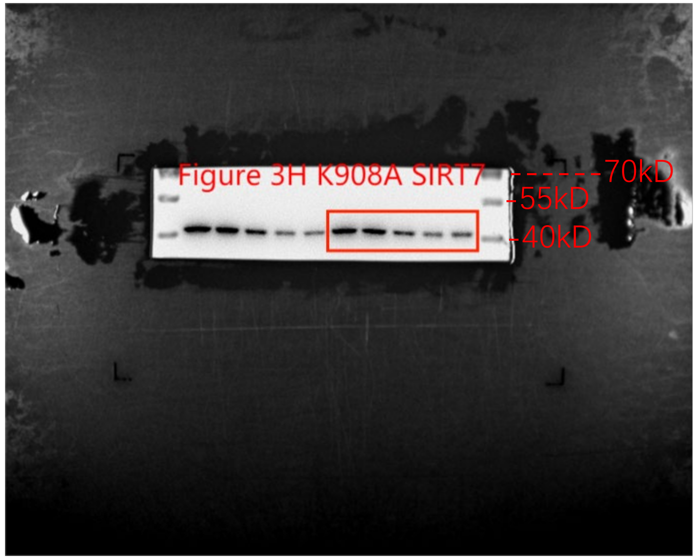

Figure 3I NC HA

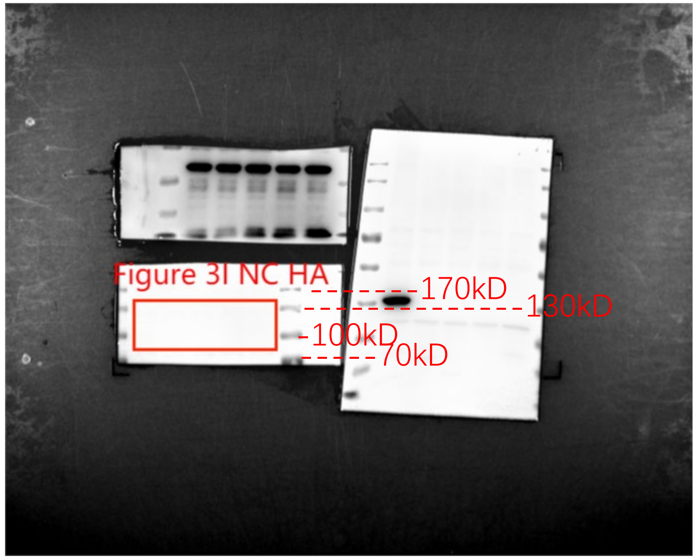

Figure 3I WT HA

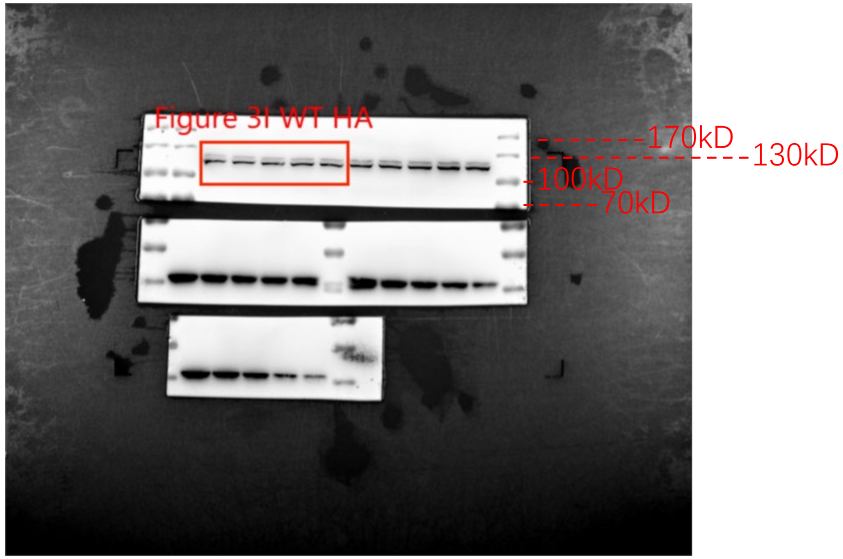

Figure 3I K908A HA

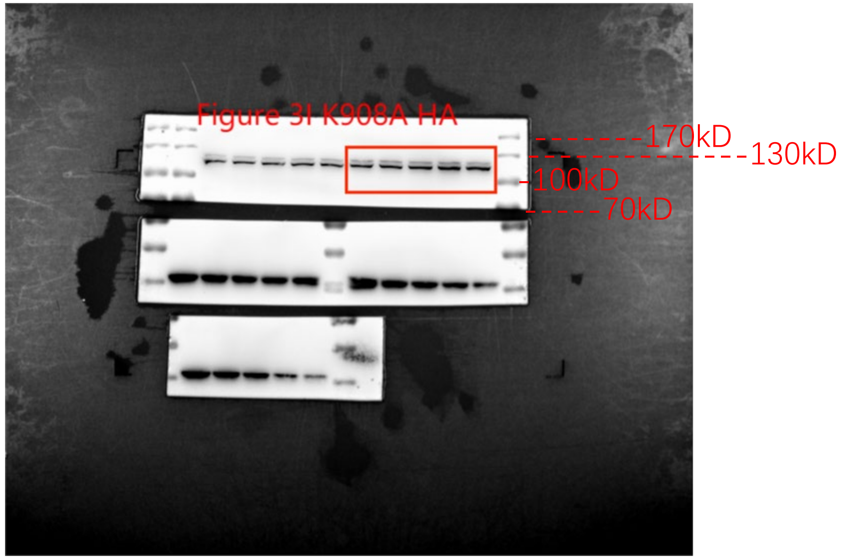

Figure 3I NC GAPDH

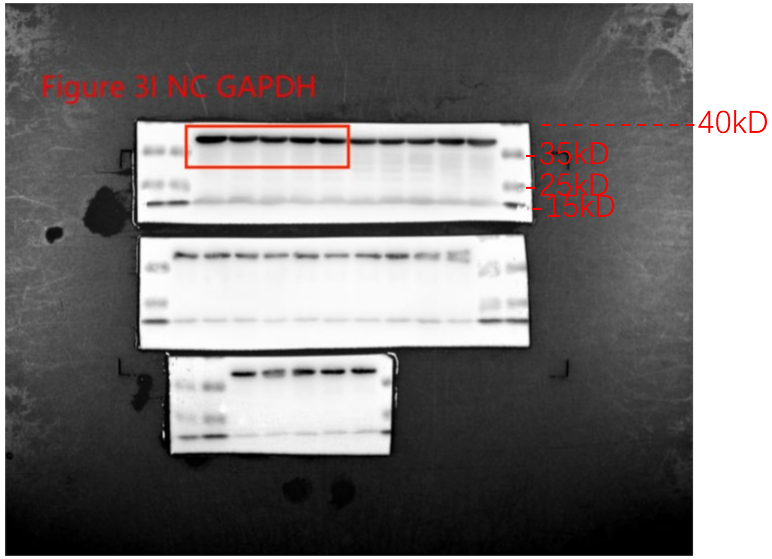

Figure 3I WT GAPDH

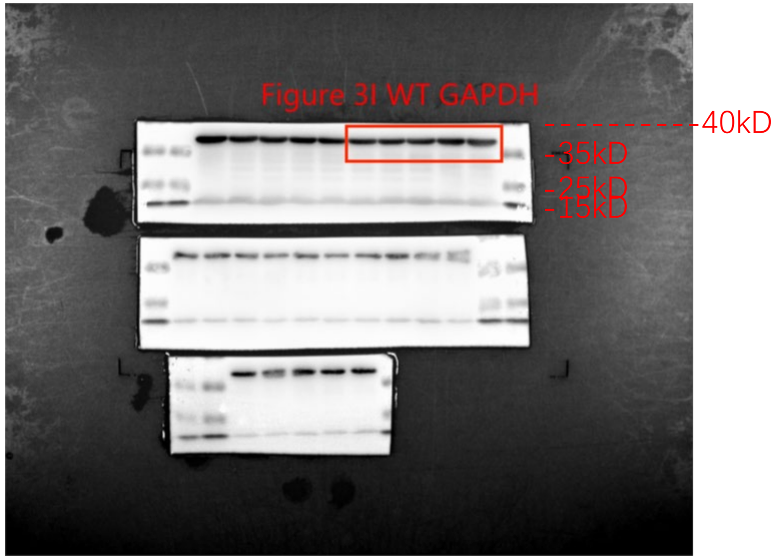

Figure 3I K908A GAPDH

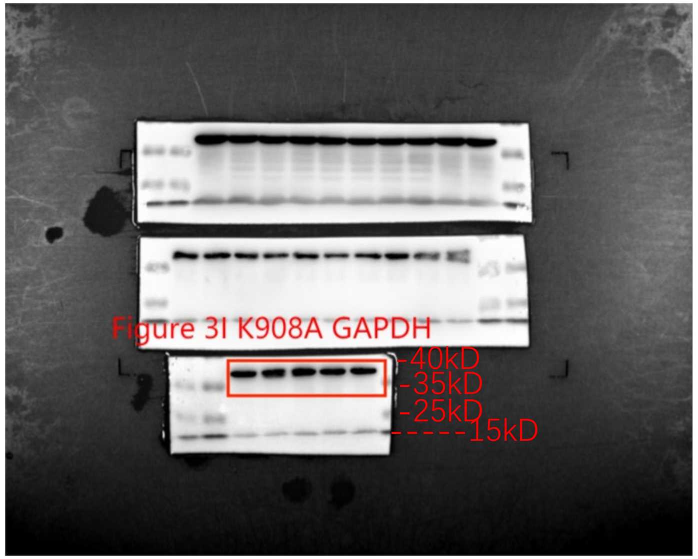

Figure 3I NC SIRT7

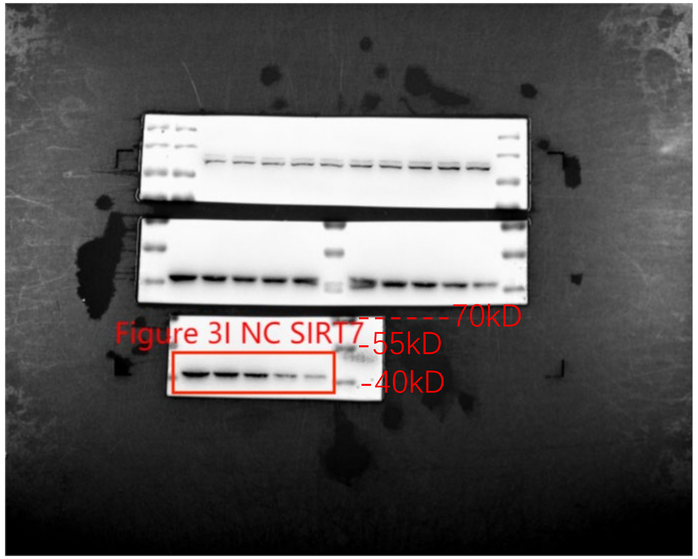

Figure 3I WT SIRT7

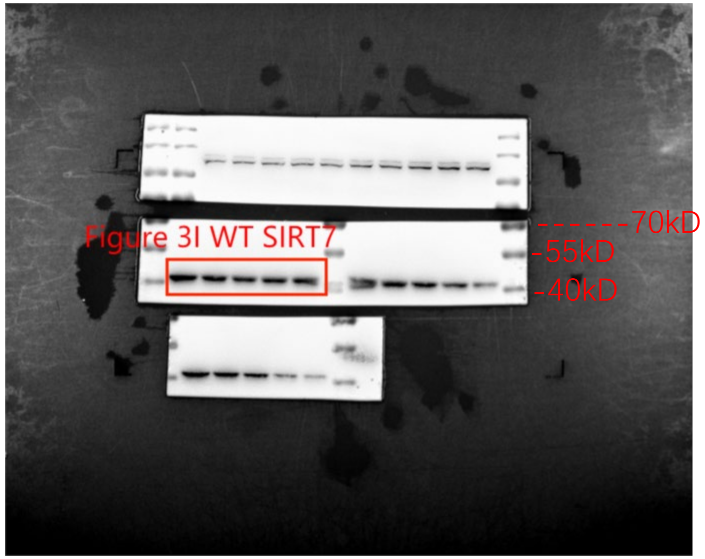

Figure 3I K908A SIRT7

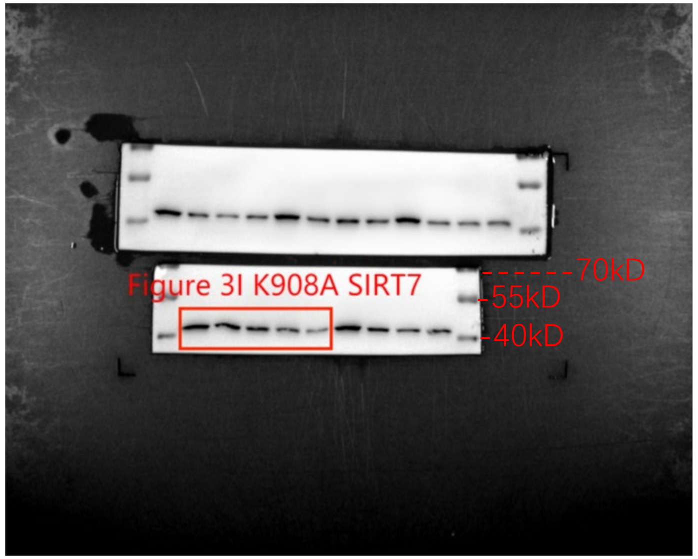

Figure 4A PANC-1 SIRT7

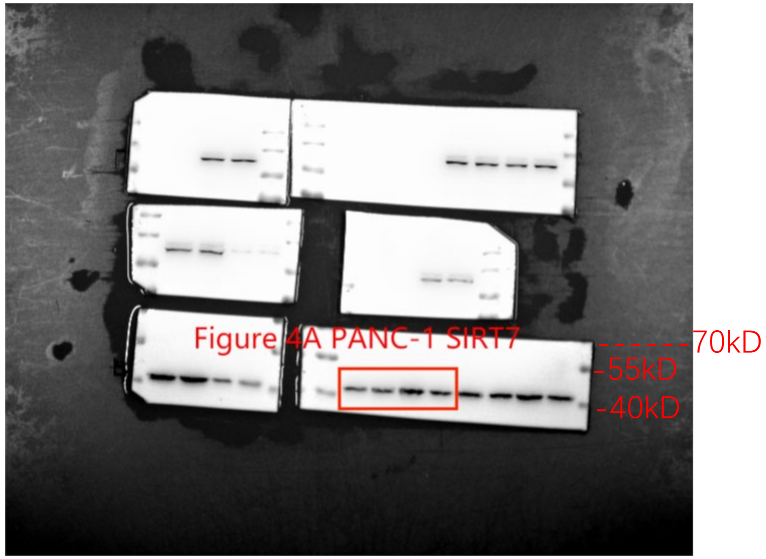

Figure 4A MiaPaCa-2 SIRT7

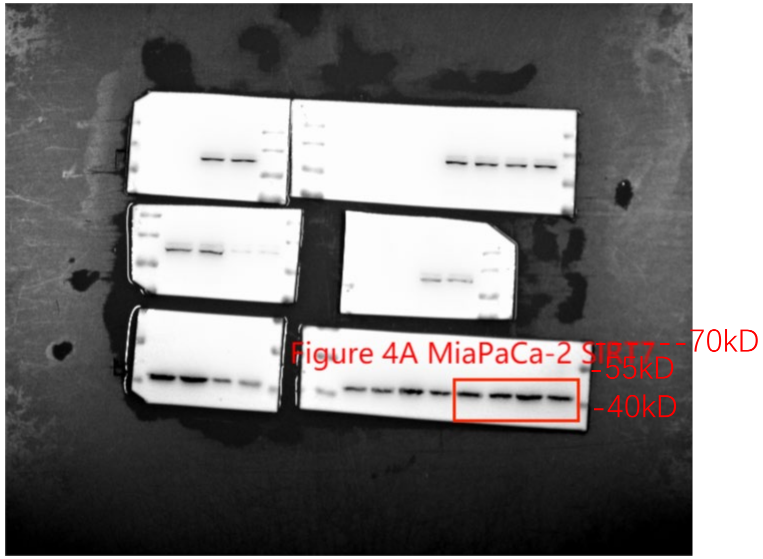

Figure 4A PANC-1 MYC

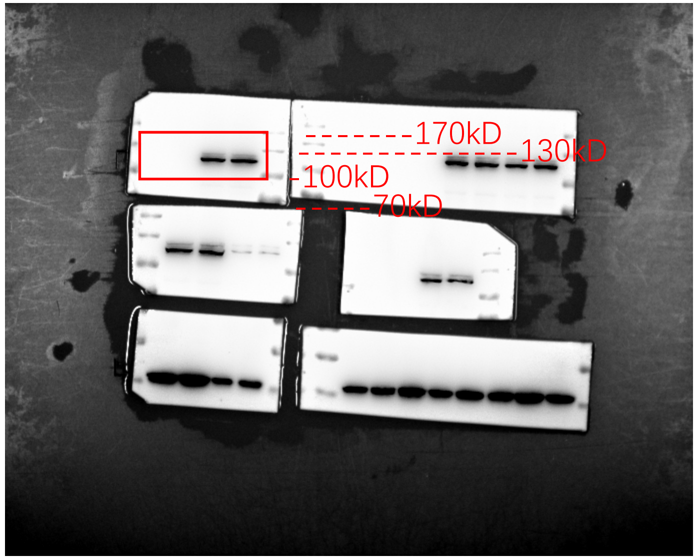

Figure 4A MiaPaCa-2 MYC

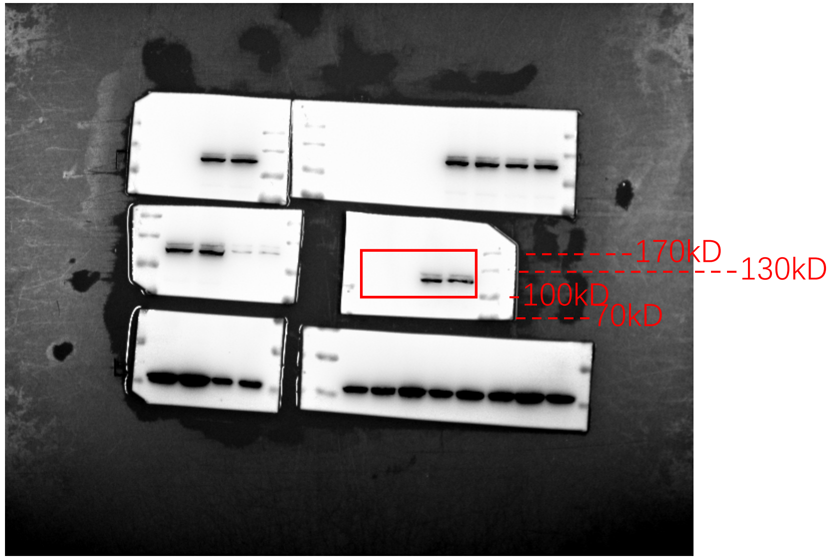

Figure 4A PANC-1 GAPDH

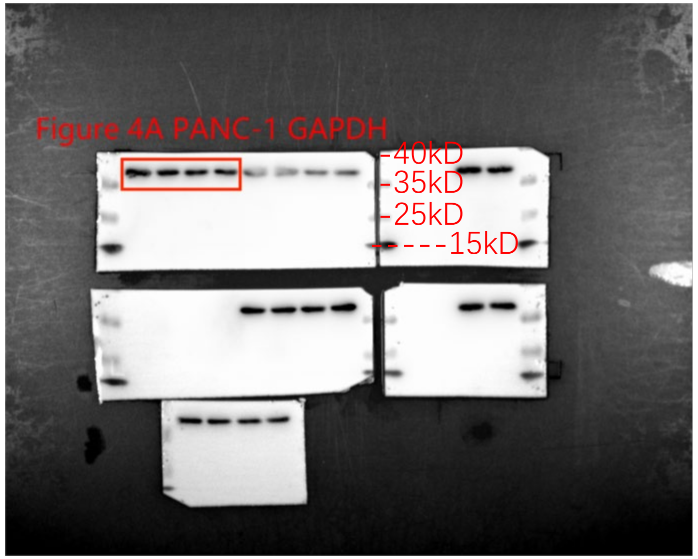

Figure 4A MiaPaCa-2 GAPDH

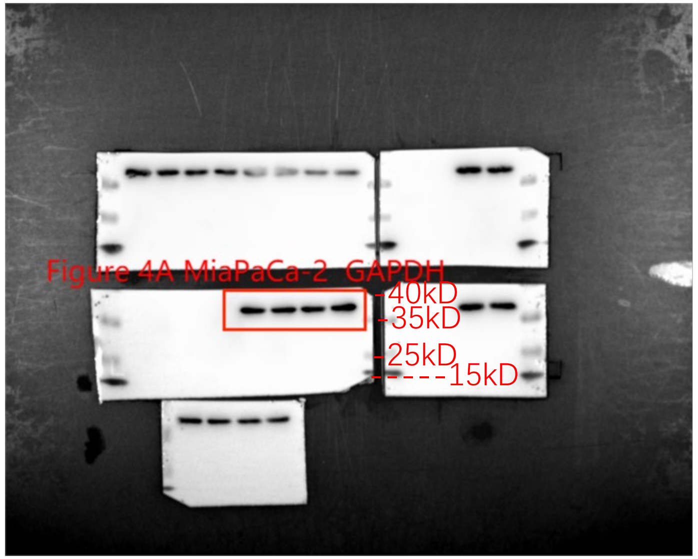

Figure 4A PANC-1 IP SIRT7 IB HA

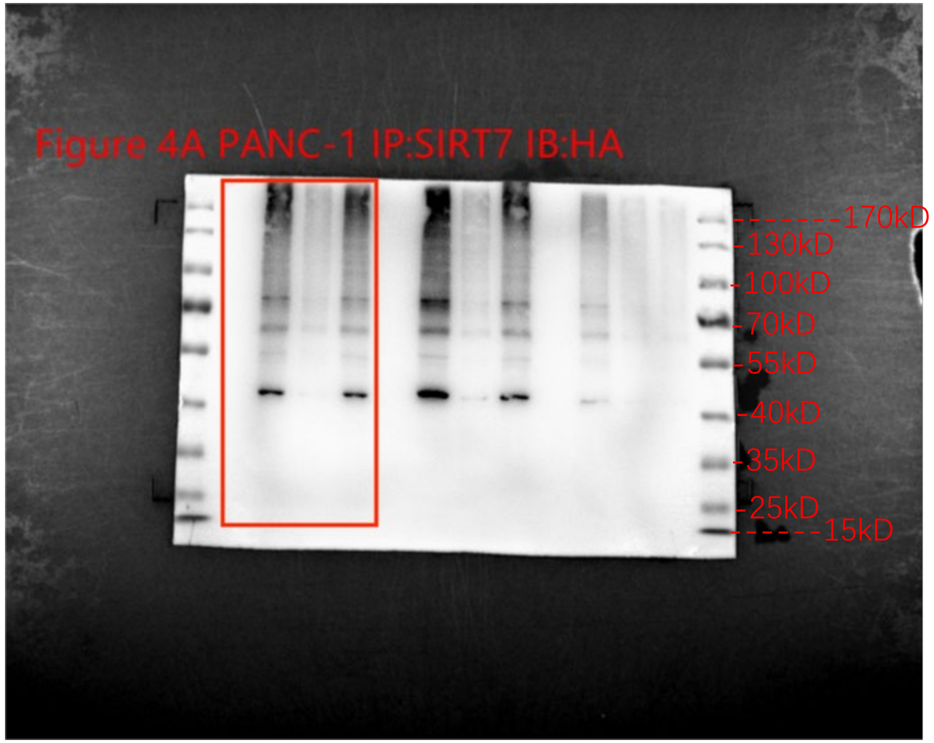

Figure 4A MiaPaCa-2 IP SIRT7 IB HA

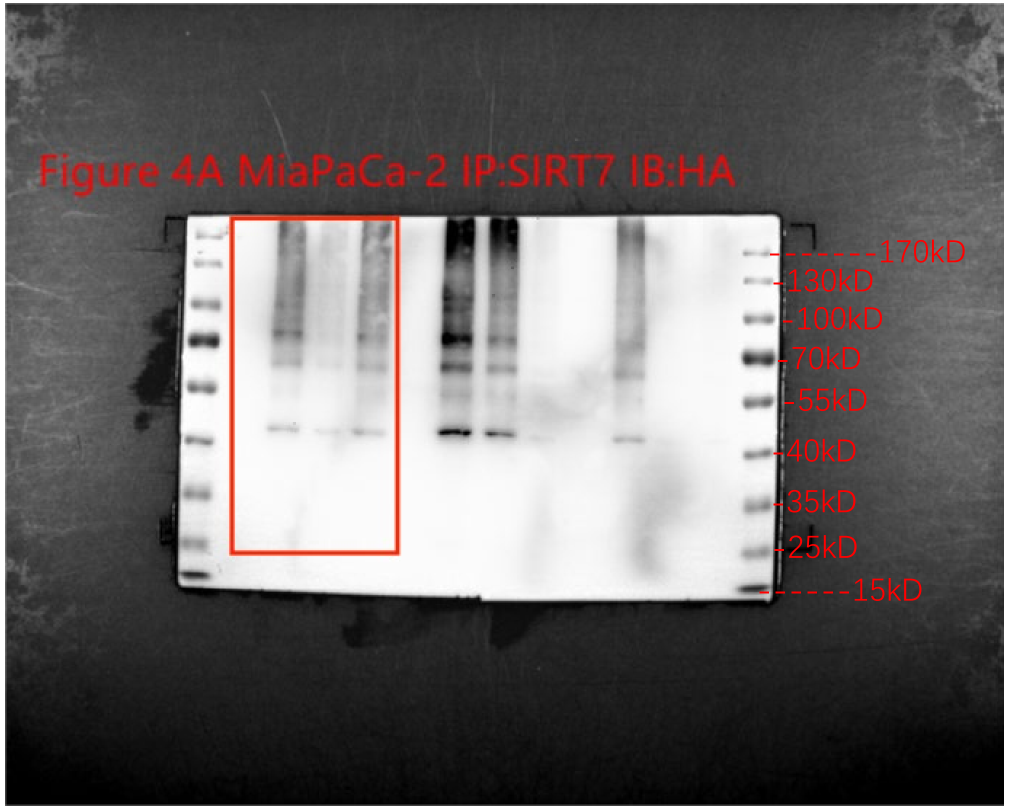

Figure 4B PANC-1 SIRT7

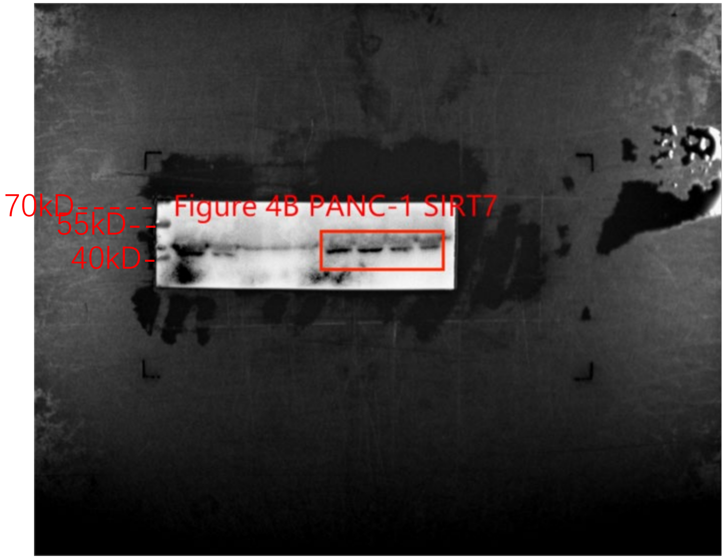

Figure 4B MiaPaCa-2 SIRT7

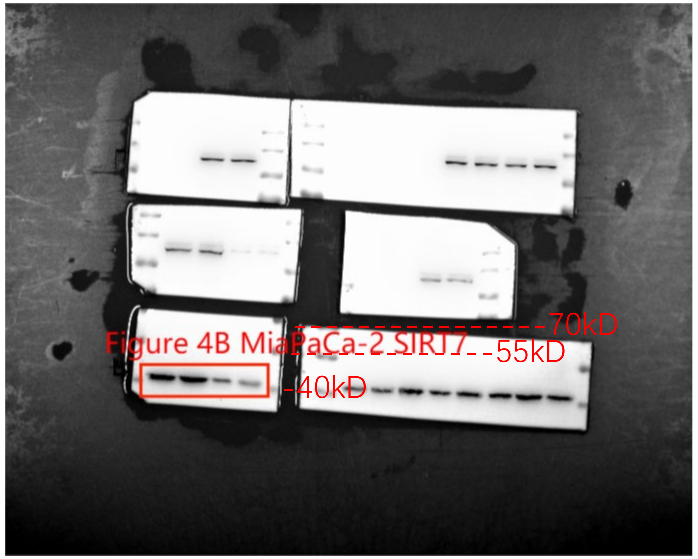

Figure 4B PANC-1 OGT

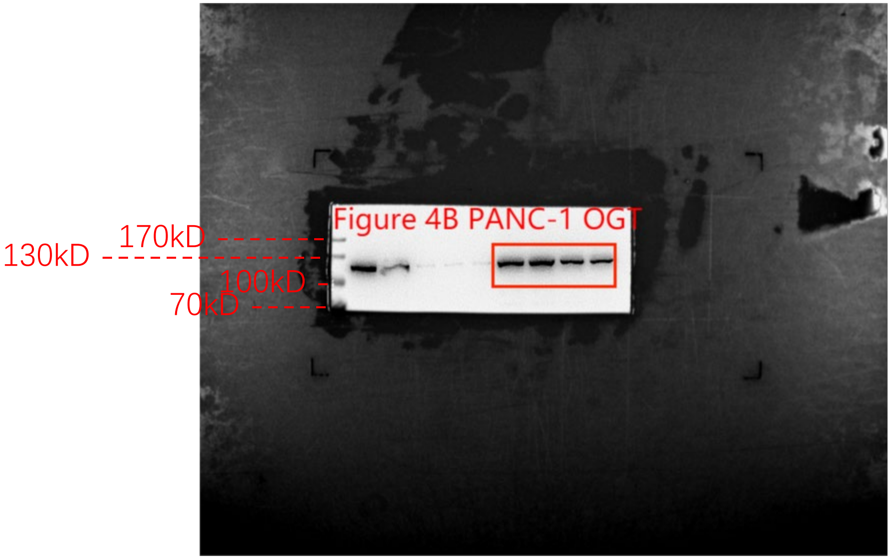

Figure 4B MiaPaCa-2 OGT

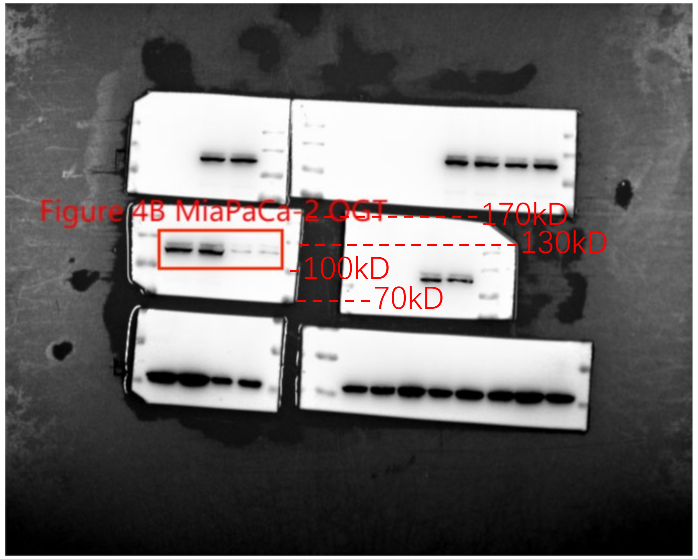

Figure 4B PANC-1 GAPDH

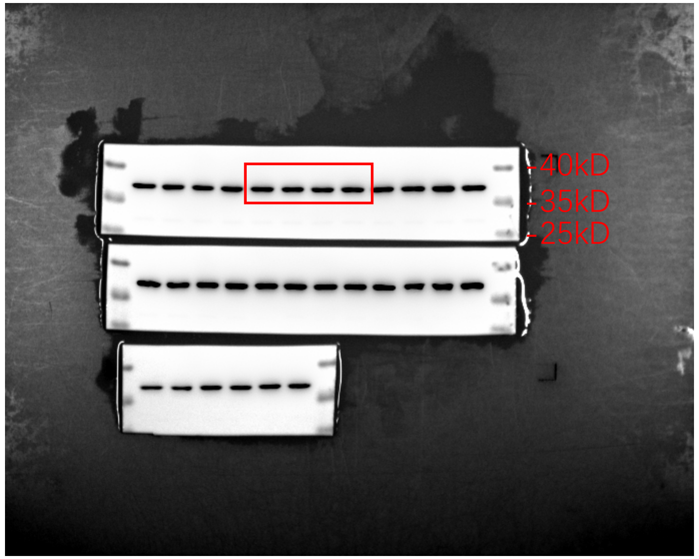

Figure 4B MiaPaCa-2 GAPDH

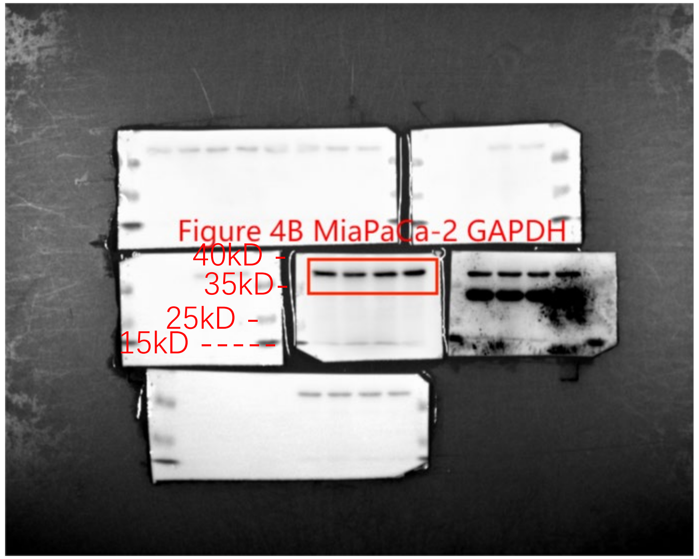

Figure 4B PANC-1 IP SIRT7 IB HA

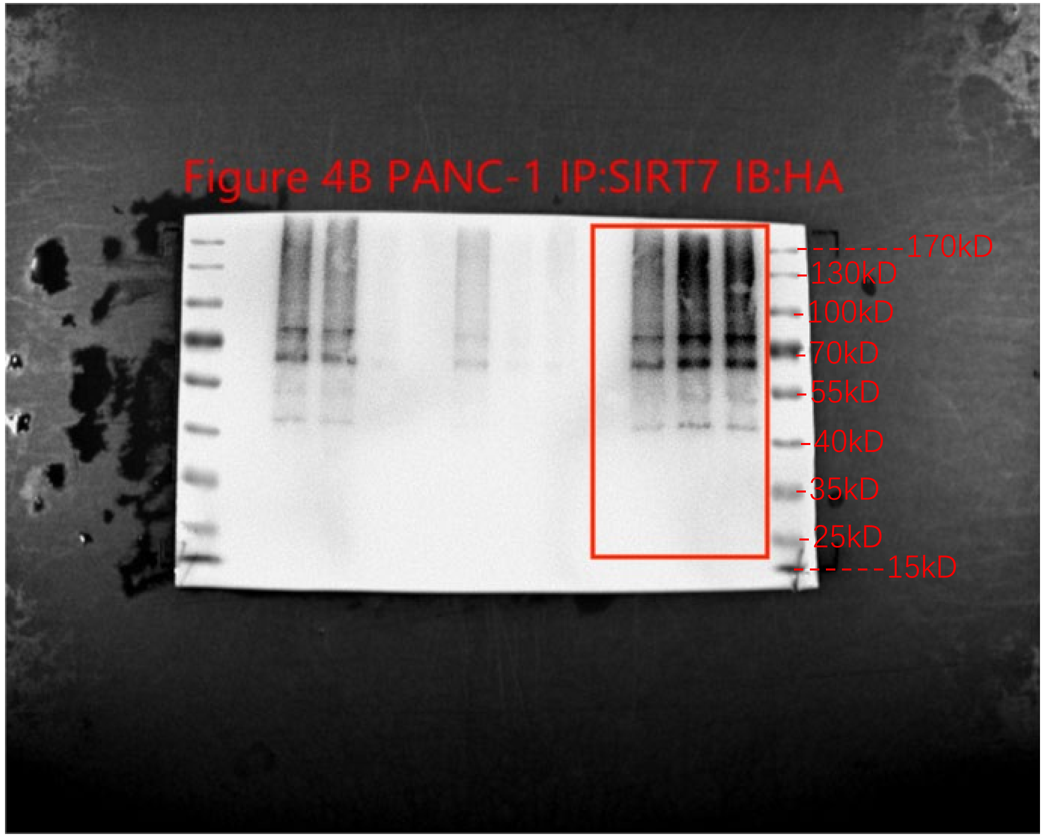

Figure 4B MiaPaCa-2 IP SIRT7 IB HA

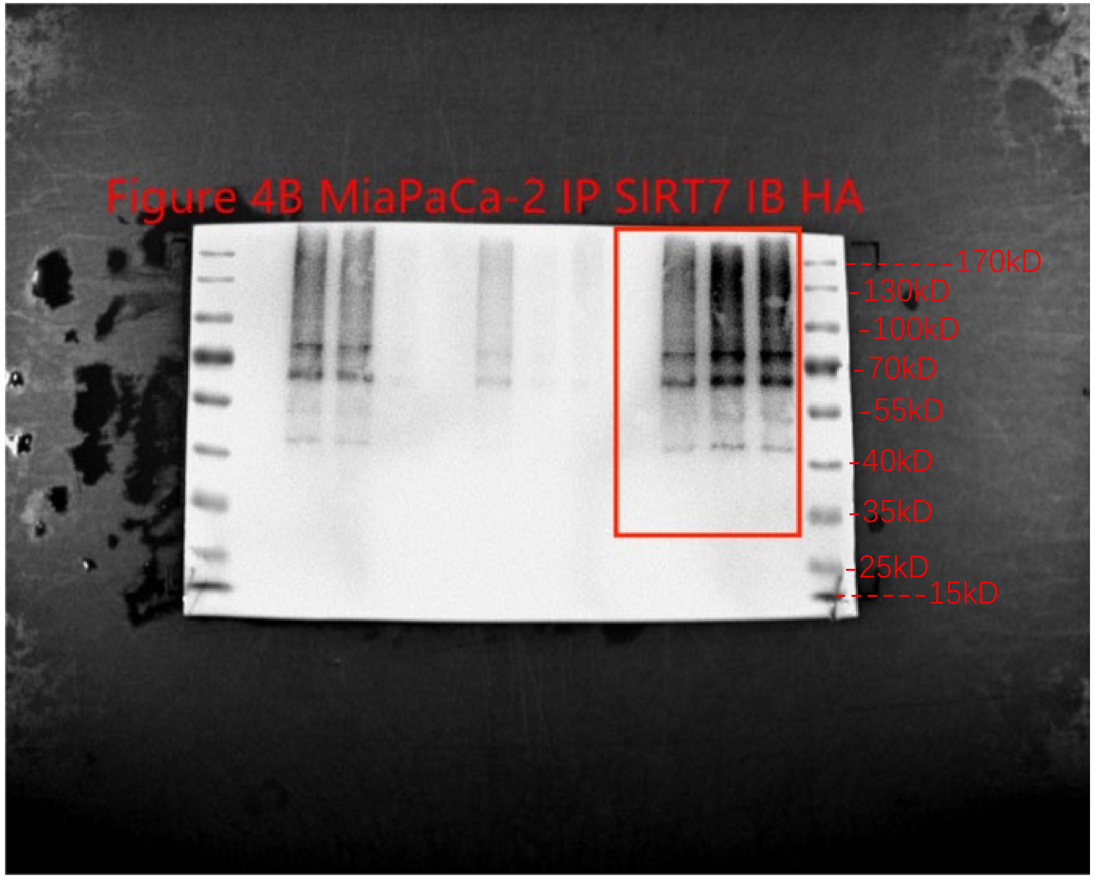

Figure 4C PANC-1 SIRT7

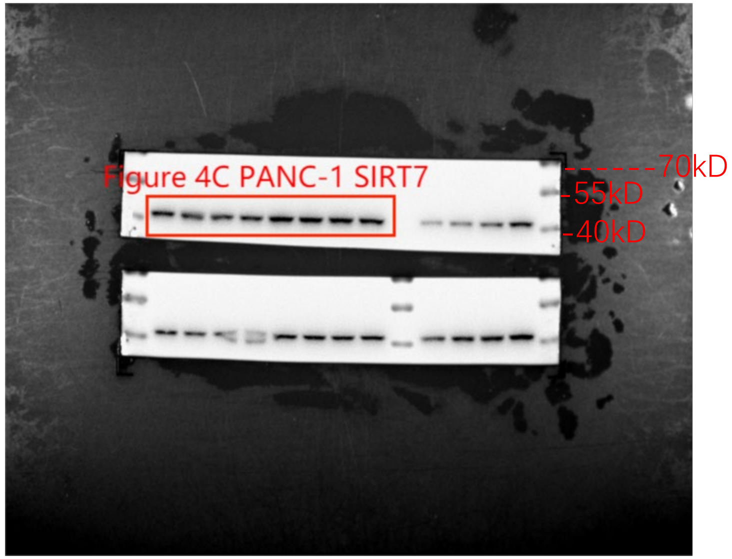

Figure 4C MiaPaCa-2 SIRT7

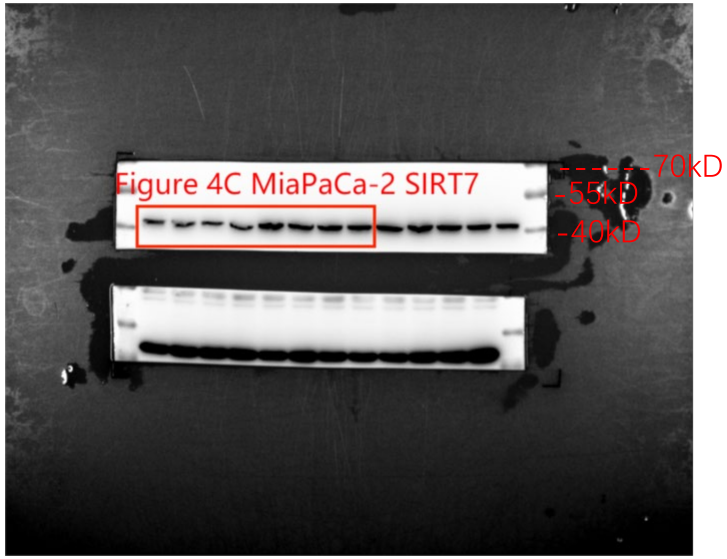

Figure 4C PANC-1 MYC

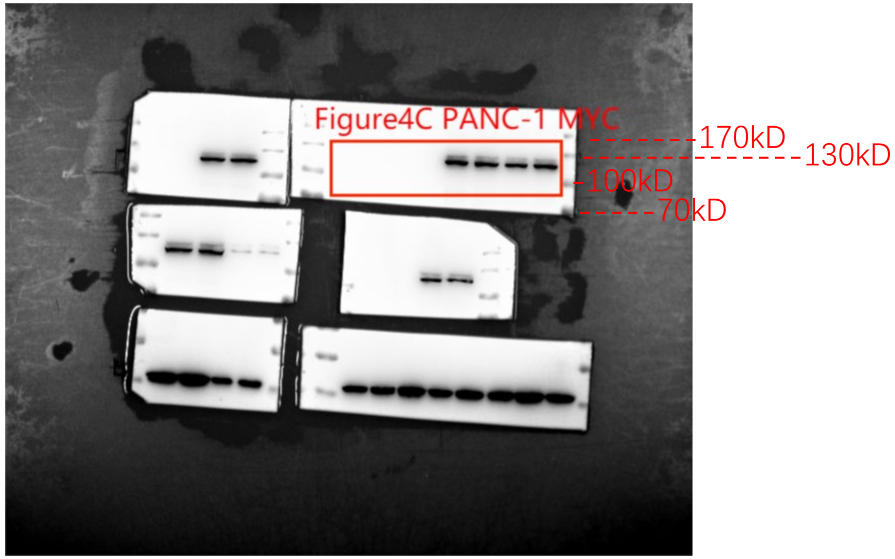

Figure 4C MiaPaCa-2 MYC

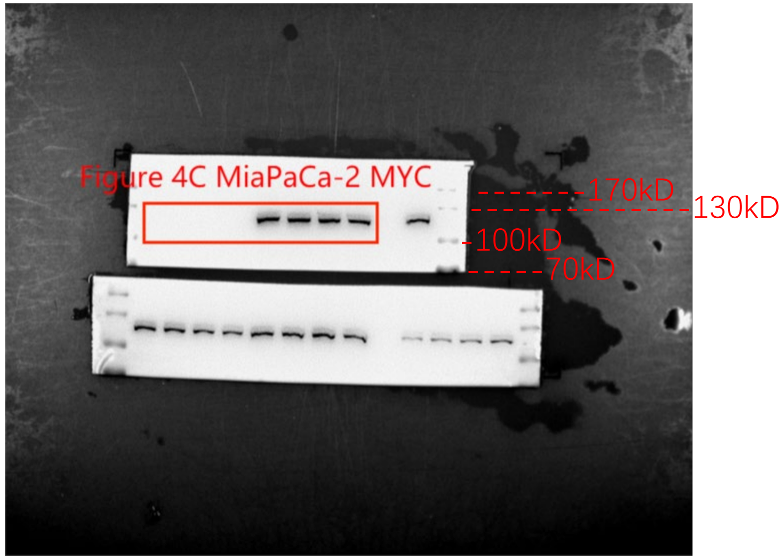

Figure 4C PANC-1 GAPDH

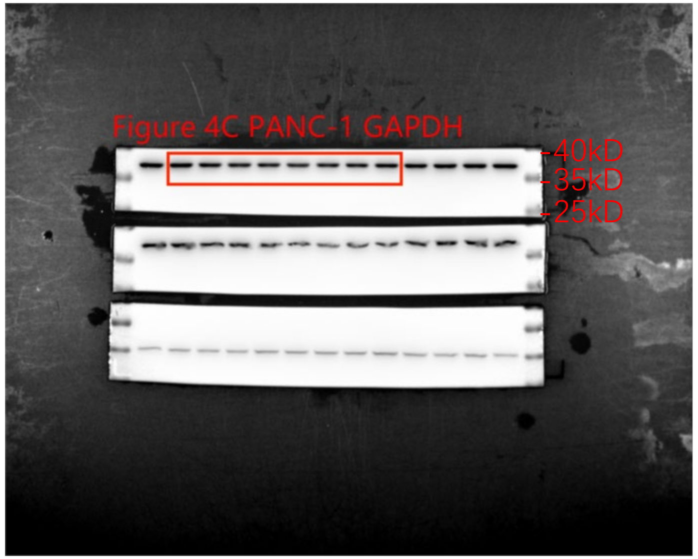

Figure 4C MiaPaCa-2 GAPDH

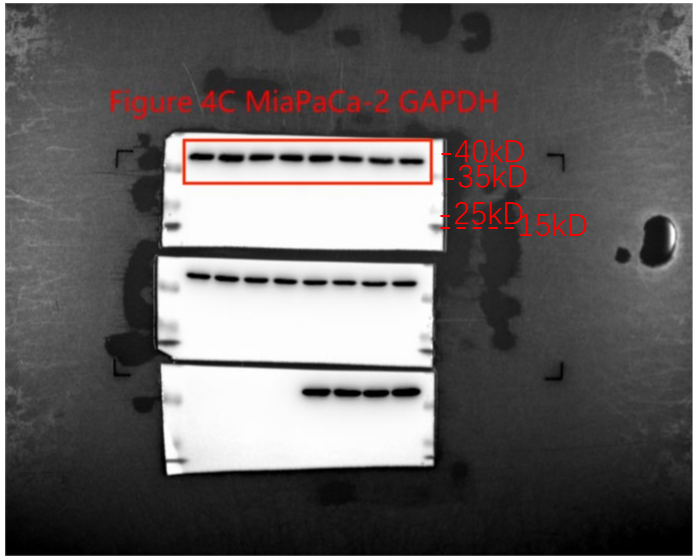

Figure 4C PANC-1 IP SIRT7 IB HA

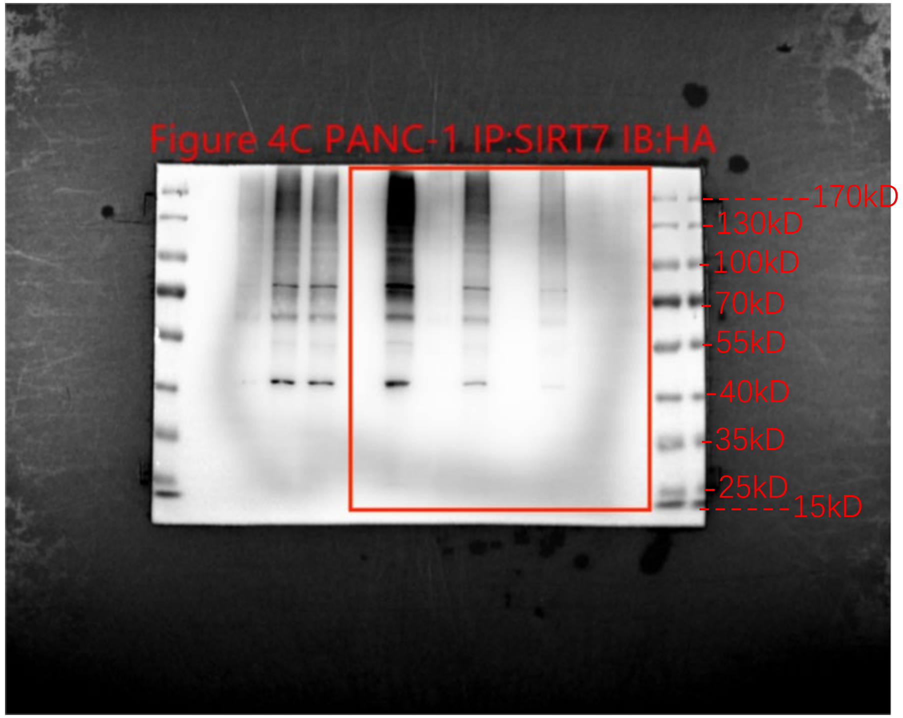

Figure 4C MiaPaCa-2 IP SIRT7 IB HA

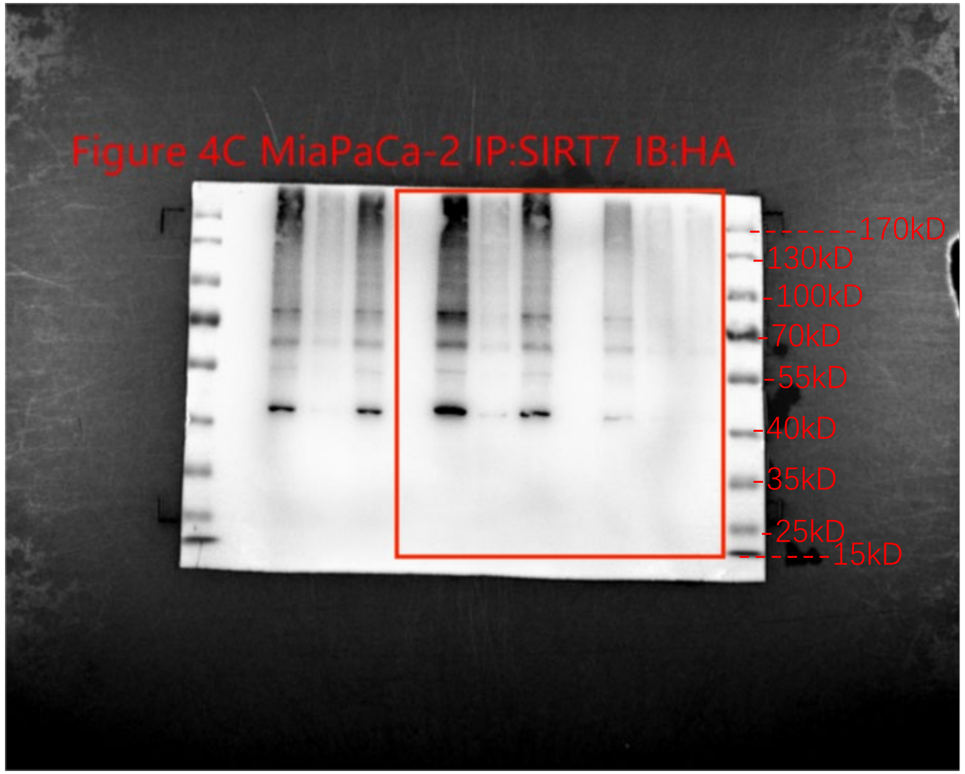

Figure 4D PANC-1 SIRT7

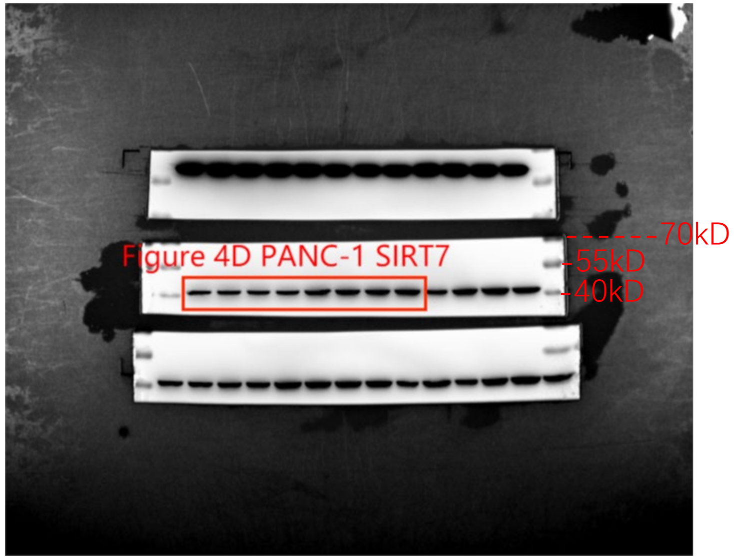

Figure 4D MiaPaCa-2 SIRT7

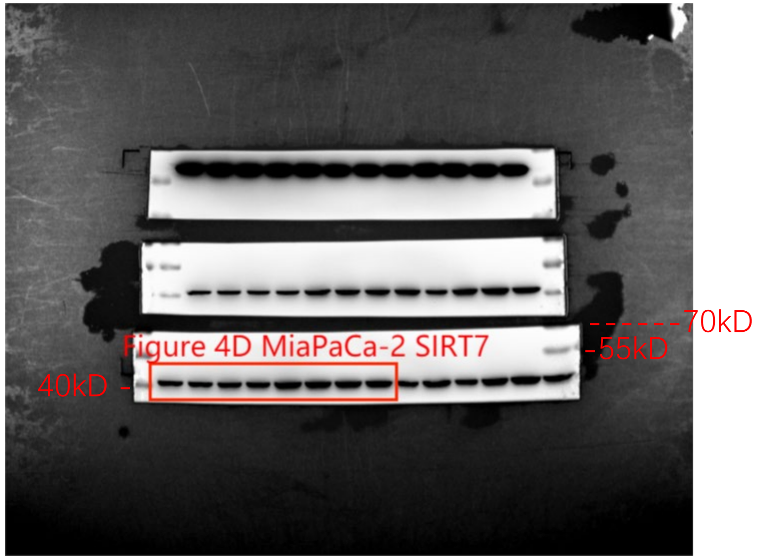

Figure 4D PANC-1 MYC

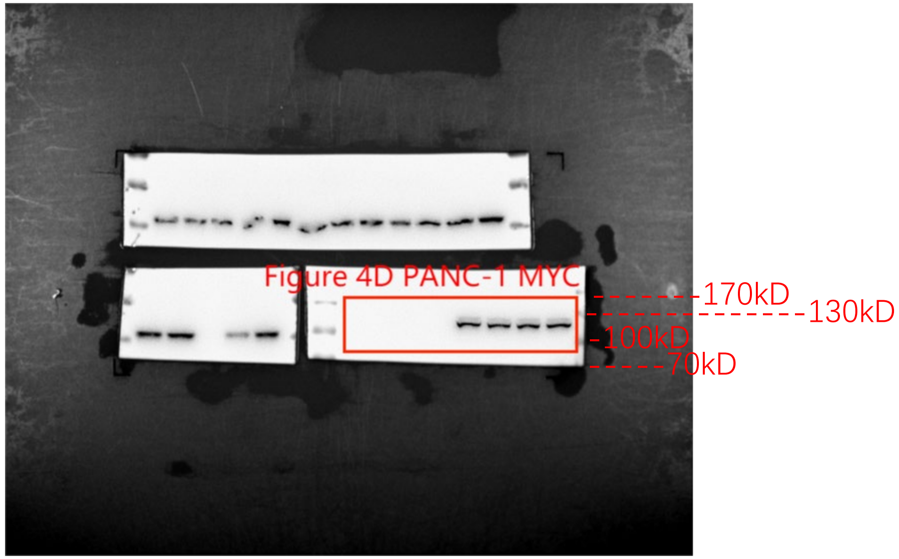

Figure 4D MiaPaCa-2 MYC

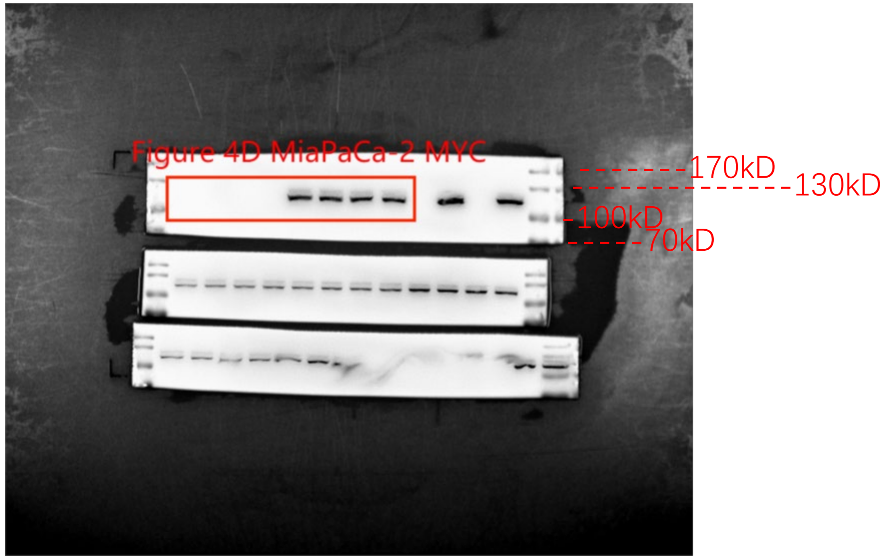

Figure 4D PANC-1 GAPDH

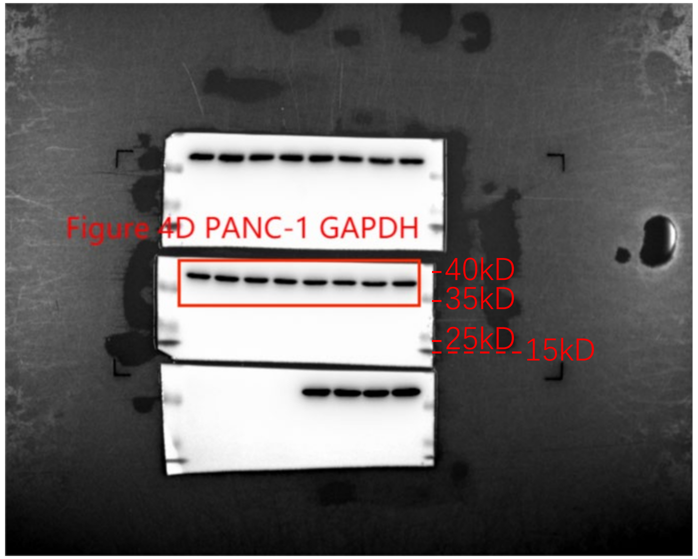

Figure 4D MiaPaCa-2 GAPDH

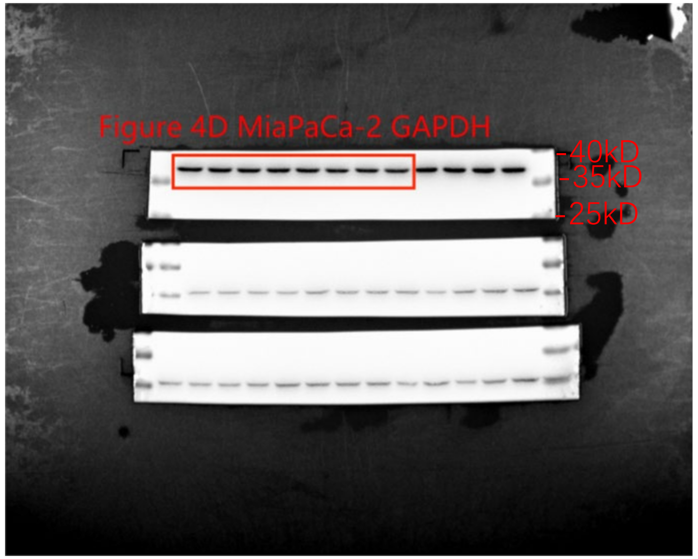

Figure 4D PANC-1 IP SIRT7 IB HA

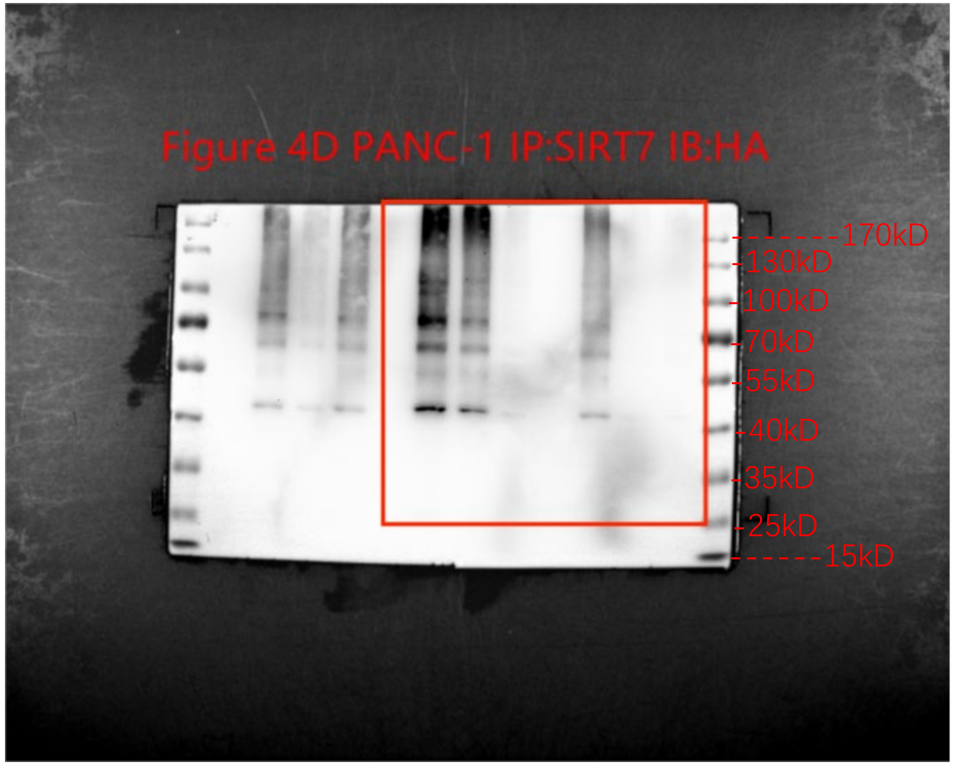

Figure 4D MiaPaCa-2 IP SIRT7 IB HA

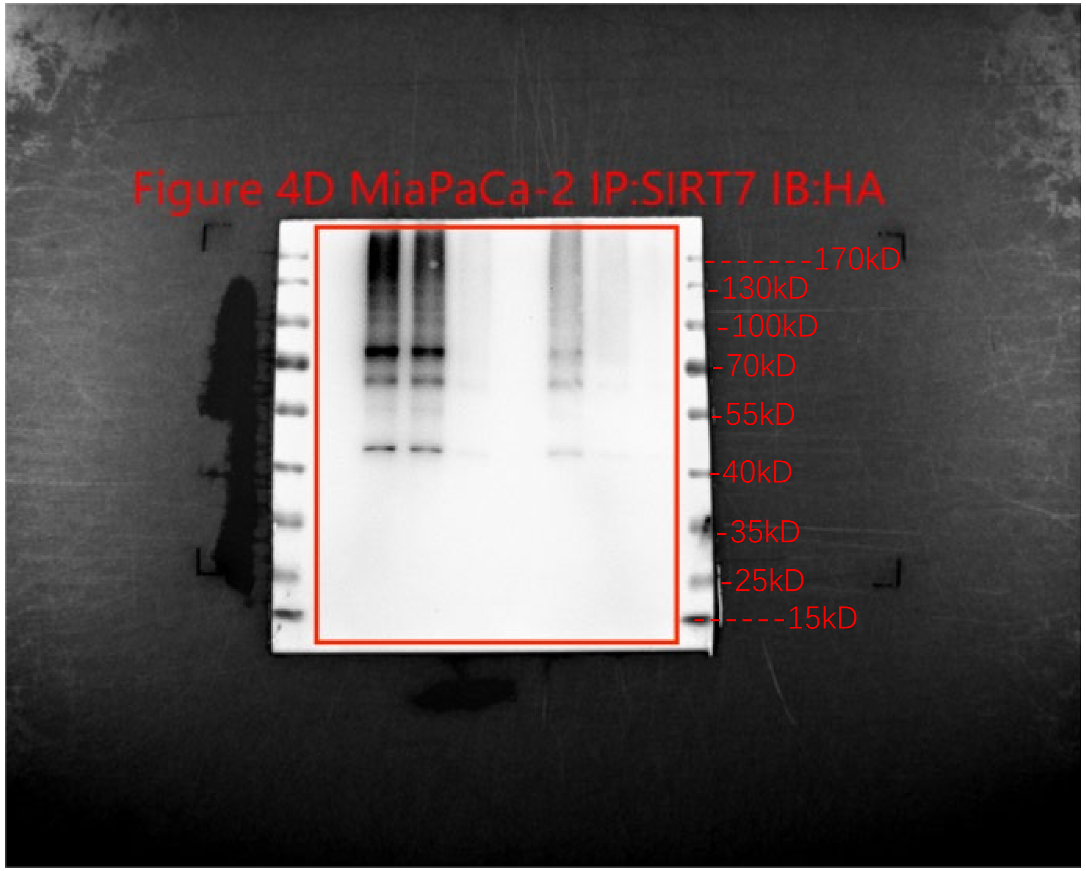

Figure 4E PANC-1 HA

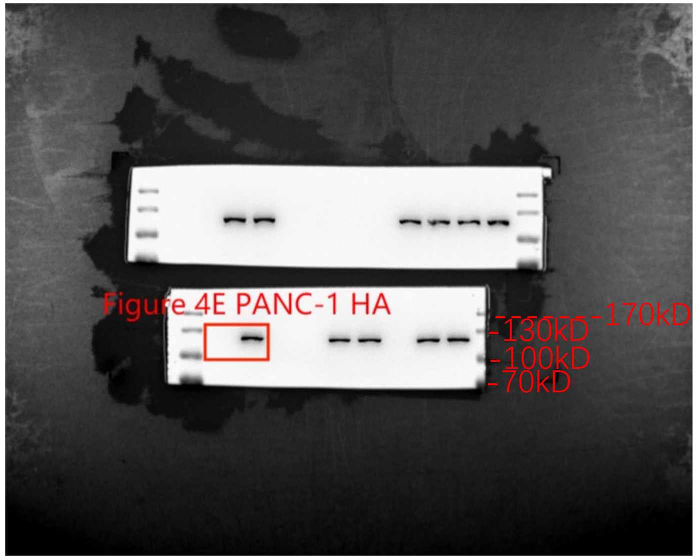

Figure 4E PANC-1 SIRT7

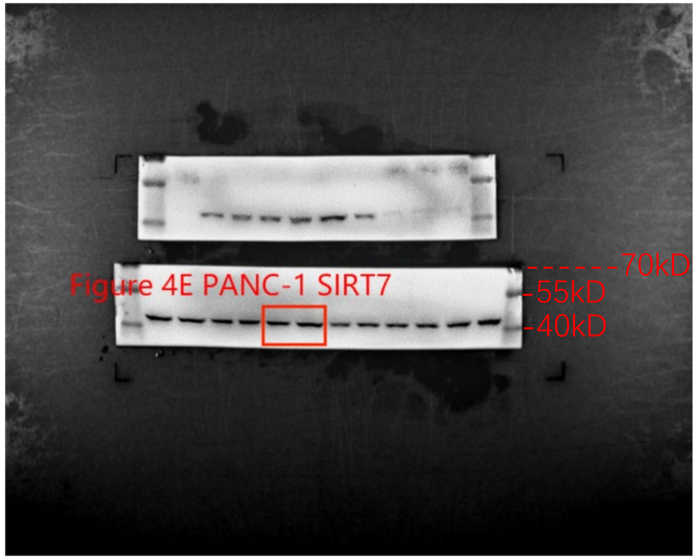

Figure 4E PANC-1 GAPDH

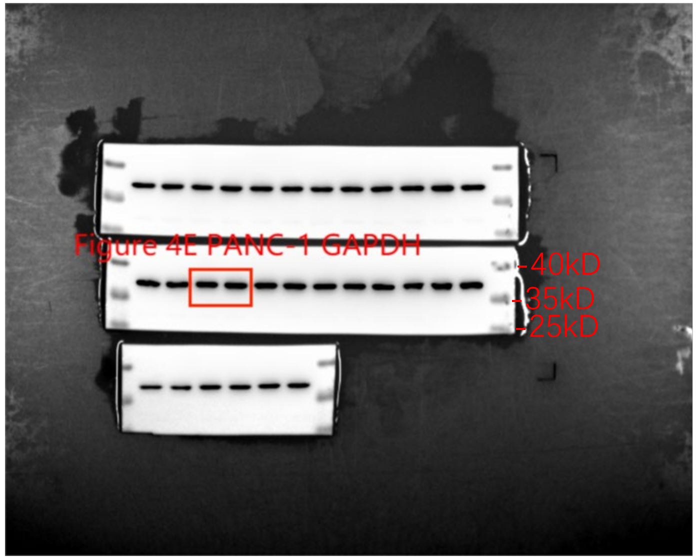

Figure 4E PANC-1 IP SIRT7 IB SIRT7

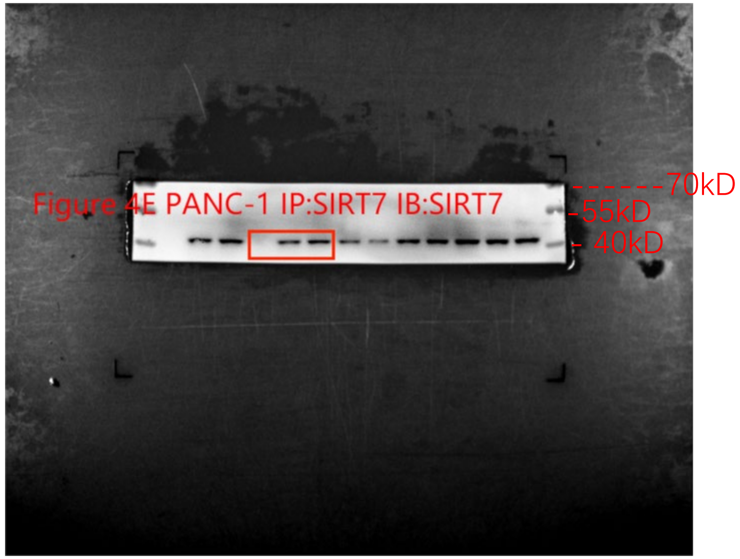

Figure 4E PANC-1 REGγ

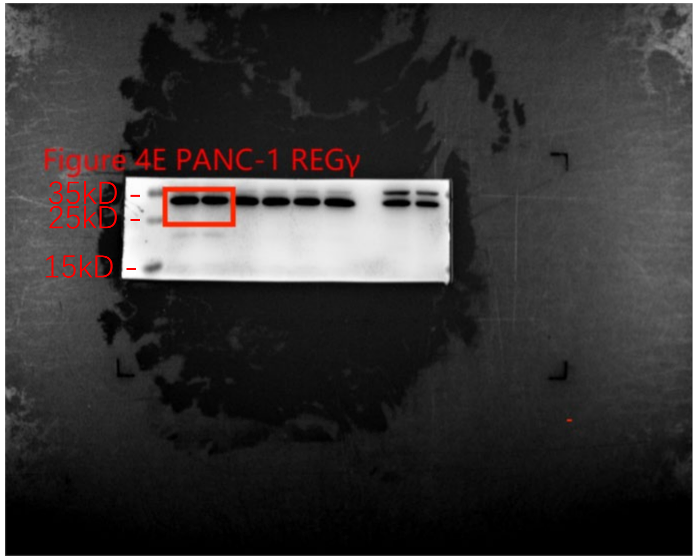

Figure 4E PANC-1 IB REGγ

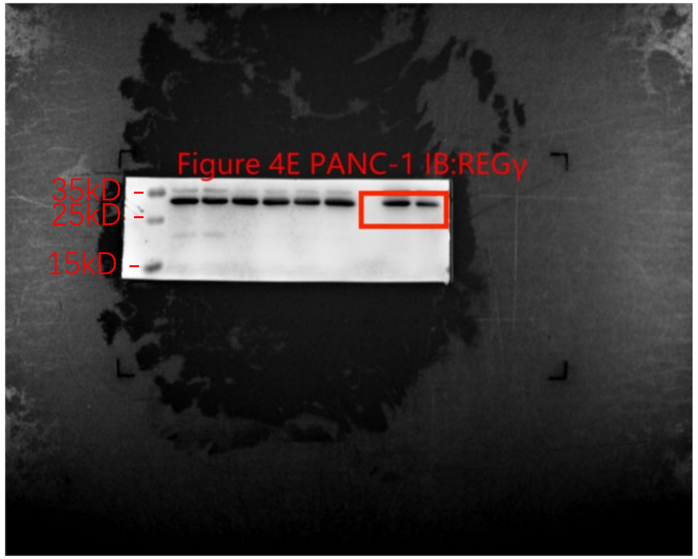

Figure 4E MiaPaCa-2 HA

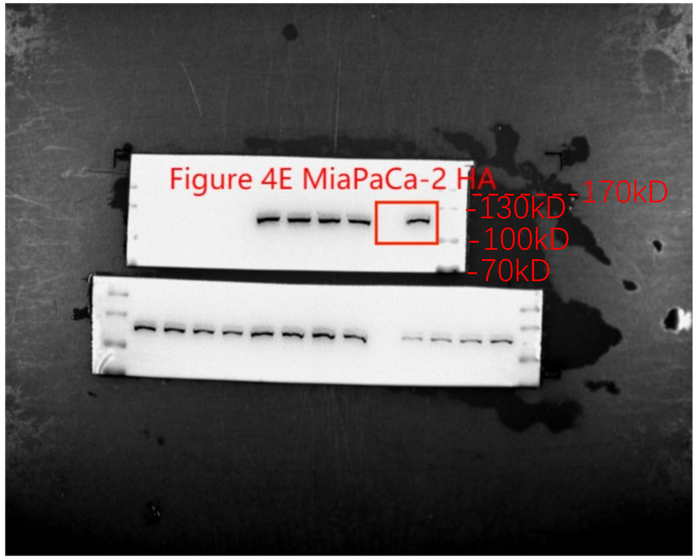

Figure 4E MiaPaCa-2 SIRT7

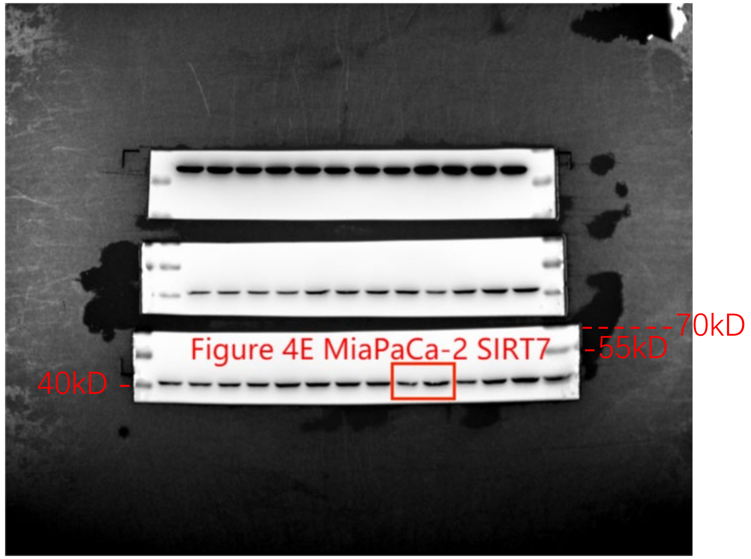

Figure 4E MiaPaCa-2 GAPDH

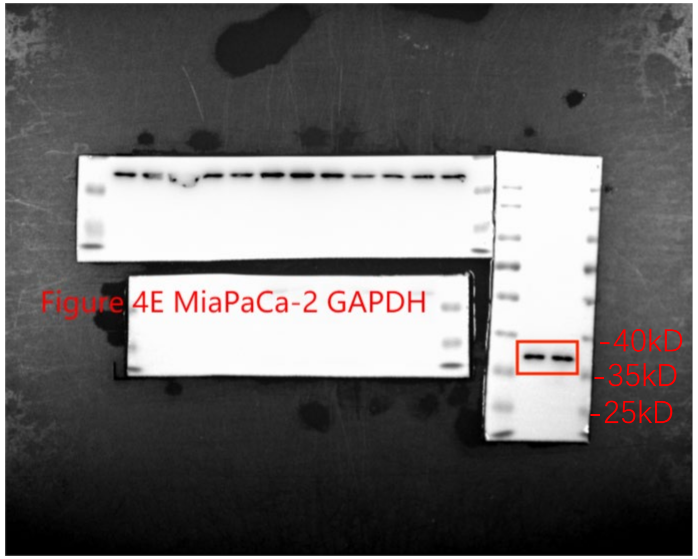

Figure 4E MiaPaCa-2 IP SIRT7 IB SIRT7

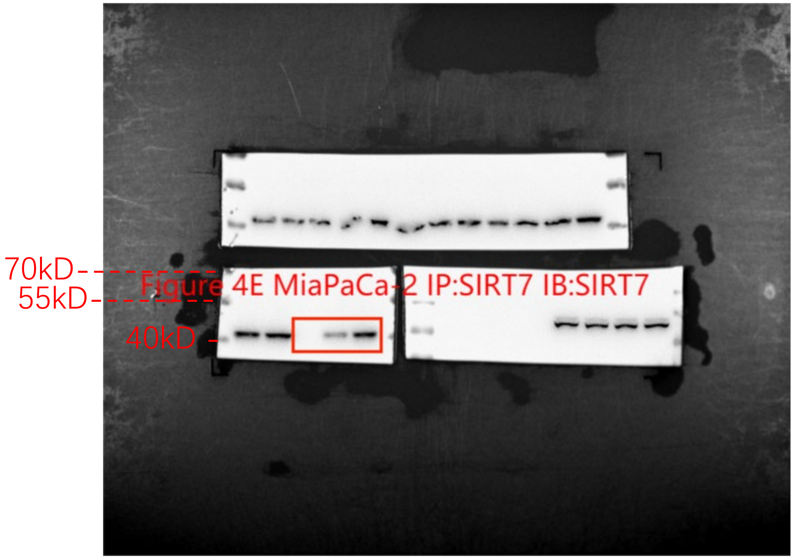

Figure 4E MiaPaCa-2 REGγ

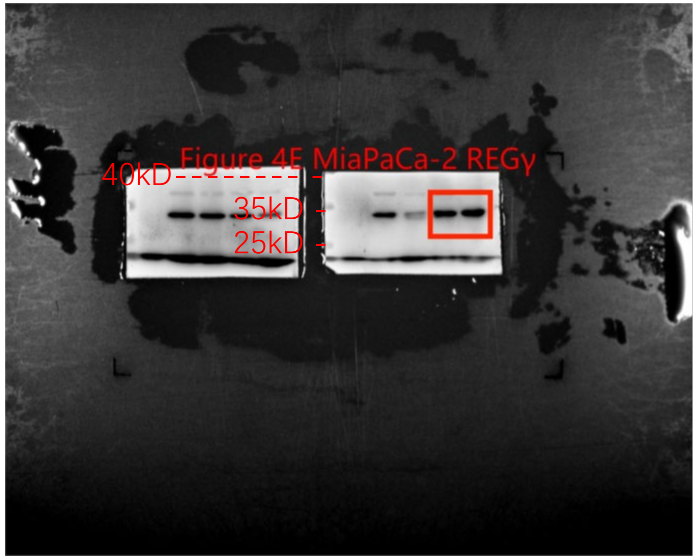

Figure 4E MiaPaCa-2 IB REGγ

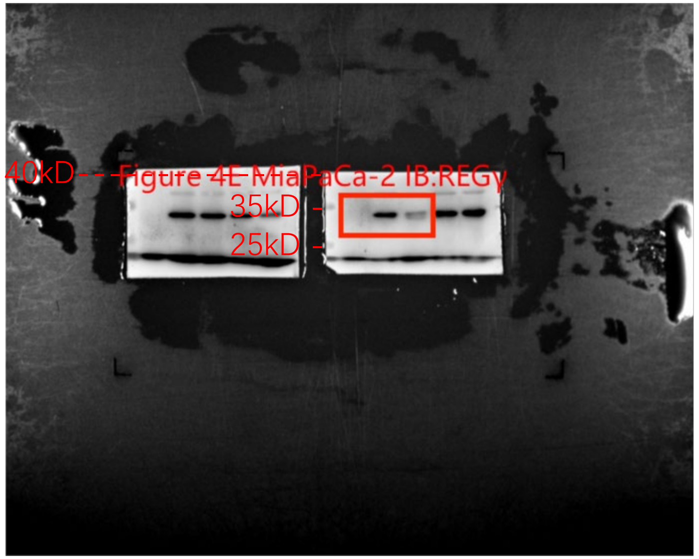

Figure 4F PANC-1 SIRT7

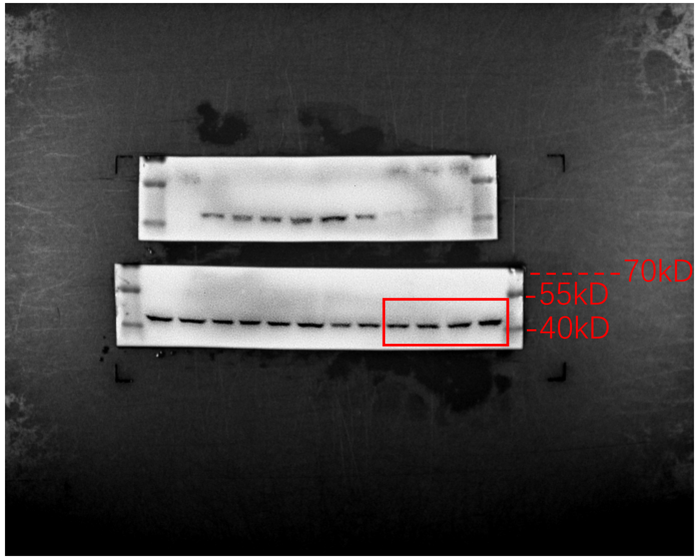

Figure 4F PANC-1 GAPDH

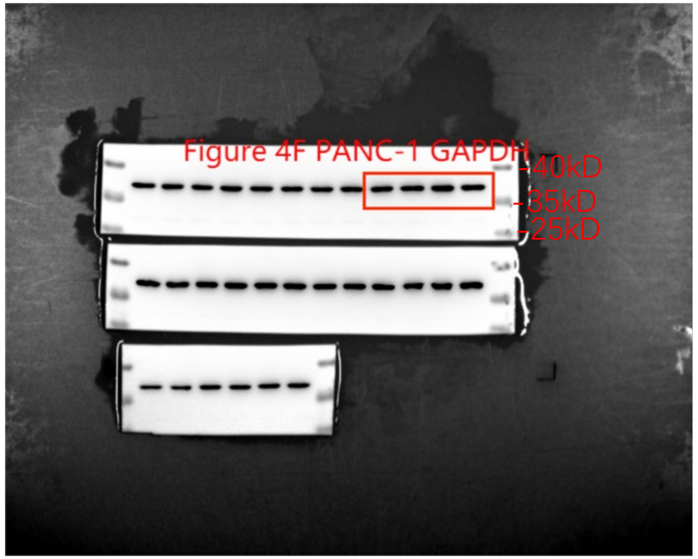

Figure 4F PANC-1 IP SIRT7 IB SIRT7

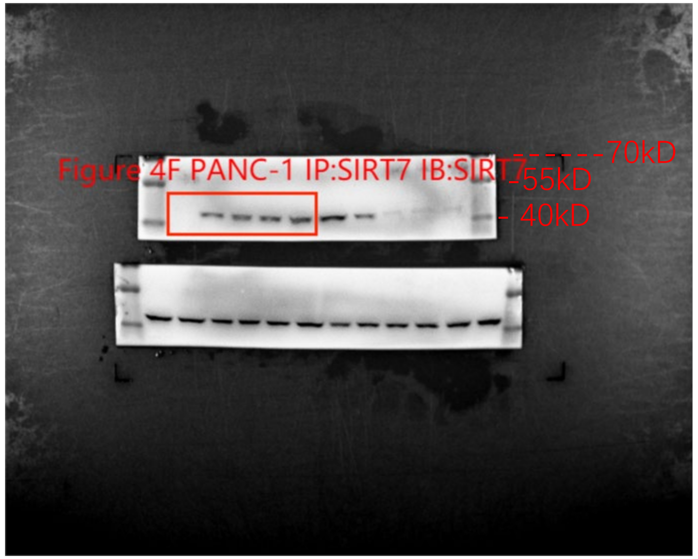

Figure 4F PANC-1 REGγ

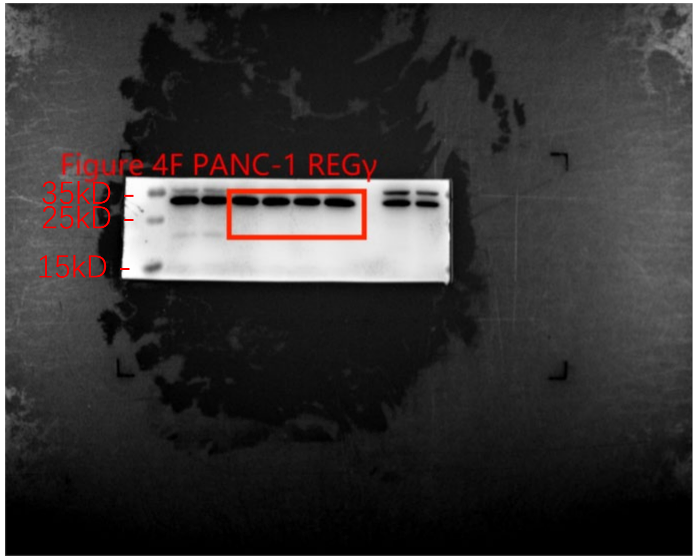

Figure 4F PANC-1 IB REGγ

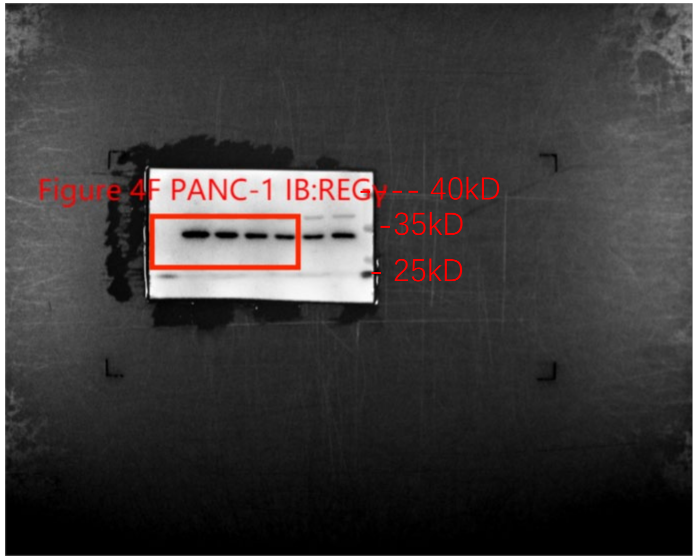

Figure 4F MiaPaCa-2 SIRT7

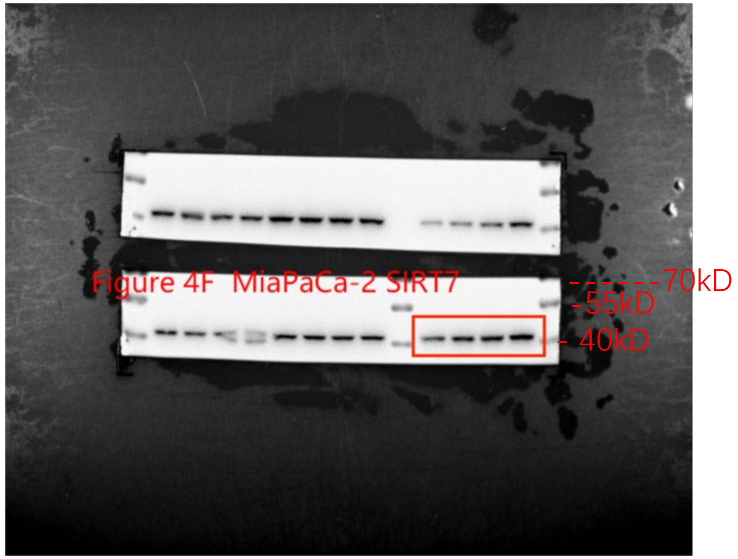

Figure 4F MiaPaCa-2 GAPDH

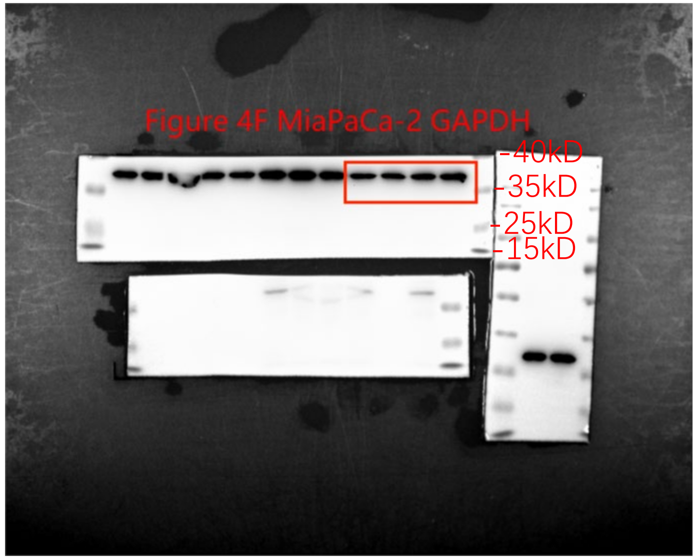

Figure 4F MiaPaCa-2 IP SIRT7 IB SIRT7

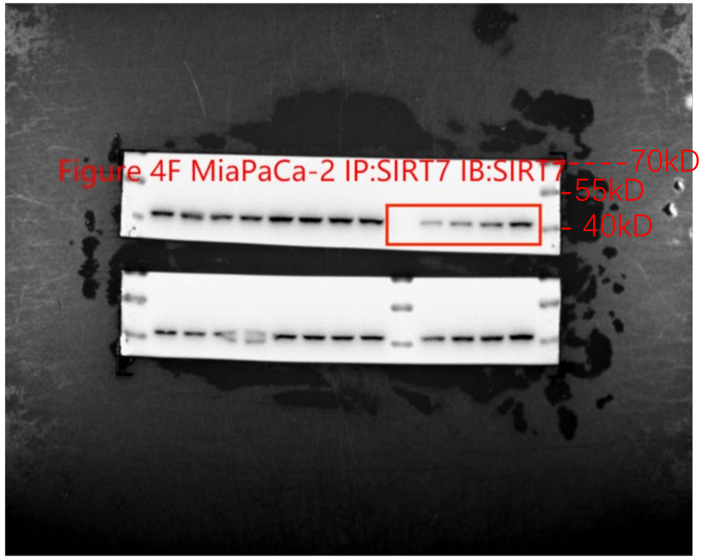

Figure 4F MiaPaCa-2 REGγ

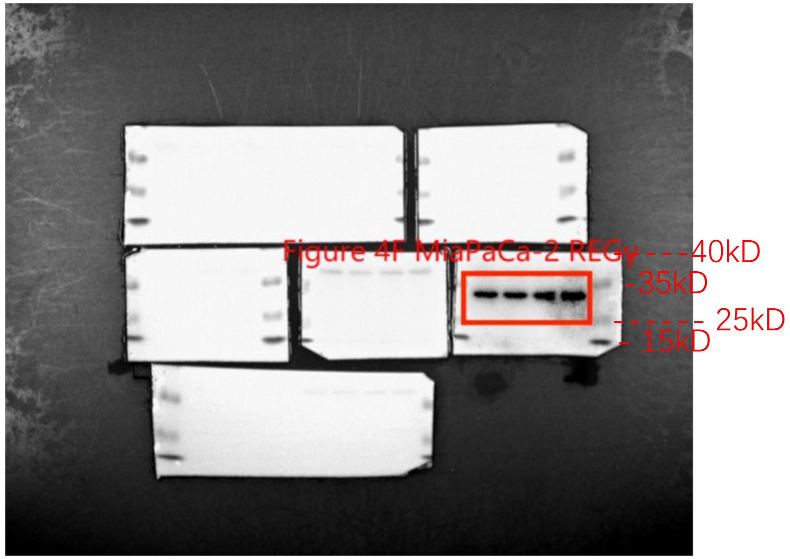

Figure 4F MiaPaCa-2 IB REGγ

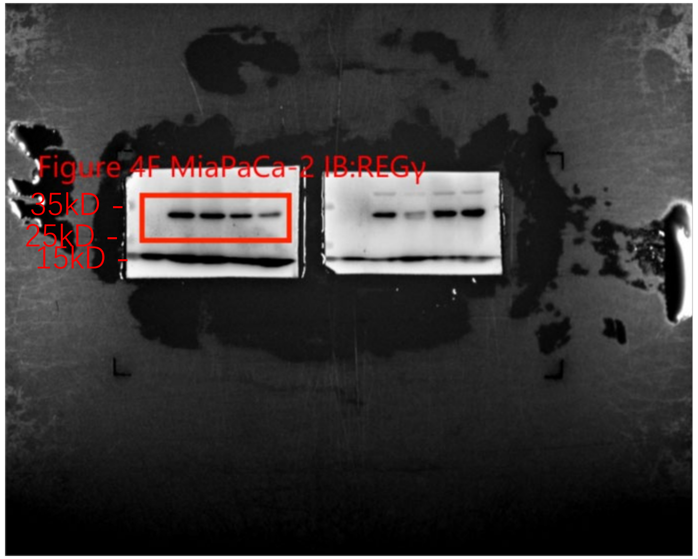

Figure 4G PANC-1 HA

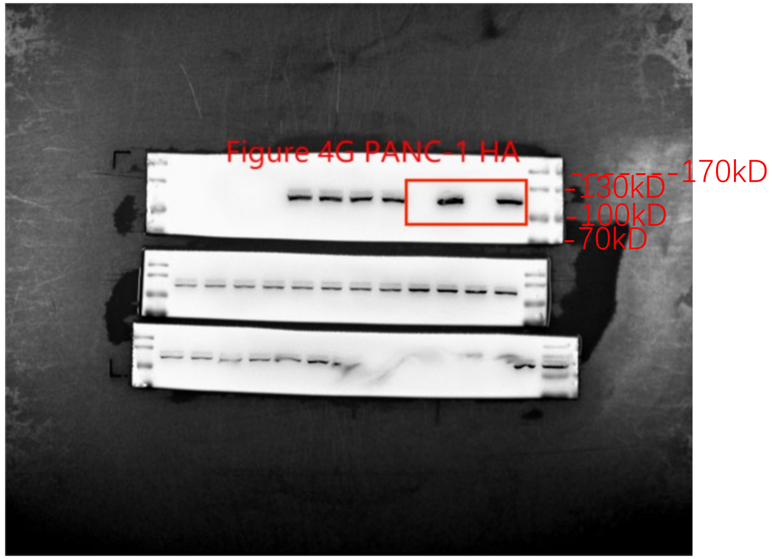

Figure 4G PANC-1 REGγ

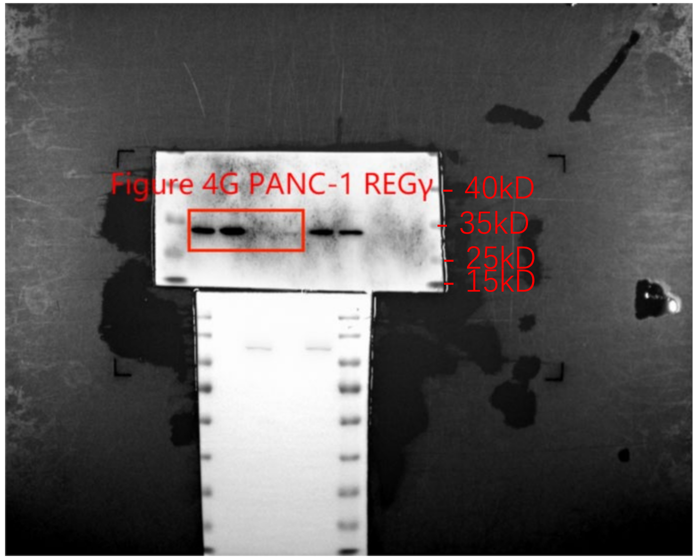

Figure 4G PANC-1 SIRT7

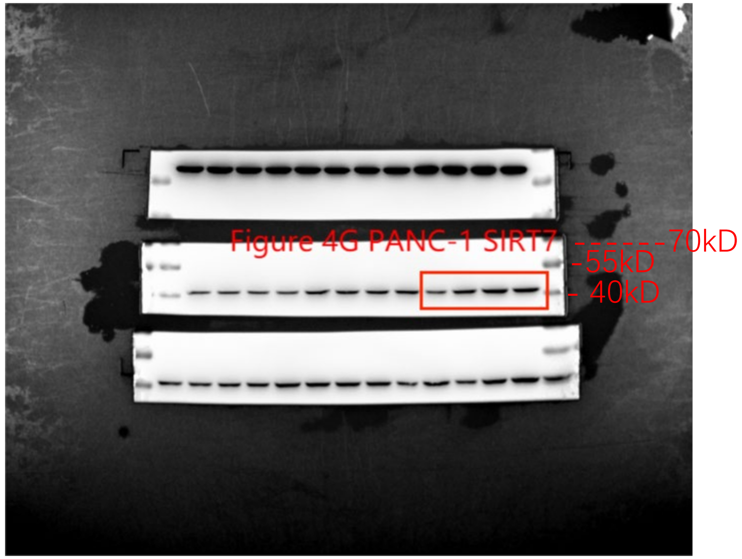

Figure 4G PANC-1 GAPDH

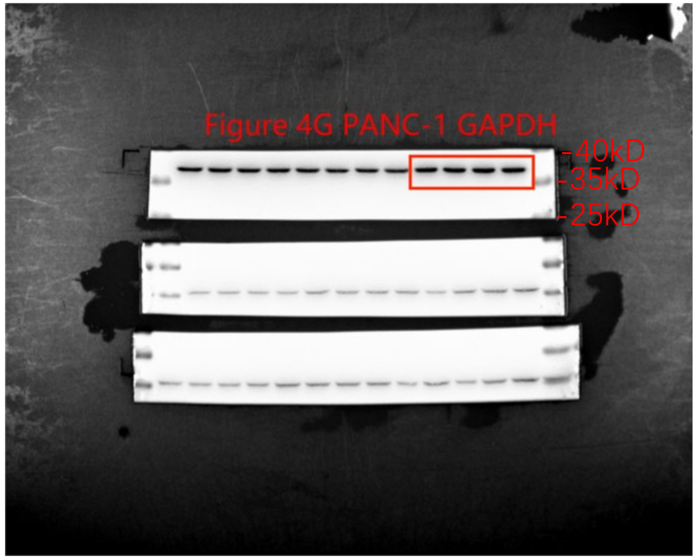

Figure 4G MiaPaCa-2 HA

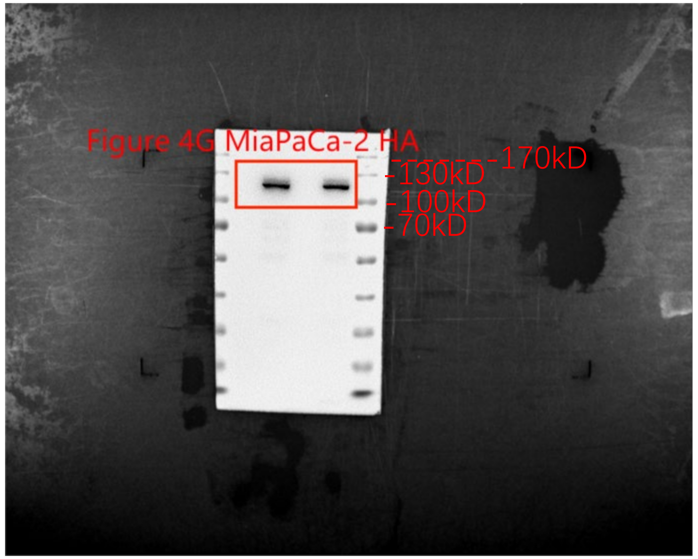

Figure 4G MiaPaCa-2 REGγ

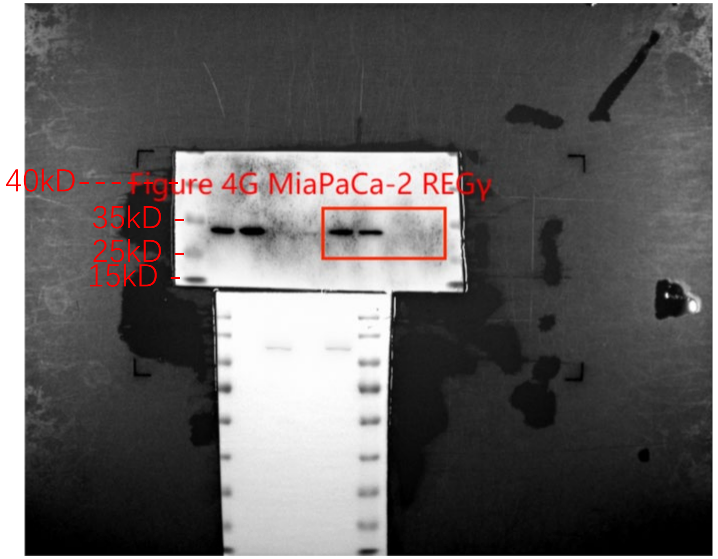

Figure 4G MiaPaCa-2 SIRT7

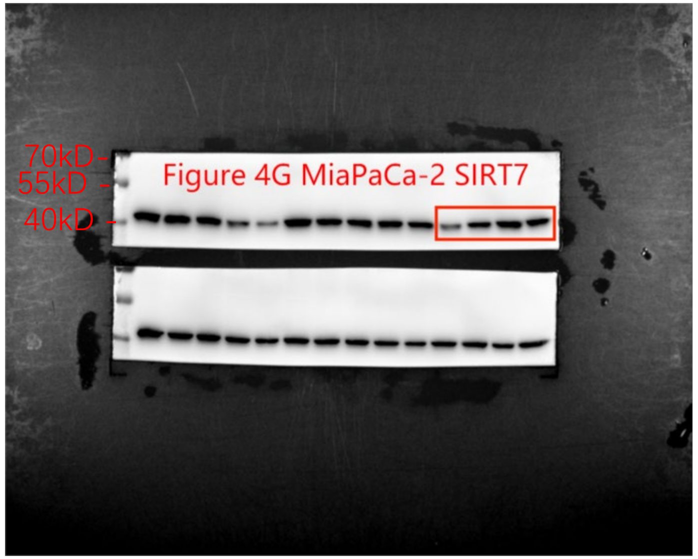

Figure 4G MiaPaCa-2 GAPDH

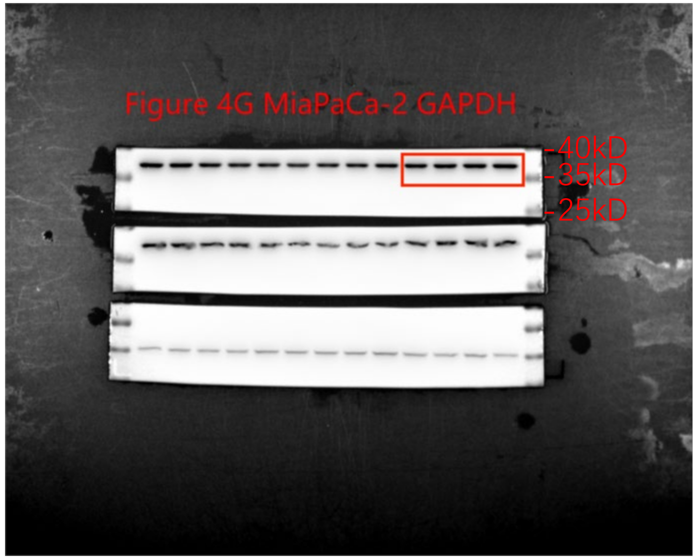

Figure 5A PANC-1 OGT

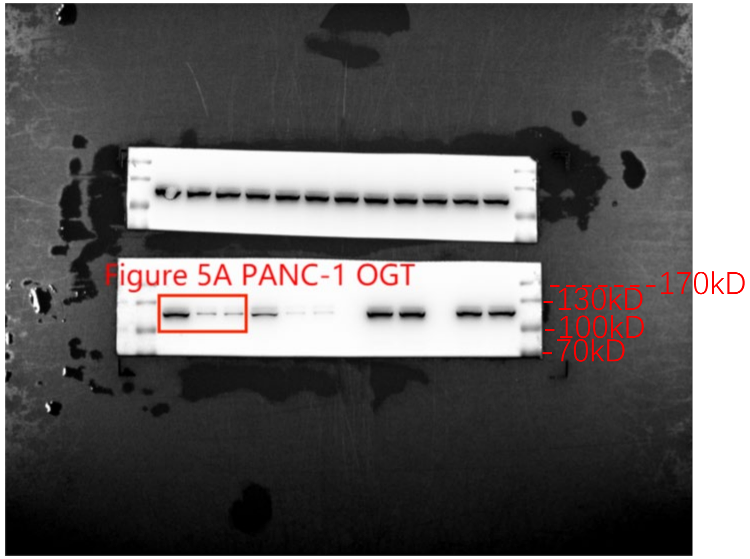

Figure 5A MiaPaCa-2 OGT

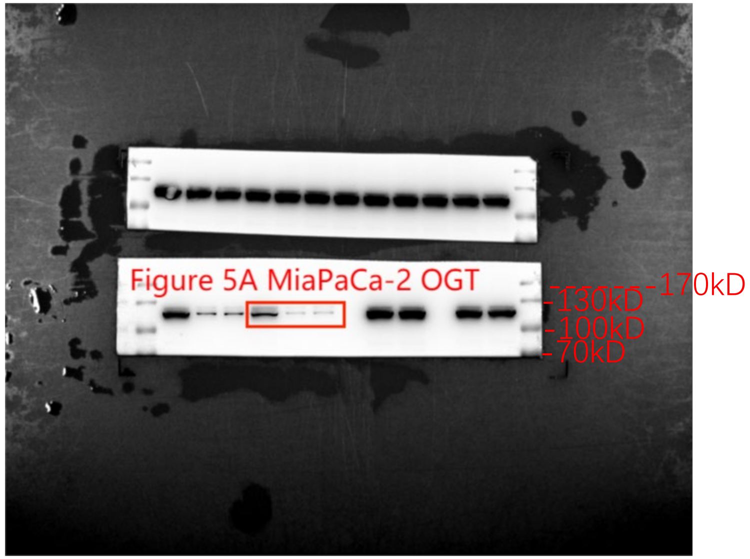

Figure 5A PANC-1 H3

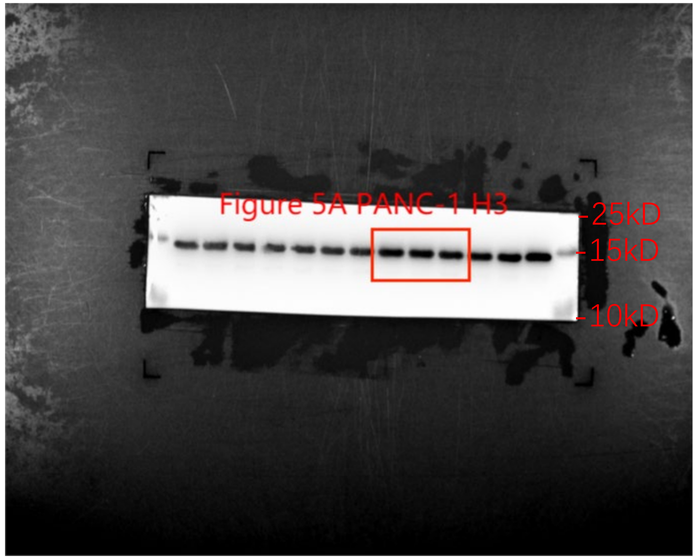

Figure 5A MiaPaCa-2 H3

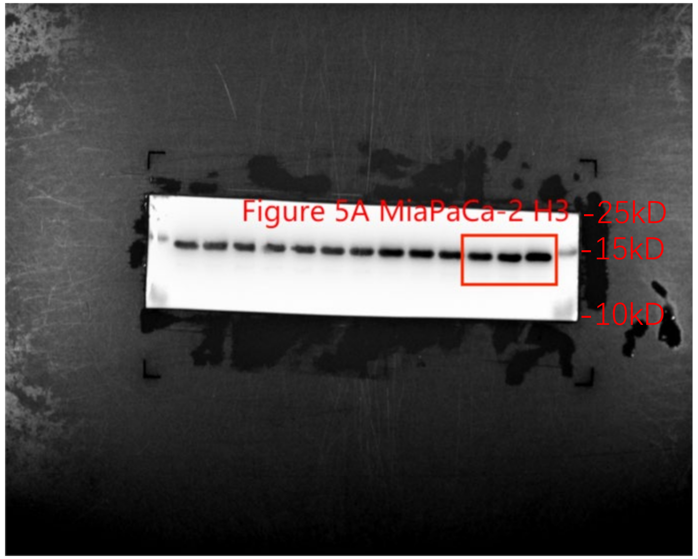

Figure 5A PANC-1 H3K18Ac

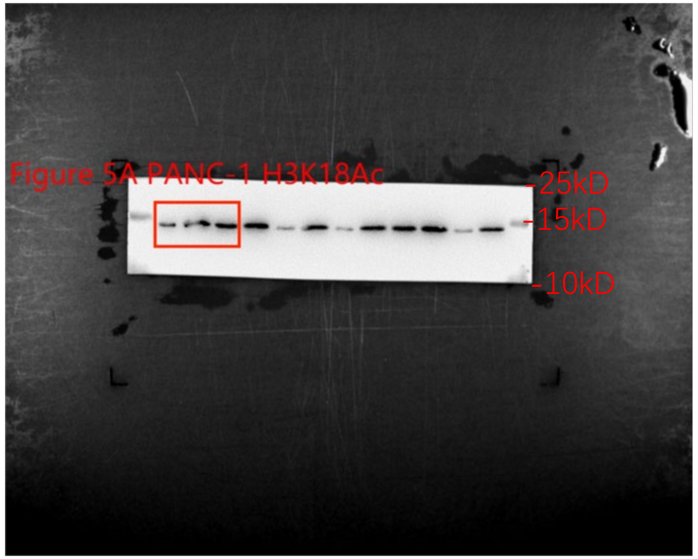

Figure 5A MiaPaCa-2 H3K18Ac

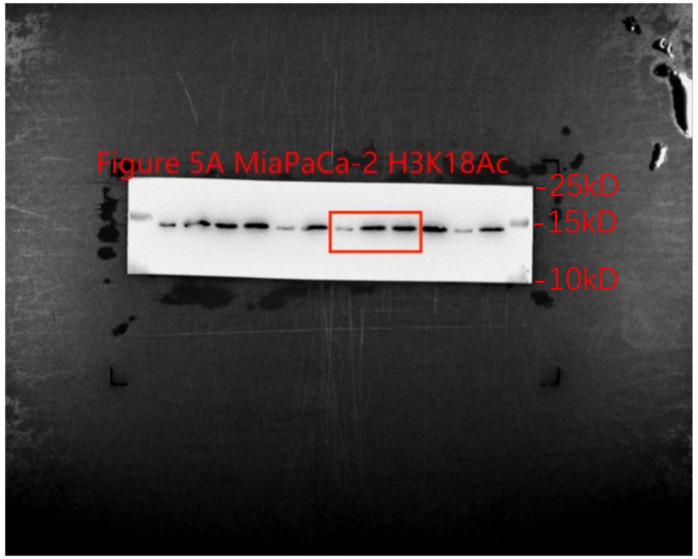

Figure 5A PANC-1 H3K9Ac

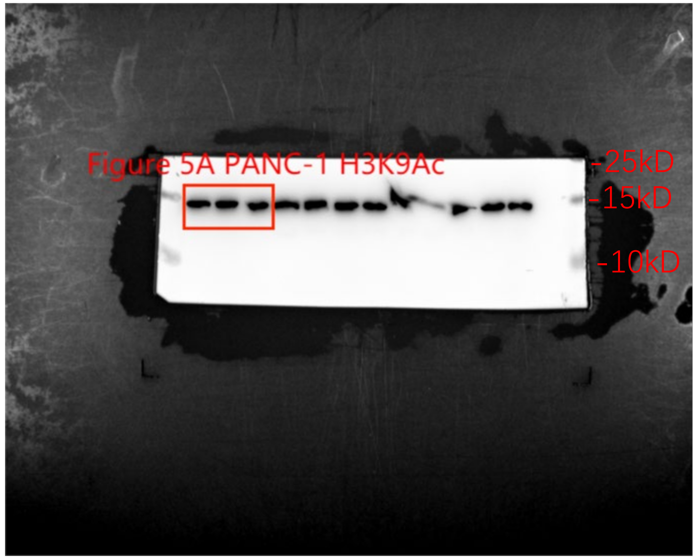

Figure 5A MiaPaCa-2 H3K9Ac

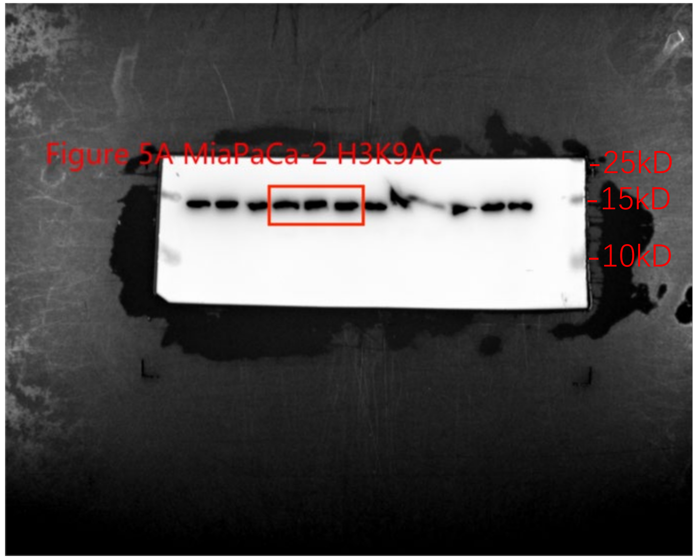

Figure 5A PANC-1 H3K14Ac

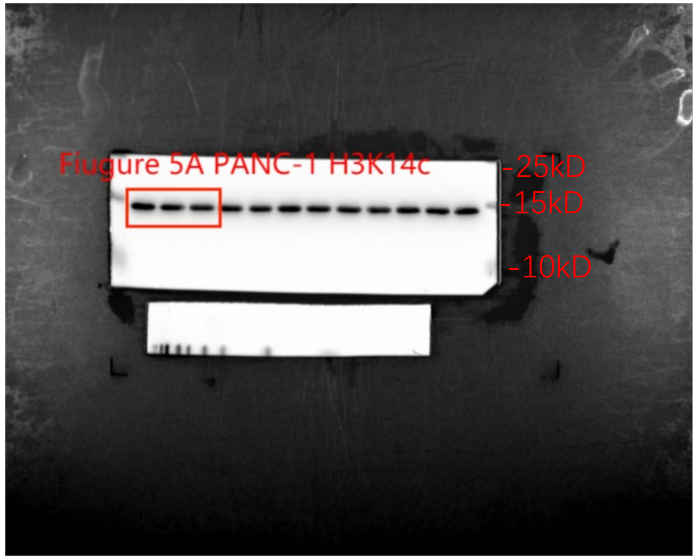

Figure 5A MiaPaCa-2 H3K14Ac

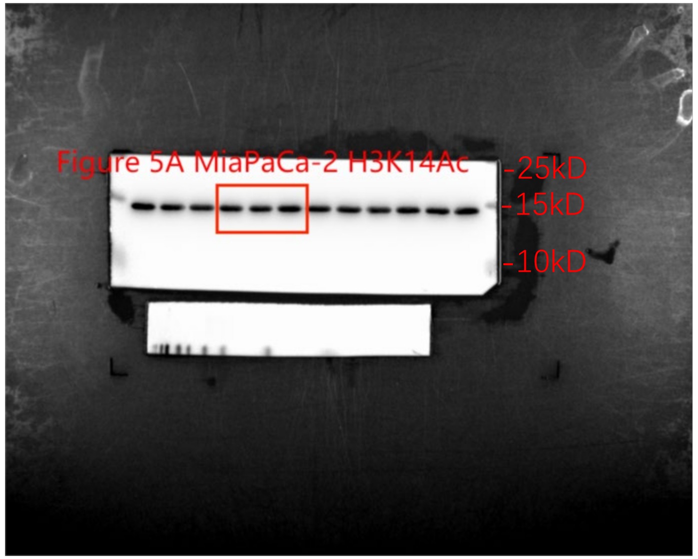

Figure 5A PANC-1 H3K23Ac

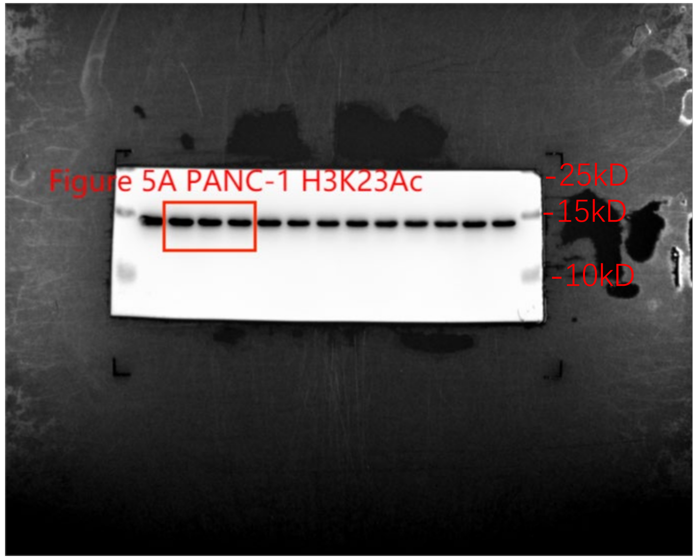

Figure 5A MiaPaCa-2 H3K23Ac

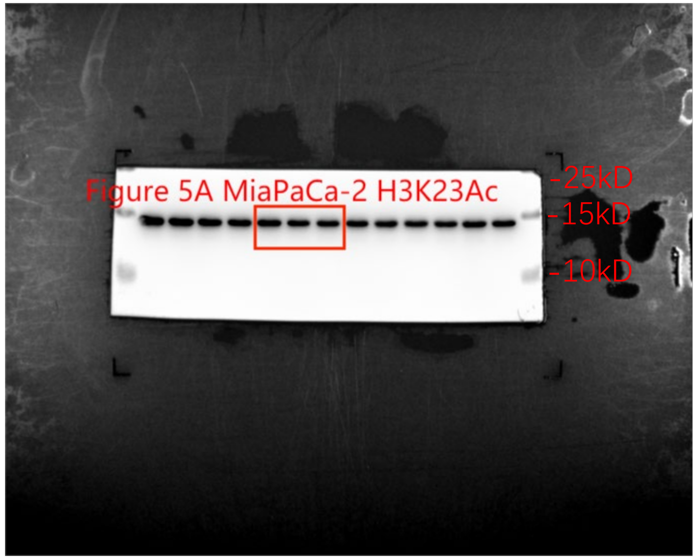

Figure 5A PANC-1 H4

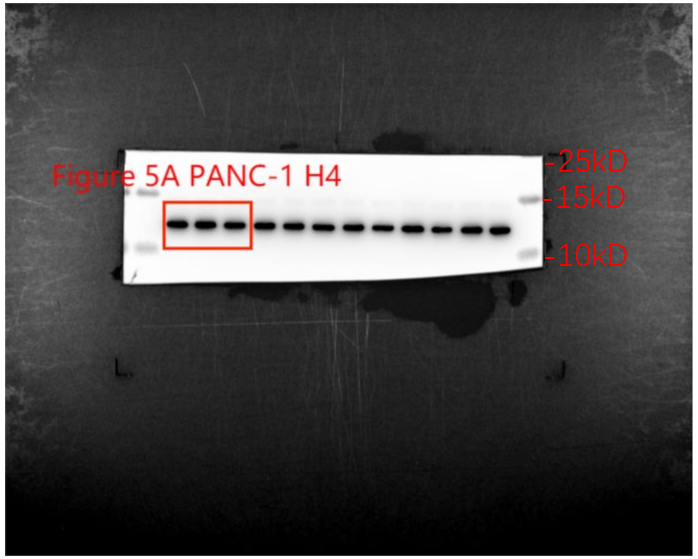

Figure 5A MiaPaCa-2 H4

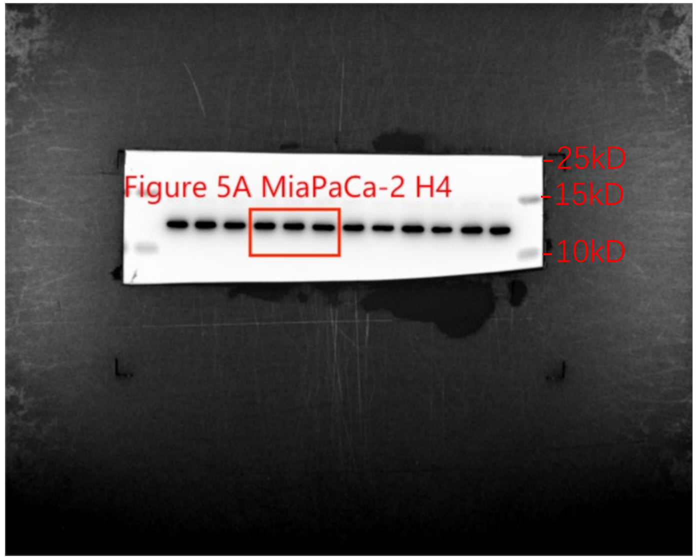

Figure 5A PANC-1 H4K16Ac

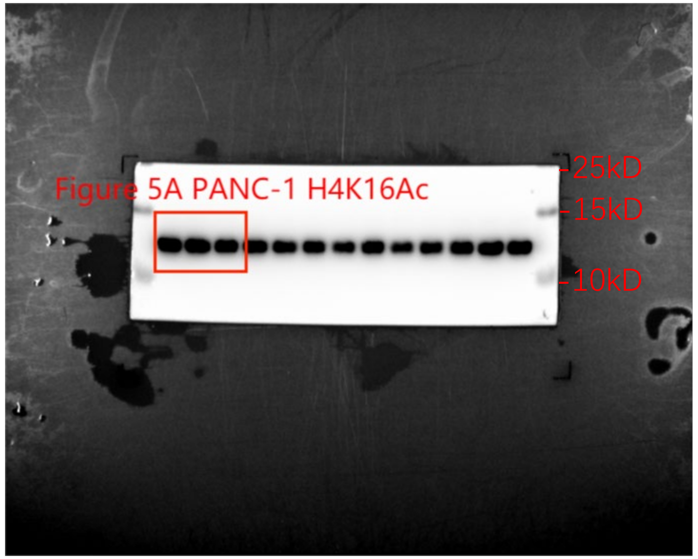

Figure 5A MiaPaCa-2 H4K16Ac

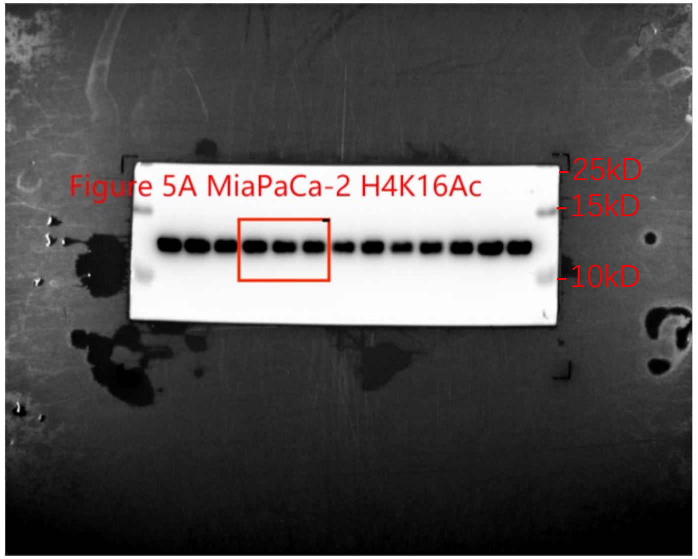

Figure 5B PANC-1 HA

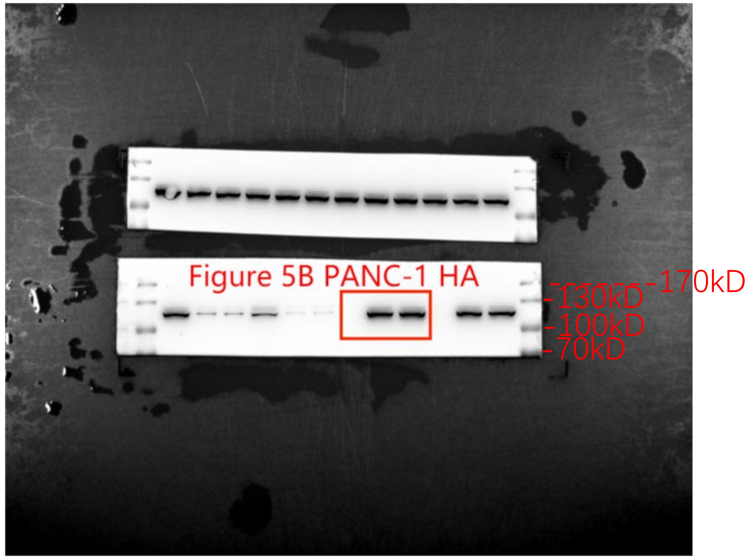

Figure 5B MiaPaCa-2 HA

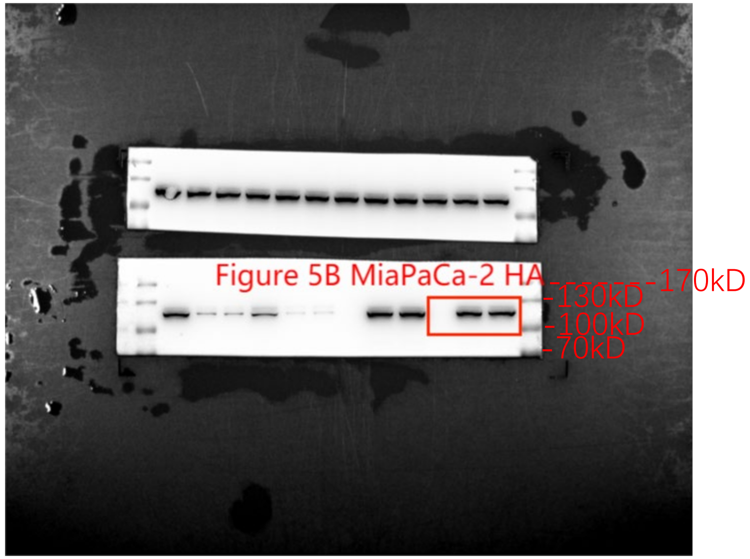

Figure 5B PANC-1 H3

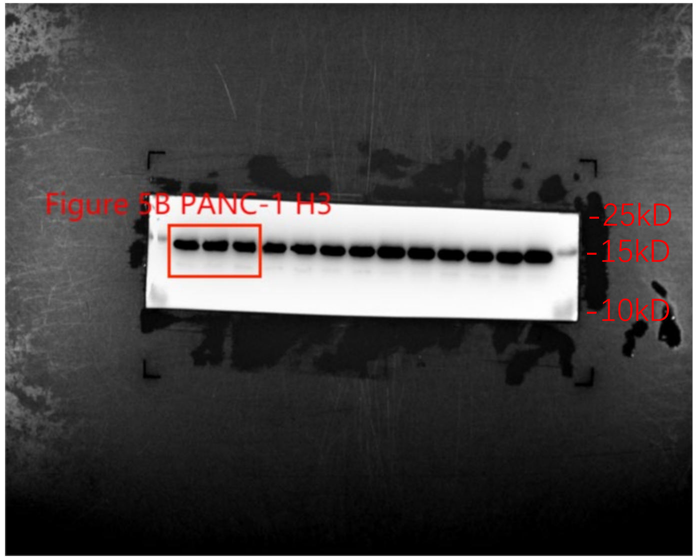

Figure 5B MiaPaCa-2 H3

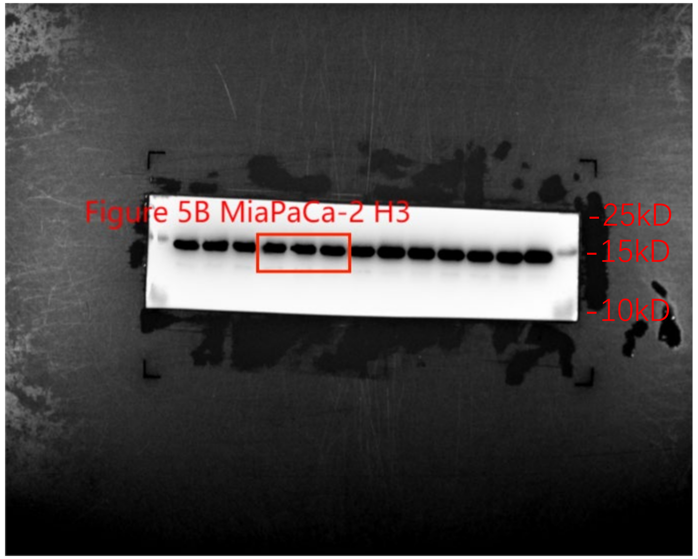

Figure 5B PANC-1 H3K18Ac

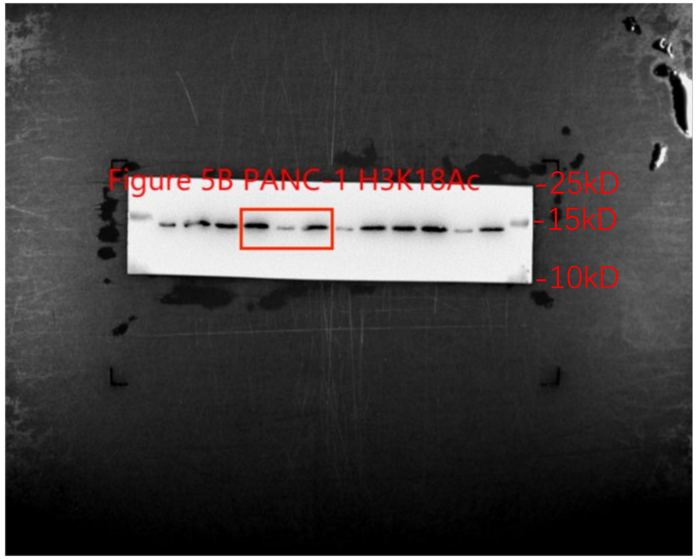

Figure 5B MiaPaCa-2 H3K18Ac

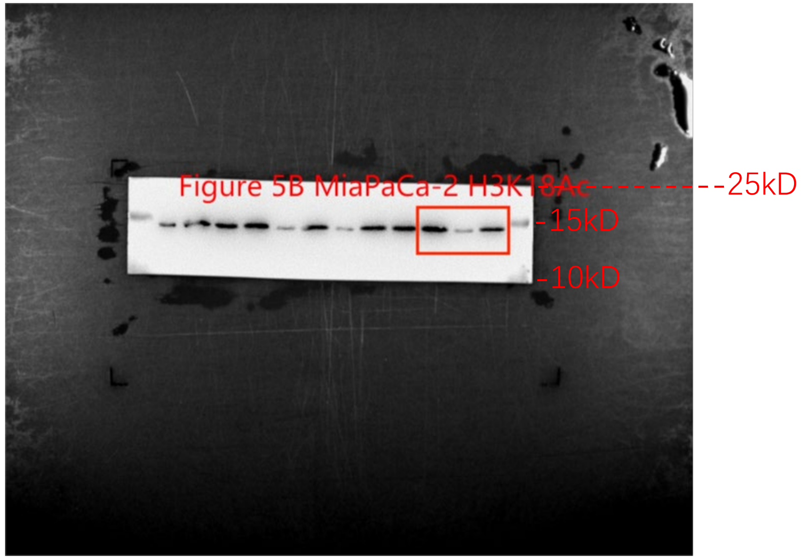

Figure 5B PANC-1 H3K9Ac

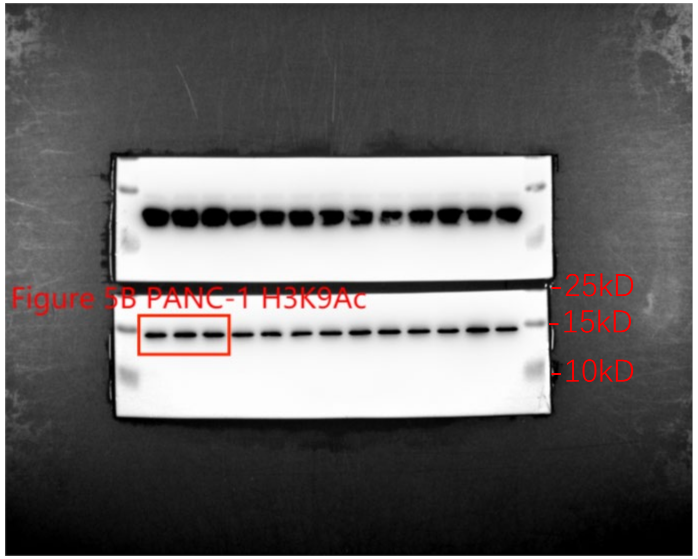

Figure 5B MiaPaCa-2 H3K9Ac

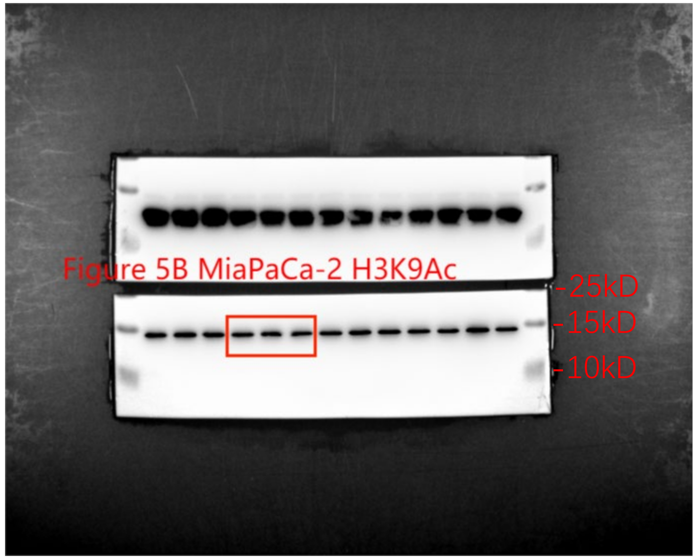

Figure 5B PANC-1 H3K14Ac

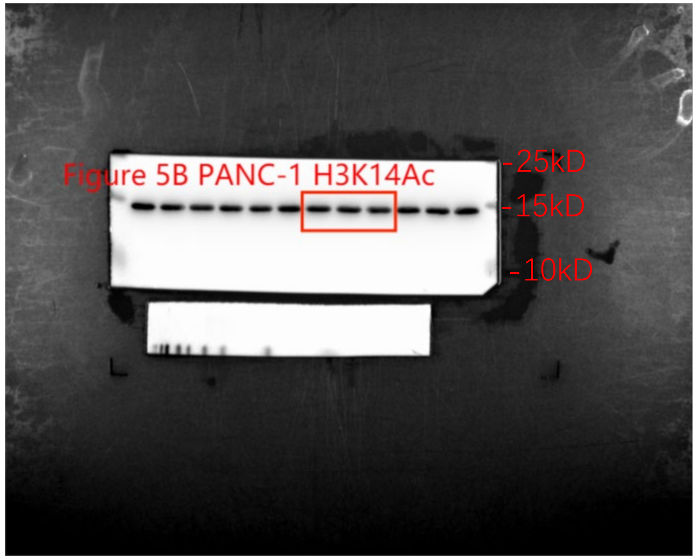

Figure 5B MiaPaCa-2 H3K14Ac

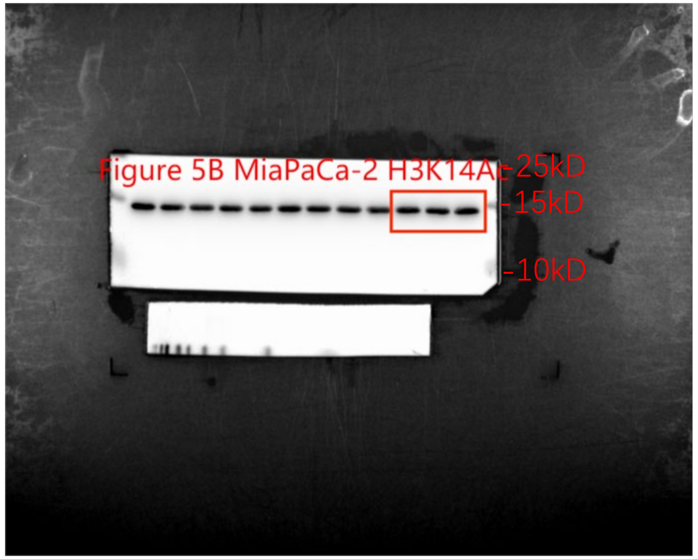

Figure 5B PANC-1 H3K23Ac

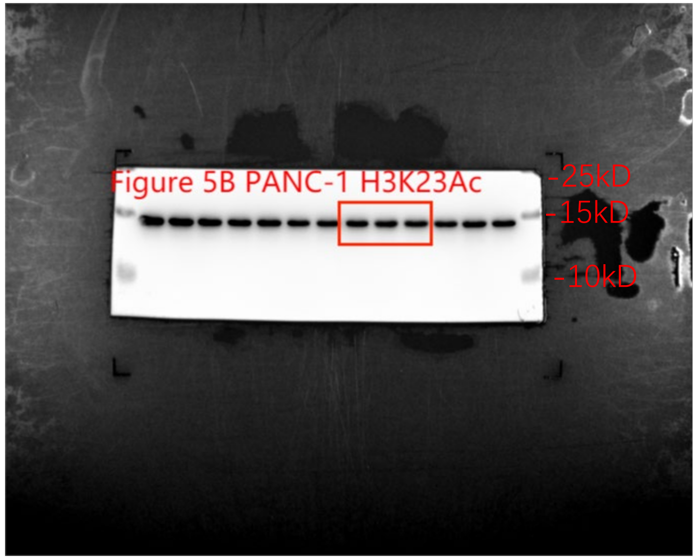

Figure 5B MiaPaCa-2 H3K23Ac

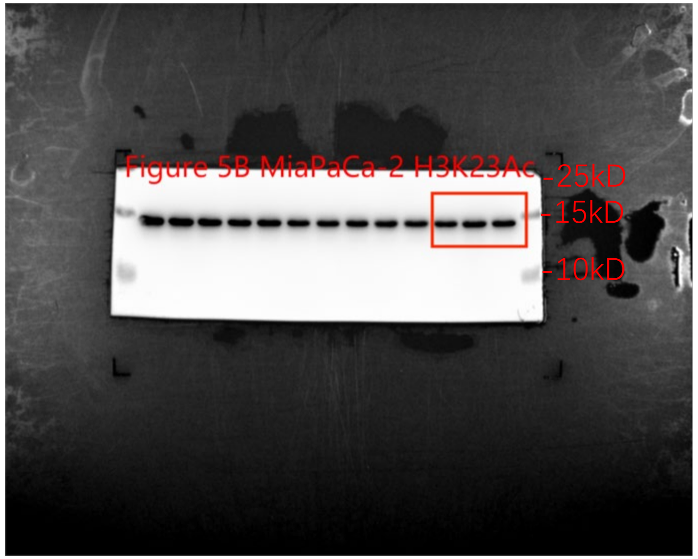

Figure 5B PANC-1 H4

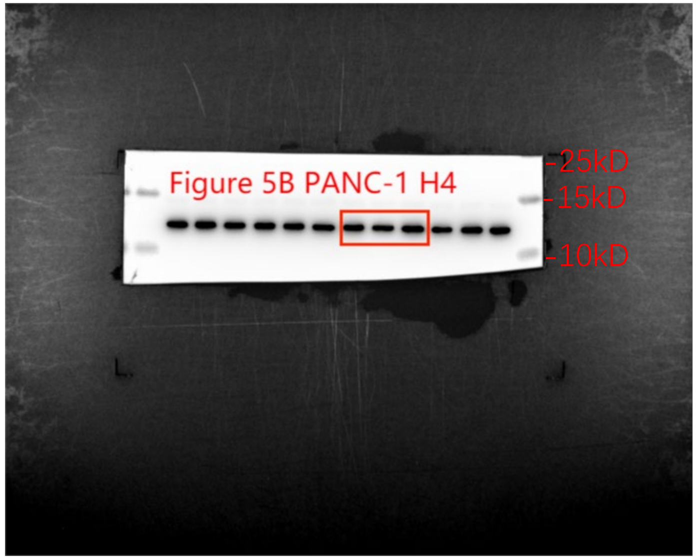

Figure 5B MiaPaCa-2 H4

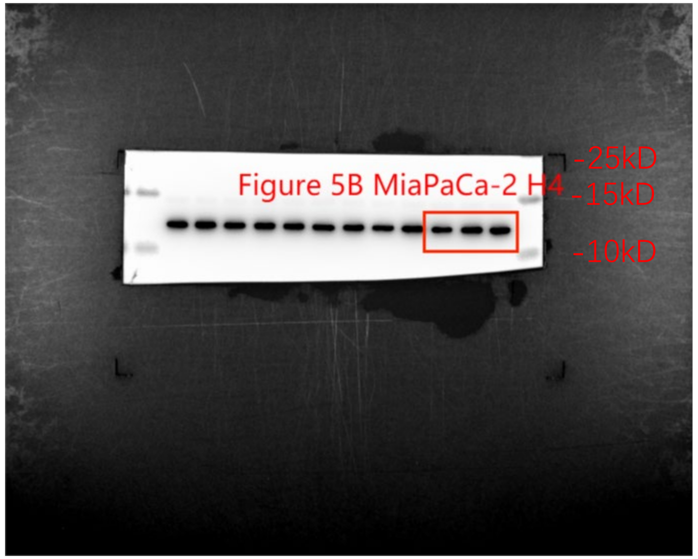

Figure 5B PANC-1 H4K16Ac

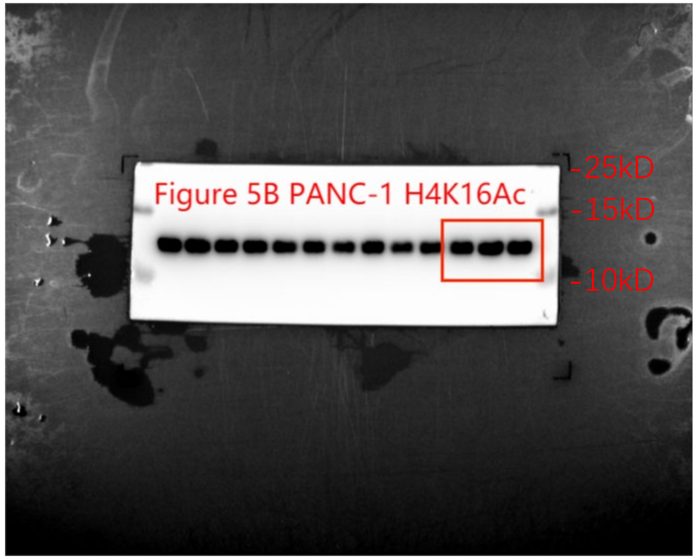

Figure 5B MiaPaCa-2 H4K16Ac

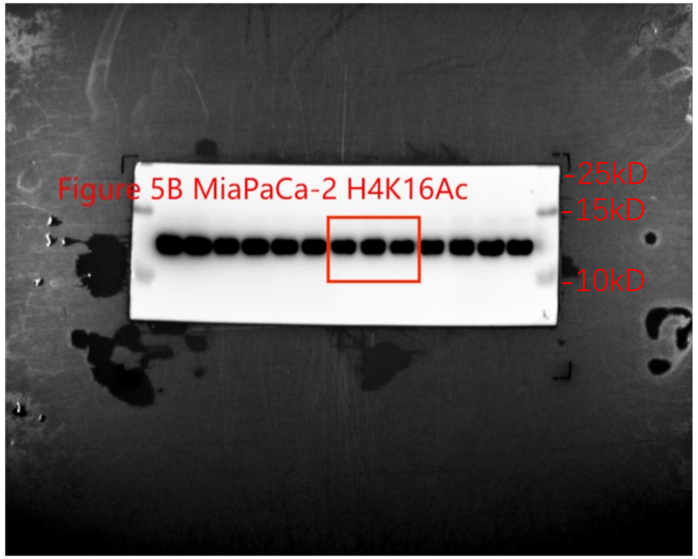

Figure 5C PANC-1 HA

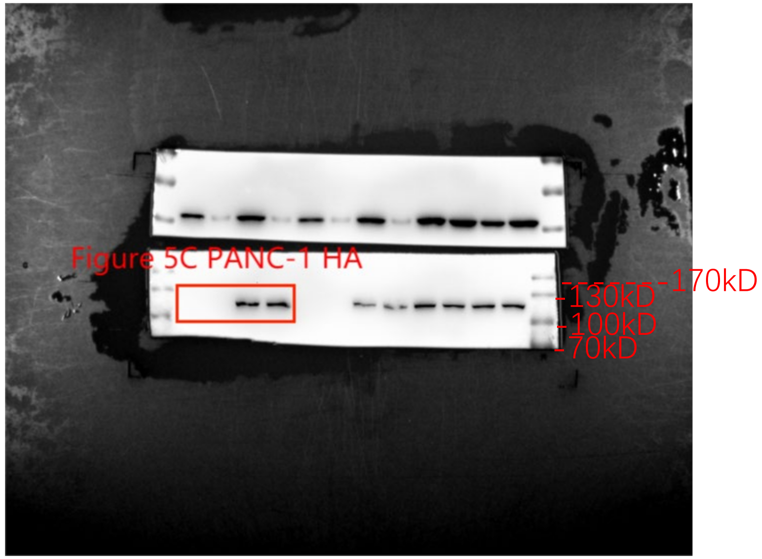

Figure 5C MiaPaCa-2 HA

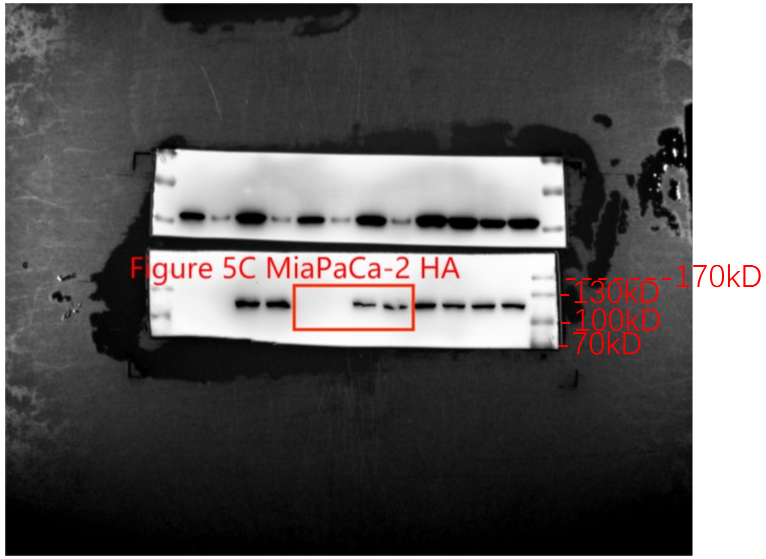

Figure 5C PANC-1 SIRT7

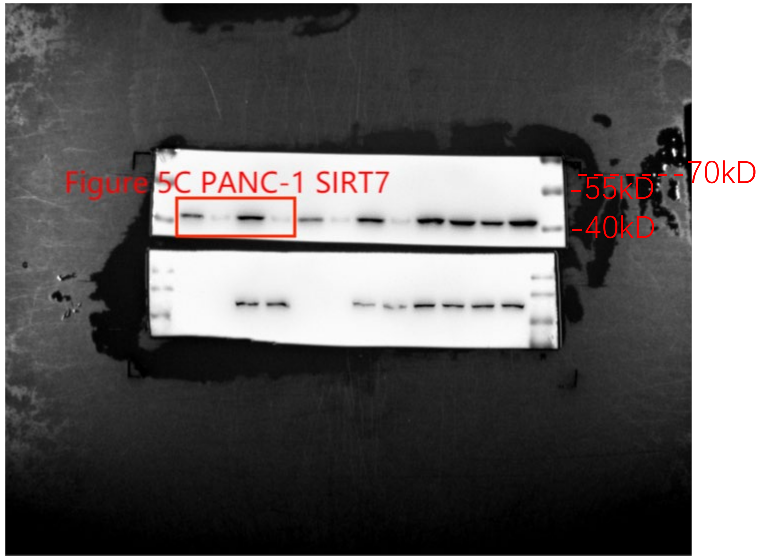

Figure 5C MiaPaCa-2 SIRT7

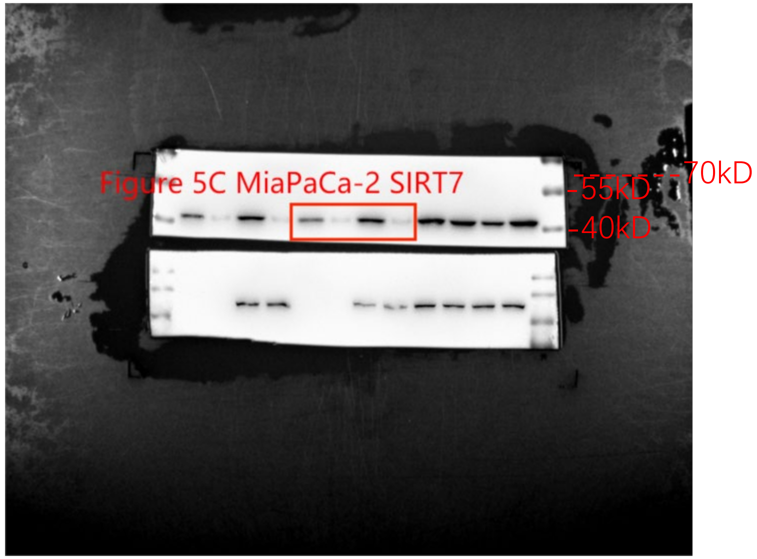

Figure 5C PANC-1 H3

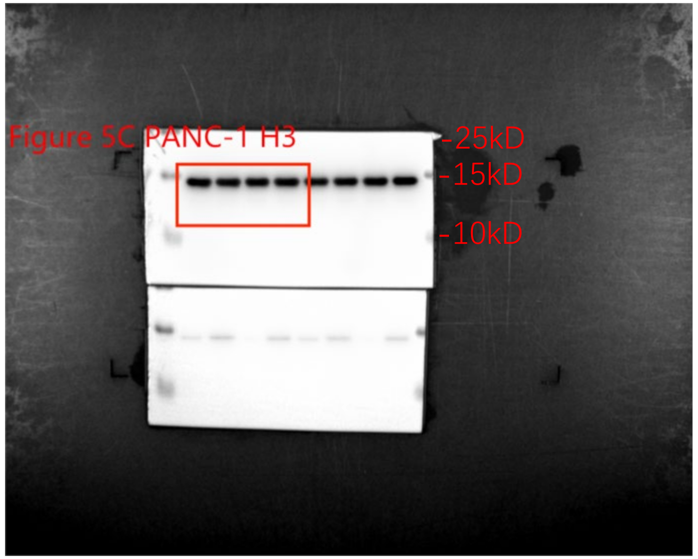

Figure 5C MiaPaCa-2 H3

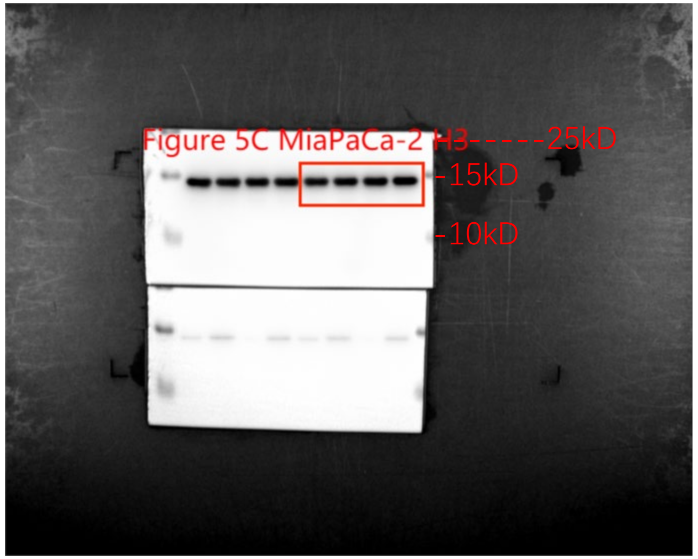

Figure 5C PANC-1 H3K18Ac

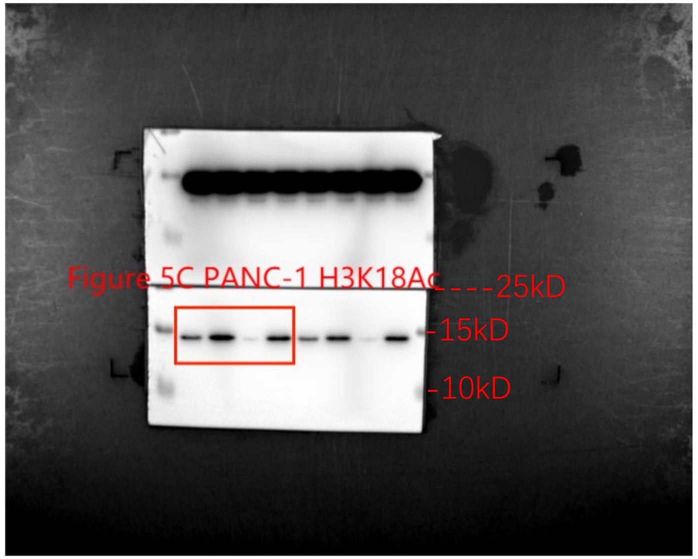

Figure 5C MiaPaCa-2 H3K18Ac

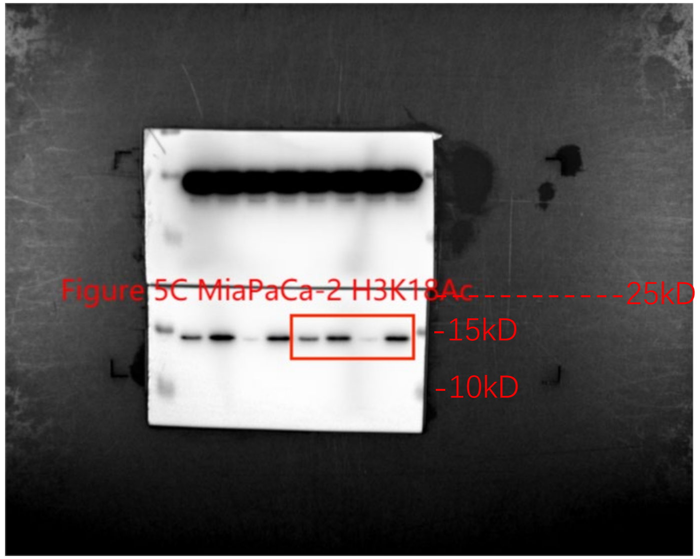

Figure 5C PANC-1 H3K9Ac

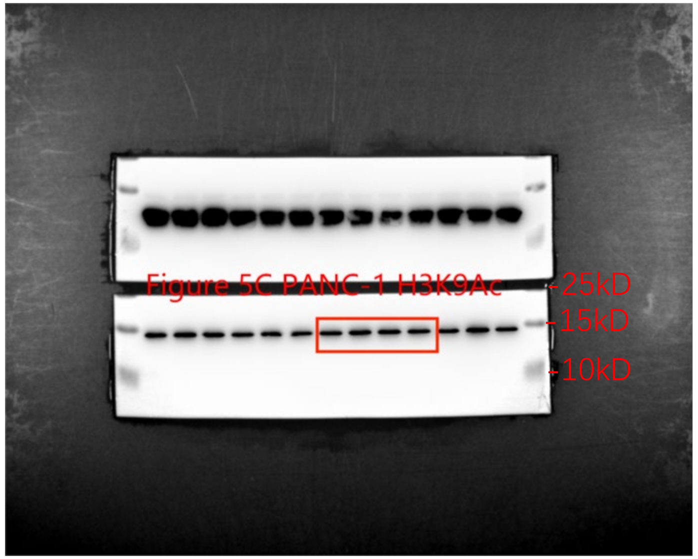

Figure 5C MiaPaCa-2 H3K9Ac

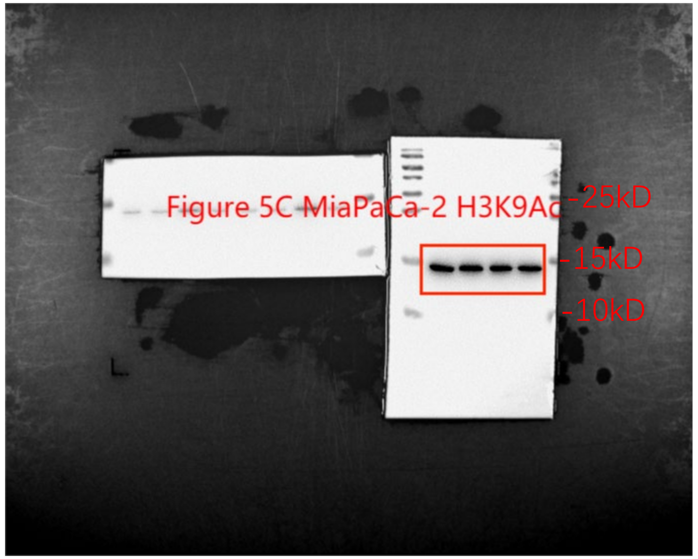

Figure 5C PANC-1 H3K14Ac

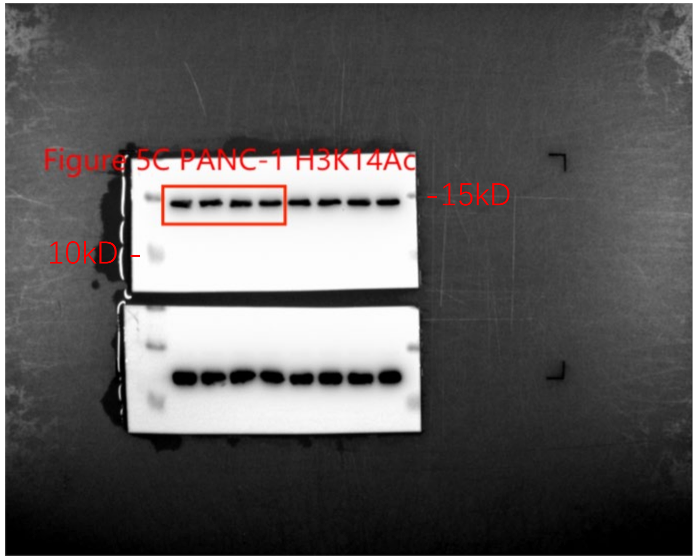

Figure 5C MiaPaCa-2 H3K14Ac

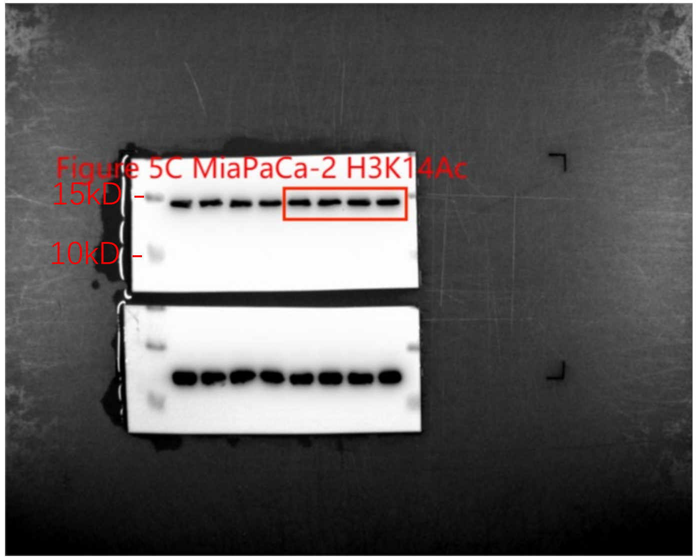

Figure 5C PANC-1 H3K23Ac

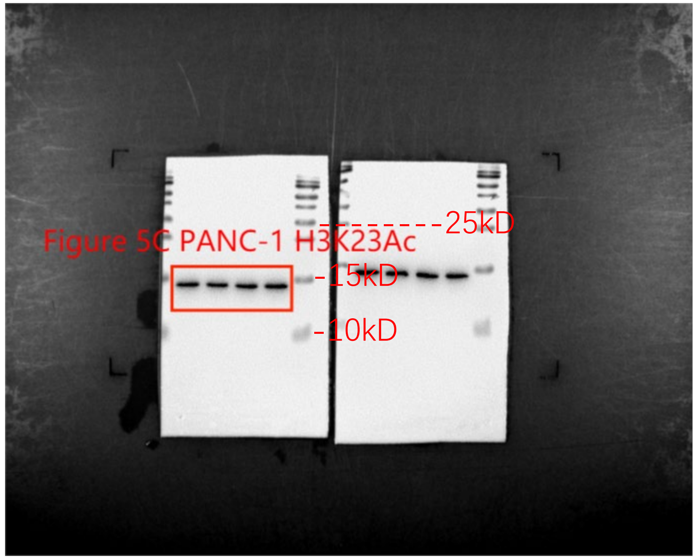

Figure 5C MiaPaCa-2 H3K23Ac

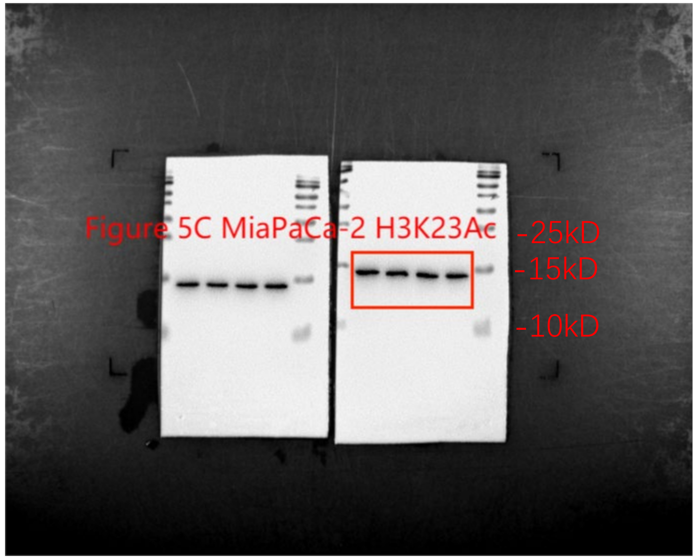

Figure 5C PANC-1 H4

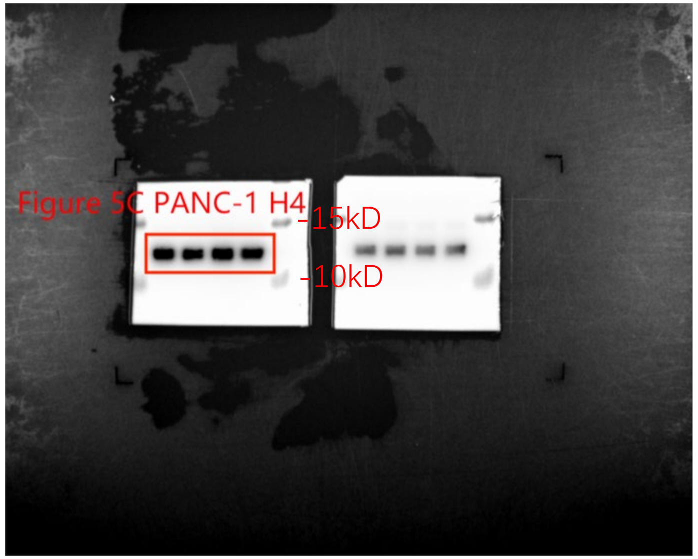

Figure 5C MiaPaCa-2 H4

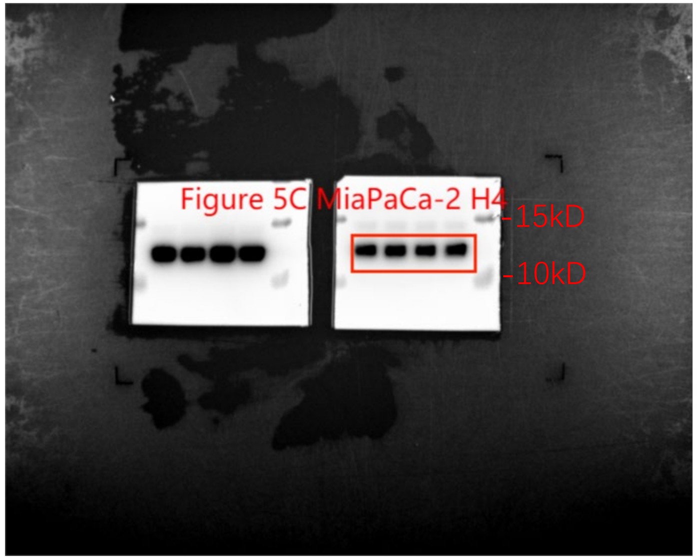

Figure 5C PANC-1 H4K16Ac

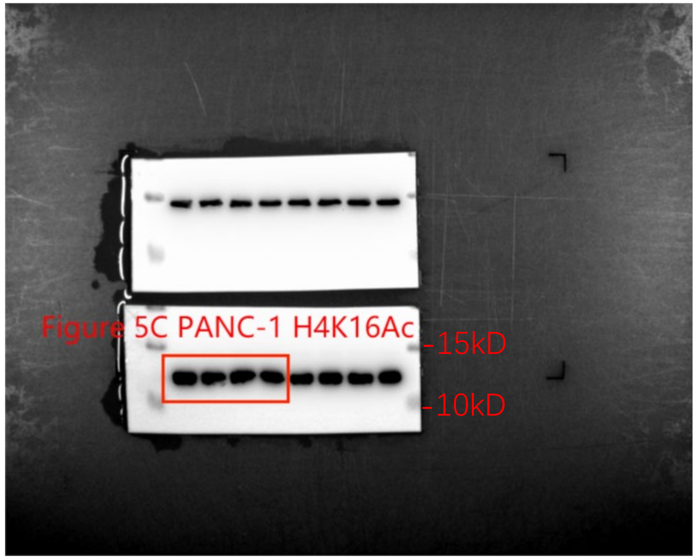

Figure 5C MiaPaCa-2 H4K16Ac

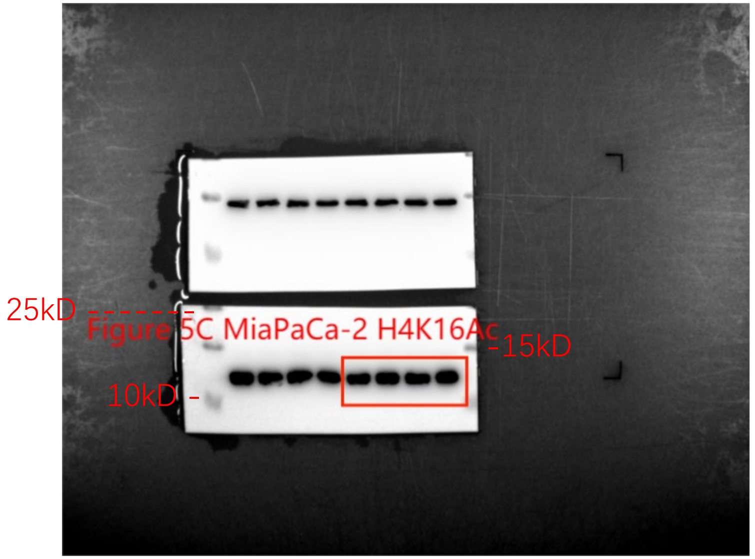

Figure 6B PANC-1 Flag

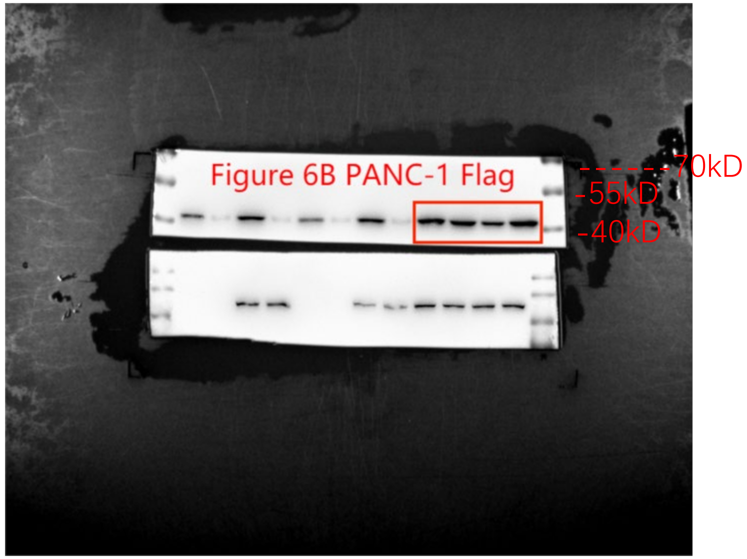

Figure 6B PANC-1 HA

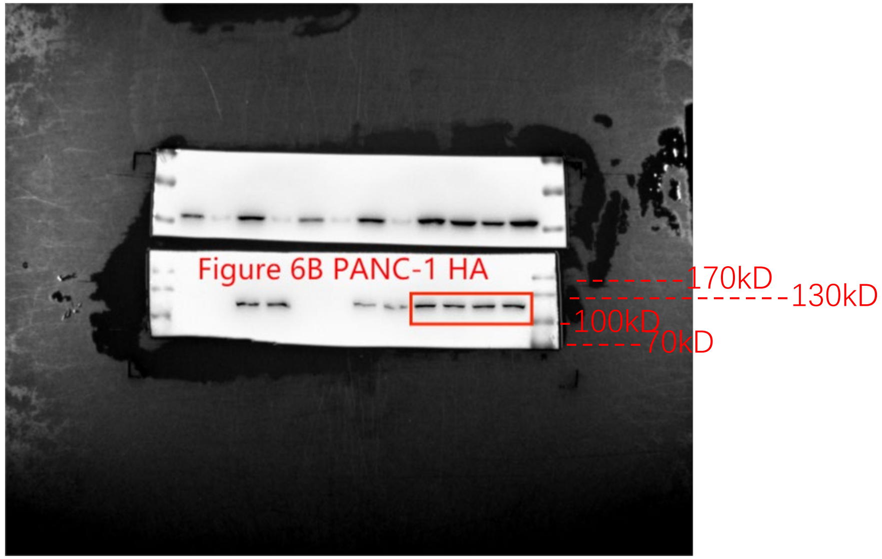

Figure 6B PANC-1 GAPDH

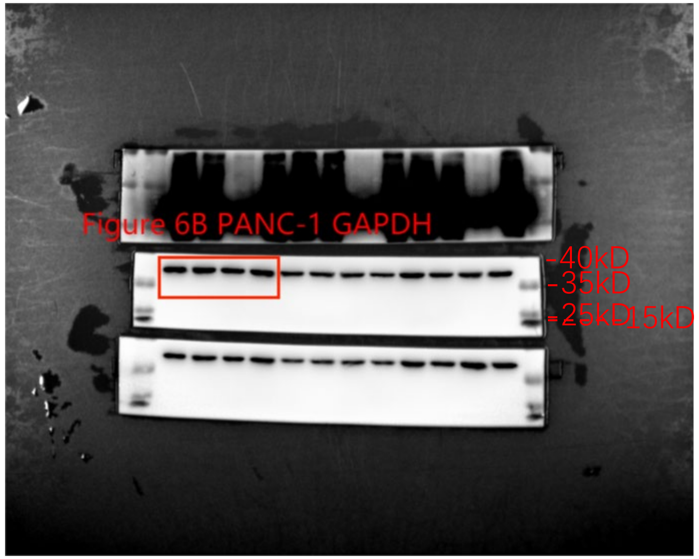

Figure 6B PANC-1 IP O-GlcNAc IB Flag

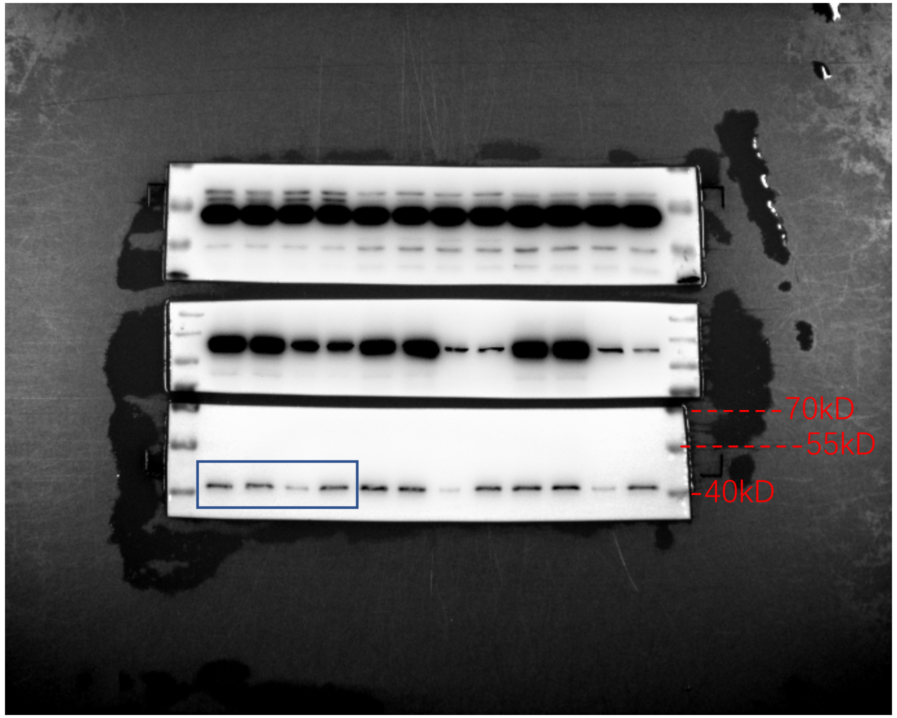

Figure 6B MiaPaCa-2 Flag

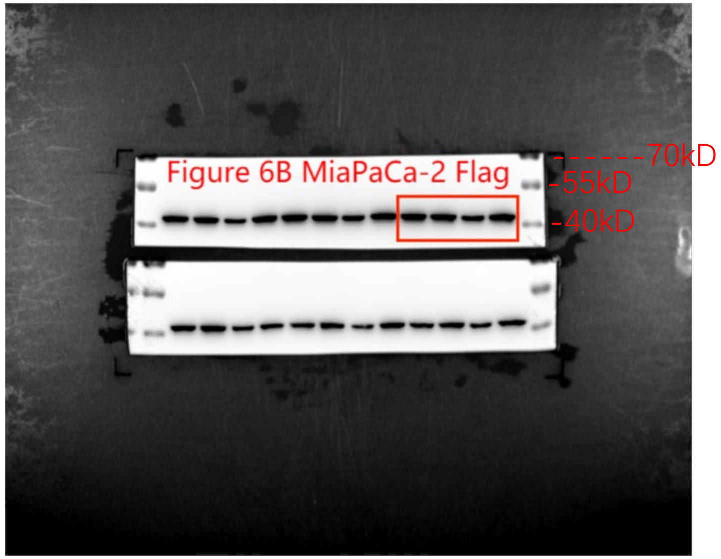

Figure 6B MiaPaCa-2 HA

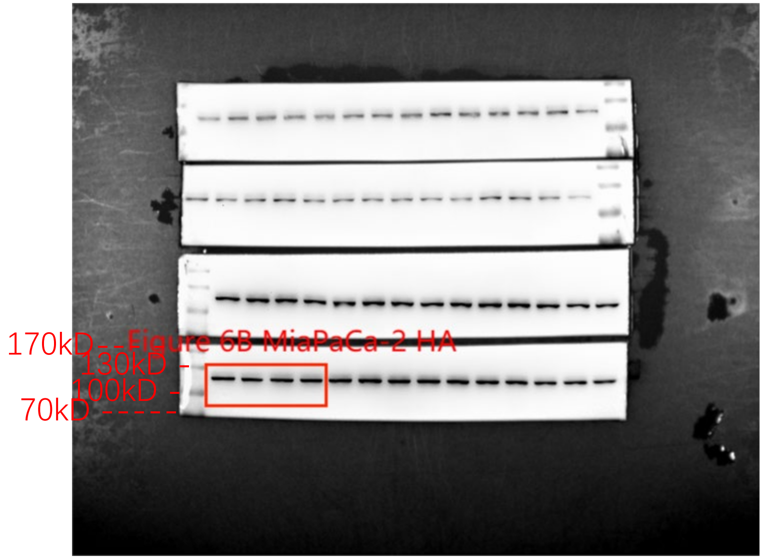

Figure 6B MiaPaCa-2 GAPDH

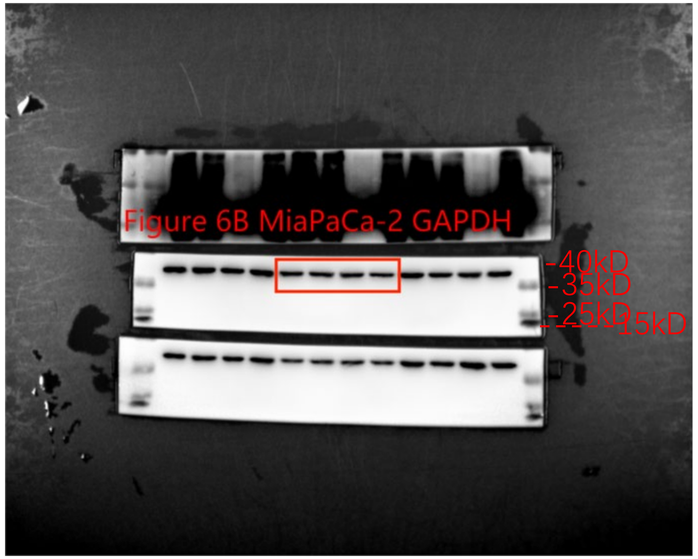

Figure 6B MiaPaCa-2 IP O-GlcNAc IB Flag

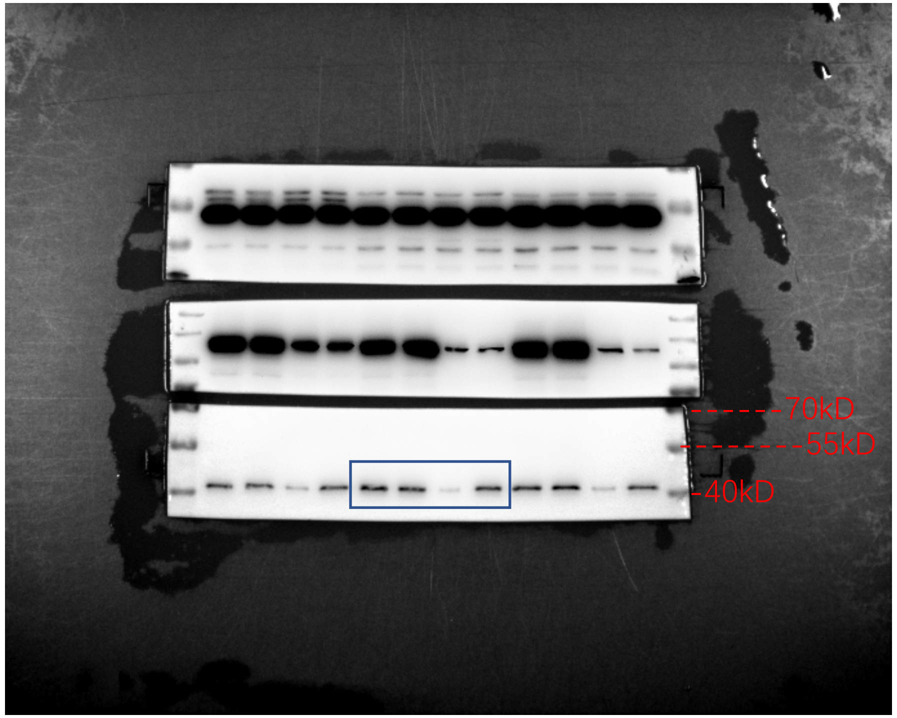

Figure 6B BxPC-3 Flag

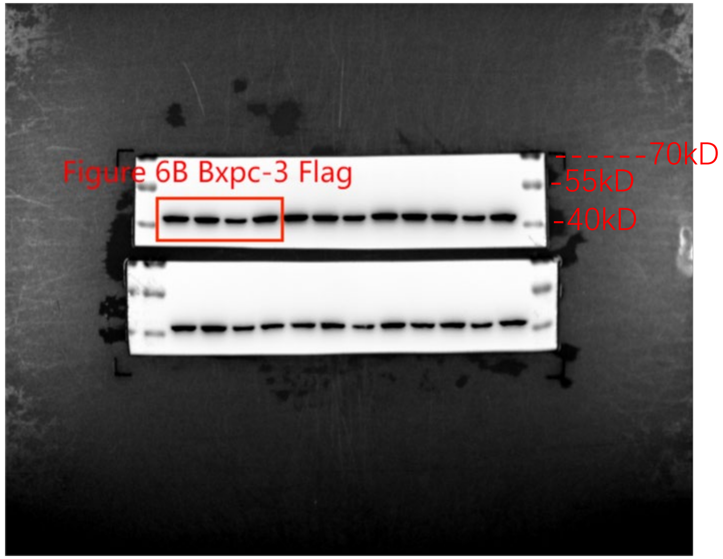

Figure 6B BxPC-3 HA

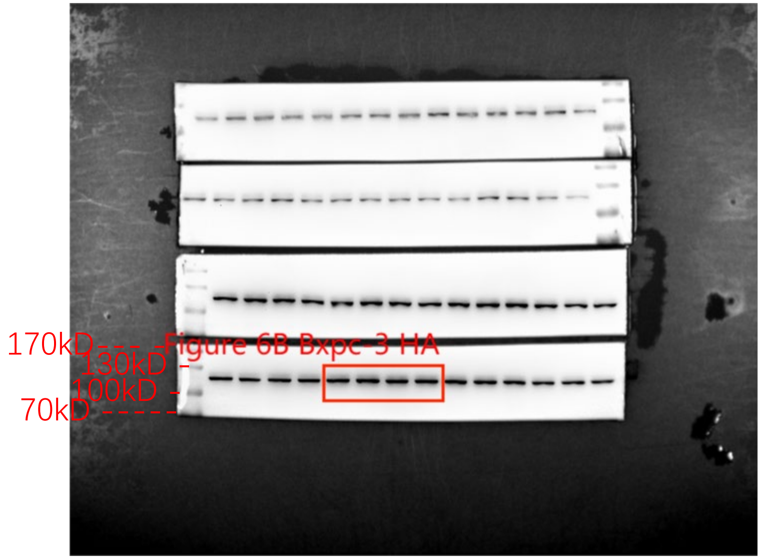

Figure 6B BxPC-3 GAPDH

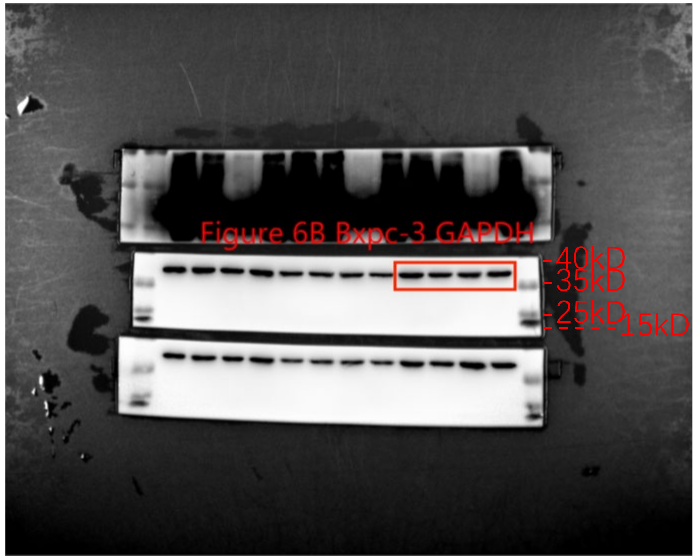

Figure 6B BxPC-3 IP O-GlcNAc IB Flag

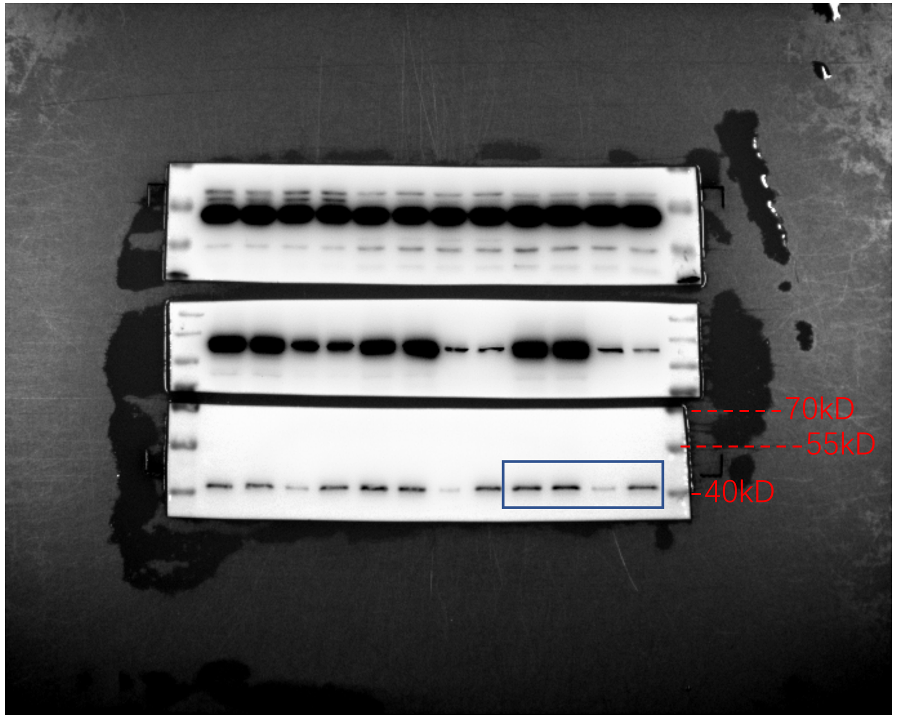

Figure 6D PANC-1 WT Flag

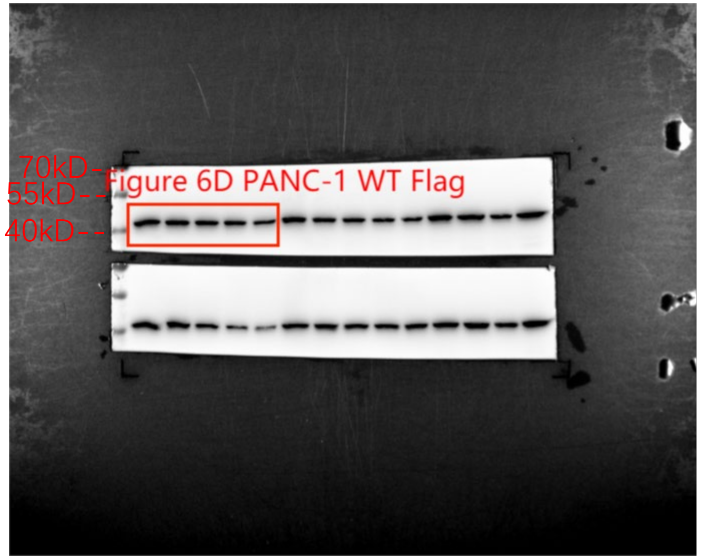

Figure 6D PANC-1 WT GAPDH

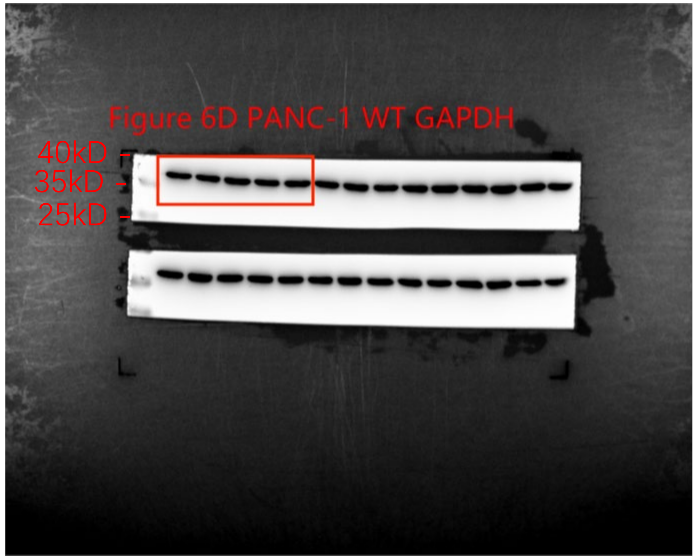

Figure 6D PANC-1 S134A Flag

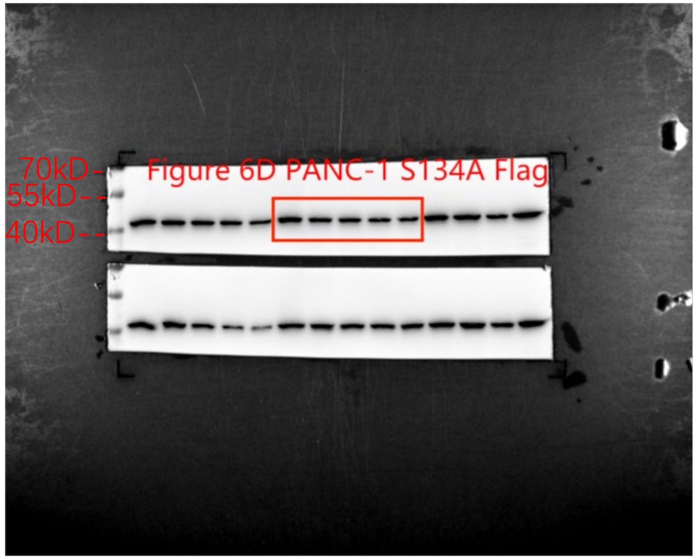

Figure 6D PANC-1 S134A GAPDH

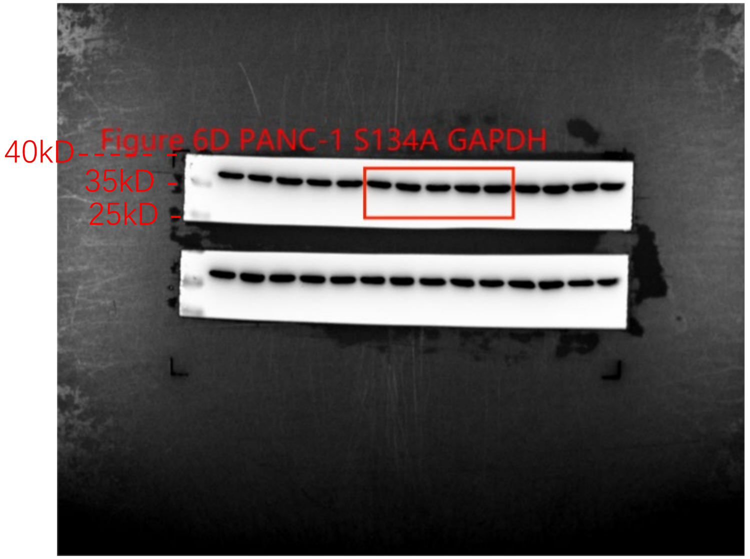

Figure 6D PANC-1 S136A Flag

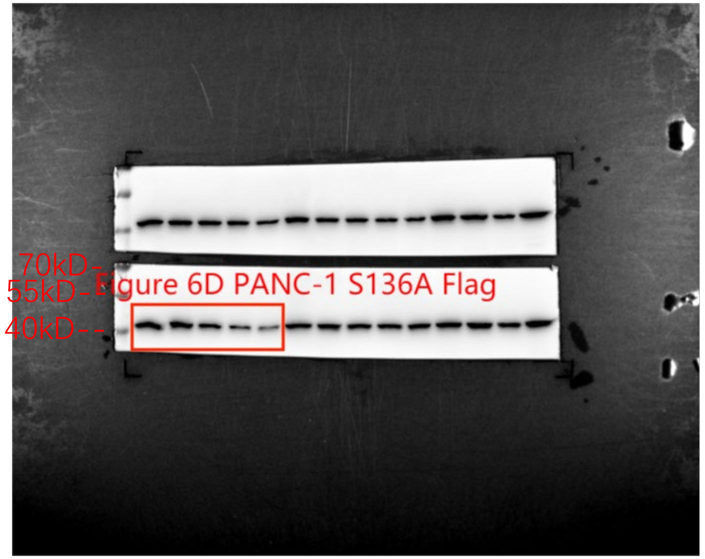

Figure 6D PANC-1 S136A GAPDH

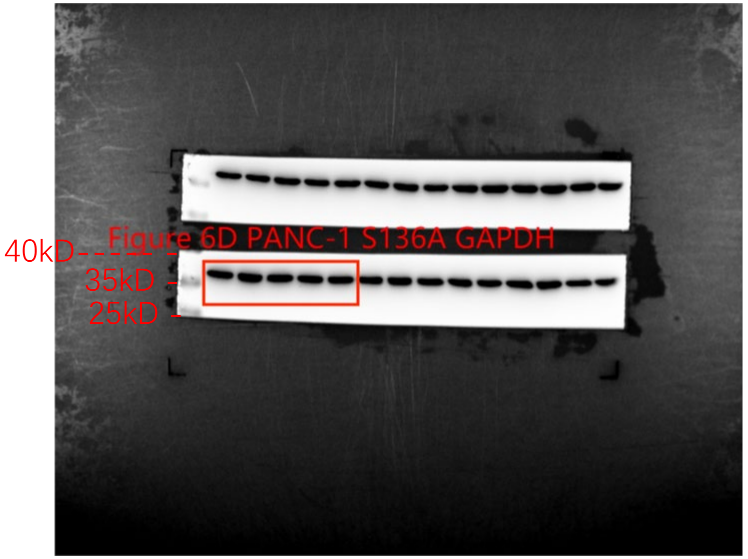

Figure 6D PANC-1 S377A Flag

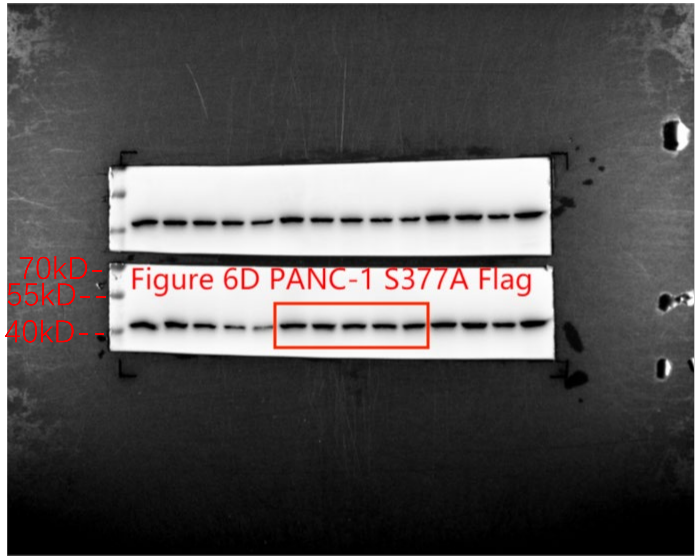

Figure 6D PANC-1 S377A GAPDH

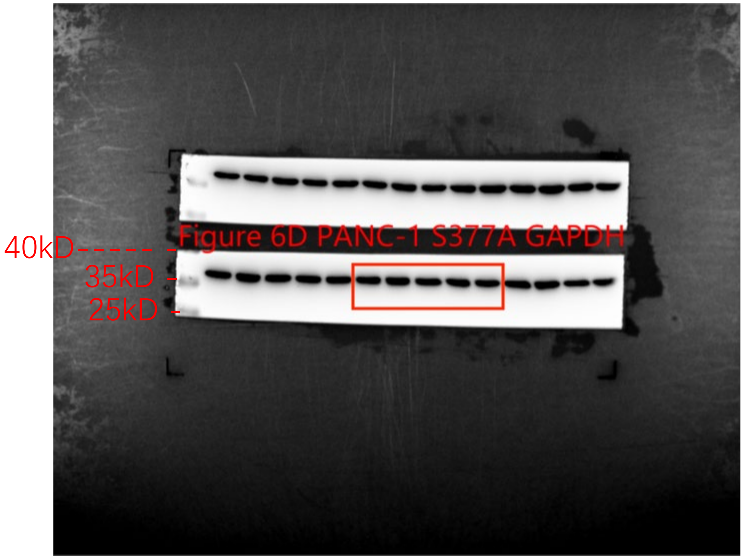

Figure 6D MiaPaCa-2 WT Flag

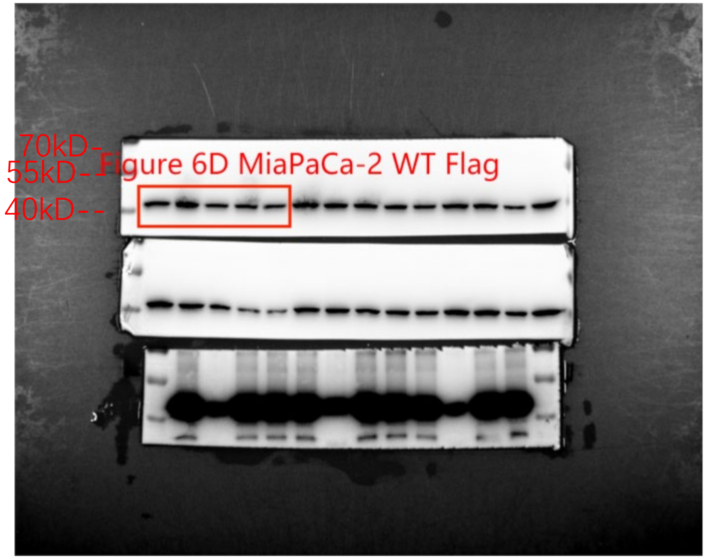

Figure 6D MiaPaCa-2 WT GAPDH

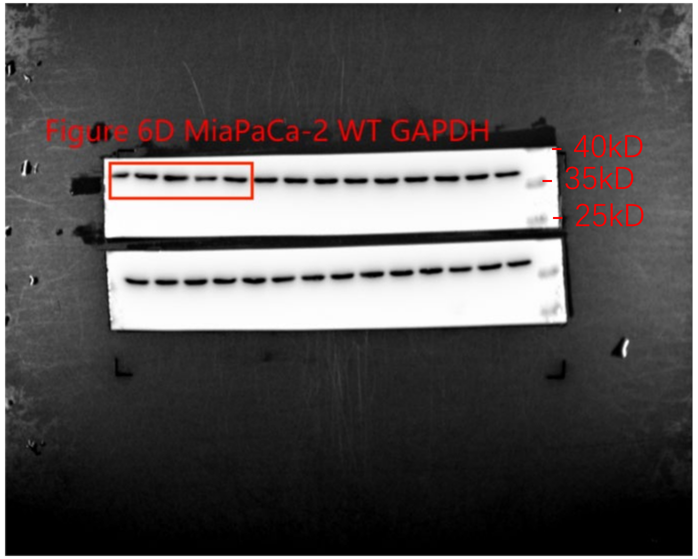

Figure 6D MiaPaCa-2 S134A Flag

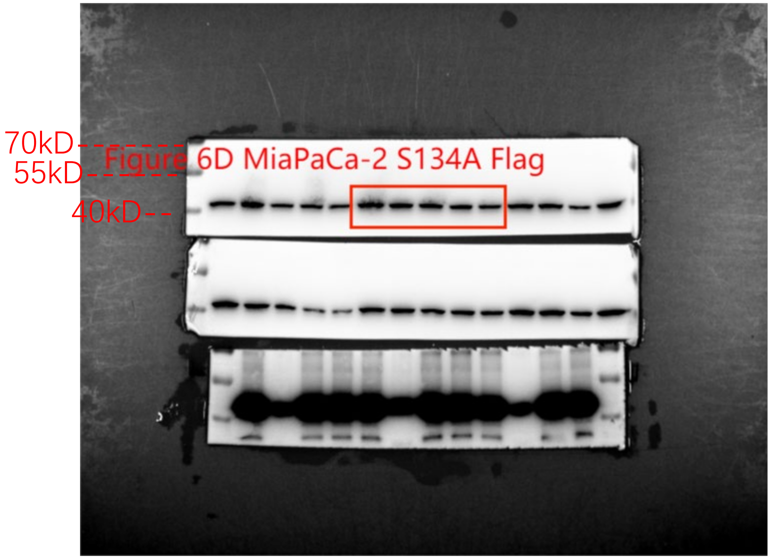

Figure 6D MiaPaCa-2 S134A GAPDH

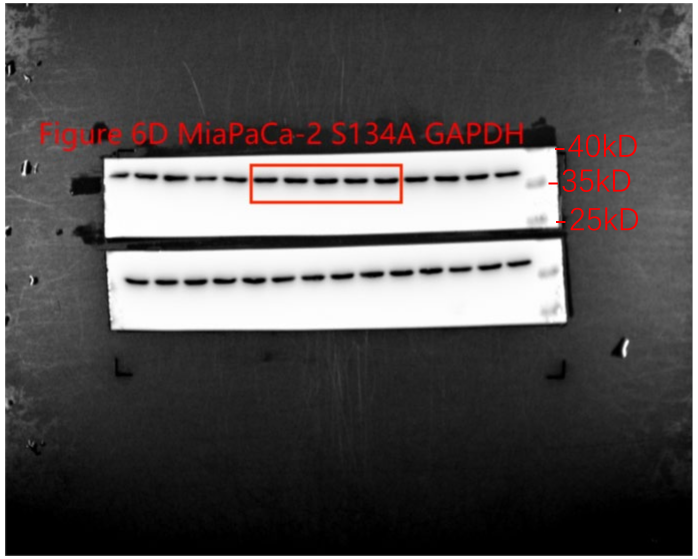

Figure 6D MiaPaCa-2 S136A Flag

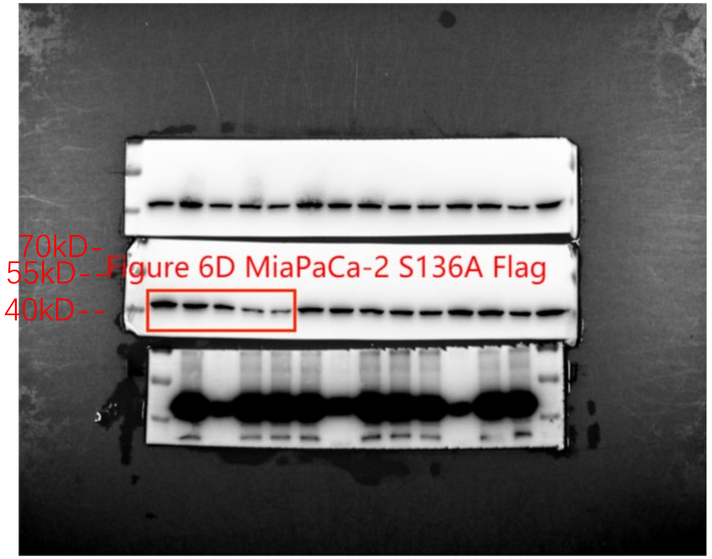

Figure 6D MiaPaCa-2 S136A GAPDH

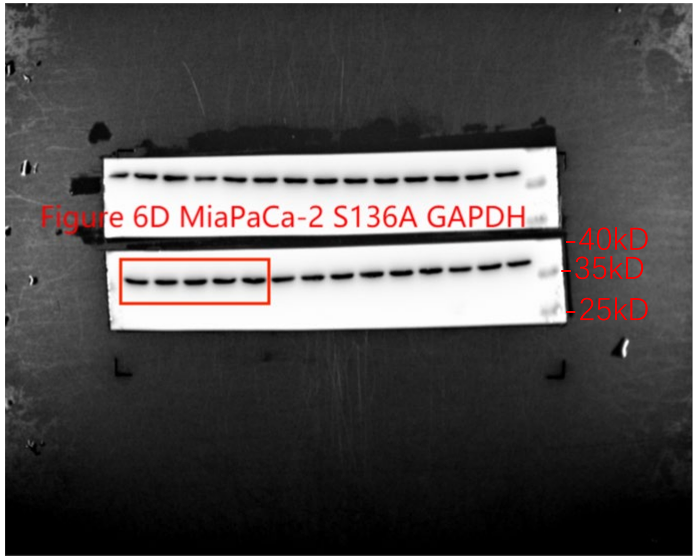

Figure 6D MiaPaCa-2 S377A Flag

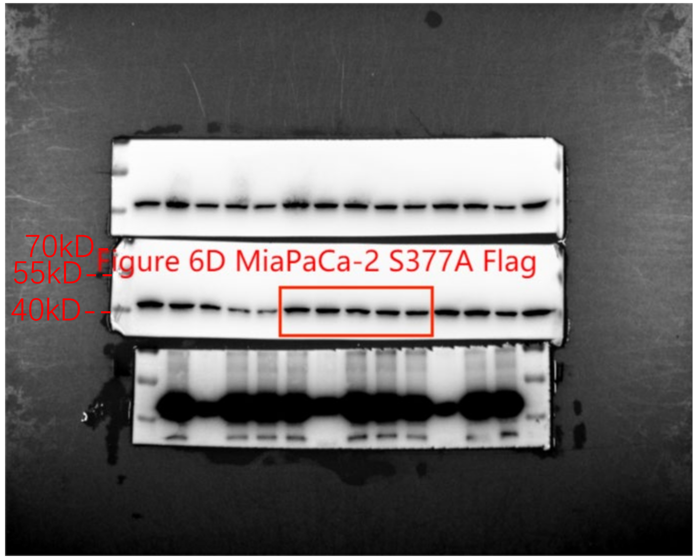

Figure 6D MiaPaCa-2 S377A GAPDH

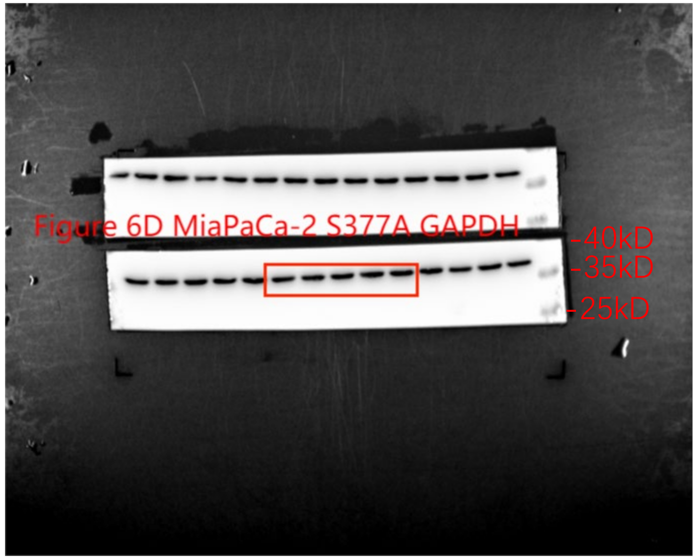

Figure 6D BxPC-3 WT Flag

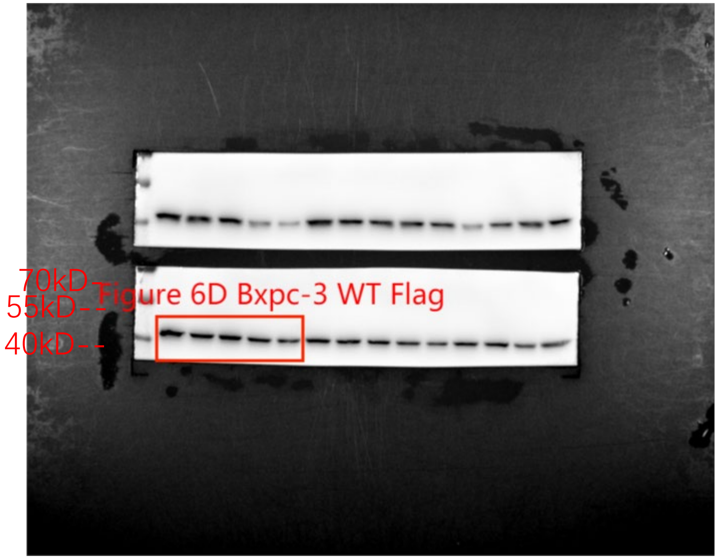

Figure 6D BxPC-3 WT GAPDH

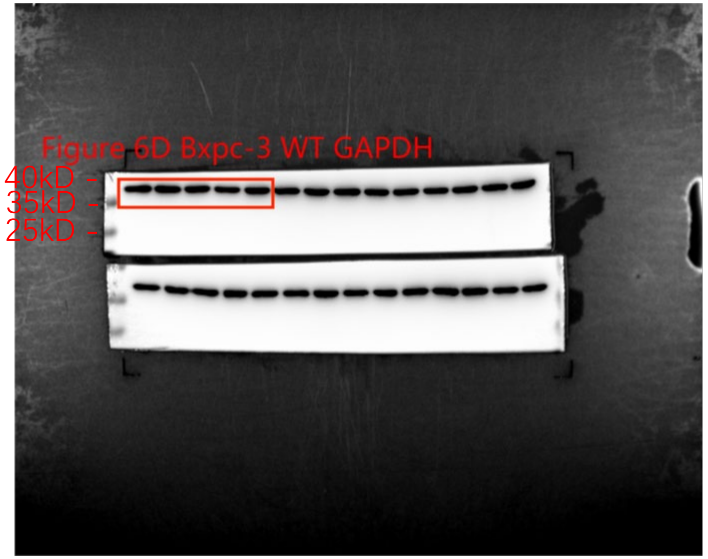

Figure 6D BxPC-3 S134A Flag

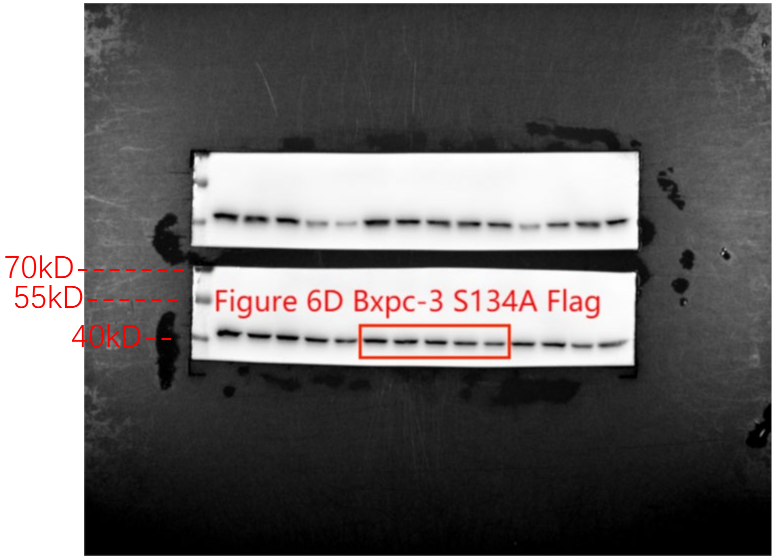

Figure 6D BxPC-3 S134A GAPDH

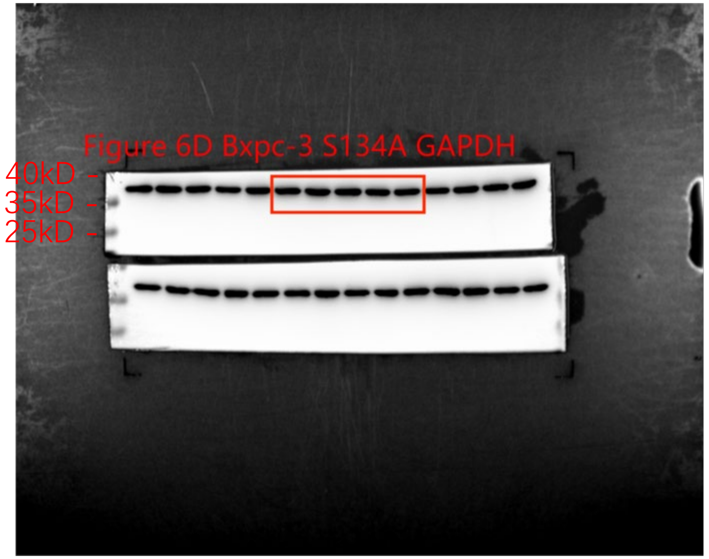

Figure 6D BxPC-3 S136A Flag

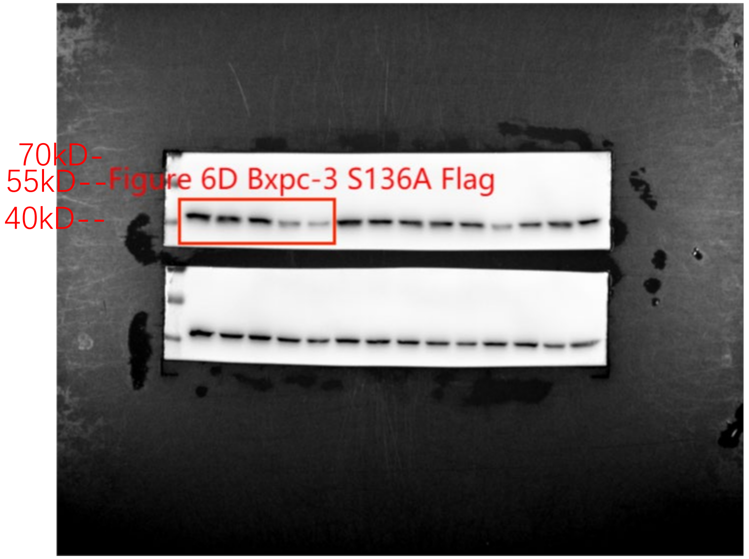

Figure 6D BxPC-3 S136A GAPDH

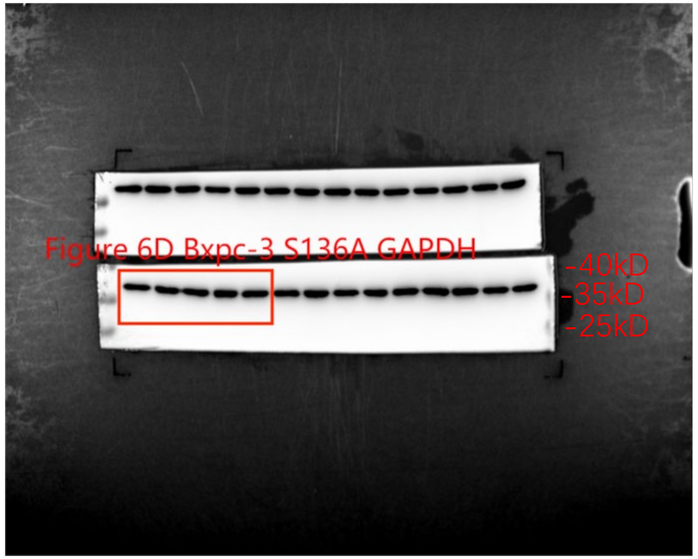

Figure 6D BxPC-3 S377A Flag

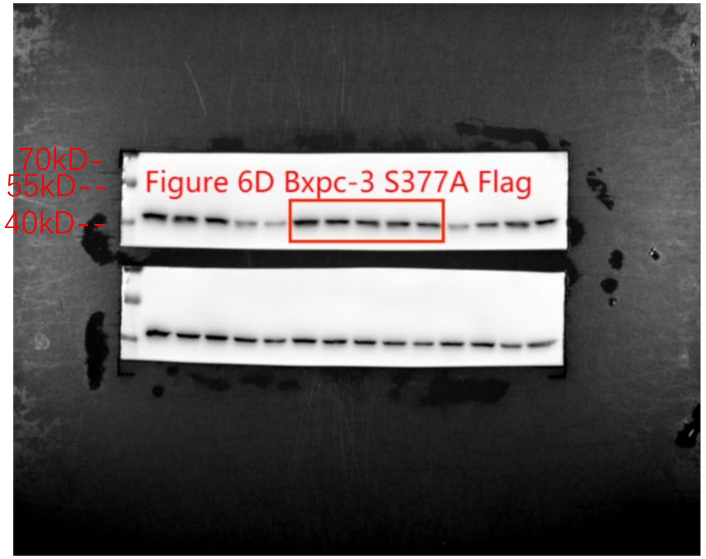

Figure 6D BxPC-3 S377A GAPDH

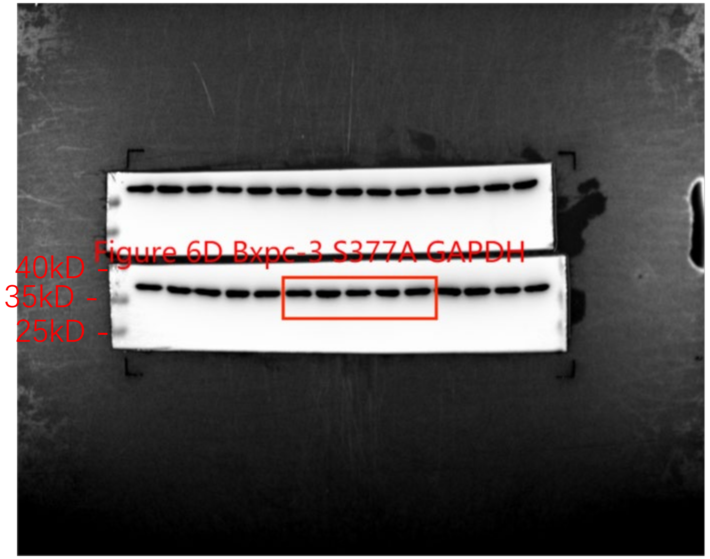

Figure 6E PANC-1 GAPDH

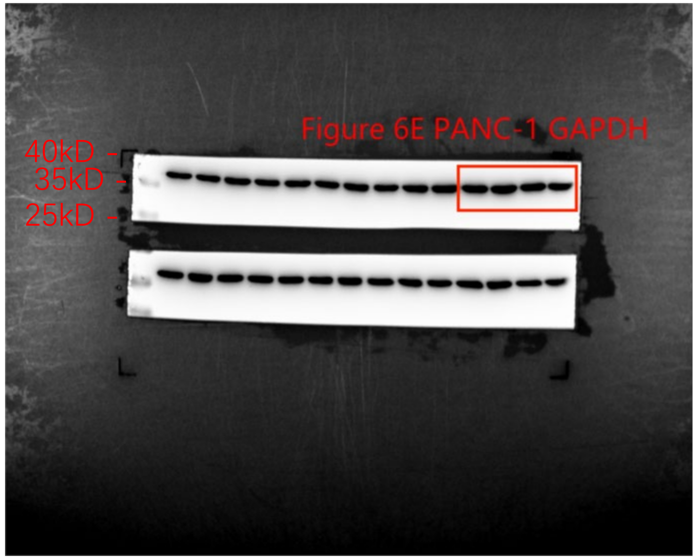

Figure 6E PANC-1 Flag

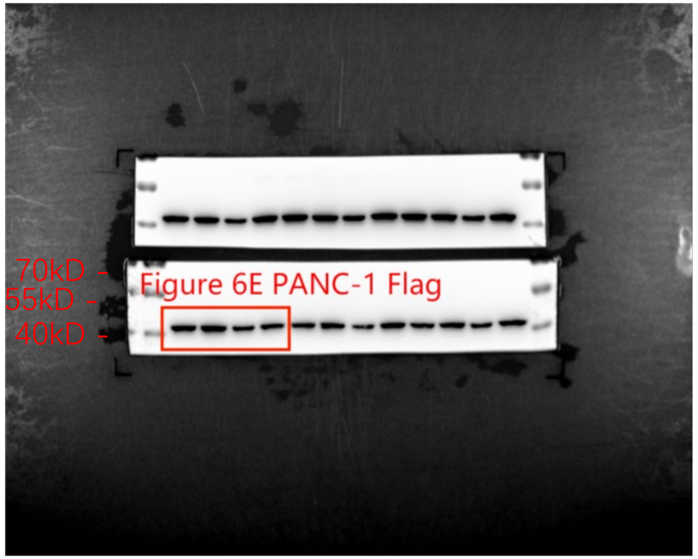

Figure 6E PANC-1 REGγ

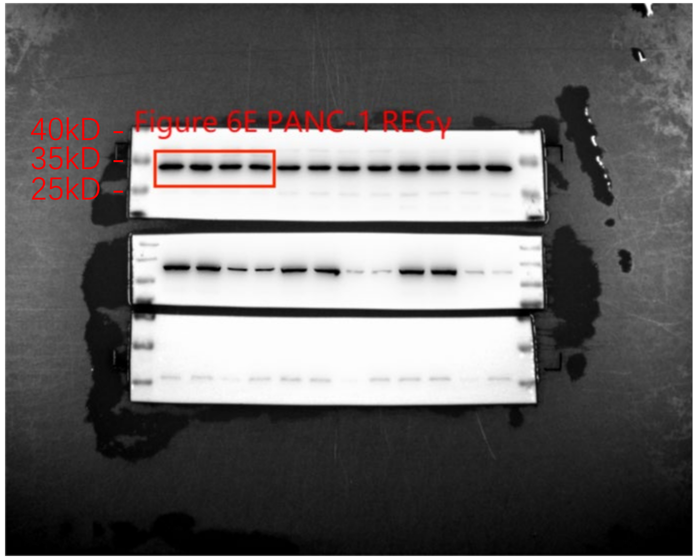

Figure 6E PANC-1 IP SIRT7 IB REGγ

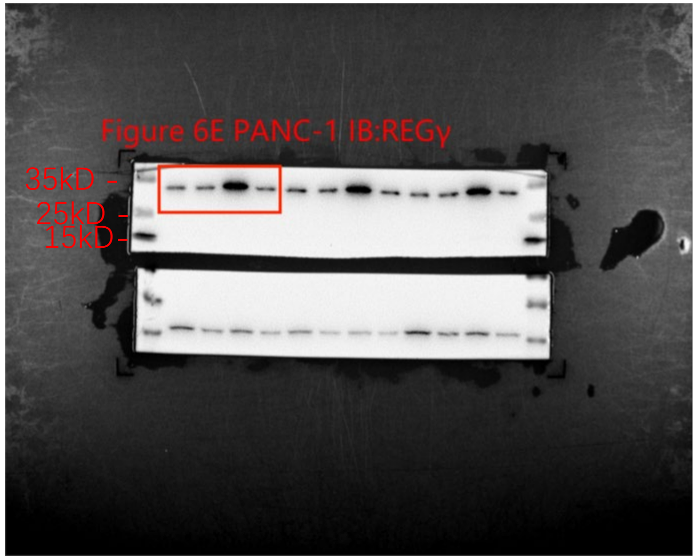

Figure 6E MiaPaCa-2 GAPDH

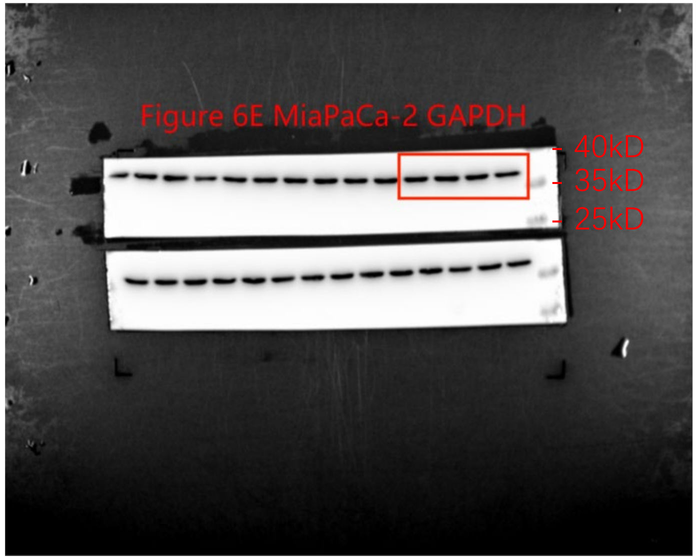

Figure 6E MiaPaCa-2 Flag

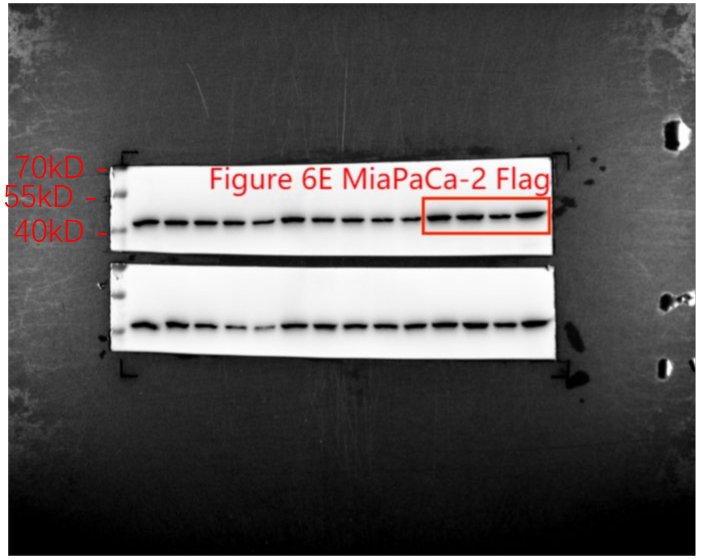

Figure 6E MiaPaCa-2 REGγ

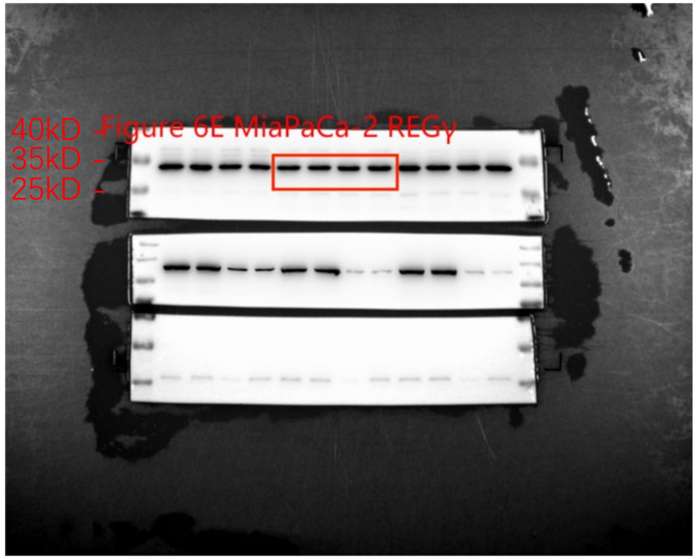

Figure 6E MiaPaCa-2 IP SIRT7 IB REGγ

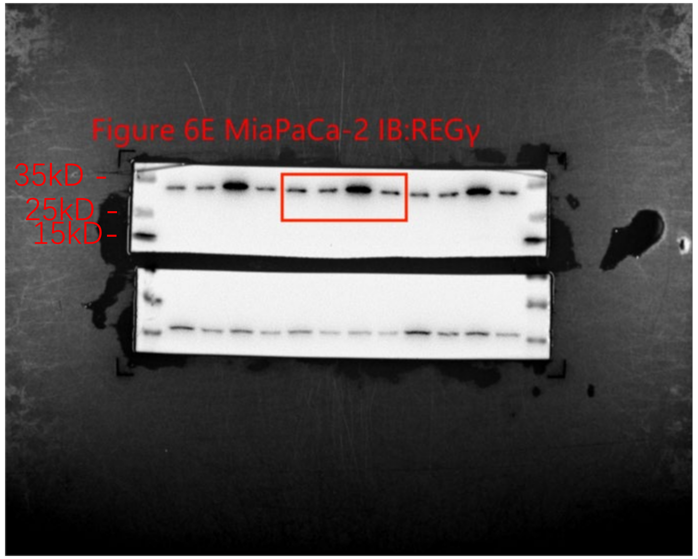

Figure 6E BxPC-3 GAPDH

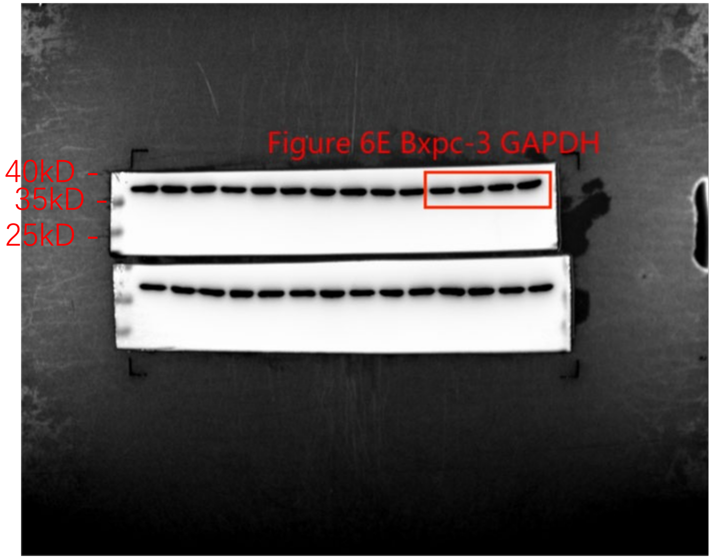

Figure 6E BxPC-3 Flag

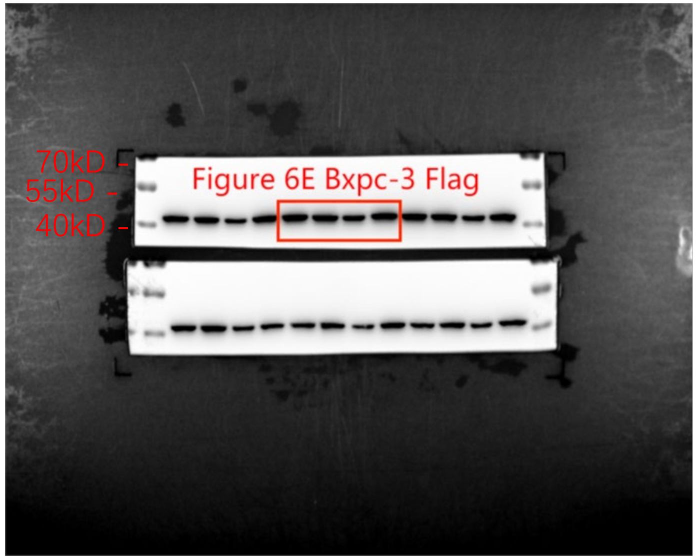

Figure 6E BxPC-3 REGγ

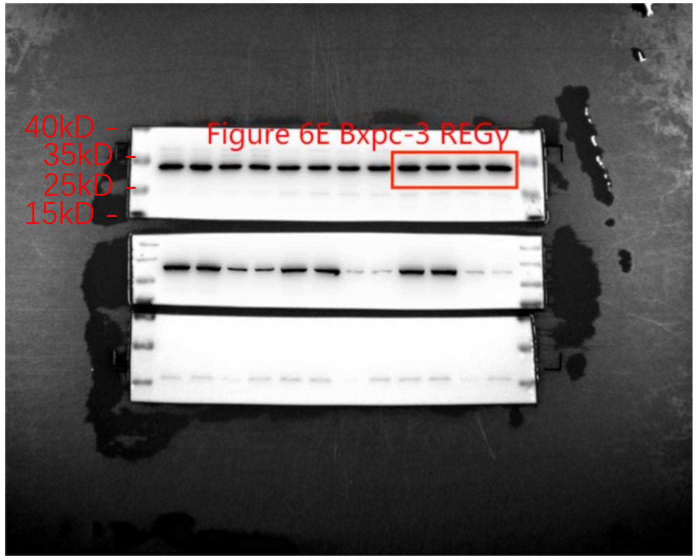

Figure 6E BxPC-3 IP SIRT7 IB REGγ

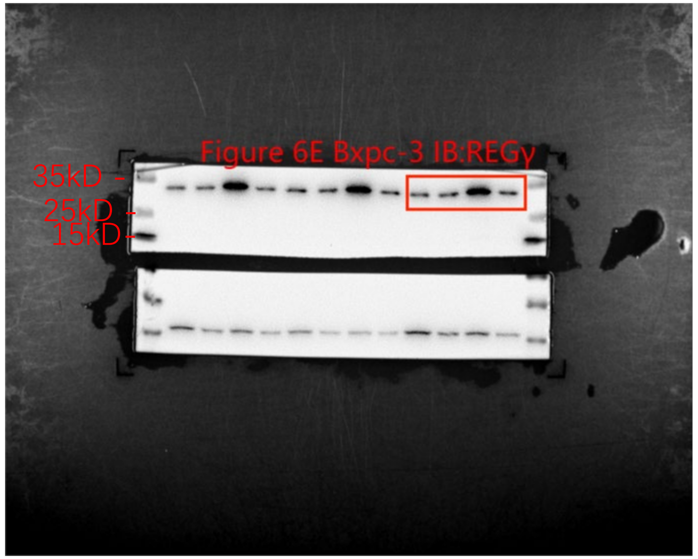

Figure 6F PANC-1 Flag

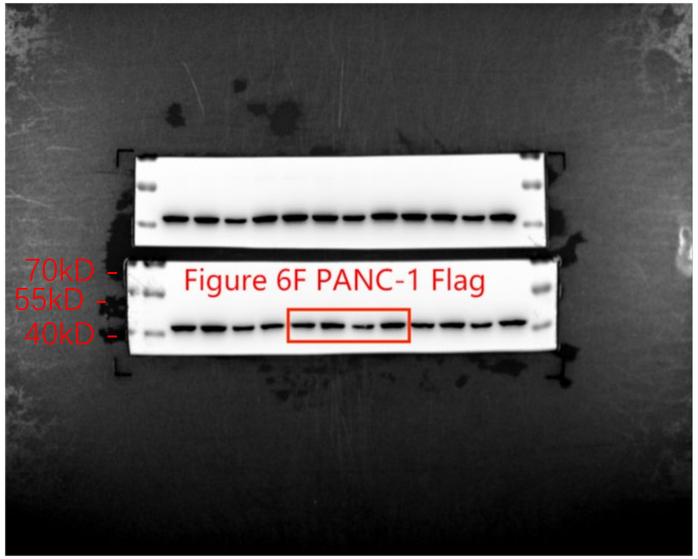

Figure 6F PANC-1 GAPDH

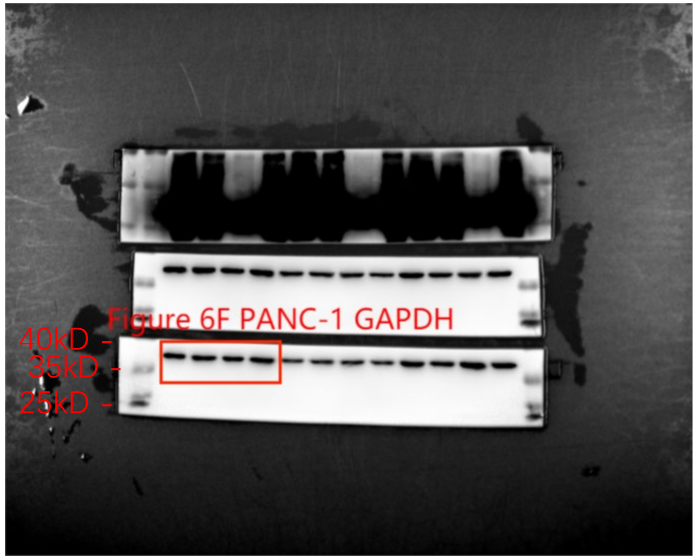

Figure 6F PANC-1 H3

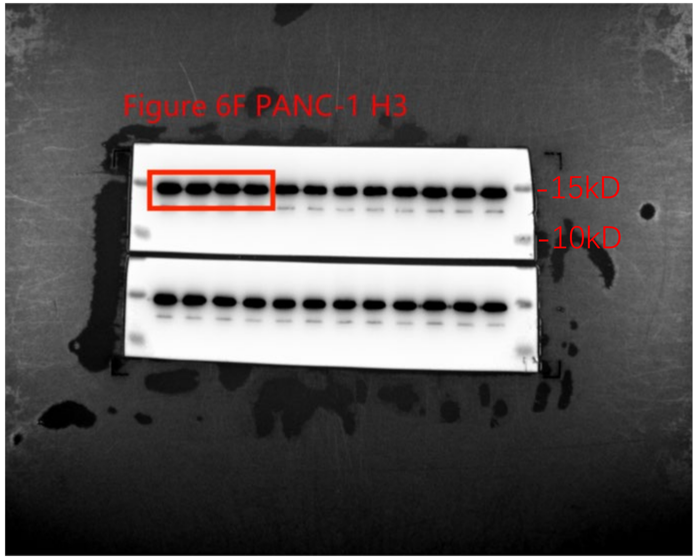

Figure 6F PANC-1 H3K18Ac

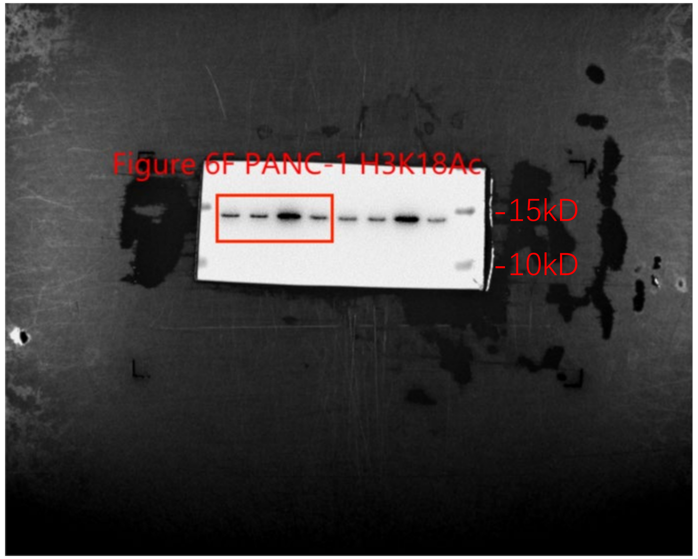

Figure 6F MiaPaCa-2 Flag

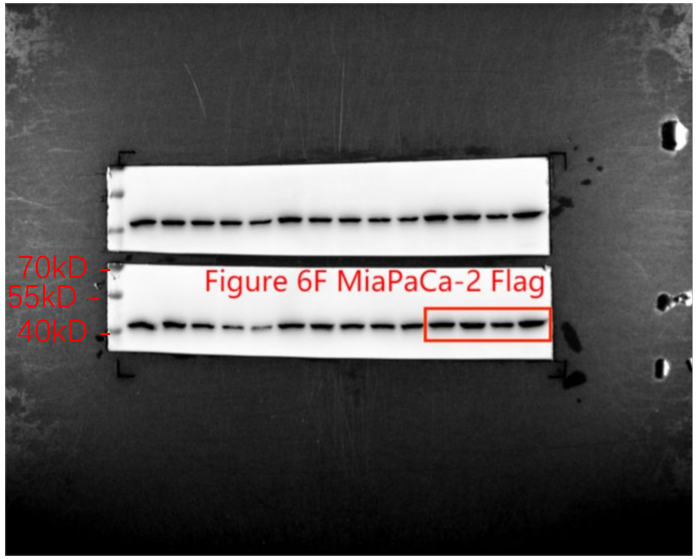

Figure 6F MiaPaCa-2 GAPDH

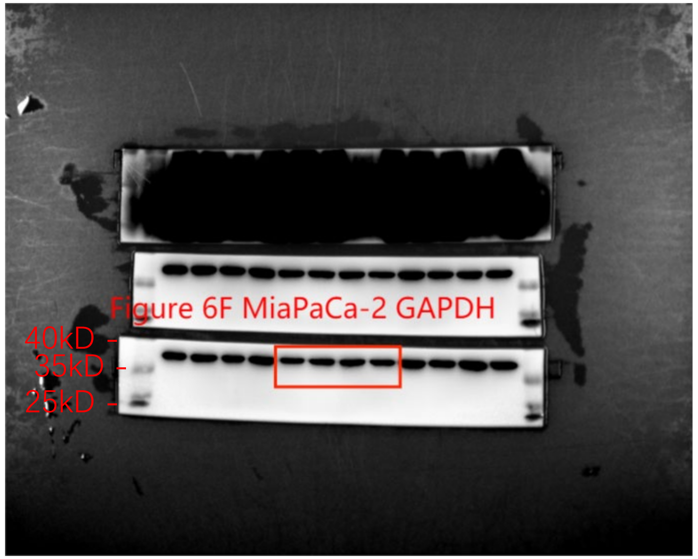

Figure 6F MiaPaCa-2 H3

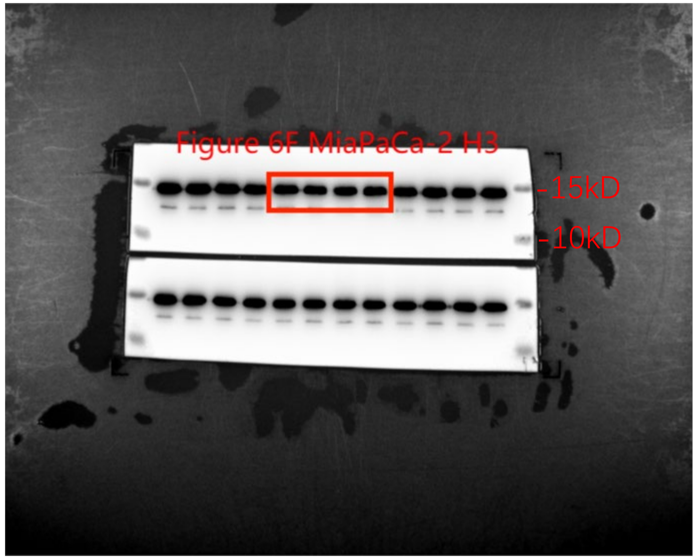

Figure 6F MiaPaCa-2 H3K18Ac

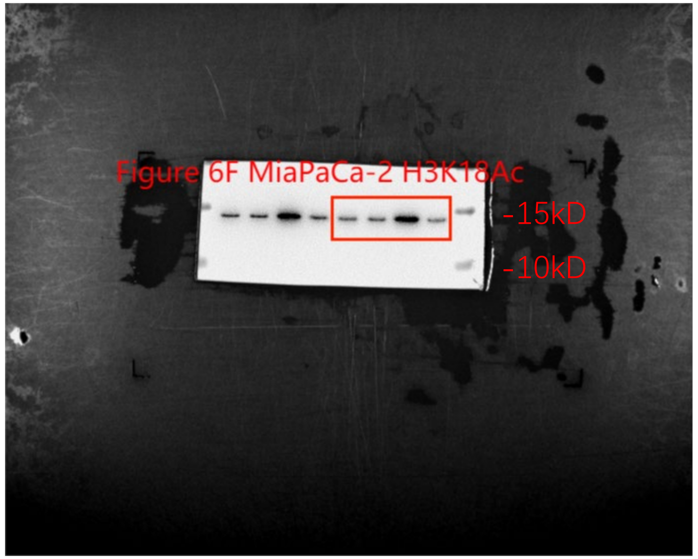

Figure 6F BxPC-3 Flag

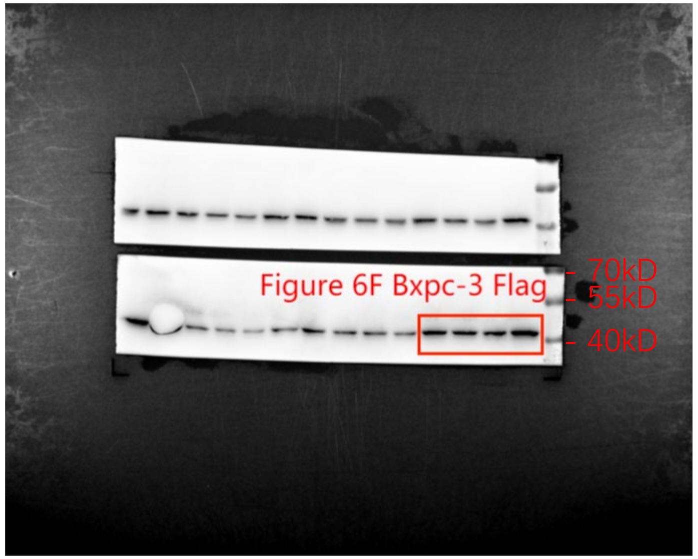

Figure 6F BxPC-3 GAPDH

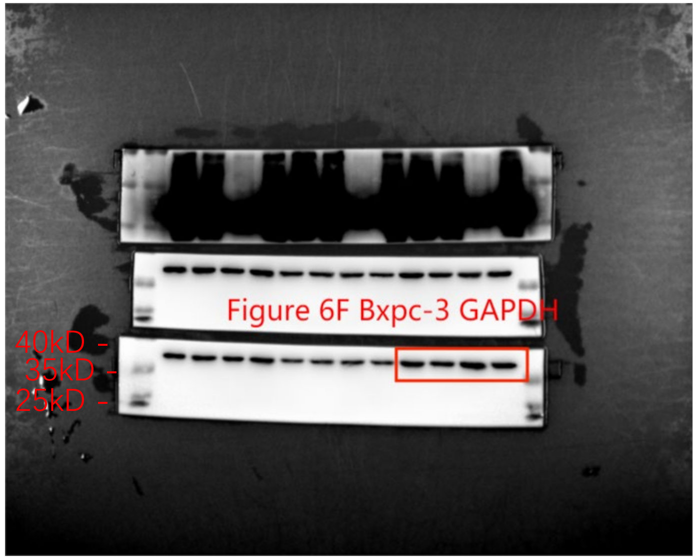

Figure 6F BxPC-3 H3

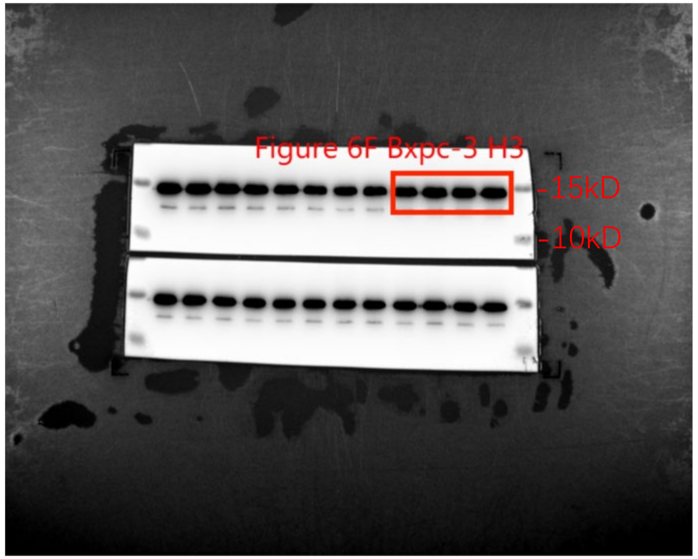

Figure 6F BxPC-3 H3K18Ac

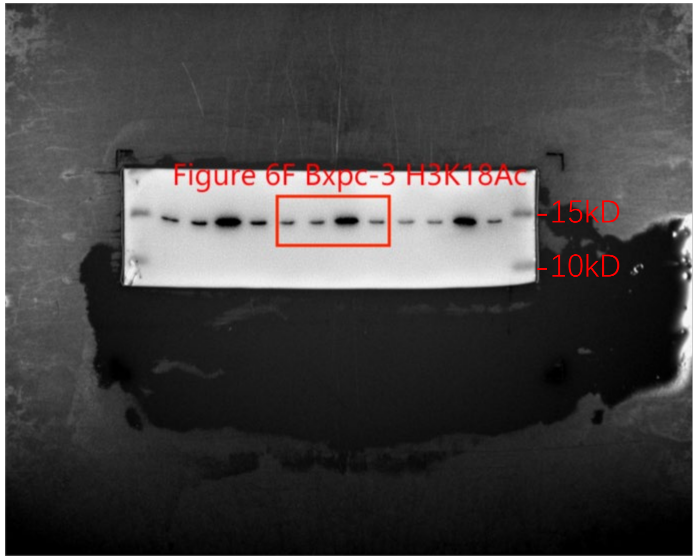

Figure 6G PANC-1 OGT

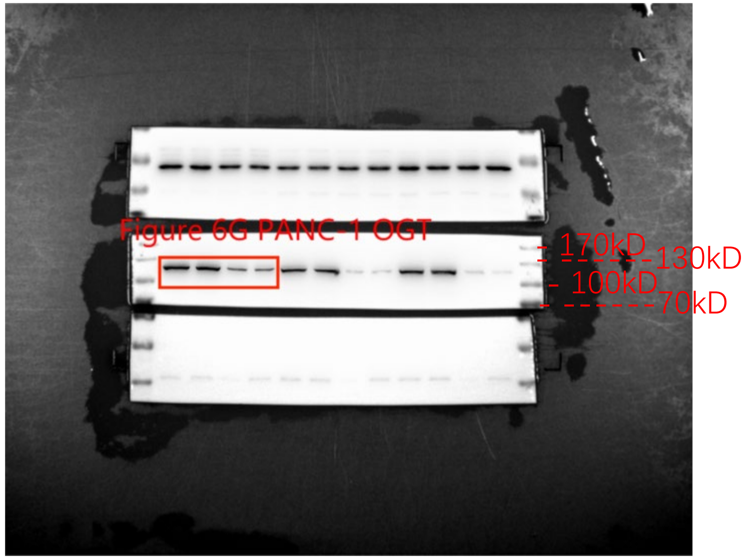

Figure 6G PANC-1 Flag

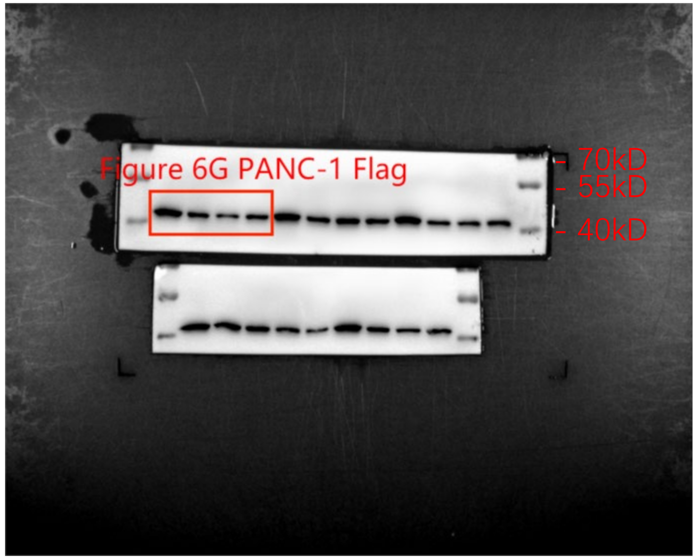

Figure 6G PANC-1 GAPDH

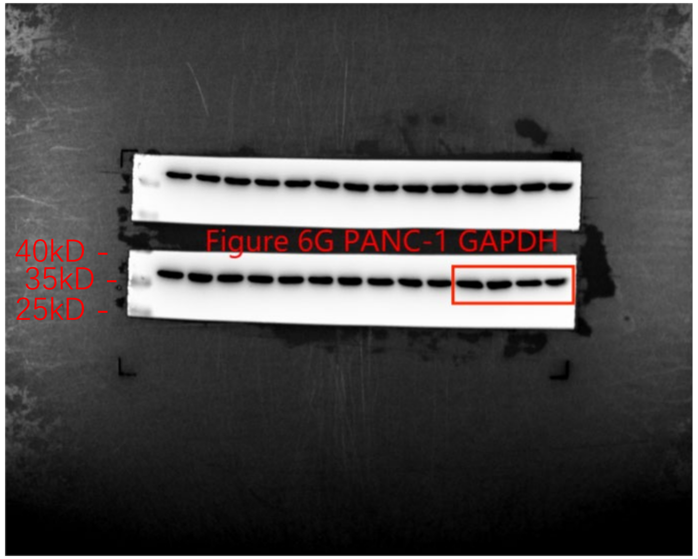

Figure 6G PANC-1 H3

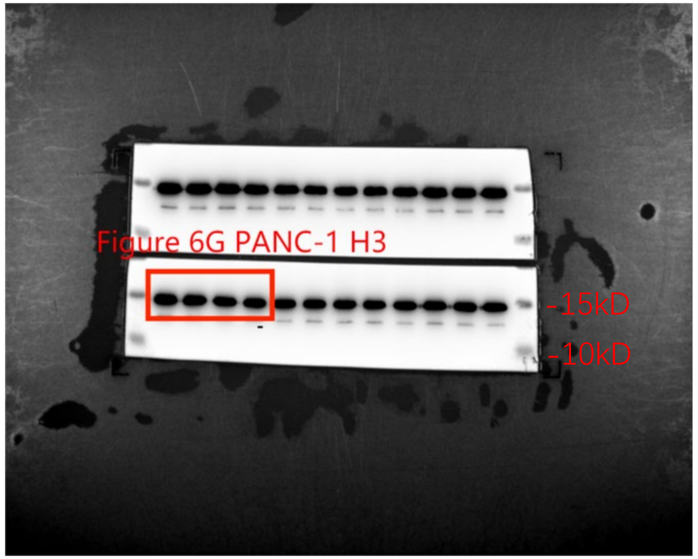

Figure 6G PANC-1 H3K18Ac

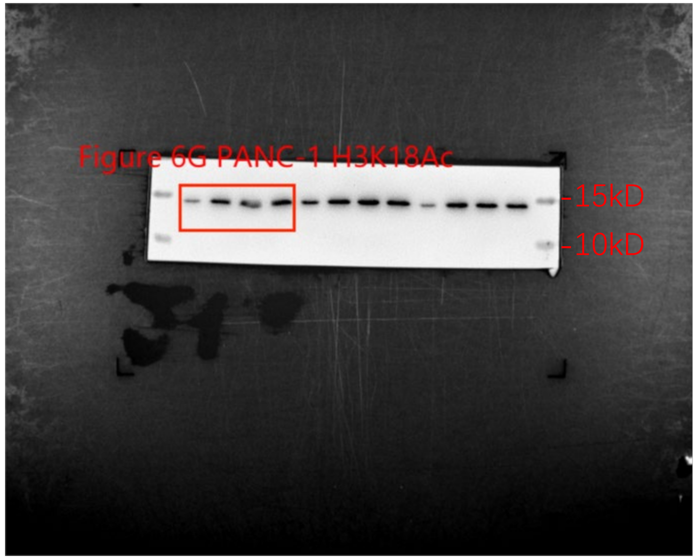

Figure 6G MiaPaCa-2 OGT

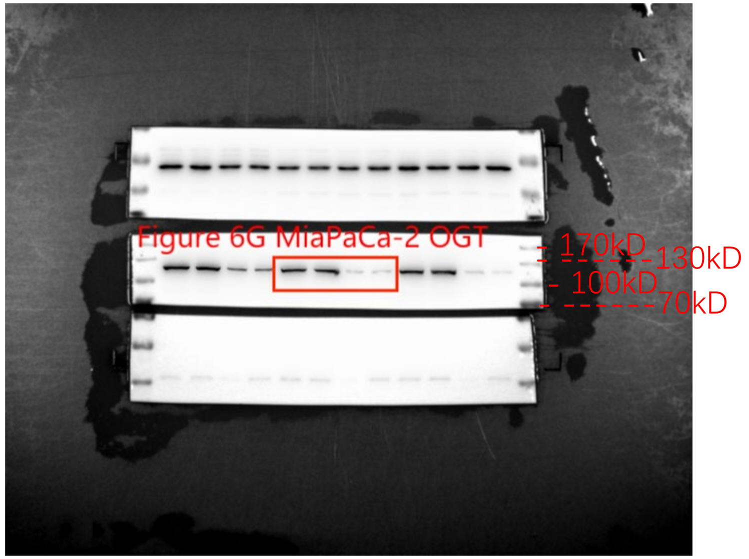

Figure 6G MiaPaCa-2 Flag

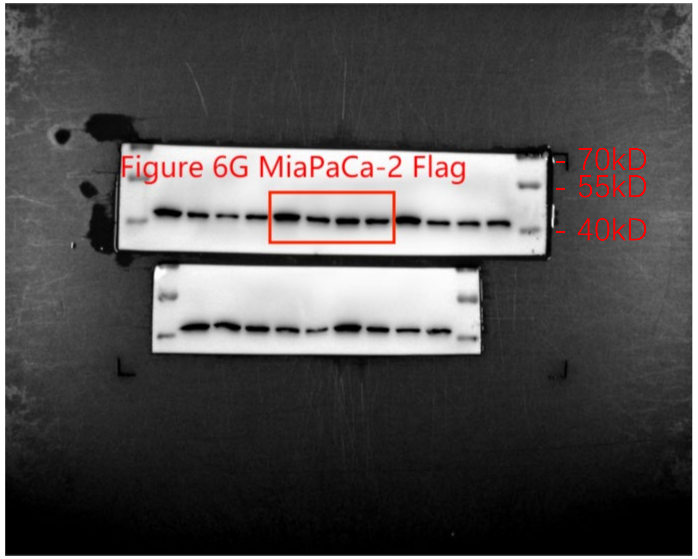

Figure 6G MiaPaCa-2 GAPDH

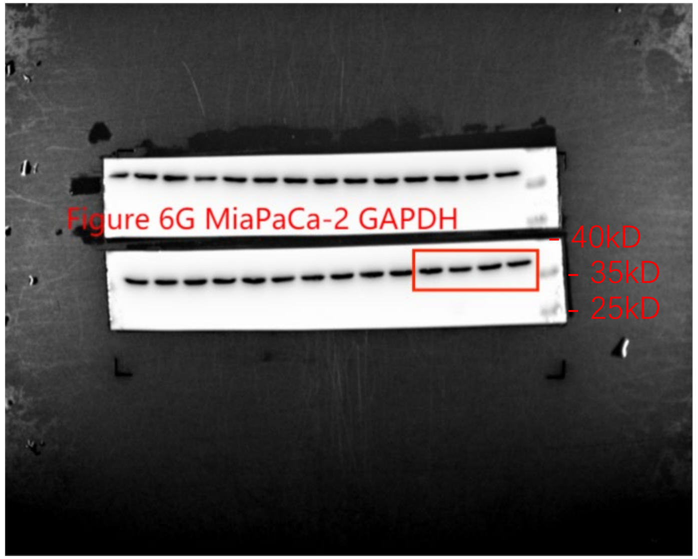

Figure 6G MiaPaCa-2 H3

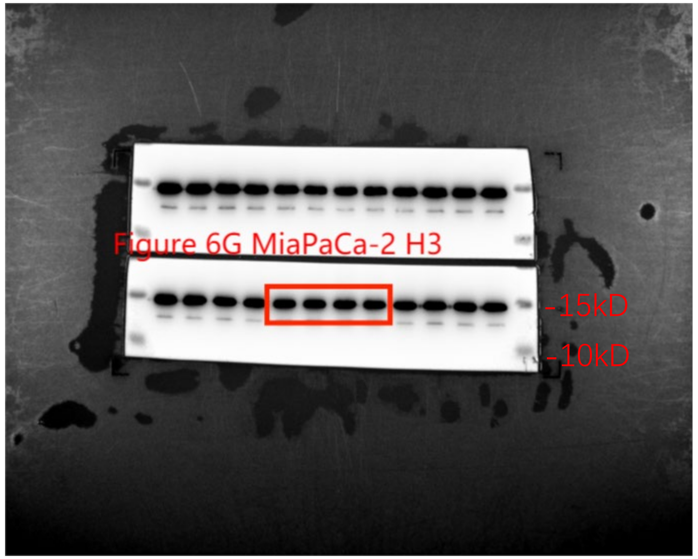

Figure 6G MiaPaCa-2 H3K18Ac

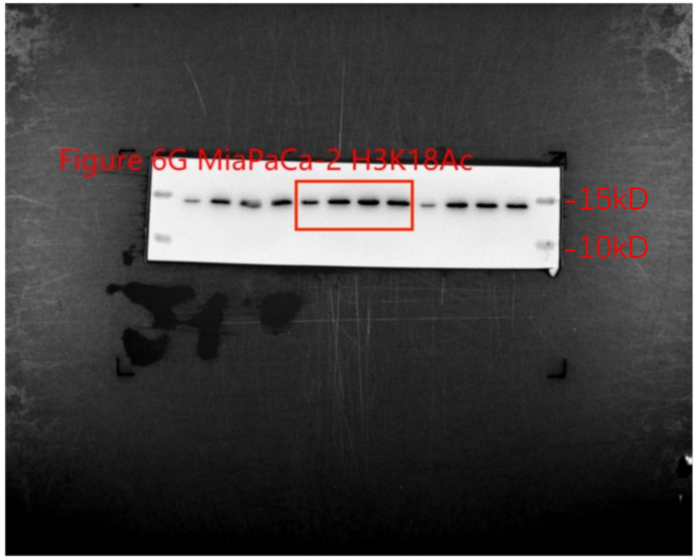

Figure 6G BxPC-3 OGT

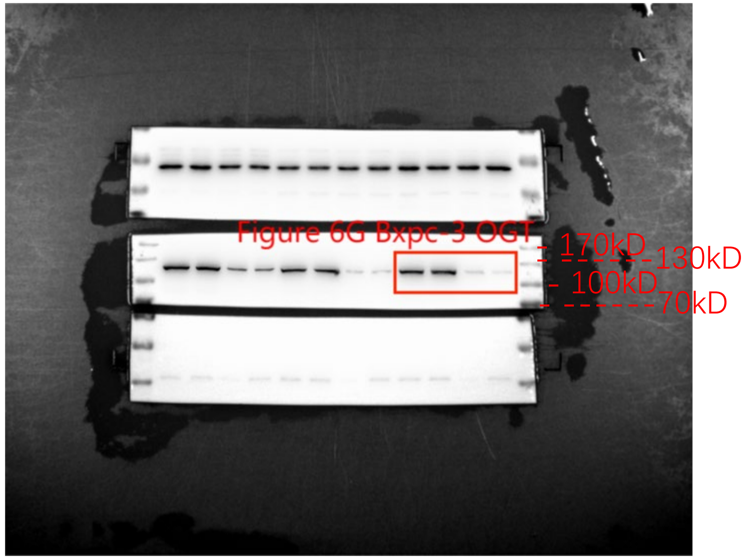

Figure 6G BxPC-3 Flag

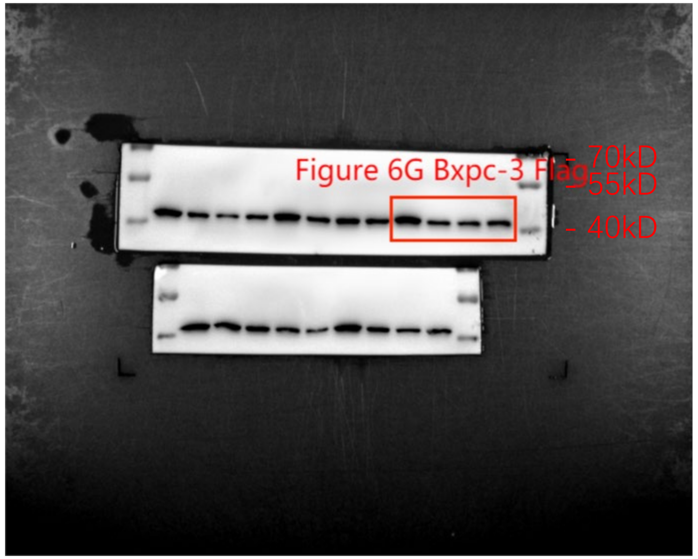

Figure 6G BxPC-3 GAPDH

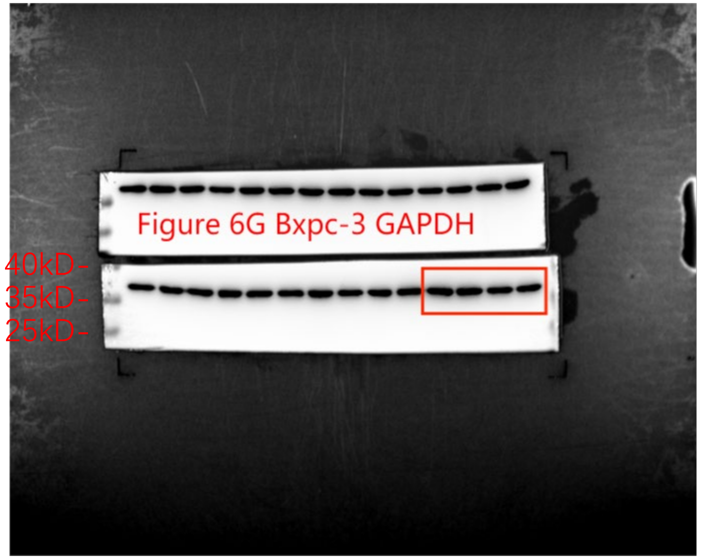

Figure 6G BxPC-3 H3

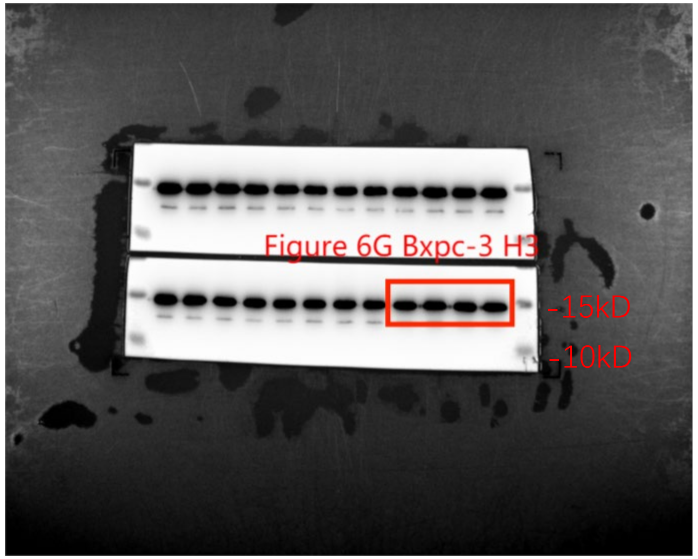

Figure 6G BxPC-3 H3K18Ac

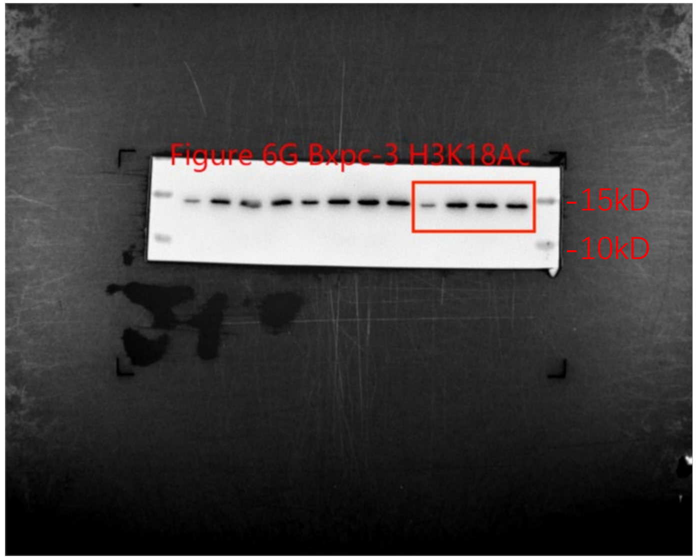

Supplementary Figure 1A SIRT7

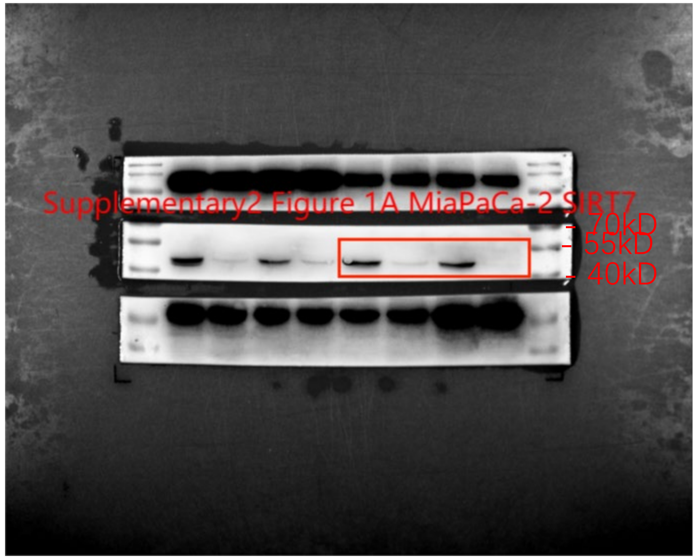

Supplementary Figure 1A GAPDH

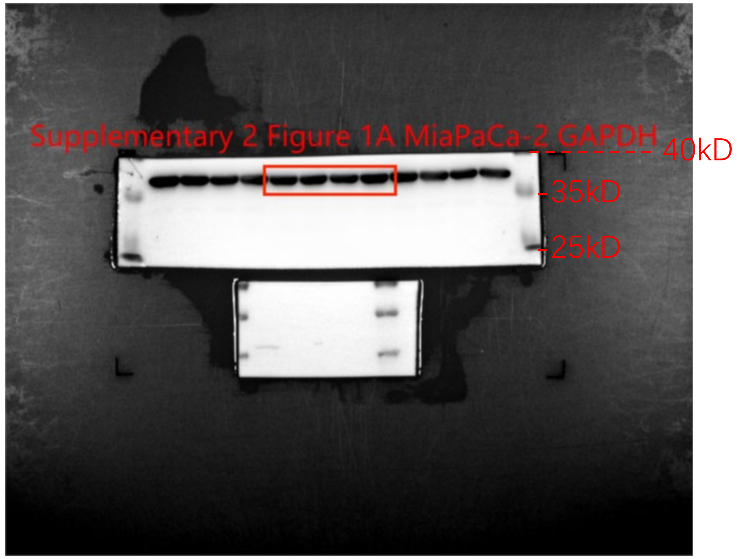

Supplementary Figure 1H SIRT7

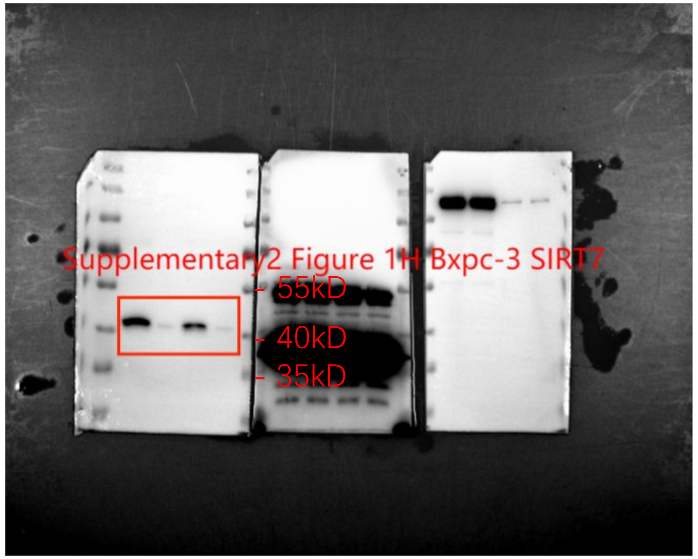

Supplementary Figure 1H GAPDH

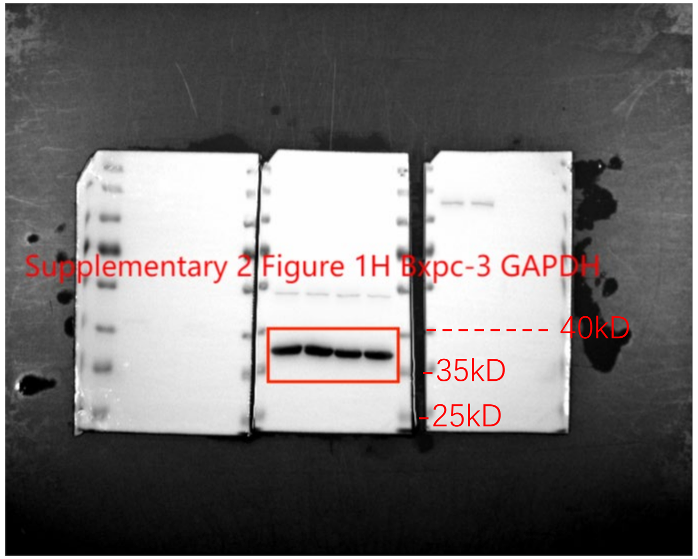

Supplementary Figure 2A O-GlcNAc

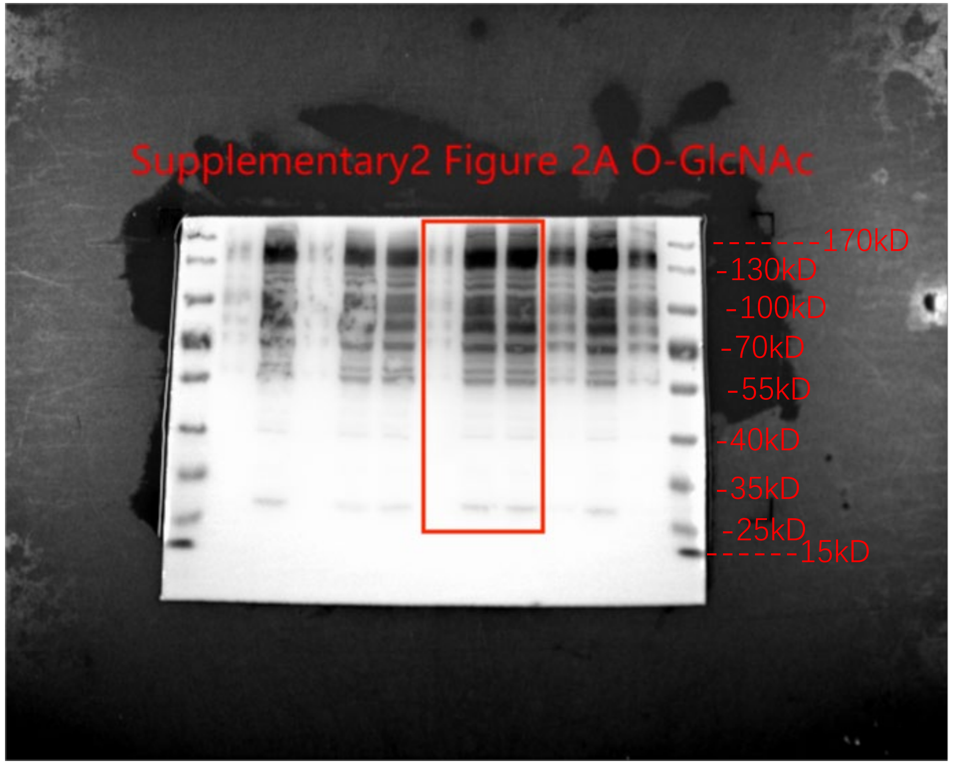

Supplementary Figure 2A SIRT7

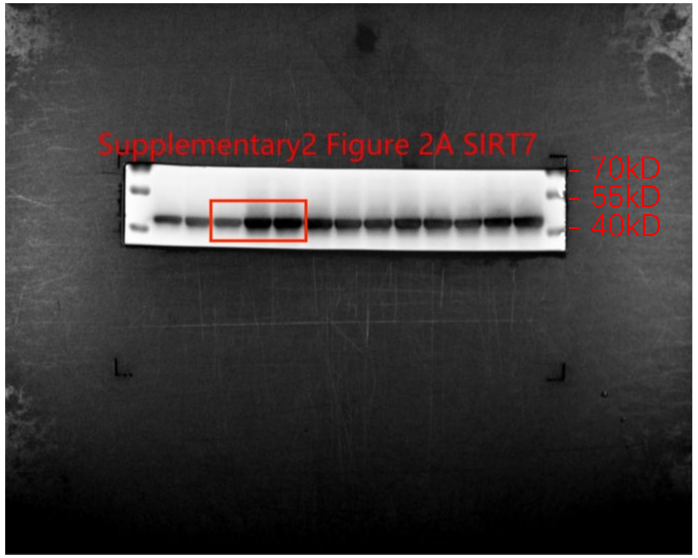

Supplementary Figure 2A GAPDH

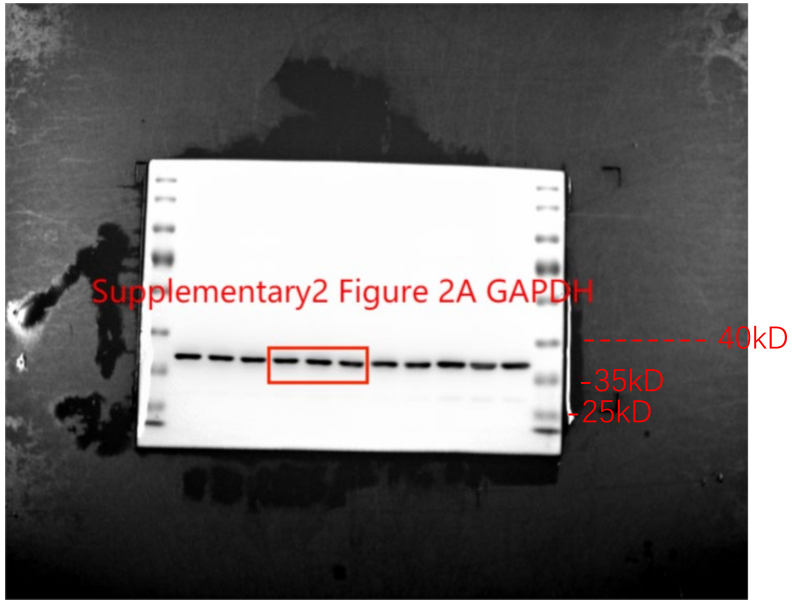

Supplement: Supplementary file 5 — Supplementary original data [file 41418_2022_984_MOESM5_ESM.pdf]
